# Bone Mineral Density Changes among HIV-Uninfected Young Adults in a Randomised Trial of Pre-Exposure Prophylaxis with Tenofovir-Emtricitabine or Placebo in Botswana

**DOI:** 10.1371/journal.pone.0090111

**Published:** 2014-03-13

**Authors:** Michael Kasonde, Richard W. Niska, Charles Rose, Faith L. Henderson, Tebogo M. Segolodi, Kyle Turner, Dawn K. Smith, Michael C. Thigpen, Lynn A. Paxton

**Affiliations:** 1 Centers for Disease Control and Prevention- Botswana, HIV Prevention Research Unit, Gaborone, Botswana; 2 Centers for Disease Control and Prevention, Division of HIV/AIDS Prevention, Atlanta, Georgia, United States of America; Asociacion Civil Impacta Salud y Educacion, Peru

## Abstract

**Background:**

Tenofovir-emtricitabine (TDF-FTC) pre-exposure prophylaxis (PrEP) has been found to be effective for prevention of HIV infection in several clinical trials. Two studies of TDF PrEP among men who have sex with men showed slight bone mineral density (BMD) loss. We investigated the effect of TDF and the interaction of TDF and hormonal contraception on BMD among HIV-uninfected African men and women.

**Method:**

We evaluated the effects on BMD of using daily oral TDF-FTC compared to placebo among heterosexual men and women aged 18–29 years enrolled in the Botswana TDF2 PrEP study. Participants had BMD measurements at baseline and thereafter at 6-month intervals with dual-energy X-ray absorptiometry (DXA) scans at the hip, spine, and forearm.

**Results:**

A total of 220 participants (108 TDF-FTC, 112 placebo) had baseline DXA BMD measurements at three anatomic sites. Fifteen (6.8%) participants had low baseline BMD (z-score of <−2.0 at any anatomic site), including 3/114 women (2.6%) and 12/106 men (11.3%) (p = 0.02). Low baseline BMD was associated with being underweight (p = 0.02), having high blood urea nitrogen (p = 0.02) or high alkaline phosphatase (p = 0.03), and low creatinine clearance (p = 0.04). BMD losses of >3.0% at any anatomic site at any time after baseline were significantly greater for the TDF-FTC treatment group [34/68 (50.0%) TDF-FTC vs. 26/79 (32.9%) placebo; p = 0.04]. There was a small but significant difference in the mean percent change in BMD from baseline for TDF-FTC versus placebo at all three sites at month 30 [forearm −0.84% (p = 0.01), spine −1.62% (p = 0.0002), hip −1.51% (p = 0.003)].

**Conclusion:**

Use of TDF-FTC was associated with a small but statistically significant decrease in BMD at the forearm, hip and lumbar spine. A high percentage (6.8%) of healthy Batswana young adults had abnormal baseline BMD Further evaluation is needed of the longer-term use of TDF in HIV-uninfected persons.

**Trial Registration:**

ClinicalTrials.gov NCT00448669

## Introduction

Several clinical trials have demonstrated that pre-exposure prophylaxis (PrEP), a strategy in which human immunodeficiency virus (HIV) uninfected persons take antiretroviral medication to reduce their infection risk, is a promising approach for the prevention of HIV transmission [1], [2], [3], [4]. Tenofovir and tenofovir-emtricitabine were chosen for these trials because of their good safety profile [5], long half-life [7], and excellent penetration into the genital compartment [6]. However, use of tenofovir resulted in decreased bone mineral density (BMD) in HIV-infected patients in clinical trials [8] and [9] and cases of fractures and/or osteomalacia due to tenofovir use have been reported [10].

Because of the mounting evidence of the effectiveness of tenofovir in preventing HIV transmission and the increasing likelihood that it will be widely used as PrEP, it is important that we understand its long term effects on the BMD of HIV-uninfected, healthy people. In a randomised clinical trial of PrEP among men who have sex with men in San Francisco, 10% of HIV-negative men had low BMD (z-score <−2.0) at baseline; tenofovir use resulted in a small but statistically significant decline in BMD at the hip and femoral neck [11].

Sub-Saharan Africa with its high HIV burden could benefit from PrEP if it is strategically implemented among high-risk heterosexuals. Because PrEP would be used during sexually active periods, persons might choose to use PrEP for many years. Many would start as adolescents or young adults during their period of skeletal maturation. It is therefore important that we have baseline BMD data for potential users of PrEP, as well as data on any long-term changes in BMD due to prolonged exposure to tenofovir.

There are few data on baseline BMD values in African populations. A study of 20 black African and 20 white European males showed that the bone mineral content (BMC) of the African males as well as their body mass index (BMI) was significantly less than those of the white Europeans. In contrast, neither BMD nor the ratio of BMC to BMI differed between the two groups [12]. In a study done in the United States, race had a highly significant effect on BMD of the lumbar spine, trochanter, femoral neck, and mid-radius. BMD was higher in blacks than in whites. There were no significant interactions between race and age or race and weight when the data from black and white men were combined [13]. In a study of BMD of recent African immigrants in the United States, spinal BMD was significantly lower in recent Sudanese immigrants than in African Americans or Caucasians. Hip and total body BMD were associated with length of stay in the United States, suggesting a potential role of environmental factors in the ethnic differences in BMD [14]

The Centers for Disease Control and Prevention sponsored a PrEP trial evaluating the efficacy and safety of daily TDF-FTC among HIV-uninfected, sexually active young men and women in Botswana [3]. In a subset of study participants, we compared the change in BMD among participants taking oral daily TDF-FTC or placebo, using dual-energy X-ray absorptiometry (DXA) as a previously validated measure of BMD. In addition, we characterized BMD at baseline as well as the risk factors for developing low BMD during the course of the study.

## Methods

The protocol for this trial and supporting CONSORT Checklist are available as supporting information; see Checklist S1 and Protocol S1.

**Figure 1:**
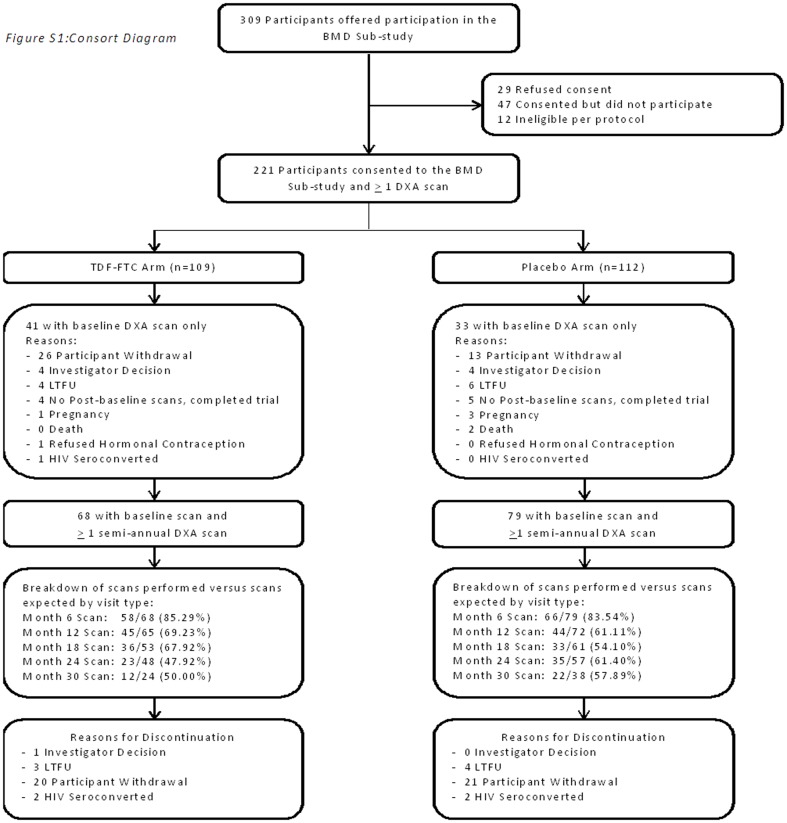
Study design and participant flow diagram.

Between March 2007 and October 2009, 1219 participants were enrolled and randomised in the TDF2 Study conducted at the CDC-Botswana research clinics in Gaborone and Francistown, Botswana. Full details of the conduct and results of the TDF2 Study have been previously published [3]. This was a randomised, double blind, placebo-controlled efficacy and safety trial of TDF-FTC among sexually active HIV-uninfected young Batswana adults. The first 309 TDF2 Gaborone participants were offered enrollment into the BMD sub-study; 221 participants (109 on TDF-FTC, 112 on placebo) consented to the BMD sub-study and had at least one DXA scan performed. The BMD sub-study DXA scans were scheduled at baseline and every subsequent 6 months while participants were enrolled in the TDF2 study. All 221 BMD sub-study participants had a baseline DXA scan but one participant had a DXA scan of the spine only.(participant was 19 years old at the time of his baseline DXA scan and the DXA machine was programmed to process from 20 years and above for the hip and forearm. Therefore a full baseline scan was not done on this participant). Sixty-eight (62.4%) participants on TDF-FTC and 79 (70.5%) on placebo had at least one follow-up DXA scan. Among those also enrolled in the BMD sub-study, forty-two (38.5%) participants on TDF-FTC and 52 (46.4%) participants on placebo completed the TDF2 study. Reasons for exclusion and discontinuation are detailed in the CONSORT diagram (Figure 1). Participants found to have low BMD (T-scores <−1.0) as per World Health Organization (WHO) reference ranges were provided calcium supplementation [15].

In this sub-study, DXA scanning was conducted at Medical Imaging Botswana in Gaborone. All participants were enrolled in Gaborone, and were seen monthly for the assessment of adverse events, rapid HIV testing, and pregnancy testing for female participants. Quarterly visits included laboratory safety monitoring of chemistry results which included, serum creatinine, blood urea nitrogen and inorganic phosphorus. Rapid HIV testing and dispensing of study medication were done monthly.

All participants were informed of the risks and benefits of participation and provided written consent for the TDF2 study and separately for the BMD sub-study. The TDF2 study and BMD sub-study were reviewed and approved by the Institutional Review Board of the Centers for Disease Control and Prevention and the Health Research and Development Committee of the Botswana Ministry of Health (ClinicalTrials.gov Identifier: NCT00448669).

### Bone mineral density assessment

BMD measurements were performed with a QDR 4500C (Hologic Co., Bedford, MA, USA) DXA scan machine by Medical Imaging Botswana at the distal and ultra-distal forearm, the lumbar spine and the hip. The distal forearm was defined as the area between the lines where the radio-ulnar distance measures 24 mm apart proximally and 8 mm apart distally. The ultra-distal forearm was defined as the 10 mm strip of radius distal to the line where the radius and ulna are 8 mm apart. The posterior-anterior L1–L4 lumber spine and the total hip were evaluated. All measurements were done on the same side for each participant for the duration of the study. Serial measurements of BMD at any of the three sites were used to assess changes in bone density. World Health Organization (WHO) reference ranges were used for clinical evaluation of DXA results.

### Additional data collection

For all participants, baseline body weight and height were recorded at enrollment, and body mass index (BMI) was calculated. Additionally, parathyroid hormone and Vitamin D, testosterone (males), calcium and alkaline phosphatase were analyzed at baseline and at any point in the study when a participant developed low BMD defined as a z-score that was <−2.0.

### Statistical Analysis

We conducted baseline and longitudinal analyses of BMD. The baseline analysis explored the relationship between low BMD and participant characteristics and laboratory parameters. The longitudinal analysis assessed the change in BMD over time between the TDF-FTC and placebo treatment groups. All fractures and their anatomic locations were compiled by treatment group and gender.

Gender and treatment group (TDF-FTC, placebo) were considered *a priori* variables that remained in all analyses regardless of statistical significance. Demographic variables included age group (18–24, 25–29 years), education (primary school or less, secondary school, postsecondary school), and marital status (married, single). Medical history variables included alcohol use in the past three months and female hormonal contraception at enrollment (oral, injection or implant, none). Clinical variables included body mass index at baseline, creatinine, creatinine clearance, blood urea nitrogen, inorganic phosphorous, vitamin D, testosterone (males), parathyroid hormone, alkaline phosphatase, albumin-corrected calcium, and mean BMD at baseline (forearm, lumbar spine, hip).

### Baseline analysis

We compiled baseline demographic characteristics and laboratory parameters for all 221 BMD enrolled subjects including those who only had a baseline DXA scan without further follow-up scans. The longitudinal analysis participants, including those who had at least one follow-up scan, were compared to the baseline only participants by characteristic (chi-square test) and laboratory parameter (t-test).

Baseline low BMD using the mean z-scores were compared by treatment group and gender using the 220 participants who had scans at all three anatomical sites. Low BMD was defined as a z-score of more than 2.0 standard deviations below the mean at any anatomic site forearm, lumbar spine, hip as per International Society for Clinical Densitometry (ISCD), 2007 Position Statement on BMD reporting in men younger than age 50 [16] One male TDF-FTC participant only had a z-score for spine (−1.8) and was excluded from the low BMD correlates analysis, leaving 220 participants. We calculated the prevalence and 95% confidence intervals (CI) and compared the observed number of low BMD cases to the expected number using an exact binomial test. We expected 2.3% (five) of our 220 DXA participants to have a BMD z-score below −2.0. Prevalence ratios (PR) were estimated using a Poisson model with robust standard error. For categorical characteristics, the PR was calculated using one category as the reference. Since laboratory parameters are continuous, the PR was based on a one-unit change.

### Longitudinal analysis

The longitudinal analysis used all subjects who had at least one post baseline follow-up DXA scan. We computed the percent of participants who had >3% BMD decrease at any time compared to their baseline measurement and compared TDF-FTC to placebo and females to males using a two-sided Fisher's exact test. A >3% loss represents more than expected BMD loss in a population of healthy men in which BMD should be stable [11] We used a linear mixed model with participants as a random effect to estimate the BMD percent change over time by anatomic site (forearm, lumbar spine, hip) and included the following fixed effect factors: treatment group (TDF-FTC, placebo), time at 6-month intervals, and interaction between the treatment group and 6-month interval. The linear mixed effects model analysis was repeated adjusting for gender and other characteristics and laboratory parameters in Table 1 that had a p-value<0.10. An additional BMD percent change analysis used only females and included the contraceptive data (injectable, oral or no contraceptive) in the linear mixed-effects model. The female only base model contained the following predictor variables: treatment group, time, and interactions for contraceptives by time and by treatment group. These models were used to estimate the mean percent change in BMD from month 0 to 30. All analyses were performed using SAS software version 9.3 (SAS Institute, Inc., Cary, NC) and p-values<0.05 were considered to indicate statistical significance.

**Table 1:** Baseline characteristics of TDF2 DXA sub study participants who had at least one DXA bone mineral density scan at forearm, spine and hip: Botswana, 2007–2010.

Characteristic	Baseline Only^a^ N = 74 (%)	Longitudinal Cohort^b^ N = 147 (%)	P-value
**Age group**			0.88
18–24 years	42 (56.8)	85 (57.8)	
24–29 years	32 (43.2)	62 (42.2)	
**Gender**			0.42
Female	41 (55.4)	73 (49.7)	
Male	33 (44.6)	74 (50.3)	
**Educational level**			0.21
Primary or less	0 (0.0)	6 (4.1)	
Secondary	51 (68.9)	99 (67.3)	
Postsecondary	23 (31.1)	42 (28.6)	
**Marital status**			0.72
Married	1 (1.4)	3 (2.0)	
Single	73 (98.6)	144 (98.0)	
**Reported any alcohol use in the last 3 months**	49 (66.2)	90 (61.2)	0.47
**Weight Assessment (Based on BMI)**			0.75
Underweight	14 (18.9)	26 (17.7)	
Normal, Overweight, or Obese	58 (78.4)	121 (82.3)	
Missing	2 (2.7)	0 (0.0)	

Characteristic	Mean (95% CI)	Mean (95% CI)	P-value
**Mean BMD at baseline (g/cm2)**			
Forearm	0.57 (0.56, 0.58)	0.58 (0.57, 0.59)	0.20
Lumbar spine	0.98 (0.95, 1.01)	1.00 (0.98, 1.02)	0.24
Hip	1.05 (1.01, 1.09)	1.05 (1.03, 1.07)	0.86
**Laboratory parameters (mean)**			
Creatinine (mg/dl)	0.72 (0.68, 0.75)	0.74 (0.72, 0.77)	0.29
Creatinine clearance (ml/min)	130.36 (122.67, 138.04)	125.28 (119.80, 130.75)	0.29
Blood urea/nitrogen (mg/dl)	11.69 (10.44, 12.94)	10.80 (10.05, 11.55)	0.20
Inorganic phosphorous (mg/dl)	3.21 (3.09, 3.33)	3.28 (3.20, 3.36)	0.34
Vitamin D (ng/ml)	28.53 (26.92, 30.13)	30.23 (28.77, 31.69)	0.12
Testosterone (ng/dl) - males	650.27 (568.12, 732.41)	607.62 (567.81. 647.42)	0.29
Parathyroid hormone (pg/ml)	32.97 (30.75, 35.18)	34.33 (31.77, 36.88)	0.42
Alkaline phosphatase (IU/L)	71.40 (66.02, 76.78)	69.28 (65.99, 72.57)	0.49
Corrected Calcium (mg/dl)	9.32 (9.26, 9.38)	9.35 (9.31, 9.39)	0.36
**Female contraception at enrollment**	**(n = 41)**	**(n = 73)**	0.24
No Hormonal Method	0 (0.0)	2 (2.7)	
Oral contraceptive	19 (46.3)	42 (57.5)	
Injection or implant	22 (53.7)	29 (39.7)	

a These are the 74 participants that only had a baseline DXA scan.

b These are the 147 participants that had at least one follow-up DXA scan (Longitudinal cohort).

## Results

### Baseline Analysis

A total of 74/221 (33.5%) BMD sub-study participants had only a baseline DXA scan. Of these, 41 were female and 33 were male and 18.9% were underweight as defined by BMI. Their baseline characteristics and laboratory parameters are presented in Table 1. There were no statistical differences between the baseline-only and longitudinal cohorts in demographic or clinical characteristics.

Fifteen of the 220 participants who had scans at all three sites had a low baseline BMD as defined as at least one anatomic site z-score <−2.0(On TDF: 2 had low BMD at spine and 5 at the arm; On placebo: 3 had low BMD at spine and arm, 3 at spine only and 2 at arm only) (prevalence 6.8%, 95% CI 3.4–11.0), versus the five (2.3%) expected (p<0.001). Of the 106 male participants, 12 (11.3%) had a low baseline BMD and for the 114 female participants, three (2.6%) had a low baseline BMD (male/female PR = 4.3, p = 0.02). There was a significantly greater prevalence of low baseline BMD for participants classified as underweight compared to normal/overweight/obese (PR = 3.06, p = 0.02). Low baseline BMD was significantly associated with increasing blood urea nitrogen (p = 0.02) and alkaline phosphatase (p = 0.03), and decreasing creatinine clearance (p = 0.04) (Table 2). In addition to the participants with low BMD (z<−2.0), 51.4% of the 220 participants (5 women and 64 men) had a T score at one or more anatomic sites of <−1.0 at baseline, corresponding to clinical osteopenia (Note that these criteria may not be appropriate in young men and women) (Table S1 in File S1).

**Table 2:** The univariate prevalence ratios (PR) for low bone mineral density at baseline for at least one DXA scan site (forearm, spine, hip) estimated by z-scores less than −2.0, with 95% confidence intervals (CI) and p-values, by demographic characteristics, risk factors and laboratory parameters (N = 220): Botswana, 2007–2010.

Characteristic	Prevalence	95% CI for prevalence	PR	95% CI for PR	P-value
**Treatment Allocation**					
Placebo	0.0714	(0.0366, 0.1393)	1.10	(0.41, 2.93)	0.85
TDF/FTC	0.0648	(0.0317, 0.1327)	REF		
**Age group**					
18–24 years	0.0794	(0.0438, 0.1438)	REF		
25–29 years	0.0532	(0.0227, 0.1248)	0.67	(0.24, 1.90)	0.45
**Gender**					
Female	0.0263	(0.0086, 0.0804)	REF		
Male	0.1132	(0.0664, 0.1929)	4.30	(1.25, 14.82)	0.02
**Educational level**					
Secondary or less	0.0645	(0.0354, 0.1175)	REF		
Postsecondary	0.0769	(0.0331, 0.1786)	1.19	(0.42, 3.35)	0.74
**Marital status** a					
Single	0.0694	(0.0426, 0.1131)			
**Reported any alcohol use in the last 3 months**					
Yes	0.0725	(0.0399, 0.1316)	REF		
No	0.0610	(0.0261, 0.1426)	0.84	(0.30, 2.38)	0.74
**Female hormonal contraception at enrollment** b					
Oral contraceptive	0.0164	(0.0023, 0.1145)	REF		
Injection or implant	0.0392	(0.0101, 0.1526)	2.39	(0.22, 25.63)	0.47
**BMI**					
Underweight	0.1538	(0.0737, 0.3212)	3.06	(1.16, 8.10)	0.02
Normal, overweight, or obese	0.0503	(0.0266, 0.0950)	REF		
**Serum creatinine** c			4.42	(0.28, 69.26)	0.29
**Creatinine clearance**			0.98	(0.97, 1.00)	0.04
**Blood urea nitrogen**			1.07	(1.01, 1.13)	0.02
**Serum inorganic phosphorus**			0.57	(0.25, 1.29)	0.18
**Vitamin D**			1.00	(0.94, 1.06)	0.99
**Corrected calcium**			0.61	(0.10, 3.88)	0.60
**Alkaline phosphatase**			1.01	(1.00, 1.03)	0.03
**Total testosterone - males**			1.00	(1.00, 1.00)	0.66
**Parathyroid hormone**			1.00	(0.94, 1.06)	0.88

a There are 4 subjects who were married, and none had a low BMD.

b There were 2 with no hormonal method, and neither had low BMD.

c Prevalence ratios for laboratory values refer to a one unit change in that parameter.

### Longitudinal Analysis

A total of 147 BMD sub-study participants had two or more scans available for the longitudinal analysis. There were no statistically significant differences for baseline characteristics or laboratory parameters between treatment groups (Table S2 in File S1). The 68 TDF-FTC longitudinal participants had a mean of 2.05 years in the study (median = 2.13 years, minimum = 0.65 years, maximum = 3.3 years), for a total of 139.3 observation-years. The 79 placebo participants had a mean of 2.1 years (median = 2.16 years, minimum = 0.54 year, maximum = 3.65 years), for a total of 165.7 observation-years.

Half of the participants on TDF-FTC (34/68, 50.0%) and a third of participants on placebo (26/79, 32.9%) (p = 0.04) had more than a 3.0% decrease in BMD at any site (arm, spine or hip) at any time point after baseline. There were no significant differences by gender for a 3% decrease, neither within the entire group of participants, nor between treatment groups: 37.0% of females (29/73) and 41.9% of males (31/74) had a 3% decrease (p = 0.87)

**Figure 2:**
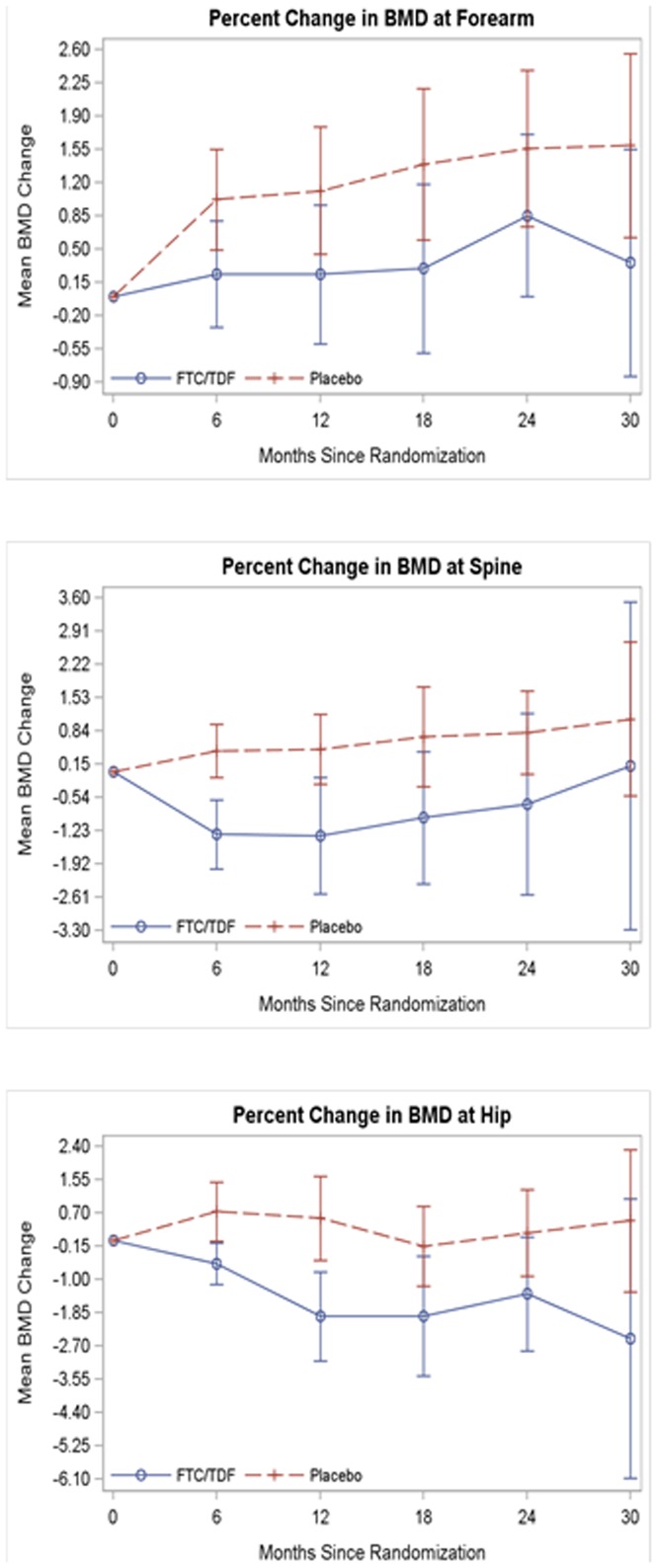
Mean percent bone mineral density (BMD) change (with 95% confidence intervals) from baseline to subsequent months by treatment group (TDF-FTC versus placebo).

A linear mixed-effects model for the percent change in BMD between baseline and each subsequent 6-month interval was fit to forearm, spine and hip separately. These base models included time, treatment group, the interaction between time and treatment group, and a participant random intercept and slope. The mean percent change from baseline and 95% CI by treatment group at weeks 6, 12, 18, 24, and 30 are presented in Figure 2. There was no significant difference in the rate of change for the percent change in BMD from baseline i.e., slope, between the TDF-FTC and placebo groups for any of the anatomic sites. Hence, a common slope parameter was estimated for each of the anatomic site models. The common TDF-FTC and placebo slopes for mean percent change in BMD for forearm (+0.37% per year; p = 0.01) and spine (+0.62% per year; p = 0.007) were significant and positive, which indicates that over time the mean BMD for both treatment groups increased. Though the BMD loss appears to stabilize over time, it never gets better relative to the placebo arm. This difference appears to be noticeable by 6 months and then doesn't get worse. There was a significant net difference in the mean percent change from baseline between the groups: the mean percent change in BMD at month 30 for the forearm was +0.97% for the TDF-FTC group versus +1.83% for the placebo group. For the spine it was −0.09% and +1.55% at month 30, respectively. These constant between-group percentage point differences of −0.86 (p = 0.008) for forearm and −1.64 (p = 0.0002) for the spine were significant (Table 3). The slope for mean percent BMD change over time was not significant for the hip, which means the net difference from baseline and between treatment groups was constant over time. The mean BMD change at month 30 for the hip was −1.03% (TDF-FTC) versus +0.52% (placebo). This results in a significant 1.55 percentage point difference from baseline in the hip mean BMD for the TDF-FTC group compared to placebo (p = 0.001).

**Table 3:** Univariate and multivariable model results for the net difference between TDF/FTC and placebo in mean percent change in bone mineral density for months 6–30 from the baseline: Botswana, 2007–2010.

Model	Comparison	Mean Percent Change in BMD	95% CI	P-value
**Univariate**				
**Hip** a	TDF/FTC vs. placebo	−1.55%	(−2.48, −0.62)	0.001
**Spine**	Slope^b^	+0.62	(+0.17, +1.07)	0.007
	TDF/FTC vs. placebo	−1.64%	(−2.48, −0.80)	0.0002
**Forearm**	Slope^b^	+0.37	(+0.08, +0.67)	0.01
	TDF/FTC vs. placebo	−0.86%	(−1.49, −0.23)	0.008
**Multivariable**				
**Hip** a	TDF/FTC vs. placebo	−1.51%	(−2.49, −0.54)	0.003
**Spine**	Slope^b^	+0.43	(−0.04, +0.90)	0.07
	TDF/FTC vs. placebo	−1.62%	(−2.46, −0.78)	0.0002
**Forearm**	Slope^b^	+0.42	(+0.11, +0.72)	0.008
	TDF/FTC vs. placebo	−0.84%	(−1.52, −0.17)	0.01
	Underweight vs. Others^c^	+1.03	(+0.29, +1.77)	0.007

a Slope was not significant in models for Hip.

b Slope is for a one unit (1 year) change.

c BMI underweight vs. others (normal, overweight, obese).

A multivariable model was fit including gender and baseline characteristics and laboratory parameters from Tables 1, 2 or Table S2 in File S1 with p-values<0.10: BMI, BUN, creatinine, and creatinine clearance. Although alkaline phosphatase was significant, it was not included in the model because it was only measured at baseline. Four BMD sub-study subjects seroconverted to HIV-positive during the TDF2 study. However, all four subjects seroconverted after their last DXA measurement. Hence, there was no need to censor these subjects during the analysis. In the multivariable model, as for the base models, there was a significant decrease in mean percent BMD change from baseline for the TDF-FTC versus placebo treatment groups of −1.62% for spine (p = 0.0002), −1.51% for hip (p = 0.003), and −0.84% for forearm (p = 0.01) (Table 3). In addition, for the forearm multivariable model BMI was significant and underweight subjects had a significant +1.03 percentage point difference from baseline compared to the BMI others (others includes normal, overweight, and obese) (p = 0.007).

Of the 220 participants with baseline scans at all three sites, there were 106 observations for which they had been placed on a calcium supplement for low BMD at some point during the 6 months prior to a DXA scan, but continued on study drug. We did a sensitivity analysis and re-ran our multivariable models by 1) controlling for those on a supplement and 2) removing time points at which a participant was on a calcium supplement. The sensitivity analysis results for the difference in mean percent change in BMD for TDF-FTC compared to placebo when controlling for calcium in the model were −1.69% for spine (p = 0.0001), −1.49% for hip (p = 0.003), and −0.82% for forearm (p = 0.02). These results are almost identical to the full model and the calcium laboratory parameter was not significant in any model. The sensitivity analysis results for the difference in mean percent change in BMD for TDF-FTC compared to placebo when removing the 106 observations with calcium supplements are −1.51% for spine (p = 0.002), −1.54% for hip (p = 0.008), and −0.58% for forearm (p = 0.11). The only significant change in the results from removing observations with a calcium supplement was that the difference by treatment group for forearm became non-significant.

Two participants from the DXA sub-study sustained traumatic fractures during the course of the study, one from each treatment group (Table S3 in File S1).

### Percent change in BMD—female contraceptive model results

There were nine contraceptive observations classified as both oral and injection. We conducted a sensitivity analysis by placing the category “both” with “injection” and then with “oral” and results indicated no substantial differences from the model with contraceptive in the original four categories (injection, oral, both and none). In the original model, there was no significant mean percent change of BMD for hip or forearm, by either injection or oral contraceptives versus none. For spine, the only contrast that was significant was a positive interaction between treatment group and oral contraceptives versus no contraceptive (p<0.0004) (Table S4 in File S1).

## Discussion

Results from the percent change in BMD models reveal significant differences at forearm, spine or hip for both the univariate and multivariable models. The TDF-FTC treatment group consistently had a greater decrease in BMD from baseline for all significant differences than the placebo group. The multivariable models revealed that the only additional risk factor that was significant was BMI and only in the forearm model (underweight compared to normal/overweight/obese classes combined) Our findings for the hip are consistent with those of a PrEP study conducted among HIV-negative men in San Francisco, which found that TDF use resulted in a small but statistically significant decline in BMD at the hip [11]. The BMD loss in this study was greater compared with those observed in the CDC MSM and iPrEx studies. This probably reflects a different study population.

A high percentage of healthy young Batswana adults in this study had baseline z-scores that were below the expected range for age (6.8%) and young men had a higher prevalence of low BMD than did women (11.3% versus 2.6%, p = 0.02). These findings are substantially greater than what has been previously reported for young persons in populations in Europe and the US [17]. It is not clear from our data whether the greater prevalence of abnormal z-scores in this otherwise healthy young population is due to true biological abnormality or whether it simply implies that the reference ranges, which were primarily drawn from European and North American cohorts, are not applicable to an African population.

Low baseline BMD was significantly associated with being underweight, high blood urea nitrogen, and low creatinine clearance, implying a relationship with nutritional or renal status. Low baseline BMD was also associated with high alkaline phosphatase, which may reflect bone mineral breakdown. Lab values of Vitamin D, calcium, parathyroid hormone, inorganic phosphorus, and testosterone (in males) were not associated with low baseline BMD. The proportion with >3% BMD loss at anatomic site during follow up was greater for the TDF-FTC group than the placebo group. BMD losses at any anatomic site at any point after baseline were significantly greater for the TDF-FTC treatment group than the placebo group for 3% decrease, but there were no significant differences of BMD loss by gender. It is noteworthy that there was BMD loss over the course of the study even among participants in the placebo arm.(BMD loss at arm only 2, spine and arm 3 and spine only 3) While this is consistent with some recent studies that indicate that BMD loss, particularly at the hip, begins shortly after achieving peak bone maturation in early adulthood and accelerates with age [18], [19], [20] it still raises the largely unanswered question of the extent to which BMD normally fluctuates among healthy young adults and whether the population in this study differs from populations in other regions.

The two participants who sustained traumatic fractures had low baseline Z-scores (TDF/FTC participant: forearm −1.7; spine −1.8; hip −0.3. Placebo participant: forearm −1.8; spine −2.3, hip −1.6). While an important risk factor for fractures, most individuals who experience a fragility fracture do not have a BMD in the lowest range [21]. Despite TDF-FTC based therapies having a greater effect on reducing BMD in antiretroviral-naïve HIV-infected patients, the incidence of fractures did not differ significantly between antiretroviral therapy regimens [22].

In our study, the mean percent changes in BMD for women on either oral or injectable hormonal contraception vs. no contraception were not significant except for a positive effect at the spine of oral contraceptives for women on TDF-FTC. (Table S4 in File S1). The balance of evidence leans towards a positive effect of oral contraceptives on BMD in women of all ages while loss of BMD was associated with use of depo-medroxyprogesterone acetate (DMPA) [23]. Loss of BMD among DMPA users has been reported without showing significant BMD changes among oral contraceptive users, both compared to controls [24].

For participants with a low BMD who were placed on a calcium supplement, there was no significant difference from the original model after controlling for supplementation. In a meta-analysis of randomised placebo controlled trials of calcium supplementation in healthy children, there was no effect of calcium supplementation on BMD at the femoral neck or at the lumbar spine at the end of the trials [25]. The effect on the upper limb was small, equivalent to about a 1.7% greater increase in BMD in the supplemented groups compared with the control groups. The authors pointed out that, in calcium supplement trials in postmenopausal women, the effect of supplementation on risk of fracture is at best small, with increases in BMD ranging from 1.13% to 2.05% depending on site of BMD measurement. Other clinical trials, however, have found a positive effect of calcium supplementation on the BMD of children and adolescents [26], [27].

Our study strength is that it is the first to look at the baseline BMD and TDF-FTC associated BMD loss in a healthy African population of young men and women. However, our study has several limitations such as relatively small sample size and no reference ranges for an African population. The trial was done at one urban location and we did not collect information about other relevant risk factors such as smoking nor did we have any information about dietary practices or precise measures of alcohol use. Only 42 participants in the TDF-FTC group and 52 participants in the placebo group completed the study, primarily due to participant relocation.

In conclusion, we found that TDF-FTC use among HIV-negative young African heterosexuals resulted in a minimal but statistically significant decline in BMD. As plans for PrEP implementation proceed, there is a need to further evaluate long-term use of TDF-FTC in HIV uninfected persons.

## Supporting Information

File S1 **Supporting Tables.** Table S1. The number and percent of participants having a T score less than −1.0 at baseline. Table S2. Baseline characteristics among TDF2 DXA longitudinal study participants who had at least one six-month follow-up DXA scan: Botswana, 2007–2010. Table S3. The fractures occurring among DXA (n = 221) participants by treatment group and gender. Table S4. The results for the female contraceptive BMD percent change over time analysis.
(DOCX)

Protocol S1 **Trial Protocol.**
(DOC)

Checklist S1 **CONSORT Checklist.**
(DOC)

## References

[1] Grant RM, Lama JR, Anderson PL, McMahan V, Liu AY, et al (2010) Pre-exposure chemoprophylaxis for HIV prevention in men who have sex with men. N Engl J Med 363: 2587–2599. 21091279 10.1056/NEJMoa1011205 PMC3079639

[2] Baeten JM, Donnell D, Ndase P, Mugo NR, Campbell JD, et al (2012) Antiretroviral prophylaxis for HIV prevention in heterosexual men and women. N Engl J Med 367 (5) 399–410. 22784037 10.1056/NEJMoa1108524 PMC3770474

[3] Thigpen MC, Kebaabetswe PM, Paxton LA, Smith DK, Rose CE, et al (2012) Antiretroviral pre-exposure prophylaxis for heterosexual HIV transmission in Botswana. N Engl J Med 367 (5) 423–344. 22784038 10.1056/NEJMoa1110711

[4] Choopanya K, Martin M, Sangkum U, Mock PA, Leethochawalit M, et al. (2013) Antiretroviral prophylaxis for HIV infection in injecting drug users in Bangkok, Thailand (the Bangkok Tenofovir Study): a randomised, double-blind, placebo-controlled phase 3 trial 10.1016/S0140-6736(13)61127-7 23769234

[5] Gallant JE, DeJesus E, Arribas JR, Pozniak AL, Gazzard B, et al (2006) Tenofovir-DF, emtricitabine, and efavirenz vs. zidovudine, lamivudine and efavirenz for HIV. N Engl J Med 354: 251–260. 16421366 10.1056/NEJMoa051871

[6] Dumond JB, Yeh RF, Patterson KB, Corbet AH, Jung BH, et al (2007) Antiretroviral drug exposure in the female genital tract: implications for oral pre-and post-exposure prophylaxis. AIDS 21: 1899–1907. 17721097 10.1097/QAD.0b013e328270385a PMC2862268

[7] Hawkins T, Veiled W, St Claire RL, Guyer B, Clark N, et al (2005) Intracellular pharmacokinetics of tenofovir diphosphate, carbovir triphosphate and lamivudine triphosphate in patients receiving triple-nucleoside regimens. JAIDS 39: 406–411. 16010161 10.1097/01.qai.0000167155.44980.e8

[8] Gallant JE, Staszewski S, Pozniak AL, Gazzard B, Suleiman JM, et al (2004) Efficacy and safety of tenofovir-DF vs. stavudine in combination therapy in antiretroviral-naive patients: a 3-year randomized trial. JAMA 292: 191–201. 15249568 10.1001/jama.292.2.191

[9] Martin A, Block M, Amin J, Baker D, Cooper DA, et al (2009) Simplification of antiretroviral therapy with tenofovir-emtricitabine or abacavir-lamivudine: a randomized, 96-week trial. Clin Infect Dis 49: 1591–1601. 19842973 10.1086/644769

[10] Perrot S, Aslangul E, Szwebel T, Caillat-Vigneron N, Le Jeunne C (2009) Bone pain due to fractures revealing osteomalacia related to tenofovir- induced proximal renal tubular dysfunction in a human immunodeficiency virus-infected patient. J Clin Infect Dis 51: 963–972. 10.1097/RHU.0b013e31819c20d8 19265350

[11] Liu AY, Vittinghoff E, Sellmeyer DE, Irvin R, Mulligan K, et al (2011) Bone mineral density in HIV-negative men participating in a tenofovir pre-exposure prophylaxis randomized clinical trial in San Francisco. PLoS ONE 6 (8) e23688. 21897852 10.1371/journal.pone.0023688 PMC3163584

[12] Bileckot R, Audran M, Masson C, Ntsiba H, Simon P, et al (1991) Bone density in 20 black African young adults of the Bantu race is identical to that in subjects of white race. Rev Mal Osteoartic 58 (11) 787–789. 1780654

[13] Bell NH, Gordon L, Stevens J, Shary JR (1995) Demonstration that bone mineral density of the lumbar spine, trochanter, and femoral neck is higher in black than in white young men. Calcif Tissue Int 56 (1) 11–13. 7796339 10.1007/BF00298737

[14] Gong G, Haynatzki G, Haynatzka V, Kosoko-Lasaki S, Howell R, et al (2006) Bone mineral density of recent African immigrants in the United States. JNMA 98 (5) 746–752. PMC2569286 16749650

[15] World Health Organization (1994) Assessment of fracture risk and its application to screening for postmenopausal osteoporosis. Available at: http://whqlibdoc.who.int/trs/WHO-TRS-843.pdf. Accessed on December, 02, 2011. 7941614

[16] International Society for Clinical Densitometry (ISCD) (2007) Position Statement on BMD reporting in men younger than age 50. Available at www.iscd.org/official-positions. Accessed: 20 Nov 2013.

[17] Ferrari S, Bianchi ML, Eisman JA, Foldes AJ, Adami S, et al (2012) Osteoporosis in young adults: pathophysiology, diagnosis, and management. Osteoporos Int [Epub ahead of print]. 10.1007/s00198-012-2030-x 22684497

[18] Nordstrom P, Neovius M, Nordstrom A (2007) Early and rapid bone mineral density loss of the proximal femur in men. J Clin Endocrinol Metab 92: 1902–1908. 17311855 10.1210/jc.2006-2613

[19] Lee EY, Kim D, Kim JM, Kim KJ, Choi HS, et al (2012) Age-related bone mineral densit patterns in Koreans KNS IV). J Clin Endocrinol Metab 97: 3310–3318. 22701016 10.1210/jc.2012-1488

[20] Riggs BL, Melton LJ, Robb RA, Camp JJ, Atkinson EJ, et al (2008) A population-based assessment of rates of bone loss at multiple skeletal sites: evidence for substantial trabecular bone loss in young adult women and men. J Bone Miner Res 23: 205–214. 17937534 10.1359/JBMR.071020 PMC2665699

[21] Womack JA, Goulet JL, Gibert C, Brandt C, Chang CC, et al (2011) Increased risk of fragility fractures among HIV infected compared to uninfected male veterans. PLoS ONE 6 (2) e17217. 21359191 10.1371/journal.pone.0017217 PMC3040233

[22] McComsey GA, Kitch D, Daar ES, Tierney C, Jahed NC, et al (2011) Bone mineral density and fractures in antiretroviral-naive persons randomized to receive abacavir-lamivudine or tenofovir disoproxil fumarate-emtricitabine along with efavirenz or atazanavir-ritonavir. J Infect Dis 203: 1791–1801. 21606537 10.1093/infdis/jir188 PMC3100514

[23] Berenson AB, Breitkopf CR, Glady JJ, Rickert VI, Thomas A (2004) Effects of hormonal contraceptives on bone mineral density after 24 months. Obstet Gynecol 103 (5pt1) 899–906. 15121563 10.1097/01.AOG.0000117082.49490.d5

[24] Cromer BA (1999) Effects of hormonal contraceptives on bone mineral density. Drug Saf 20 (3) 213–222. 10221851 10.2165/00002018-199920030-00002

[25] Winzenberg T, Shaw K, Fryer J, Jones G (2006) Effects of calcium supplementation on bone density in healthy children: meta-analysis of randomised controlled trials. BMJ doi:10.1136/bmj.38950.561400.55 10.1136/bmj.38950.561400.55 PMC1602024 16980314

[26] Lloyd T, Andon MB, Rollings N, Martel JK, Landis JR, et al (1993) Calcium supplementation and bone mineral density in adolescent girls. JAMA 270 (7) 841–844. 8340983

[27] Johnson CC, Miller JZ, Slemenda CW, Reister TK, Hui S, et al (1992) Calcium supplementation and increases in bone mineral density in children. N Engl J Med 327: 82–87. 1603140 10.1056/NEJM199207093270204

