# Supplementary material for: Bone Mineral Density Changes among HIV-Uninfected Young Adults in a Randomised Trial of Pre-Exposure Prophylaxis with Tenofovir-Emtricitabine or Placebo in Botswana
Source: PLoS One. 2014 Mar 13;9(3):e90111. doi: 10.1371/journal.pone.0090111 (PMC3953113; doi:10.1371/journal.pone.0090111)
Supplement: File S1 — Supporting Tables. Table S1. The number and percent of participants having a T score less than −1.0 at baseline. Table S2. Baseline characteristics among TDF2 DXA longitudinal study participants who had at least one six-month follow-up DXA scan: Botswana, 2007–2010. Table S3. The fractures occurring among DXA (n = 221) participants by treatment group and gender. Table S4. The results for the female contraceptive BMD percent change over time analysis. (DOCX) [file pone.0090111.s001.docx]

Supporting information files

**Botswana TDF2: The BMD Manuscript Appendices and Supplemental Tables**

Table S1. The number and percent of participants having a T score less than -1.0 at baseline.

|  | **T-Score < -1.0 (n)** | **Participants (total)** | **Percent** | **P-value** |
| --- | --- | --- | --- | --- |
| **Overall^a^** | 113 | 220 | 51.4 | <0.0001 |
|  |  |  |  |  |
| **By Treatment** |  |  |  |  |
| Placebo | 58 | 112 | 51.8 | 0.69 |
| TDF | 59 | 108 | 54.6 |  |
|  |  |  |  |  |
| **By Gender** |  |  |  |  |
| Female | 53 | 114 | 46.5 | 0.043 |
| Male | 64 | 106 | 60.4 |  |

^a^P-value for the overall t-score test is an exact binomial test of the percent observed versus the percent expected. Percent expected for t-score is 0.1592.

**Table S2**. Baseline characteristics among TDF2 DXA longitudinal study participants who had at least one six-month follow-up DXA scan: Botswana, 2007-2010.

| **Characteristic** | **TDF-FTC**  **n=68 (%)** | **Placebo**  **n=79 (%)** | **P-value** |
| --- | --- | --- | --- |
| **Age group** |  |  | 0.66 |
| 18-24 years | 38 (55.9) | 47 (59.5) |  |
| 24-29 years | 30 (44.1) | 32 (40.5) |  |
| **Gender** |  |  | 0.21 |
| Female | 30 (44.1) | 43 (54.4) |  |
| Male | 38 (55.9) | 36 (45.6) |  |
| **Educational level** |  |  | 0.87 |
| Primary or less | 3 (4.4) | 3 (3.8) |  |
| Secondary | 47 (69.1) | 52 (65.8) |  |
| Postsecondary | 18 (26.5) | 24 (30.4) |  |
| **Marital status** |  |  | 0.65 |
| Married | 1 (1.5) | 2 (2.5) |  |
| Single | 67 (98.5) | 77 (97.5) |  |
| **Reported any alcohol use in the last 3 months** | 43 (63.2) | 47 (59.5) | 0.64 |
| **Weight Assessment (Based on BMI)** |  |  | 0.38 |
| Underweight | 10 (14.7) | 16 (20.3) |  |
| Normal, Overweight, or  Obese | 58 (85.3) | 63 (79.7) |  |
| **Characteristic** | **Mean (95% CI)** | **Mean (95% CI)** | **P-value** |
| **Mean BMD at baseline (g/cm2)** |  |  |  |
| Forearm | 0.58 (0.57, 0.59) | 0.58 (0.57, 0.60) | 0.76 |
| Lumbar spine | 0.99 (0.96, 1.01) | 1.01 (0.99, 1.03) | 0.15 |
| Hip | 1.04 (1.01, 1.07) | 1.06 (1.02, 1.09) | 0.50 |
| **Laboratory parameters (mean)** |  |  |  |
| Creatinine (mg/dl) | 0.77 (0.72, 0.81) | 0.72 (0.69, 0.75) | 0.09 |
| Creatinine clearance (ml/min) | 119.87 (113.64, 126.10) | 129.94 (121.27, 138.60) | 0.06 |
| Blood urea/nitrogen (mg/dl) | 10.56 (9.64, 11.48) | 11.01 (9.85, 12.18) | 0.54 |
| Inorganic phosphorous (mg/dl) | 3.28 (3.16, 3.40) | 3.28 (3.17, 3.39) | 0.99 |
| Vitamin D (ng/ml) | 30.54 (28.09, 32.99) | 29.96 (28.20, 31.72) | 0.70 |
| Testosterone (ng/dl) - males | 588.91 (535.50, 642.32) | 627.37 (566.03, 688.71) | 0.34 |
| Parathyroid hormone (pg/ml) | 33.36 (29.13, 37.59) | 35.08 (31.78, 38.37) | 0.51 |
| Alkaline phosphatase (IU/L) | 69.32 (65.10, 73.54) | 69.24 (64.22, 74.26) | 0.98 |
| Corrected Calcium (mg/dl) | 9.37 (9.32, 9.42) | 9.33 (9.28, 9.39) | 0.30 |
| **Female contraception at enrollment** | **(n=30)** | **(n=43)** |  |
| No Hormonal Method | 0 (0) | 2 (4.7) | 0.34 |
| Oral contraceptive | 16 (53.3) | 26 (60.5) |  |
| Injection or implant | 14 (46.7) | 15 (34.9) |  |

**Table S3**. The fractures occurring among DXA (n = 221) participants by treatment group and gender.

| **Cause** | **TDF-FTC** | | **Placebo** | |
| --- | --- | --- | --- | --- |
|  | **Males** | **Female** | **Males** | **Females** |
| **All Participants** | 5 | 2 | 4 | 2 |
| Trauma | 2^a^ | 1^b^ | 1^c^ | 0 |
| Road traffic accident | 3 | 1 | 0 | 2 |
| Sports | 0 | 0 | 3 | 0 |
| Birth Injury | 0 | 0 | 0 | 0 |
| Others | 0 | 0 | 0 | 0 |
|  |  |  |  |  |
| **DXA Participants** | 0 | 1 | 1 | 0 |
| Trauma | 0 | 1^b^ | 1^c^ | 0 |
| Road traffic accident | 0 | 0 | 0 | 0 |
| Sports | 0 | 0 | 0 | 0 |
| Birth Injury | 0 | 0 | 0 | 0 |
| Others | 0 | 0 | 0 | 0 |
| Baby Fractured Clavicle | 0 | 0 | 0 | 1^d^ |

^a^One participant was involved in an occupational accident with heavy machinery. The other participant was involved in an accident and sustained a traumatic fracture to left distal the radius.

^b^Participant fractured her left distal humerus after a fall.

^c^Participant fractured rib after assault.

^d^Sex of baby unknown.

**Table S4.** The results for the female contraceptive BMD percent change over time analysis. The difference is defined as the net difference in mean percent change over time between groups over time (negative values indicate a decrease). Model I uses all four contraceptive categories. Model II and III place the contraceptive category “both” into injection and oral, respectively.

|  | **Model 1** | | **Model II** | | **Model III** | |
| --- | --- | --- | --- | --- | --- | --- |
| **Comparison** | **Difference** | **P-value** | **Difference** | **P-value** | **Difference** | **P-value** |
| **Hip** |  |  |  |  |  |  |
| Injection vs. None | -1.43 | 0.16 | -1.36 | 0.16 | -1.45 | 0.15 |
| Oral vs. None | -0.13 | 0.89 | -0.13 | 0.89 | -0.40 | 0.67 |
|  |  |  |  |  |  |  |
| **Forearm** |  |  |  |  |  |  |
| Injection vs. None | -0.68 | 0.399 | -0.85 | 0.27 | -0.72 | 0.38 |
| Oral vs. None | -0.58 | 0.45 | -0.60 | 0.44 | -0.74 | 0.33 |
|  |  |  |  |  |  |  |
| **Spine** |  |  |  |  |  |  |
| **Placebo** |  |  |  |  |  |  |
| Injection vs. None | -1.19 | 0.30 | -1.26 | 0.23 | -1.16 | 0.33 |
| Oral vs. None | +1.05 | 0.30 | +1.04 | 0.30 | +0.35 | 0.73 |
|  |  |  |  |  |  |  |
| **TDF-FTC** |  |  |  |  |  |  |
| Injection vs. None | +1.49 | 0.32 | +1.51 | 0.31 | +1.49 | 0.34 |
| Oral vs. None | +5.86 | 0.0004 | +5.84 | 0.0004 | +5.63 | 0.0009 |
