# Supplementary material for: Bone Mineral Density Changes among HIV-Uninfected Young Adults in a Randomised Trial of Pre-Exposure Prophylaxis with Tenofovir-Emtricitabine or Placebo in Botswana
Source: PLoS One. 2014 Mar 13;9(3):e90111. doi: 10.1371/journal.pone.0090111 (PMC3953113; doi:10.1371/journal.pone.0090111)
Supplement: Protocol S1 — Trial Protocol. (DOC) [file pone.0090111.s002.doc]

**Study of the Safety and Efficacy of Daily Oral Antiretroviral Use**

**For the Prevention of HIV Infection**

**in Heterosexually Active Young Adults in Botswana**

**Funded and Sponsored by**

Epidemiology Branch

Division of HIV/AIDS Prevention-Surveillance and Epidemiology

National Center for HIV, STD, and TB Prevention

U.S. Centers for Disease Control and Prevention (CDC)

1600 Clifton Road, Mailstop E-45

Atlanta, GA 30333 USA

**Conducted at**

The BOTUSA Project

A collaboration between the Government of Botswana and CDC

Box 90

Gaborone, Botswana

**Principal Investigator**

Michael C. Thigpen, MD, DTM&H

Associate Director

HIV Prevention Research Unit

The BOTUSA Project

**Co-Principal Investigators**

Lynn Paxton, MD, MPH

Team Leader, Antiretroviral Prophylaxis and Microbicides Team

Epidemiology Branch, DHAP-SE, NCHHSTP, CDC

Poloko M. Kebaabetswe, MPH, PhD

Senior Clinical Research Scientist

HIV Prevention Research Unit

The BOTUSA Project

**Pharmaceutical Support Provided by**

Gilead Sciences, Inc.

333 Lakeside Drive

Foster City, CA 94404

USA

Clinicaltrials.gov Registration Number NCT00448669

5 December 2007

TABLE OF CONTENTS

[PROTOCOL TEAM AND SITES 3](#__RefHeading___Toc153088215)

[PROTOCOL SUMMARY 5](#__RefHeading___Toc153088216)

[LIST OF ABBREVIATIONS AND ACRONYMS 7](#__RefHeading___Toc153088217)

[INTRODUCTION 9](#__RefHeading___Toc153088218)

[STUDY OBJECTIVES 15](#__RefHeading___Toc153088219)

[TRIAL DESIGN 15](#__RefHeading___Toc153088220)

[STUDY POPULATION 17](#__RefHeading___Toc153088221)

[STUDY VISIT SUMMARY 21](#__RefHeading___Toc153088222)

[SUBSTUDIES 30](#__RefHeading___Toc153088223)

[STUDY PRODUCTS 42](#__RefHeading___Toc153088224)

[CONCOMMITANT INTERVENTIONS 48](#__RefHeading___Toc153088225)

[CLINICAL SAFETY EVENT MANAGEMENT AND REPORTING 50](#__RefHeading___Toc153088226)

[SOCIAL TRIAL RISKS 55](#__RefHeading___Toc153088227)

[STATISTICAL SUMMARY 56](#__RefHeading___Toc153088228)

[TRAINING PLAN 64](#__RefHeading___Toc153088232)

[MONITORING PLAN 64](#__RefHeading___Toc153088233)

[LOCAL ADVISORY GROUPS 67](#__RefHeading___Toc153088234)

[DATA MANAGEMENT PLAN 67](#__RefHeading___Toc153088235)

[ADMINISTRATIVE PROCEDURES 69](#__RefHeading___Toc153088236)

[LABORATORY SPECIMENS AND BIOHAZARD CONTAINMENT 71](#__RefHeading___Toc153088237)

[ETHICS AND RESEARCH INTEGRITY 74](#__RefHeading___Toc153088238)

[PUBLICATION AND PRESENTATION POLICY 79](#__RefHeading___Toc153088239)

[APPENDICES 80](#__RefHeading___Toc153088240)

[CONSENT FORMS 81](#__RefHeading___Toc153088241)

[RECRUITMENT AND PARTICIPANT EDUCATION MATERIALS 115](#__RefHeading___Toc153088242)

[VISIT REIMBURSEMENT TABLE 201](#__RefHeading___Toc153088243)

[LABORATORY TESTING 204](#__RefHeading___Toc153088244)

[INVESTIGATOR’S BROCHURE 206](#__RefHeading___Toc153088245)

[DAIDS TABLE FOR GRADING THE SEVERITY OF ADULT ADVERSE EVENTS 263](#__RefHeading___Toc153088246)

[STI DIAGNOSIS AND TREATMENT GUIDELINES 278](#__RefHeading___Toc153088247)

[MANAGEMENT OF CLINICAL AND LABORATORY ADVERSE EVENTS 280](#__RefHeading___Toc153088248)

[MANAGEMENT OF CREATININE ELEVATION 281](#__RefHeading___Toc153088249)

[HIV TESTING ALGORITHMS 282](#__RefHeading___Toc153088250)

[STUDY PARTICIPANT DATA COLLECTION FORMS 284](#__RefHeading___Toc153088251)

[REFERENCES 424](#__RefHeading___Toc153088252)

**PROTOCOL TEAM AND SITES**

**Protocol Team Roster**

BOTUSA

Michael C. Thigpen, Principal Investigator

Poloko Kebaabetswe, Co-Principal Investigator

Margarett Davis, Investigator

Peter Fonjungo, Investigator

Sandra Johnson, Senior Data Manager

Tebogo Segolodi, Laboratory Coordinator

Joyce Kgampi, Protocol Operations Manager - Gaborone

Bakgaki Ratshaa, Acting Protocol Operations Manager – Francistown

Rodreck Mutanhaurwa, Medical/Pharmacy Officer – Gaborone

Evans Buliva, Medical/Pharmacy Officer - Gaborone

Lovemore Chirwa, Medical/Pharmacy Officer – Francistown

Daniel Abebe, Medical/Pharmacy Officer - Francistown

CDC, Atlanta

Lynn Paxton, Principal Investigator

Dawn K. Smith, Investigator

Kata Chillag, Investigator

Roman Gvetadze, Statistician

Peter Kilmarx, Investigator

Kevin Malotte, Investigator (IPA, California State University, Long Beach)

**Clinical Laboratories**

BOTUSA HIV Prevention Research Laboratories - Gaborone

Nyangabgwe Referral Hospital Cytology Laboratory – Francistown

National Health Laboratory, Cytology section – Gaborone

Lancet Laboratories, Francistown

Diagnofirm Laboratories, Gaborone

Johns Hopkins University (antiretroviral drug levels), Baltimore, MD

Medical Imaging Botswana, Gaborone

Pathcare, Cape Town, South Africa

**Institutional Review Boards**

Botswana Ministry of Health, Health Research Development Committee

(IRB 00002218, FWA 00005698, expiration 19 April 2009)

CDC IRB-B

(IRB 00000184, FWA 00001413, expiration 6 August 2010)

Johns Hopkins University School of Medicine

(IRB 00003794, FWA 00005752, expiration 15 December 2009)

**Trial Monitoring Consultants**

GCP Trial Monitoring, Pharmaceuticial Product Development Inc (PPDI), Wilmington, NC

Pharmacy Consultant, Pharmaceuticial Product Development Inc (PPDI), Johannesburg , RSA

Counseling Quality Assurance Monitoring, Institute for Development Management, Gaborone

Investigator Responsibilities

| Lynn Paxton | Scientific and operational responsibility for the trial |
| --- | --- |
| Michael C Thigpen | Scientific and operational responsibility for the trial |
| Poloko Kebaabetswe | Collaborate in design and management of the trial |
| Margarett Davis | Collaborate in the design and management of the trial |
| Peter Fonjungo | Scientific leadership of laboratory aspects of the trial |
| Tebogo Segolodi | Operational responsibility for laboratory aspects of the trial |
| Sandra Johnson | Operational responsibility data management aspects of the trial |
| Joyce Kgampi | Operational responsibility for conduct of the trial in Gaborone |
| Bakgaki Ratshaa | Operational responsibility for conduct of the trial in Francistown |
| Rodreck Mutanhaurwa | Physician, Accountability for study product in Gaborone |
| Evans Buliva | Physician, Accountability for study product in Gaborone |
| Lovemore Chirwa | Physician, Accountability for study product in Francistown |
| Daniel Abebe | Physician, Accountability for study product in Francistown |
| Eugene Jooste | Diagnostic Radiologist |
| Kata Chillag | Collaborate in design and scientific aspects of the trial |
| Roman Gvetadze | Trial statistician |
| Dawn K. Smith | Collaborate in design and scientific aspects of the trial |
| Peter Kilmarx | Collaborate in design of the trial |
| Kevin Malotte | Collaboration on counseling aspects of the trial |
| Craig Hendrix | Scientific leadership on pharmacology studies |

Trial Sites

The study will be conducted in free-standing research facilities in Gaborone and Francistown, Botswana. Botswana is a small country of 1.7 million inhabitants in southern Africa. It has been a stable democracy since 1966 and has a high literacy rate for both men and women. Gaborone, the capital city is located near the southern border, near to South Africa. Its population is about 215,000. Francistown, the second largest city, with a population of 100,000, is a five-hour drive from Gaborone, near the northeastern border with Zimbabwe. There are university campuses, national referral hospitals, and laboratories in both cities. BOTUSA has active TB and HIV programs and has done TB and perinatal HIV studies in both cities. For the conduct of HIV biomedical prevention studies, we have renovated a clinic space in each city with private interview, exam, and counseling rooms, a laboratory (CDC biosafety level 2) for limited onsite testing, and data management. We have established offsite support laboratories (e.g., for chemistry, hematology, PAP smears, EIA, viral load, and CD4 determination) in both cities (CDC biosafety level 3).

**PROTOCOL SUMMARY**

**Title:** Study of the Safety and Efficacy of Daily Oral Antiretroviral Use for the Prevention of HIV Infection in Heterosexually-Active Young Adults in Botswana

**Design:** A Phase III, randomized, double-blind, placebo-controlled trial evaluating the safety and efficacy of a single daily oral tablet containing 300 mg of tenofovir disoproxil fumarate(TDF) and 200 mg emtricitabine (FTC) for the prevention of HIV infection.

Sexually-active young adult males and females will be randomized 1:1 to once daily TDF/FTC or to placebo. HIV-uninfected participants will remain on assigned medication until the last enrolled participant has completed 12 months on drug.

**Population:** 1200 HIV-uninfected sexually-active young adults ages 18-29 in Francistown and Gaborone, Botswana.

**Study Duration:** The trial is estimated to take 36 months: 15 months for accrual, an additional 12 months to complete follow-up, 3 months for close-out, and 6 months for analysis. All subjects will remain on study medication until the last enrolled patient completes 12 months of dosing.

**Test Products:** Tenofovir DF 300 mg and Emtricitabine 200 mg or matched placebo, QD

po

**Study Procedures:** Targeted interviews, physical examinations, and/or laboratory evaluations will occur at screening, baseline, and monthly visits while on study drug and for those who seroconvert (and are taken off drug).

**Objectives:** The primary objectives of the study are:

- To evaluate the extended safety of daily TDF/FTC compared to placebo in sexually active young adults
- To evaluate the efficacy of daily TDF/FTC in reducing heterosexual HIV transmission in young adults

The secondary objectives of the study are:

- - - To evaluate the adherence of young adults to daily TDF/FTC or placebo
    - To evaluate changes in sexual behavior and condom use in the trial
    - To evaluate early virologic and immunologic responses to HIV exposure and seroconversion while receiving TDF/FTC

**Endpoints:**

Safety: ▪ The frequency of grade 3-4 renal or hepatic function laboratory, or

clinical, toxicities

▪ The frequency of adverse clinical events

▪ % change in bone density of those receiving TDF/FTC

Efficacy: ▪ The rate of HIV seroconversion

Other: ▪ Change in rates of unprotected vaginal sex

▪ The rates of secondary and periodic abstinence

▪ Adherence to daily medication doses

▪ Acceptability of TDF/FTC and trial procedures

▪ Antiretroviral sensitivity of HIV acquired by seroconverters

▪ Viral set point of seroconverters with/without TDF/FTC exposure

▪ Mucosal immunologic response to HIV exposure without

seroconversion

**LIST OF ABBREVIATIONS AND ACRONYMS**

ACASI audio computer assisted self-interview

AE adverse event

AIDS acquired immunodeficiency syndrome

ALT (SGPT) alanine aminotransferase

ART antiretroviral therapy

AST (SGOT) aspartate aminotransferase

BMD bone mineral density

BOTUSA PROJECT a collaboration between the Government of Botswana and CDC

BV bacterial vaginosis

CDC U.S. Centers for Disease Control and Prevention

CPT cell preparation tube

CRF case report form

CTL cytotoxic T-lymphocyte

CT chlamydia trachomatis

DEXA Dual-energy X-ray absorbiometry

DL deciliter

DSMB data safety monitoring board

DRU Drug Regulatory Unit, MOH, GOB

EC Ethics Committee

EDTA Ethylene diamine tetra acetic acid

ELISA enzyme-linked immunosorbant assay

FDA U.S. Food and Drug Administration

FP family planning

FTC 5-fluoro thio analogue of cytidine also called emtricitabine

g gram(s)

GC gonorrhea

GCP Good Clinical Practice guidelines

GOB Government of Botswana

HAART highly active antiretroviral therapy

HIV-1C human immunodeficiency virus, type 1, clade C

HRDC Health Research Development Committee, MOH, GOB

ICH International Conference on Harmonization

IRB Institutional Review Board

ITT intent to treat analysis

IU international unit(s)

kg kilogram(s)

LLN lower limit of normal

MCH maternal and child health

mg milligram(s)

ml milliliter(s)

mm^3^ cubic millimeter(s)

MOH Ministry of Health

NNRTI non-nucleoside reverse transcriptase inhibitor

NRTI nucleoside reverse transcriptase inhibitor

NtRTI nucleotide reverse transcriptase inhibitor

PBMC peripheral blood mononuclear cell

PCR polymerase chain reaction

PD pharmacodynamics

PEP post-exposure prophylaxis

PI protease inhibitor

PK pharmacokinetics

po by mouth

PrEP pre-exposure prophylaxis

py person-years of observation

QA quality assurance

QC quality control

RPR rapid plasma reagin

RT reverse transcriptase

RTI reverse transcriptase inhibitor

SAE serious adverse event

SIV simian immunodeficiency virus

STI sexually transmitted infection

TDF Tenofovir Disoproxil Fumarate

µg microgram(s)

ULN upper limit of normal

1. **INTRODUCTION**
   1. **Background**

25 years after its recognition, the HIV epidemic continues to spread aggressively. While relatively stable in developed nations of North America and Europe, it has reached extreme levels in southern Africa, and is growing rapidly in the two most populous Asian nations, India and China.[[1]](#endnote-2)

Changes in sexual behavior (e.g., delay in onset of sexual activity in youth, reduction in the number of partners – including mutual monogamy, and increased condom use) have helped decrease HIV transmission in a few countries. However, it is likely to be many years before large scale behavior change will occur in most other countries with high or increasing HIV prevalence. And in yet other countries, the availability of highly active antiretroviral therapy (HAART) and “AIDS battle fatigue” have led to reversals in gains achieved earlier with sexual behavior change, resulting in increases in HIV infection rates.[[2]](#endnote-3) As HAART is made increasingly available in Africa and other resource-constrained settings, stabilization or lowering of HIV prevalence rates may also begin to be eroded as the population of HIV-infected persons grows and fear of the consequences of HIV infection decreases. While condoms are an effective HIV prevention technology for those who continue to be sexually active if used consistently and correctly[[3]](#endnote-4), despite two decades of education, social marketing, and distribution of free or inexpensive condoms, they are insufficiently used to impede the worldwide epidemic.

In a setting like Botswana, where despite continuing active promotion of the ABC prevention strategy, HIV incidence is still high, 89% of childbearing women of all ages are unmarried, and 31% of 20-24 year-old childbearing women are already HIV positive, there is need both to strengthen behavioral prevention methods and supplement them with effective biomedical ones. Since preventive vaccines and microbicides now in development are still many years, if not decades, from being proven effective and made available for widespread use, it is critical that we explore other biomedical interventions that may be effective in the shorter term, particularly for those who are unable or unwilling to respond to currently available behavioral interventions. Pre-exposure prophylaxis (PrEP) with TDF/FTC, a once-daily combination antiretroviral pill with low toxicity and slow development of resistance mutations, may be such a new intervention with the capability to significantly reduce HIV transmission in high incidence settings worldwide.

- 1. **Why PrEP?**

There is ample evidence in animals and humans that post-exposure antiretroviral prophylaxis, if given promptly, can reduce the risk of acquiring HIV infection. In the case of health care workers with needle-stick injuries from HIV-infected patients, AZT monotherapy is estimated to reduce the risk by 81%[[4]](#endnote-5). Observational studies have provided evidence of efficacy in newborns whose HIV infected mothers were not given antiretrovirals during pregnancy but only during delivery[[5]](#endnote-6) and in gay/bisexual men with sexual exposure[[6]](#endnote-7). It is biologically plausible and consistent with data from both perinatal HIV prevention studies and other infectious diseases that daily prophylaxiswould be at least as effective and likely more so since medication would be taken both before and after exposure.

Early in the epidemic, the power of combined behavioral and biomedical interventions was demonstrated in the reduction of transfusion-associated HIV transmission. Initially, donor-deferral based largely on sexual risk history significantly reduced seroprevalence in blood donations. When the first HIV test was developed and blood could be effectively screened, the number of infected units transfused became negligible. In a similar way, it is possible that adding antiretroviral prophylaxis to existing behavior change and condom promotion efforts will increase significantly our ability to reduce HIV spread.

- 1. **Why TDF/FTC?**

Suggested criteria for an ideal PrEP agent include the following:[[7]](#endnote-8)

- Potent antiretroviral
- Rapidly bioavailable and long duration of action
- Easily administered on a once-daily or less dosing schedule
- Safety history shows low rates of clinical toxicity or hypersensitivity and well tolerated
- Favorable drug interaction profile for commonly used medications
- High “genetic barrier” to the emergence of drug resistance
- Affordable

TDF/FTC meets all these criteria. In animal studies, TDF has shown the ability to protect non-human primates against IV, oral, and mucosal SIV challenge, although both TDF doses and viral innocula were substantially greater than what occurs in humans.[[8]](#endnote-9) `And in a recent macaque study with weekly mucosal SHIV challenges, while TDF or FTC alone provided some protection, the combination provided complete protection at 14 weeks.[[9]](#endnote-10)

**TDF+FTC (SC 22 and 20 mg)**

**FTC (SC 20 mg)**

**TDF (Oral 22 mg)**

**Control**

**For FTC the exact log-rank p-value = 0.005 For the 4 TDF (daily) the exact log-rank p-value = 0.095**

**Cox PH 3.9 times (p-value = 0.021)**

**The average number of exposures till infectionThe median number of exposures till infection**

**TDF; 7 FTC; 10TDF; 6 FTC; 11**

**Percent Survival**

**Weekly Exposure**

**Survival Proportions**

**100**

**75**

**50**

**25**

**0**

**0**

**4**

**6**

**8**

**10**

**14**

**2**

**12**

In addition, TDF/FTC is approved for use in treating HIV infection in both the US and Europe, there is safety data for treatment use of TDF in several African countries, TDF/FTC has minimal drug-drug interactions, its clinical contraindications are uncommon among HIV-negative young adults, and it has received a Class B designation by the US FDA as “Animal studies have revealed no evidence of harm to the fetus, however, there are no adequate and well-controlled studies in pregnant women.” To date, there are no preclinical, pharmacokinetic, or clinical use data suggesting sex or race differences in TDF or FTC absorption, blood levels, or effectiveness in suppressing HIV replication.

- 1. **Transition from the Botswana TDF trial**

BOTUSA HPR conducted a safety trial of TDF vs placebo in Gaborone and Francistown. When the new information about the potential superiority of TDF/FTC was presented, discussion ensued and a decision was taken to transition from the TDF trial to a TDF/FTC trial. Enrollment in the TDF trial was halted but follow-up of those already enrolled continued. When the TDF/FTC trial was ready to begin, all participants in the TDF trial were offered enrollment in the new trial and the TDF trial was closed.

Meetings were held with stakeholders, advisory groups, and current trial participants about whether and how best to replace the TDF trial with the TDF/FTC trial. All were supportive that since data indicated the new two-drug regimen might be more effective, has no expected increase in safety concerns, and offers less concerns about resistance, we should proceed with the transition. Current trial participants suggested calling the new trial the “TDF2 trial”. They felt this name would be easier than some other options (e.g., TDF/FTC trial), less likely to suggest that participants were HIV-infected (e.g., TDF Plus trial), and reinforces that we are not dropping TDF but adding FTC to it. (i.e., using FTC too, the second TDF prophylaxis trial, uses 2 drugs).

In addition, some lessons learned from the TDF trial so far have been incorporated in refinements to procedures and instruments reflected in the protocol, which otherwise is based on the currently approved TDF trial protocol. These lessons will be referenced in the relevant places throughout this protocol.

- 1. **Why Botswana Young Adults?**

Botswana is one of the most intensively infected country in the world with an estimated 28% of the entire population 15-49 years of age infected with HIV in 2005[[10]](#endnote-11) and 17% of the population ages 6 months to 64 years infected in 2004[[11]](#endnote-12). We have chosen to do the TDF/FTC trial with sexually active young adults in part because of the great need in this population with rapidly rising prevalence over the ages included in the trial population (indicative of high incidence), and in part because the recruitment pool of seronegative participants becomes increasingly limited as age increases.

Calculated from the 2005 surveillance in childbearing women^10^ and data from the 2004 nationwide Botswana AIDS Impact Survey II^11^, 18% of all young women ages 15-19 are infected, 30% of those ages 20-24, and 44% of those ages 25-29. Among young men, peak infection occurs later but 6 % of those ages 15-19, 11% of those ages 20-24, and 25% of those ages 25-29 are infected. In all districts, measured prevalence in childbearing women was over 20%.

In Gaborone and Francistown, the two sites where the trials will be conducted, HIV prevalence rates in young age groups is higher than the national average.

|  | 15-19 | 20-24 | % increase  from 15-19 to 20-24 | 25-29 | % increase  from 20-24 to 25-29 |
| --- | --- | --- | --- | --- | --- |
| Males, Gaborone | 5.0 | 10.3 | 106% | 22.0 | 113% |
| Males, Francistown | 8.2 | 11.7 | 43% | 29.3 | 150% |
|  |  |  |  |  |  |
| Females, Gaborone | 23.5 | 29.6 | 26% | 39.3 | 33% |
| Females, Francistown | 25.5 | 33.3 | 31% | 52.4 | 57% |

The high incidence in young women was recently confirmed in a seroincidence (STARHS) study of 2600 pregnant women in southeastern Botswana where HIV incidence was found to be 10% in those 15-19 and 15% in those 20-24 years old [[12]](#endnote-13). This may overestimate incidence in the general population of sexually active young adult women for technical assay reasons, and for population reasons as may antenatal prevalence data[[13]](#endnote-14). However, for both young men and young women, the rate of increase in prevalence between adjacent age groups, strongly suggests high incidence in these age groups for both genders in the cities where the trial will be done.

Therefore HIV prevention trials focused on developing additional options, especially for young people, are urgently needed in Botswana. There is multisectoral support for HIV prevention research and the demonstrated political will and financial resources to implement prevention approaches shown to be effective here. Interventions like PrEP, if proven effective in Botswana will have impact throughout sub-Saharan Africa and many other settings in the world where heterosexual transmission among youth is driving the HIV epidemic.

Botswana may also be a good choice because HAART is already being provided by the government to citizens making treatment (when indicated) readily available to seroconverters in the trial. The first line regimen (94% of patients) is Combivir (zidovudine+3TC) with Nevirapine for women of child-bearing potential and Combivir with Efavirenz for all others**.** Tenofovir is used for treatment only in some of those failing the second line regimen in Botswana. Emtricitabine is not used in the treatment program. Consequently, TDF and FTC-associated resistance mutations are rare. In surveillance among untreated HIV positive persons, no primary mutations were found against NRTIs, NNRTIs, or PIs [[14]](#endnote-15). Among 16 patients with virologic failure on the first line regimens, 3 of 8 on CBV/NVP had the K65R mutation, 0 of 8 on CBV/EFV had this mutation most strongly associated with TDF resistance. 4 of 16 (2 on CBV/EFV and 2 on CBV/NVP) had the M184V (without the K65R) mutation which is associated with FTC resistance [[15]](#endnote-16) and with slight increase in sensitivity to TDF.[[16]](#endnote-17) The virologic failure rate at 6 months among HAART patients in Botswana is 2%. Therefore it is unlikely that persons on TDF/FTC trial in Botswana will encounter resistant virus which might compromise any prophylactic efficacy of the combination medication[[17]](#endnote-18).

- - 1. **Bone Mass**

Osteopenia and osteoporosis are both associated with HIV infection, especially among those being treated with HAART (without Tenofovir).[[18]](#endnote-19) In studies of pregnant macaques given very high doses of TDF during pregnancy (6-12 times the dose to be used in this study), there was significant reduction in bone mineralization in the infants.[[19]](#endnote-20) However, in adult human TDF trials (in HIV-infected persons) to date, fracture rates have been lower in the TDF arm than in the placebo arm and nearly all fractures resulted from trauma[[20]](#endnote-21). Reports from a recent pediatric treatment trial in which TDF was added to optimized HAART regimens for 48 weeks, measured lumbar spine bone mass density (BMD) at baseline, 24, and 48 weeks. At baseline, median BMD z-scores were already depressed (-1.18); by 24 weeks, 10/18 children had decreases (median -0.38) from baseline; at 48 weeks 5/18 subjects had decreases from baseline (median -0.31). Analysis of bone biomarker data showed evidence of increased bone resorption.[[21]](#endnote-22) FTC has no known effect on BMD.

Bone mineralization is accelerated during the adolescent and young adult period[[22]](#endnote-23) and any factors that affect mineralization during this period may interfere with peak bone mass and possibly increase the risk of fractures in later life. Gender differences in both age at peak bone mass and rates of bone mass accumulation in youth have been demonstrated. In females, incremental rate of bone density was most pronounced between ages 11 and 14 and fell markedly after age 16 with no significant annual incremental gains between ages 17 and 20. In males, the rate of BMD increase was most pronounced from ages 13-17 and while the rate declined, significant increases occurred through age 20 for some measured sites.[[23]](#endnote-24) So it will be important in this study to examine the effects of TDF on recently established bone density in uninfected young adults as part of determining the long-term effects of PrEP and weighing any worrisome findings against the risk of HIV infection. If PrEP is protective against acquiring a fatal HIV infection, the benefit of this effect would outweigh a possible harm from osteopenia or osteoporosis later in life, particularly when there are simple and affordable preventive therapies (e.g., vitamin D and calcium supplementation) available.

While TDF has been shown not to have significant pharmacokinetic drug interactions with oral contraceptives [[24]](#endnote-25), the effects of hormonal contraception on bone density will also need to taken into account in this young adult study population. The two most common hormonal methods used in Botswana are injectable progestins and oral contraceptives. Overall there is fair evidence that low-dose oral contraceptives have positive effects on BMD although some studies have shown no effect.[[25]](#endnote-26) On the other hand, Depo-medroxyprogesterone acetate (e.g., Depo-Provera) use is associated with losses in bone density in adult women but recovery occurs on discontinuation.[[26]](#endnote-27) An effect similar to that on BMD has been shown for biochemical markers of bone metabolism suggesting that bone resorption exceeds bone formation.[[27]](#endnote-28)

- - 1. **Adherence behavior in young adults**

Experience is limited, but rapidly increasing, with ART in Botswana (and most developing countries) and the number of young adults receiving treatment is still low. So we have no directly applicable information about the adherence of young adults to antiretroviral medications. In general, adherence for treatment in Botswana is very high with >80% of patients on HAART for 6 months having undetectable virus. However, the motivations to take a single antiretroviral for prevention may be entirely different from taking multiple drugs for a perceived immediate survival benefit.

- - 1. **Condom migration and sexual disinhibition**

In all populations being studied for PrEP and other biomedical interventions (e.g., vaccines, microbicides), concerns have been raised about “condom migration” and other forms of sexual disinhibition. It is possible that trial participants will, despite education otherwise and the use of a placebo, believe that they are protected by the study medication and will reduce their condom use or otherwise increase behaviors that may put them at risk of HIV acquisition. Data are available from previous prevention trials in similar populations to suggest that this may not occur frequently enough to represent a significant increase in HIV transmission risk. In a recent placebo-controlled microbicide safety study in South Africa where women were instructed to use both condoms and gel at each vaginal sex episode, there were an equal number of HIV transmissions in each arm. There was no increase in the number of partners reported by women during the trial. Combined gel and condom use was reported in more than half of sex acts, gel only in about a third, condoms only in about a tenth, and neither gel nor condoms were used in a little more than 10% of acts.[[28]](#endnote-29) This represents a significant increase in condom use for this population. And in a Phase II microbicide study in Uganda, all women reported condom use at least as, or more frequently as before the trial.[[29]](#endnote-30)

- 1. **Preparatory Community Consultation and Research**

The HIV Prevention Research Section of BOTUSA has been working for the past few years to develop effective mechanisms for increasing community awareness and involvement in its research studies. These mechanisms have been used to consult with community segments about the TDF PrEP study. We have met with national and local government officials in the National AIDS Coordinating Committee as well as the Ministries of Health, Education, and Local Government. We have discussed the study with the HIV Prevention Research Reference Group, a standing advisory group composed of government unit representatives from both Gaborone and Francistown (e.g., Senior District Medical Officers, Family Health Division [MCH and FP services], Drug Regulatory Unit, AIDS/STD Unit, National AIDS Coordinating Committee). Discussions have been started with CBOs which will be ongoing throughout the trial (e.g., Botswana Family Welfare Association [BOFWA]; Youth Health Organization [YOHO]; Botswana Network on Ethics, Law, and HIV/AIDS [BONELA]; Botswana Christian AIDS Intervention Program [BOCAIP]).

Community Advisory Groups are well established in Francistown and Gaborone and include several young adult members and will provide ongoing advice and a communication conduit with the trial communities.

In addition, information obtained from preparatory research for HIV prevention trials already completed, and from the TDF PrEP Phase II trial now in the field, have been tapped to inform the processes of this trial. This includes: 1) quantitative and qualitative data on comprehension of HIV prevention trial concepts (e.g., blinding, randomization) and attitudes about possible trial research participation, 2) performance of sexual behavior and condom use questions (through both face-to-face interviews and audio-computer delivered interviews (ACASI), comprehension of informed consent, and selected interviews, and 3) experience with factors affecting recruitment, retention, and medication adherance measurement.

**STUDY OBJECTIVES**

- 1. **Primary Objectives**
- To further evaluate the safety of 300 mg TDF and 200 mg of FTC in a single pill po daily in HIV uninfected, healthy young adults in Botswana
- To evaluate the efficacy of 300 mg TDF and 200 mg of FTC in a single pill po daily in reducing the incidence of HIV acquisition by healthy young adults in Botswana
  1. **Secondary Objectives**
- To evaluate the adherence of young adults to daily TDF/FTC and placebo
- To evaluate changes in sexual behavior and condom use over the duration of the trial
- To evaluate early virologic and immunologic consequences of HIV exposure and seroconversion while receiving TDF/FTC PrEP

1. **TRIAL DESIGN**

This is a Phase III, fully blinded, randomized, placebo-controlled trial to evaluate the safety and efficacy of 300 mg TDF and 200 mg FTC in a single pill po daily in HIV-1 uninfected young adults (ages 18-29) in Botswana.

The primary goal of the Phase III study is to assess the extended safety and efficacy of a simple antiretroviral regimen that would be appropriate for use to prevent sexual HIV transmission in resource-constrained settings with high HIV incidence. Assessment of adherence and changes in sexual behavior and condom use will also be done.

- 1. **Primary Endpoints**

The primary safety endpoints will be measured by:

- The frequency of Grade 3 or 4 renal or hepatic function laboratory toxicities or clinical toxicities in blinded TDF/FTC and placebo arms, as defined by the NIAID Division of AIDS (DAIDS) Table for Grading Severity of Adult and Pediatric Adverse Events (December 2004), and which cannot be directly attributed to a cause other than study medications
- the frequency of adverse clinical events in TDF/FTC and placebo arms
- % change (TDF/FTC compared to placebo) in BMD by DEXA scan every six months.

The primary efficacy endpoint will be measured by:

- Rates of HIV-1 seroconversion measured at monthly intervals
  1. **Secondary Endpoints**

Medication adherence will be measured as the proportion of participants who:

- By interview, report taking at least 80% of daily doses of assigned study medication
- By medication diary, report taking at least 80% of doses at during the same period of the day
- At visits measured, have adequate plasma and intracellular concentrations of TDF/FTC

Changes in sexual behavior and condom use will be measured by:

- The number of unprotected sexual acts reported over the course of the trial
- Rates of sexual partner change
- Rates of secondary and periodic abstinence

Virologic and immunologic responses to HIV exposure and seroconversion while receiving TDF/FTC

- Rates and nature of antiretroviral genotypic and phenotypic resistance detected in virus from seroconverters in the TDF/FTC and placebo arms
- Viral set point at 6 months and trajectory following seroconversion in trial participants with and without TDF/FTC exposure
- Immunological responses detected among trial participants who remain uninfected despite reported high risk of HIV exposure (with and without TDF/FTC exposure)
  1. **Study Design**

At two centers in Botswana, 1200 sexually-active HIV-seronegative young adult males and females (50% of each gender) will be enrolled and randomized 1:1 to once daily TDF (300 mg) with FTC (200 mg) or to placebo.

Participants will be followed monthly, with targeted examinations and interviews at defined intervals while on medication. HIV-uninfected participants will remain on assigned medication until the last enrolled participant has completed 12 months on drug. We are keeping those enrolled early in the study for longer than 12 months to contribute additional person/years to the final safety and efficacy analyses. Participants who acquire HIV infection after enrollment (seroconverters) will be taken off study medication and followed until the end of the study on the same visit schedule as uninfected participants.

- 1. **Duration of the Study**

Estimating that 35% of those screened are eligible and consenting, full accrual of 1200 Phase III participants is anticipated to take 15 months total (approximately 2 enrolled per site per work day). If the last person enrolled is followed for 12 months (and earlier enrollees followed longer, the timeline will be approximately as follows.

Months

|  | **1-12** | **13-24** | | **25-36** | | | | |
| --- | --- | --- | --- | --- | --- | --- | --- | --- |
| **Screen/Enroll** |  | |  | | | | | |
| **On-Drug** |  | | | |  | | | |
| **Off-Drug** |  | | | |  | |  | |
| **DSMB REVIEWS** | at either ½ of expected follow-up time or ½ seroconversions, and at closure | | | | | | | |
| **Closeout** |  | | | | |  | |  |
| **Analyses** |  | | | | |  | | |

1. **STUDY POPULATION**

We need to assess safety and efficacy in both males and females of all included ages during this Phase III trial. Because of differences in the target population sizes of the two cities, we anticipate that approximately 60% of participants will be recruited in Gaborone and 40% in Francistown.

- 1. **Inclusion Criteria**

For those enrolled in the TDF oral prophylaxis trial

- Completed scheduled study visit within prior 30 days
- Consenting to enroll into this trial

For those newly screened for this trial

- 18-29 years of age, inclusive
- Parental/Guardian consent if 18-20 years of age (pending lower age limit of 16 by law)
- Sexually active (at least one sex partner in the past 3 months)
- Citizen of Botswana
- Laboratory values as follows within 30 days prior to enrollment:
  - HIV uninfected by dual, parallel, rapid whole blood testing
  - Hemoglobin >8 gm/dL
  - ALT and AST < 2 x ULN
  - Total bilirubin <1.5 mg/dL
  - Serum amylase < 1.5 x ULN
  - Serum phosphorus >2.2 mg/dL
  - Hepatitis B surface Ag negative
  - Calculated creatinine clearance 60 mL/min by the Cockcroft-Gault formula where CrCl in mL/min =

Male: (140 – age in years) x (wt in kg)

72 x (serum creatinine in mg/dL)

Female: (140 – age in years) x (wt in kg) _x 0.85_

72 x (serum creatinine in mg/dL)

- Willing to use effective contraception during the trial (oral or injection hormonal contraception, an intrauterine device [IUD], or who have had surgical interventions such as bilateral tubal ligation or hysterectomy), if female
- Living within 1 hour travel distance of Francistown or Gaborone
- Pass comprehension test
- Willing and able to provide informed written consent for study participation
  1. **Exclusion Criteria**
  - Any chronic illness requiring ongoing prescription medication
  - Positive urine pregnancy test (females)
  - Breastfeeding (females)
  - Planning to move away from trial communities within 12 months
  - History of significant renal or bone disease
  - Any other clinical condition or prior therapy that, in the opinion of the study physician would make the subject unsuitable for the study or unable to comply with the dosing requirements
  - Current participation in any other HIV prevention trial or drug/vaccine safety trial
  1. **Exclusion of youth under the age of 18 and parental/guardian consent for those**

**ages 18-20**

Although many younger adolescents, especially those 15-17, are sexually active and therefore at risk of acquiring HIV infection, we will not enroll them in this trial. While NACA has made recommendations to lower the legal age for autonomous consent for HIV testing and care, including receipt of antiretroviral treatment, this change has not yet been enacted into law. Discussions were held with colleagues in the research community, the Botswana Ministry of Health, community stakeholders in Botswana, and senior staff at CDC and while the urgent need for effective prevention strategies in this young population was universally recognized, there was not a consensus that wisdom and ethics supported attempting to include them this clinical trial.

A recent advisory from the Attorney General’s chambers to the Ministry of Health has recommended that, at present, person under the age of 21 not be enrolled in clinical trials without parental/guardian consent. They advised further that when the age for autonomous consent of HIV testing and care is lowered by act of parliament (age 16 is recommended), they would similarly lower the age of participation in HIV clinical trials without parental/guardian consent to the enacted lower age. We therefore will enroll young adults 18-20 when we can obtain parental/guardian consent until the new age limit is established and will then enroll without parental/guardian consent.

- 1. **Recruitment**

Participants in the TDF oral prophylaxis trial who are receiving study drug within 30 days of the start of this trial, were offered the chance to roll-over into this TDF/FTC trial. Seroconverters being followed in the TDF trial were also offered the chance to continue follow-up in this TDF/FTC trial. We met with all participants in the current TDF trial (protocol 4321) before stopping enrollment to explain to them about the new animal data, the reasons for stopping enrollment, and the plans for the TDF/FTC trial being planned. They had opportunities to ask questions during the participant group sessions and in their subsequent individual meetings with trial counselors during follow-up study visits. Active TDF trial participants were required to complete the informed consent process for the TDF2 trial.

Additional participants will be recruited through multiple venues by use of outreach in public venues by study recruiters and by the use of community-based household recruitment. Recruitment sessions will also be held at voluntary counseling and testing centers and at STI and family planning clinics. We may also have study recruiters at these venues during some times. In addition, recruitment will be done by distribution of flyers to youth organizations (the definition of youth in Botswana goes up through age 29). Radio, newspaper, and TV advertisement (See Appendix B) will be used to inform interested young adults where to call or come for information about the study. All materials developed for these uses will be submitted to the community advisory boards and to IRB/ECs for approval before use.

In recognition of the hesitance some young adults ages 18-20 will feel about disclosing their sexual activity in order to obtain parental/guardian consent, we developed special recruitment strategies. For example, recruiting young mothers since their parents will already know they are sexually active, outreach and public advertisements targeting parents of 18-20 year olds, asking them to refer their adult children to the study and let them know they are willing to consider providing consent, and the use of household recruitment where both parents and eligibile youth can be educated about the trial at the same time.

Special efforts will be made to recruit seronegative young adults who believe or know their partners are HIV infected. Such youth are at especially high risk of acquiring HIV infection and will benefit from the risk reduction counseling, STI diagnosis and treatment, and perhaps TDF/FTC use. This will include soliciting referrals from HIV care providers and voluntary counseling and testing centers of seronegative young adults whose partners have tested HIV positive.

- 1. **Participant Retention**

Once a participant enrolls in this study, the study site will make every effort to retain them for the duration of scheduled follow-up to minimize possible bias associated with loss-to-follow-up. Components of retention procedures will include:

- Thorough explanation of the study visit schedule and procedural requirements during the informed consent process, and brief review at each study visit.
- Collection of detailed locator information during screening/enrollment, and active review and updating of this information at each study visit.
- Checking the accuracy of locator information between the screening and enrollment visit to ensure that retention contacts are possible during the trial. In the TDF trial, participants sometimes gave inaccurate or incomplete information at their screening visit.
- Use of appropriate, timely, and confidentially worded visit reminder mechanisms (e.g., cell phone reminders).
- Immediate follow-up on missed visits by trained outreach workers to complete in-person contact with participants at their homes and/or other community locations.
- Restricting female eligibility to those willing to use effective contraception (hormonal, IUD or who have undergone surgical interventions such as tubal ligation or hysterectomy) to prevent falling pregnant, as pregnancy requires withdrawal of study medication.

Retention rates are expected to be high because of the interest in the trial and the health checks and health information provided. With the frequent contact planned with monthly study visits and additional home contacts, we estimate moderate loss to follow-up (<15% in year 1 and <5% in year 2 of follow-up) based on regional experience with other longitudinal studies in similar populations.

- 1. **Participant Withdrawal**

Participants may voluntarily withdraw from this study for any reason at any time.

The Principal Investigator also may withdraw participants from the study drug temporarily or permanently to protect their safety and/or if they are unwilling or unable to comply with required study procedures. Such reasons include, but are not limited to:

- HIV seroconversion (See Section 4.3.7)
- Grade 2-4 creatinine elevation (See Section 7.4.2)
- Grade 3-4 elevation with recurrence of safety labs other than creatinine (See Section 7.4.1)
- Pregnancy (confirmed by 2^nd^ pregnancy test 1 month after first positive test)
- The onset of health conditions that compromise study participation
- Withdrawal from hormonal contraception in the first 6 months after enrollment
- Failure to attend 3 consecutive monthly visits or inability to contact a participant for 3 months.
- Imprisonment

Participants who are permanently discontinued from study treatment early for any reason will be asked to remain in the study.

Participants also may be withdrawn if the study sponsor or local government or regulatory authorities terminate the study prior to its planned end date.

- 1. **Study Drug Interruption**

Study drug may be interrupted temporarily in some circumstances (e.g.):

- the participant runs out of study drug because of a missed visit
- to allow resolution of a transient adverse event

In these cases, an HIV test (and a pregnancy test for females) will be done to confirm seronegative, nonpregnant status before resuming study drug administration.

- 1. **Co-Enrollment Guidelines**

Phase III trial participants may not be co-enrolled in other HIV prevention studies. However, participants who HIV seroconvert may enroll in non-drug studies for HIV-infected persons while completing their off-drug follow-up visits in this trial.

1. **STUDY VISIT SUMMARY**

Presented below is information on visit-specific study procedures. Detailed instructions to guide and standardize all study procedures will be provided in the study-specific procedures manual.

- 1. **Screening Visit**

Screening of young men and women for the Phase III study will proceed in a stepwise manner.

- Provide brief introductory information about the study
- assign a screening ID number
- conduct a brief screening interview (including demographic and eligibility questions)
- provide pre-test counseling and obtain consent for HIV testing
- obtain written consent for screening labs
- conduct dual, concurrent HIV rapid tests from a fingerstick sample
- conduct urine pregnancy test for females
- those testing HIV positive or pregnant will be referred for additional counseling and medical care
- collect blood from those who are eligible by interview criteria and not pregnant
  - HIV neg (1 serum and 1 purple tube) for chemistry, serology, and hematology eligibility tests, and HIV confirmatory/QA testing;
  - HIV pos (1 CPT and 1 purple) for CD4 and repository and seroincidence testing[[30]](#endnote-31)
- if eligible by interview and rapid testing, allow participants to use a touch-screen, computer-based study education program, then interviewer will review the description of the trial and answer questions.
- provide post-test risk-reduction counseling
- collect detailed locator information, take photograph, and give an appointment in the following 1-2 weeks when eligibility laboratory test results will be available.

HIV test consent

Potentially Eligible

Oral Consent to Screening Interview (S01)

Screening Interview

Multi-media trial education criteria

Blood draw

for referral to care

*(EIA, CD4)*

Return to clinic (S02) for lab results

*(final eligibility determination)*

Potentially Eligible

NOT ELIGIBLE

*(by pregnancy or HIV)*

Comprehension Test

Retake Comprehension Test

Return to the clinic (S03)

Passed Test

ELIGIBLE

Failed Test

NOT ELIGIBLE

*(exit study)*

Failed Test

Potentially Eligible

NOT ELIGIBLE

*(exit study)*

Passed Test

ELIGIBLE

HIV+

HIV-

*(exit study)*

NOT ELIGIBLE by interview

(exit study)

Potentially Eligible by Interview

Written Consent to Screening Labs

Multi-Media Trial Education

Pre-test Counseling

Written Consent to HIV test

No HIV test consent

NOT ELIGIBLE (exit study)

Blood draw for lab eligibility

Return to clinic (S02)

for lab results

*(exit study)*

- 1. **Screening Revisit and Enrollment Visit**
     1. **Screening Revisit**
- administer comprehension test.

If failed, go over incorrect answers and schedule retest at next visit (within 30 days of initial screening visit)

- determine final trial eligibility by review of laboratory tests and the score on a comprehension test (>80% of items answered correctly including Q16 and Q18.
  - 1. **Enrollment Visit**  (preferably on the same day as successful Screening Revisit)

- those meeting all eligibility criteria will be asked to provide written consent for trial participation.
- assign enrollment ID to consenting participants.
- administer baseline interviews with ACASI used for sexual behavior and condom use questions
- conduct a targeted medical history and physical exam, including pelvic exam for women, and genital exam for men. (Women who are menstruating during their enrollment visit will be re-scheduled at a later date for a complete physical exam including pelvic exam. All other procedures will still be completed.)
- collect blood (one serum and one CPT tube), urine, and genital tract samples for pregnancy and STI testing and evaluation of laboratory-based safety criteria.
- provide any indicated STI treatment based on stat STI lab test results.
- provide family planning and medication adherence counseling.
- dispense condoms and any other indicated contraception.
- dispense a month’s supply of daily study medication according to assigned enrollment ID.
- schedule appointments for the duration of study participation
- obtain a baseline DEXA scan (for the first 100 males and first 100 females enrolled in Gaborone) the same day if possible, otherwise by appointment within 1 week.
  1. **Follow-up Visits**

Monthly visits will be conducted in order to: 1) reduce the duration of monotherapy for those who seroconvert which may facilitate the development of antiretroviral resistance that would complicate future treatment; 2) reduce recall bias in measures of adherence and sexual risk behavior; 3) limit the amount of drug in the community at any given time where it may be susceptible to theft, sharing, or loss; and 4) closely monitor symptoms, toxicities, and adverse events. These frequent visits are designed to be brief and will be supplemented by more extensive data collection done quarterly.

For those who seroconverted in the TDF trial or who seroconvert while in this study, monthly visits will serve to provide ongoing supportive counseling and education about HIV infection and to study the psychosocial, immunologic, and virologic consequences of seroconversion with/without TDF/FTC exposure.

For participants who permanently discontinue study treatment early for any reason (other than seroconversion) will be asked to remain in the study and will continue to receive HIV risk reduction counseling and condoms or other contraceptives, but will not undergo other parts of each visit noted below.

- - 1. **Monthly Visits**

Visit windows will be set at the midpoints between scheduled visits calculated in months from the date of the enrollment visit.

At each monthly visit on study medication, the following will be done:

- a clinical interview to elicit symptoms and off-study medical care
- a brief interview to assess medication adherence*
- a brief interview to assess sexual behavior and condom use
- do a urine pregnancy test for females*
- do an oral transudate rapid HIV test
- collect/count unused medication doses and dispense a new one-month supply*
- dispense condoms, contraceptives (if indicated)
- collect and review medication diaries*
- provide medication adherence counseling*

* Participants who have been taken off the study intervention for any reason will not undergo these parts of the visit.

First two monthly visits only

- collect a CPT tube for plasma/PBMC repository (Participants who have been taken off the study intervention for any reason will not undergo this blood draw.)

For seroconverters monthly visits will include:

- a brief interview about psychosocial responses to HIV infection
- a supportive counseling session
- an education session (first month only, then on request)
  - 1. **Quarterly Visits**

In addition to the monthly procedures, for both HIV negatives and seroconverters:

- at the 3, 6, 9, 12, 15, 18, (21, 24) month visits:
- conduct an additional sexual behavior interview
- collect a CPT tube for plasma/PBMC repository*
- collect a serum tube (safety labs for seronegatives, repository for both)*
- provide HIV risk reduction follow-up counseling
- update locator information

* Participants who have been taken off the study intervention for reasons other than seroconversion will not undergo these parts of the visit.

For all HIV negatives (including the 30 participating in the PK/PD substudy) at the first quarterly visit only, for PK/PD assessment (See Section 6.5):

- collection information on the times medication was taken for the prior 2 days
- collect 3 CPT tubes and 1 EDTA tube at the start of the visit (for pre-dose plasma and intracellular drug level testing)
- observe the daily medication dose
- collect a 2 CPT tubes and 1 EDTA tube 2 hours after taking their medication (for post-dose plasma and intracellular drug level testing)
  - - 1. Pharmacokinetics/Pharmacodynamics Substudy Participants Only

At the first quarterly visit only for 30 seronegative participants consenting to PK/PD Substudy (See Section 6.5), in addition to the above:

- collect 1 EDTA tube at 1, 4, and 8 hours after taking their medication dose (for post-dose plasma drug level testing)
- collect 2 CPT tubes at 4 and 8 hours after taking their medication dose (for intracellular drug assays)
  - 1. **Semi-Annual Visits**

In addition to the monthly and quarterly procedures, for both HIV negatives and seroconverters

- at the 6, 12, 18 (24) month visits:
- ask extended sexual behavior and condom use questions by ACASI
- do a physical exam (including pelvic/genital exam)*
- collect genital tract samples for STI testing and repository*

For seronegatives only

- administer comprehension test (visit-specific version)
- do DEXA scans for the subset of 200 in Gaborone

For seropositives only

- collect a purple-top for CD4 testing
- measure viral load on plasma from CPT tube collected for repository

* Women who are menstruating during the semi-annual visits will be re-scheduled at a later date for a complete physical exam including pelvic exam. All other procedures will still be completed.

- - 1. **Exit Visit for Enrolled Participants**

When exiting the study for any reason the following will be done:

- Administer study exit interview
- collect blood and urine samples for pregnancy, safety labs, and serum/plasma/PBMC repository
- do an oral transudate HIV test and provide post-test counseling (if positive) (unless seroconverted at a prior visit)
- dispense condoms
- update locator information
  - 1. **Interim Visits**

Participants will be instructed to return to the clinic for evaluation (without an appointment) whenever they may experience symptoms or illness that may be related to study medication or if they have signs or symptoms of STIs. Assessments will be done by study clinicians, findings entered into study records, treatment or referral for care provided as indicated, and any adverse event reporting indicated will be promptly completed.

Participants may also be recalled for interim visits when labs return with results that merit retesting and/or discontinuation of study medication

**Schedule of Main and Substudy Visits**

|  | **Enr** | **Mo**  **1** | **Mo**  **2** | **Mo**  **3** | **Mo**  **4** | **Mo**  **5** | **Mo**  **6** | **Mo**  **7** | **Mo**  **8** | **Mo**  **9** | **Mo**  **10** | **Mo**  **11** | **Mo**  **12** | **Mo**  **13** |
| --- | --- | --- | --- | --- | --- | --- | --- | --- | --- | --- | --- | --- | --- | --- |
| Main Study Visits | **X** | **X** | **X** | **X** | **X** | **X** | **X** | **X** | **X** | **X** | **X** | **X** | **X** |  |
| Dexa Substudy  (n=200 in Gabs only) | **X** |  |  |  |  |  | **X** |  |  |  |  |  | **X** |  |
| Qualitative Substudy* (n=24) |  |  |  |  | **X** |  |  | **X** |  |  | **X** |  |  | **X** |
| PK/PD Substudy  (n=30) |  |  |  | **X** |  |  |  |  |  |  |  |  |  |  |

* 12 selected at Mo3 with interviews at months 4, 7, and 10; 12 selected at Mo6 with interviews at months 7, 10, and 13

- 1. **Primary Measured Outcomes – Phase III**

|  | **Screening** | **Enrollment** | **Follow-Up**  **(on drug)**  monthly | **Exit Visit**  **(off drug)**  +4 wks | **Measures** |
| --- | --- | --- | --- | --- | --- |
| **TDF/FTC or PLACEBO Use/Safety/Tolerability** | | | | | |
| Use/Adherence |  |  | X |  | % dispensed pills returned  % doses missed/mistimed (diary and interview)  drug levels |
| Safety |  |  | X | X | rate, grade, duration of lab toxicities, by arm  rate, grade, duration of clinical toxicities, by arm  AE/SAE type and number, by arm |
| Tolerability |  |  | X | X | rate of reported symptoms, by arm |
| Acceptability |  |  |  | X | best/worst features |
| Bone health  (subset) |  | X | X  (q 6 months) |  | % change in BMD by DEXA (200 in Gabs only) and vitamin D levels at baseline |
| **HIV Exposure/Transmission** | | | | | |
| Sexual Behavior | X  (limited) | X | X  (ACASI q 6 months) | X | age at first sex, # partners, transactional sex, recent vag/oral/anal sex, etoh with/without condoms |
| Condom Use | X  (limited) | X | X  (ACASI q 6 months) | X | ever, recent freq with vag sex by partner type, baseline and change |
| HIV Status | X |  | X | X | seroconversion |
| Genital Exam |  | X | X  (q 6 months) |  | circumcision (males)  cervical ectopy (females) |
| STI/Genital Health |  | X | X  (q 6 months) |  | GC, CT, syphilis, trich, ulcers, BV, vaginitis |
| Pregnancy | X | X | X | X | falling pregnant (F), reported partner pregnancy (M) |
| **Participant Procedures** | | | | | |
| Screening consent | X |  |  |  | Rate/reasons for refusing  Rate/reasons for consenting |
| HIV consent | X |  |  |  | % refusing HIV test |
| Comprehension | X |  | X  (q 6 months) |  | Score on comprehension test |
| Eligibility | X | X |  |  | % eligible, % by reasons for ineligibility |
| Enrolled consent |  | X |  |  | Rate/reasons for refusing |
| Visit compliance |  | X | X | X | % visits completed, % lost to follow-up |

- - 1. **HIV Seroconversion**

When any enrolled person who tests HIV positive by oral transudate testing (Oraquick) during a study visit, the following will occur at that visit:

- Dual, parallel, fingerstick rapid HIV tests will be done to confirm the positive oral result for purposes of counseling the participant at that visit.
- Blood samples will be taken to confirm HIV seroconversion by EIA, to measure baseline viral load and CD4 count (required for the national antiretroviral treatment program), and to measure resistance mutations (by standard and ultrasensitive resistance testing) of the acquired virus
- Study medications will be discontinued
- Supportive counseling and assistance with partner notification will be provided
- Seroconverter monthly study visits will be scheduled as listed above.

At the next visit,

- - Seroconverter interviews will begin
  - Safety labs (red top tube) and blood sample for measurement of resistance mutations by standard and ultrasensitive resistance testing (CPT tube) will be drawn 4 weeks off study med
  - Supportive counseling will be provided
  - Referral to the HIV care clinic will be made

At six months post HIV seroconversion,

- - - Blood sample (CPT tube) will be drawn for measurement of resistance mutations by standard and ultrasensitive resistance testing to look for reversion to wild-type virus.

HIV-infected participants will be provided all CD4 counts, HIV viral loads and antiretroviral resistance testing results. Participants will also be provided a letter explaining their participation in this clinical study and encouraged to show this letter to their HIV/AIDS care providers. Furthermore, participants will be encouraged to give the above results (especially antiretroviral resistance testing results) to their healthcare providers as these results will be critical for the management of the HIV-infected participant.

- - 1. **External Medical Records**

Written consent will be obtained (see Appendix A) and medical records from hospitals, clinics, or private medical offices (surgeries), as well as participant held health cards, will be reviewed and abstracted as indicated to:

- Obtain information for completing serious adverse event reports
- Obtain information to confirm whether a specific illness affects participant eligibility for continuation in the study
- Obtain information about pregnancy outcomes

1. **SUBSTUDIES**
   1. **Bone Density Substudy**

In Gaborone, the first 100 females and 100 males enrolled will undergo longitudinal measurement of bone density by DEXA scanning. DEXA scans are not available in Francistown, and there is no reason to believe that bone health will differ between participants in Gaborone and those in Francistown.

- - 1. **DEXA Scanning**

Measurements will be performed every six months with a QDR 4500C (Hologic Co, Bedford MA, USA) at the distal and ultradistal parts of the forearm, the lumbar spine and the hip. The distal forearm is defined as the area between: the point where the distance between the radius and ulnar bones measures 24 mm (the proximal end) and the site where they were 8 mm apart (the distal end). The utradistal forearm is defined as the 10 mm strip of the radius distal to the point that the radius and ulnar bones are 8 mm apart. The distal forearm is composed of 75% cortical bone and 25% trabecular bone, whereas the ultradistal part is 65% trabecular and 35% cortical bone[[31]](#endnote-32). BMD measures in the forearm are equally sensitive in both genders.[[32]](#endnote-33) However, more comparison data to other populations is available for the lumbosacral spine and hip and since BMD data for a young, healthy African population is not available, having all 3 standard measures will better define any BMD abnormalities identified.

Serial measurement of BMD (gm/cm^2^) will be used to assess changes in bone density over the course of participation in the study and this measure will be evaluated at each DSMB review and the Phase III interim analysis.

- - 1. **Responses to significant deficits in BMD**

Participants whose BMD readings at any visit are consistent with the WHO definitions for osteoporosis (T-score less than -2.5, more than 2 SD below that for “young adult” NHANES norms) will be provided with nutritional supplementation. The frequency of deficiencies in either vitamin D or calcium intake in young adults in Botswana is not known but in the TDF trial 11 of the first 28 persons scanned had DEXA scans consistent with osteopenia. Therefore in the TDF/FTC trial, vitamin D levels will be done on all 200 participants in the DEXA substudy at enrollment. Because it is safe, inexpensive, simple, and recommended to promote development of bone density in adolescents and young adults[[33]](#endnote-34), we will optimize vitamin D and calcium intake for those participants who develop BMD scores consistent with osteoporosis or vitamin D deficiency at enrollment. A combination tablet will be provided for once daily dosing, containing calcium citrate and cholecalciferol.

NIAID toxicity grading tables will be used to define AEs and SAEs for DEXA results.

- 1. **Pharmacokinetics/Pharmacodynamics (PK/PD) Studies**
     1. **Main Study (all trial participants)**

We propose to assay plasma and intracellular tenofovir and emtricitabine (Truvada®) using two blood collections in conjunction with the first scheduled 3 monthly study visit. The first blood collection will be in the 15 minutes prior to the participant taking their daily dose of study medication and the second collection approximately 2 hours (between 1 and 3 hours) after the dose is taken. These two blood collections are in order to estimate the relationship between blood plasma (pro-drug) and intracellular drug (active drug) levels using population pharmacokinetic methods. This information, in turn, will be used to describe the relationship between active drug exposure and prevention of HIV infection.

- - - 1. **Objectives**

1. Correlate steady-state plasma tenofovir and emtricitabine pharmacokinetics with intracellular tenofovir diphosphate (TDP) and emtricitabine triphosphate pharmacokinetics using population pharmacokinetic methods.

2. Correlation of tenofovir and emtricitabine HIV prevention efficacy with intracellular tenofovir and emtricitabine drug levels based upon population pharmacokinetic models.

- - - 1. **Rationale**

Understanding the relationship between tenofovir and emtricitabine drug levels over time (pharmacokinetics, PK) and the prophylactic efficacy of tenofovir in combination with emtricitabine (pharmacodynamics, PD) will inform optimization of prophylactic tenofovir/emtricitabine (Truvada®) dosing regimens in the future, based on relevant individual variables, to improve the efficacy of HIV prevention. The picture is complicated by the fact that tenofovir difumarate (TDF) is a prodrug, which is converted to tenofovir (TFV) in plasma in the process of absorption, which in turn is anabolized within cells to the antivirally active form, tenofovir diphosphate (TDP)^37^. Emtricitabine (FTC) is also a prodrug that is converted to the active moiety, emtricitabine triphosphate (E-TP), intracellularly. It is most likely the level of exposure to intracellular TDP and E-TP that best predicts the prophylactic antiviral effect. Accordingly, there is clearly a need to understand the intracellular- extracellular (IC-EC) relationships of both drugs in order to more completely understand the relationship of drug dose to prophylactic efficacy.

Demonstrating the relationship between plasma and intracellular drug concentrations, however, goes much further in that it allows one to build models that can predict intracellular drug levels and, therefore, the likelihood of efficacy, based solely on parent drug levels in the plasma.

To achieve our objectives in the context of the main TDF/FTC study in Botswana, we propose the additional assessment of plasma and intracellular tenofovir and emtricitabine levels at two points in time, 2 hours apart, following the same TDF/FTC dose in all seroconverters and a matched number of non-seroconverters in order to build a model of the relationship between plasma and intracellular drug levels and between these drug levels and seroconversion outcomes. If one builds a mixed effects model as we propose, giving consideration to relevant covariates that modify both plasma kinetics (potentially gender, race, renal and hepatic function) and intracellular kinetics (state of lymphocyte activation), then the model should have even greater predictive ability.

Because we desired to have intracellular drug levels on all seroconverters, but these individuals cannot be known at the beginning of the study, we plan to collect plasma and cells for assessment in all research subjects once steady-state is established (after >1 month on TDF/FTC). To best coincide with regularly scheduled study events, this collection will occur on the earliest possible three monthly visit which will be after steady-state is established and, hopefully, before seroconversion in most subjects. At study end, after unblinding, seroconverters and a similarly sized cohort of non-seroconverters who were assigned to the TDF/FTC study arm will have samples pulled from the repository for analysis. This will allow construction of the IC-EC PK model separately for seroconverters and non-seroconverters in order to identify important pharmacokinetic differences in these populations. Application of the PK data to samples around the time of seroconversion will allow estimation of intracellular levels and measurement of the plasma levels as correlates of HIV acquisition..

Finally, expression of a cellular activation marker, CD38, will also be assessed one time point at the time of the intracellular assays to allow inclusion as an explanatory covariate in the PK model. In vitro studies indicate that TDP half-life is shorter in cells activated with PHA and IL-2. There has been no clinical studies of TDP kinetics in terms of activation markers. Neither are there any published studies if the effect of cellular activation or specific activation markers and E-TP kinetics.

- - - 1. **Procedures**

We propose the following procedures be done at the first 3 monthly visit .

1. Three 10 mL CPT tubes and one 4 mL EDTA tube will be added to the current blood collection scheduled for that visit. These will be collected in the 15 minutes before the daily dose of study medication is taken and the time of blood drawing will be recorded. The CPT samples will be processed to isolate PBMCs from which cell lysates will be prepared from the cellular fraction. The remaining CPT sample will be processed to isolate PBMCs. Plasma from the EDTA tube will be assayed for TFV and FTC; cell lysates will be assayed for TDP and E-TP; PBMC will be assayed for CD38, a marker of PBMC stimulation.

2. Two 10 mL CPT tubes and one 4 ml EDTA tube will be collected between 1 and 3 hours (optimally at 2 hours) after the daily dose of study medication is taken.and the time of blood drawing will be recorded. The CPT samples will be processed to isolate PBMCs from which cell lysates will be prepared.. Plasma from the EDTA tube will be assayed for TFV and FTC; cell lysates will be assayed for TDP and E-TP.

3. The exact date and time (hour: minute) of each subject’s prior two daily doses of study medication will be recorded. The time of each dose should only be recorded if it is accurate within 15 minutes.

4. CD38 surface marker monoclonal antibody will be done (from PBMCs).

- - 1. **Substudy (separately consented participants)**

We propose to assay plasma and intracellular tenofovir and emtricitabine using 3 additional blood collections in conjunction with the scheduled 3 monthly study at which intracellular samples will be collected. This will provide 3 (plasma) and 2 (intracellular) additional observations in the intensive substudy over an 8 hour period with which to estimate plasma (pro-drug) and intracellular (active drug) pharmacokinetic (PK) parameters. This will be used to strengthen the parameter estimation for the population PK model being built with a larger set of subjects from the entire study. In turn, this information will be used to describe the relationship between active drug exposure and prevention of HIV infection.

- - - 1. **Substudy Objectives**

1. Correlate steady-state plasma tenofovir and emtricitabine pharmacokinetics with intracellular tenofovir diphosphate (TDP) and emtricitabine triphosphate pharmacokinetics using traditional and population pharmacokinetic methods.

2. Correlation of tenofovir and emtricitabine HIV prevention efficacy with intracellular tenofovir and emtricitabine drug levels based upon population pharmacokinetic models (also using sparse sampling plasma and intracellular data along with seroconversion events).

- - - 1. **Substudy Rationale**

To achieve our objectives in the context of the current study, we propose a substudy in 30 subjects in Botswana to assess plasma TFV and FTC levels at 3 additional time points and intracellular tenofovir-diphosphate and emtricitabine-triphosphate levels at 2 additional time points over an 8 hour period following dosing. These samples will provide adequate information to estimate traditional PK parameters at steady-state for plasma and more limited PK parameters for intracellular (active) drug levels. If one builds a mixed effects model as we propose, giving consideration to relevant covariates that modify both plasma kinetics (potentially gender, race, renal and hepatic function) and intracellular kinetics (state of lymphocyte activation), then the model should have even greater predictive ability.

To minimize the blood collected, these additional timed samples can be drawn during the same visit as the scheduled blood collections for the main PK/PD study.

- - - 1. **Substudy subjects**

Subjects enrolled in the Botswana TDF/FTC PrEP study will be eligible to participate in this intensive PK sampling substudy. There are no additional inclusion or exclusion criteria. The first thirty subjects who consent will be recruited at each site; 15 will be recruited from Gaborone and 15 from Francistown.

- - - 1. **Substudy Procedures**

The following procedures will occur at the first 3 monthly follow-up visit.

1. Subjects will fast (except for water) 8 hours prior to the morning PrEP study drug dosing. The fast should continue until 2 hours following the dose.

2. A pre-dose sample for plasma (4 mL EDTA) and intracellular (20 mL CPT) [tenofovir and emtricitabine]) drug levels will be collected within 15 minutes prior to the day’s dose. The exact time (hour:minute) of each blood drawing will be recorded.

3. The regularly scheduled TDF/FTC dose will be administered under observation and the exact time (hour: minute) will be recorded. The exact time of the prior two day’s doses (if it is accurate within 15 minutes) should also be recorded.

3. Plasma (4 mL EDTA) will be collected at 1, 2, 4, and 8 hours following TDF/FTC dosing. The exact time of each blood drawing will be recorded. These samples will be assayed for TFV and FTC.

4. Blood for cell lysate (two 10 mL CPT tubes) will be collected at 4 and 8 hours after TDF/FTC dosing. The exact time (hour:minute) of each blood draw will be recorded. The sample will be processed to separate plasma and cells. A cell lysate will be prepared from the cellular fraction and frozen for future assay. These cell lysates will be assayed for TDP and FTC-TP.

**Main PK/PD Study and Intensive Substudy Sampling Schedule**

|  |  |  | **Post-dose** | | | |
| --- | --- | --- | --- | --- | --- | --- |
|  | **Sample** | **Pre-dose*** | **1 hour** | **2 hours** | **4 hours** | **8 hours** |
| Intensive (n=30) | Plasma (4 mL EDTA) |  | X |  | X | X |
| Standard  (all) | Plasma (4 mL EDTA) | X |  | X |  |  |
|  |  |  |  |  |  |  |
| Intensive  (n=30) | Cells for lysate (20 mL CPT)* |  |  |  | X | X |
| Standard  (all) | Cells for lysate (30 mL CPT)* | X^∧^ |  | X |  |  |

*within 15 minutes prior to dose ^ 1 CPT tube will also be used for CD38 assay

- - - 1. **Substudy Risks/Benefits**

This substudy adds additional phlebotomy with its associated risk of pain, bruising, and infection. Since the visit will be scheduled to coincide with a regularly scheduled 3 month visit at which pre-dose and post-dose samples are already being collected for plasma and intracellular drug levels, then the increase in blood collected solely for the purposes of the intensive PK substudy is 44 mL. This represents a small and medically safe increase in the current protocol requirements. There are no direct benefits to the research subjects. However, there is the anticipation of generalizable knowledge to be gained in describing both (1) the relationship between plasma prodrug levels and intracellular active drug levels and (2) the concentration-response relationship between prodrugs, active drugs and prevention of HIV infection.

- - 1. **Data Analysis of both PK/PD studies (Main and Substudy)**
       1. **Unblinding**

It is critical to maintain blinding of trial investigators to the medication allocation status of participants. Therefore, all specimens and data collected for this substudy will be collected, stored, and shipped to Johns Hopkins using the blinded participant IDs.

In order to direct the testing of specimens to those from participants receiving TDF/FTC rather than placebo, after pharmacology substudy specimen shipments are received at Johns Hopkins, unblinding information will be provided directly to Dr. Craig Hendrix at Johns Hopkins by the CDC statistician, Dr. Roman Gvetadze. Specimens selected for shipment from Hopkins to Gilead for testing (intracellular assays) will be sent with blinded IDs only; the unblinding codes will not be shared with the Gilead lab. This will allow testing to proceed during the trial rather than waiting until the trial itself is completed and unblinded to be able to begin drug level testing.

Interim pharmacology substudy results (those reported before full unblinding of the trial) will only be shared with trial investigators at BOTUSA and CDC in aggregate forms that do not compromise the blinding of trial investigators.

- - - 1. **Traditional PK analysis.**

Plasma TFV and FTC levels will be used to calculate maximum concentration (Cmax), time to maximum concentration (Tmax), area under the concentration time curve (AUC_0-τ_), volume of distribution (Vd/F), and Clearance (CLf/F), and half-life (t-1/2). Descriptive statistics will be used to summarize these PK parameters for each site and for the substudy as a whole. Formal statistical comparison between sites and with historical controls will be performed.

Intracellular TFV-DP and FTC-TP levels will be used to estimate the minimum intracellular drug concentration (Cτ) and intracellular elimination rate constant. Given the sparse sampling design, other parameters may not provide accurate estimates, but will be explored.

These data will be used to build an intracellular-extracellular PK model. This model will contribute to parameter estimation for the sparse sampling PK strategy and PK-PD model building for the larger study.

- - - 1. **Integrated PK-PD Model Building**

**PK Model.** Based on our understanding about the dispositional pathway of TFV[[34]](#endnote-35), [[35]](#endnote-36) and FTC[[36]](#endnote-37), a two-compartment open linear PK model with an intracellular tenofovir disphosphate (TD) compartment will be tried. The model is summarized in Figure 2, where *C* and *V* represent concentration and volume in each compartment, respectively, indexed by the subscript (*C*, instead of *C_c_* was used for tenofovir concentration in the central compartment). *CL_f, TD_* is the ‘formation’ clearance of TD, and *CL_Other_* represents the clearance of tenofovir by other, mostly renal, pathways. Likewise, *CL_TD_* is the total clearance of TD, assumed to be mainly renal. *CL_IC_* is the intercompartmental (i.e., between the central and peripheral compartments) clearance of tenofovir, and *R_in_* is some function of oral absorption. The model building is the same for FTC and E-TP.

**Figure 2. The pharmacokinetic model of TFV**

We will try to first estimate the fraction of the enzymatic pathway and renal excretion as unchanged such that the sum of the relative elimination process is 1 (i.e., 100%). Practically, this will be implemented by assuming that the elimination of the metabolite in the terminal phase is formation rate-limited, i.e., the rate of metabolite excretion is equal to the rate of metabolite formation (=formation clearance * corresponding plasma tenofovir concentration). If this is not possible or metabolically implausible, the ratio of *CL_f,TD_* to *CL_Other_* will be fixed using *in vitro* data. Based on this analysis, the fraction of each clearance pathway will be derived and fixed in the PK model development.

However, given the sparse sampling nature of this population PK study, a bi-exponential disposition models of tenofovir and emtricitabine may not be estimated. In this case, a mono-exponential PK model will be fit instead.

Demographic data and other covariates will be tested for significance in reducing the interindividual variability for the parameters

**PD Model.** A binomial scale will be used to represent seroconversion with *0 = No seroconversion* and *1 = Yes seroconversion*. To estimate the probability of observing seroconversion, a logistic regression model will be used. Assuming *p* is the probability of observing seroconversion, this model has the following general structure:

$logit (p) = Ln \frac{p}{(1-p)} = f_{placebo}+f_{drug} +\eta$ (1)

and:

$p = \frac{\exp(f_{placebo}+f_{drug}+ \eta)}{1 + \exp(f_{placebo}+f_{drug} + \eta) }$ (2)

, where *f_placebo_* is the placebo effect model, *f_drug_* is the drug model, and *η* is subject-specific random effect in the logit domain. The placebo effect model can be any form or function of time. Inhibitory linear, inhibitory Emax, or inhibitory sigmoid Emax models will be tried for *f_drug_*. We will try to use PK model-predicted intracellular TDP concentration (i.e., as in Figure 3) as the exposure variable in linking with the probability of seroconversion. If this is not feasible, plasma concentrations of tenofovir will be used via hypothetical effect compartment model or indirect response model depending upon the availability of data. This will also be done for emtricitabine. In addition, in order to combine both drugs in the pharmacodynamic model, drug levels will be converted to drug/IC50 ratios for wild type HIV and the sum of this ratio (TDP/IC50 plus E-TP/IC50) will also be evaluated in the model.

**General model development strategy.** The final dataset will be created by pooling all PK and PD measurements, from which the population PK and PD parameters will be estimated. The PK parameters will be estimated first using NONMEM software (University of California, San Francisco, Version 5)[[37]](#endnote-38). Across the PK and PK model development, the first-order conditional estimation with interaction (FOCE-I) estimation method will be applied. Model development strategies will be consistent among the PK and the full PK-PD analyses. Basic structural models with inter-individual variability will be built. In addition, various covariance structures with different kinds of variability will be modeled and tested by applying the OMEGA BLOCK option in NONMEM.

**Figure 3**. **Logistic Probability Plot**. In the “Proposed” Study, each subject’s data would be plotted on the graph at a position corresponding to the subject’s intracellular trough (Cmin) concentration (other variables of drug exposure will also be assessed) and at a position along the Likelihood of Seroconversion (y) axis consistent with the subjects status as a seroconverter (top “1”) or non-seroconverter (bottom “0”). The shape of the probability curve (arrow) is determined by the cumulative proportion of subjects with a response at each position along the x-axis. Our hypothesis is that an inhibitory sigmoid response model (only imagined here) will best fit the observed data, i.e., as you increase the intracellular active drug concentration, the likelihood of seroconversion events decreases. Each drug will be tested separately in the model. Combination will be tested as described above using an HIV IC50 normalized transformation to provide a similar scale for each drug and then the drug/IC50 ratios will be summed at each time point to serve as the concentration variable.


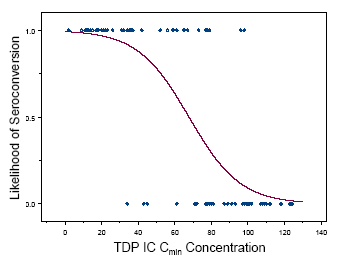


Probability Curve

- 1. **Seroconverter Studies**

The absolute number of seroconverters followed in this trial may not be large enough to define differences in the evolution of infection by TDF/FTC vs placebo use. However, several other oral antiretroviral prophylaxis studies will be following seroconverters in similar protocols and it is anticipated that combined analyses across the studies will have significant power to detect meaningful differences.

- 1. **Attitudes about the TDF/FTC Trial Substudy**
     1. **Objectives**

The overall goal of the sub-study is to enhance the likelihood the TDF/FTC trial is being implemented in an optimal manner; identify how study procedures might be improved; improve retention, if needed; and explore trial participant’s attitudes about issues surrounding implementation, should TDF/FTC prove effective and acceptable.

The sub-study has the following objectives: (1) explore participants’ experiences in the trial; (2) explore closely factors associated with sexual risk behavior change during the trial, (3) identify barriers and facilitators to recruitment, retention, and adherence to study drug; (4) investigate potential social harms and benefits associated with study participation; (5) assess participant attitudes about the acceptability of the trial drug as an HIV prevention intervention.

- - 1. **Methodology**

The study consists of a qualitative, in-depth interview administered to 24 trial participants approximately 3-6 months after they have enrolled in the trial, followed by 2 additional interviews on successive quarterly visits.

The qualitative method of in-depth individual interviews will be used. Individual interviews can be especially useful in eliciting “sensitive” information about topics such as sexuality and HIV/AIDS. The technique is used to elicit in-depth responses to a set of topics outlined in an interview guide[[38]](#endnote-39).

An ***individual interview guide*** has been developed and will form the basis of the three quarterly interviews (see Appendix L). Domains to be explored in these interviews include:

1. Participant motivation for trial participation
2. Participant experience with, and opinions of, key study procedures, e.g. recruitment/screening, study visits, counseling
3. Their behavior change and risk perception during trial
4. Their issues about retention in the study and adherence to study drug
5. The social context of their trial participation – perceptions/support of others, disclosure of participation, perceived community support, perceived roles of sponsors/government, opinions about possible intervention effectiveness at community level, and perceptions of research in relation to experience with other studies.
   - 1. **Study Population**

During the Phase II/III trial, a sample of 24 young adult participants (12 male and 12 female) will be interviewed.

- - 1. **Recruitment and Sampling**

The sub-study recruitment and sampling procedures include the following steps: 1) identify eligible participants; 2) recruit participants; 3) prepare for the interview; 4) conduct the interview; 5) manage the data. Subsequent interview rounds consist of steps 3 through 5.

The sampling strategy is designed to maximize selection of participants with a range of experiences within the trial. The selection process is thus based on two key criteria: (a) sexual behavior change; and, (b) adherence to study drug. The interviews will be evenly divided between the two trial sites, Gaborone and Francistown. The table below summarizes the characteristics of the final sample.

| **Quota** | **Criteria** | **Male** | **Female** | **Total** |
| --- | --- | --- | --- | --- |
| Sexual behavior | Increased risk | 3 | 3 | 6 |
|  | Decreased or no change in risk | 3 | 3 | 6 |
| Adherence | High adherence to study drug | 3 | 3 | 6 |
|  | Low adherence to study drug | 3 | 3 | 6 |
| **Total** |  | **12** | **12** | **24** |

Sampling for the sub-study will be quota-based, with the selection of participants coming when 60 individuals have reached their first quarterly visit, approximately at month 4 from the initiation of trial enrollment. Selection will be spread into 2 ‘stages’ over a 6 month period in order to ensure sufficient breadth of experience on the part of the participants and to provide information about study procedures in a time frame where indicated adjustments are most feasible. Approximately half of the sub-study participants will be enrolled during the first selection stage (stage I) and the remaining during the second selection stage (stage II), 6 months later. Isolated cases lost to follow-up will not be replaced. However, if loss to follow up exceeds 20% of the sample, replacement will be considered.

The sub-study PIs will review monthly data for adherence and sexual behavior to select participants with increased sexual risk-behavior and for evidence of difficulty with pill-taking or pill-sharing. Two lists will be compiled based on enrollment and monthly interview data. In list 1, participants will be ranked by increase in the number of sexual partners in the past 3 months and consistent condom use in the past 3 months. A random sample of individuals in the bottom third (riskiest) based on a weighted average of partner change and condom use. In list 2, participants will be ranked according to a weighted average of missed doses over the past 3 months and occurrences of pill-sharing in the past three months. Given the importance of capturing perspectives of those engaged in pill sharing, at least one participant per stage will be included in the sample if pill-sharing is reported. Selection will be taken from a random sample of those in the bottom third of list 2. The remaining sample will be selected randomly from the top two-thirds of each list. Those selected from List 1 will be removed from List 2 for selection.

Participation will be offered to the next person selected in each quota category until filled. Upon selection, the PI will notify the recruitment/retention officers to contact the selected participant and schedule the sub-study interview to coincide with the participant’s next monthly visit. Subsequent rounds of interviews will take place at regularly scheduled monthly visits at least 3 months apart.

- - 1. **Interview Procedures**

Just prior to the initiation of in-depth interviews, the data collection instrument will be piloted. Pilot participants will be recruited in Gaborone from study participants. Half of each pilot sample will receive interviews in English and the other in Setswana.

For initial piloting, 4 interviews will be conducted, with 2 males and 2 females. Pilot interviews are intended to identify any unrecognized problems with the questionnaires that need to be addressed before proceeding to the full study (e.g., wrong skip patterns, misunderstanding of the wording or intent of questions). If changes are trivial (e.g, correcting a skip pattern or grammar mistakes), the forms will be corrected without resubmission to the IRB. However, if changes are non-trivial (e.g, major rewording of questions, adding or deleting questions), an amendment will be submitted before proceeding to study data collection.

Interviews using the finalized interview guide will be conducted face-to-face by BOTUSA social/behavioral science staff. Each interview is expected to last between 60 to 90 minutes. The interview will be conducted either in English or Setswana at the sub-study participant’s choice and will be recorded using a digital audio recorder. In addition, the interviewer will take field notes to ensure non-verbal cues as well as ancillary observations are captured during the interview. Subsequent interview rounds will follow the same procedures but with appropriate adjustments to account for past interview responses and to incorporate any changes in the study experience since the prior interview.

- - 1. **Data Management**

Each interview will be given a unique ID number. Recordings will be transcribed in the original language. If in Setswana, the transcripts will then be translated into English for systematic textual analysis. When all transcripts and translations have been quality assured, the recordings will be erased, usually within 3 months.

Once transcribed, the data will be subjected to computer-assisted data management techniques to ensure the security and confidentiality of the data and to facilitate content analysis by senior researchers. A set of procedures will be used to password protect the digitally recorded files as well as to substitute sensitive information using a categorical substitution rule. Both recording medium and documentation will be kept in a locked file cabinet. Where records may contain confidential information from the interview participant, i.e. names of individuals, organizations, or locations, they will be systematically changed using phrase substitution. Common categorical substitution items include but are not limited to: sister, friend, partner/spouse, co-worker, doctor, study staff, clinic, hospital, non-governmental organization, print media, restaurant, neighborhood, educational facility.

- - 1. **Data coding and analysis**

Qualitative data analysis consists of identifying, coding, and categorizing patterns of meaning in textual data. One process used to reduce qualitative data to manageable units is thematic analysis[[39]](#endnote-40). How to analyze textual data varies by researcher, type of data, audience, and resources available. Regardless, the strategy must be rigorous, verifiable, and manageable given the resources available[[40]](#endnote-41).

As a first step, the transcribed interviews will be analyzed for identifiable themes and patterns. This will be compared to an a priori set of codes used in the development of the research objectives. Next the data will be sorted into classified patterns as either typical or contrasting units. A subsequent coding layer will be applied independently by two independent coders and then compared using reliability analysis. Conflicts between codes will be resolved between the coders and a final set of codes will be applied to the data. Finally, using the fully coded text, narratives will be developed to describe and assign meaning to the results found.

1. **STUDY PRODUCTS**
   1. **Study Products**

Two medications will be treated as study products, TDF/FTC and placebo.

Emtricitabine/tenofovir DF tablets are blue, capsule-shaped, film-coated tablets debossed with "GILEAD" on one side of the tablet. Each tablet contains 200 mg of emtricitabine and 300 mg of tenofovir DF. Each tablet contains the following inactive ingredients: microcrystalline cellulose, croscarmellose sodium, pregelatinized starch, lactose monohydrate, and magnesium stearate. The emtricitabine/tenofovir DF tablets are film-coated with FD&C Blue #2 aluminum lake, hypromellose, lactose monohydrate, titanium dioxide, and triacetin.

Placebo tablets to match emtricitabine/tenofovir DF are blue, capsule-shaped, film-coated tablets debossed with "GILEAD" on one side of the tablet. Each tablet contains the following inactive ingredients: microcrystalline cellulose, croscarmellose sodium, pregelatinized starch, lactose monohydrate, and magnesium stearate. The placebo combination tablets are film-coated with FD&C Blue #2 aluminum lake, hypromellose, lactose monohydrate, titanium dioxide, and triacetin.

Each bottle of study product will be labeled with a randomization number, the protocol number, expiration date, and the sponsor address.

Of note, the Botswana national guidelines for ARV therapy have been recently revised and will recommend TDF/FTC for first-line treatment of HIV.

- - 1. **TDF/FTC** (See also investigator’s brochure, Appendix E)
       1. **Chemical and pharmacokinetic properties**

**Emtricitabine (FTC):** The chemical name of emtricitabine is 5-fluoro-1-(2R,5S)-[2-(hydroxymethyl)-1,3-oxathiolan-5-yl]cytosine. Emtricitabine is the (-) enantiomer of a thio analog of cytidine, which differs from other cytidine analogs in that it has a fluorine in the 5-position.

**Tenofovir disoproxil fumarate (TDF):** Tenofovir disoproxil fumarate is a fumaric acid salt of the bis-isopropoxycarbonyloxymethyl ester derivative of tenofovir. The chemical name of tenofovir disoproxil fumarate is 9-[(R)-2 [[bis[[(isopropoxycarbonyl)oxy]- methoxy]phosphinyl]methoxy]propyl]adenine fumarate (1:1).

- - - 1. **Mechanisms of Action**

**Emtricitabine:** Emtricitabine, a synthetic nucleoside analog of cytidine, is phosphorylated by cellular enzymes to form emtricitabine 5'-triphosphate. Emtricitabine 5'-triphosphate inhibits the activity of the HIV-1 reverse transcriptase (RT) by competing with the natural substrate deoxycytidine 5'-triphosphate and by being incorporated into nascent viral DNA which results in chain termination. Emtricitabine 5′-triphosphate is a weak inhibitor of mammalian DNA polymerase α, β, ε and mitochondrial DNA

polymerase γ.

**Tenofovir disoproxil fumarate:** Tenofovir disoproxil fumarate is an acyclic nucleoside phosphonate diester analog of adenosine monophosphate. Tenofovir disoproxil fumarate requires initial diester hydrolysis for conversion to tenofovir and subsequent phosphorylations by cellular enzymes to form tenofovir diphosphate. Tenofovir diphosphate inhibits the activity of HIV-1 RT by competing with the natural substrate deoxyadenosine 5′-triphosphate and, after incorporation into DNA, by DNA chain termination. Tenofovir diphosphate is a weak inhibitor of mammalian DNA polymerases α, β, and mitochondrial DNA polymerase γ.

- - - 1. **Antiviral Activity**

**Emtricitabine and tenofovir disoproxil fumarat**e: In combination studies evaluating the in vitro antiviral activity of emtricitabine and tenofovir together, synergistic antiviral effects were observed.

**Emtricitabine:** The in vitro antiviral activity of emtricitabine against laboratory and clinical isolates of HIV was assessed in lymphoblastoid cell lines, the MAGI-CCR5 cell line, and peripheral blood mononuclear cells. The IC50 values for emtricitabine were in the range of 0.0013−0.64 µM (0.0003−0.158 µg/mL). In drug combination studies of emtricitabine with nucleoside reverse transcriptase inhibitors (abacavir, lamivudine, stavudine, zalcitabine, zidovudine), non-nucleoside reverse transcriptase inhibitors

(delavirdine, efavirenz, nevirapine), and protease inhibitors (amprenavir, nelfinavir, ritonavir, saquinavir), additive to synergistic effects were observed. Most of these drug combinations have not been studied in humans. Emtricitabine displayed antiviral activity in vitro against HIV-1 clades A, B, C, D, E, F, and G (IC50 values ranged from 0.007−0.075 µM) and showed strain specific activity against HIV-2 (IC50 values ranged from 0.007−1.5 µM).

**Tenofovir disoproxil fumarate:** The in vitro antiviral activity of tenofovir against laboratory and clinical isolates of HIV-1 was assessed in lymphoblastoid cell lines, primary monocyte/macrophage cells and peripheral blood lymphocytes. The IC50 (50% inhibitory concentration) values for tenofovir were in the range of 0.04−8.5 µM. In drug combination studies of tenofovir with nucleoside reverse transcriptase inhibitors (abacavir, didanosine, lamivudine, stavudine, zalcitabine, zidovudine), non-nucleoside

reverse transcriptase inhibitors (delavirdine, efavirenz, nevirapine), and protease inhibitors (amprenavir, indinavir, nelfinavir, ritonavir, saquinavir), additive to synergistic effects were observed. Tenofovir displayed antiviral activity in vitro against HIV-1 clades A, B, C, D, E, F, G and O (IC50 values ranged from 0.5−2.2 µM). The IC50 values of tenofovir against HIV-2 ranged from 1.6 µM to 4.9 µM.

- - - 1. **Pharmacokinetics in Adults**

**Emtricitabine:** Following oral administration, emtricitabine is rapidly absorbed with peak plasma concentrations occurring at 1–2 hours post-dose.. Emtricitabine is eliminated by a combination of glomerular filtration and active tubular secretion. Following a single oral dose of emtricitabine, the plasma emtricitabine half-life is approximately 10 hours.

**Tenofovir disoproxil fumarate:** Following oral administration, maximum tenofovir serum concentrations are achieved in 1.0 ± 0.4 hour. Tenofovir is eliminated by a combination of glomerular filtration and active tubular secretion. Following a single oral dose of TDF, the terminal elimination half-life of tenofovir is

approximately 17 hours.

- - - 1. **Safety Profile in HIV Treatment Trials**

TDF and FTC have almost exclusively been studied in HIV-infected adults, either as monotherapy for brief periods to assess safety or pharmacokinetics, or more commonly, as a component of multi-drug combination therapy for treatment of HIV disease. In these situations, TDF and FTC each have excellent safety profiles, as does the combination, when used in multidrug trials (See Appendix E, Investigator’s Brochure).

Emtricitabine: In clinical treatment trials to date, all adverse events were reported with similar frequency in emtricitabine and control treatment groups with the exception of skin discoloration which was reported with higher frequency in the emtricitabine treated group. Skin discoloration, manifested by freckle-like hyperpigmentation on the palms and/or soles was generally mild and asymptomatic. The mechanism and clinical significance are unknown.

Grade 3/4 elevations of ALT and AST (>5 x ULN), bilirubin (>2.5 x ULN), creatine kinase (>4 x ULN), decreased neutrophils (<750/mm3), pancreatic amylase (>2.0 x ULN), serum amylase (>2 x ULN), serum glucose (<40 or >250 mg/dL), serum lipase (>2.0 x ULN) and triglycerides (>750 mg/dL) have been reported to occur in 1–12% of patients receiving emtricitabine.

Tenofovir Disoproxil Fumarate: In a placebo-controlled clinical treatment trial (907), all adverse events were reported with similar frequency in TDF and placebo treatment groups

**Study 907 (Phase III, double-blind RCT, TDF/placebo added to stable regimen)**

**% of patients who experienced grade 3 or 4 events (occurring in >2 patients) regardless of relationship to study drug**

| **Type of Event** | **Placebo**  **(N=182)**  **(Week 0-24)** | **TDF**  **(N=368)**  **(Week 0-24)** |
| --- | --- | --- |
| **Clinical Adverse Event**  **Grade 3 or 4*** | | |
| Asthenia | <1 % | <1 % |
| Pain | <1 % | <1 % |
| Myalgia | 0 | <1 % |
| Abdominal pain | <1 % | <1 % |
| Nausea | 1 % | <1 % |
| Diarrhea | 2 % | <1 % |
| Pancreatitis | 0 | <1 % |
| Bacterial Infection | <1 % | <1 % |
| Cellulitis | <1 % | <1 % |
| Pneumonia | 0 | <1 % |
| Sinusitis | 1 % | <1 % |
| Renal Calculus | 0 | <1 % |
| Anxiety | 0 | <1 % |
| Depression | <1 % | <1 % |
| *Events causing permanent study drug discontinuation* | 2 % | 3 % |
| **Laboratory Abnormalities**  **Grade 3 or 4*** | | |
| Triglycerides | 13 % | 8 % |
| Creatine Kinase | 14 % | 7 % |
| Amylase | 7 % | 6 % |
| Urine Glucose | 3 % | 3 % |
| Serum Glucose | 4 % | 2 % |
| AST | 3 % | 3 % |
| ALT | 2 % | 2 % |
| Neutrophils | 1 % | <1 % |
| *Events causing permanent study drug discontinuation* | 2 % | 0 |

***see Appendix F for severity grading**

In Study 907 during the placebo controlled phase, there were no grade 3 or 4 elevations in creatinine and there were two incidences of grade 3 or 4 hypophosphatemia, 1 in each arm.

It is reasonable to hypothesize that side effects and toxicities will be less in young, HIV-uninfected, healthy persons, and if true, and the current safety data in HIV-infected persons is an upper-bound of what can be expected in this trial, the data are reassuring.

- - - 1. **Safety Data from TDF Prophylaxis Trials**

TDF/FTC has only one additional side effect when compared to TDF alone, the freckle-like hyperpigmentation which occurs in <1% of persons and is asymptomatic.

Hyperpigmented lesion in a South African child receiving FTC


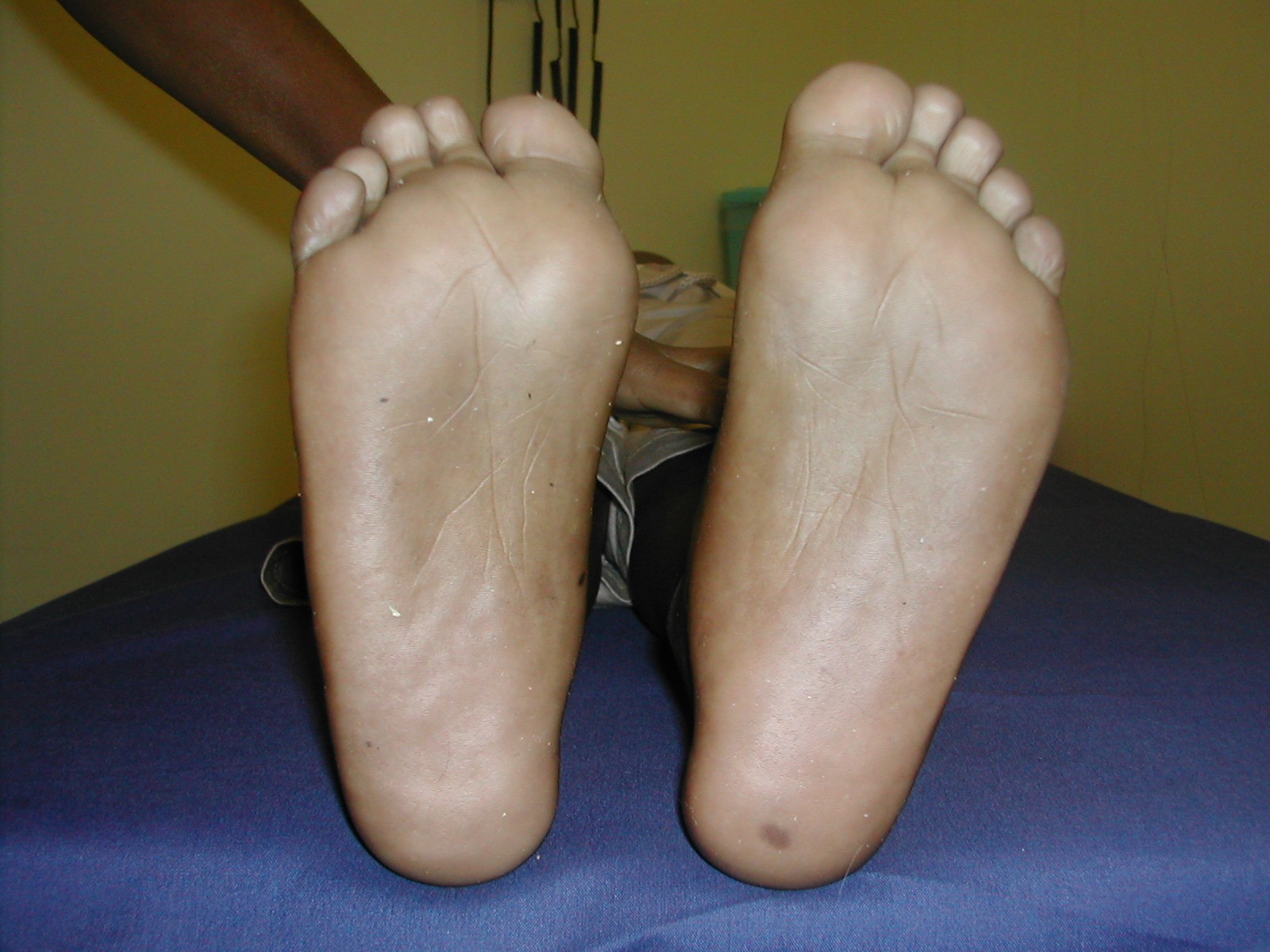


⇦

Safety data in HIV-negative persons in TDF oral prophylaxis trials is becoming available from trials conducted in Botswana, Thailand, and US by CDC; in Nigeria, Cameroon, and Ghana by Family Health International.

Preliminary safety data from these trials have been confidentially provided separately from the protocol for sharing with the HRDC and CDC IRB.

No serious safety problems have occurred in any of the trials.

- - 1. **Study Product Accountability**

The study sites will periodically receive a supply of study products, labeled with the randomization code (the Participant ID), sufficient for each study participant over the duration of the trial. At each study clinic, study product will be stored in a limited access area that is securely locked and temperature controlled at approximately 25° C.

Strict inventory will be maintained of all study product received. This will include recording the dates and quantities of study product dispensed to and returned by study participants, by participant ID, and randomization code.

Study medications will be dispensed by study clinicians. No product will be dispensed by persons not on the study staff and none will be dispensed to persons not enrolled in the trials.

Study product that has expired or returned unused by participants will be destroyed as clinical waste in Botswana by incineration or burial.

- - 1. **Study Product Dispensing**

At each relevant study visit, participants will be dispensed a bottle containing 30 pills. If a visit is missed, participants will be called to return to the clinic to pick up additional medication. If unused pills remain at a follow-up visit, these will be counted and redispensed along with a new bottle and participants will be instructed to finish the partial bottle first, then start the new bottle, and bring both bottles back to the next visit.

Participants will be instructed to take TDF/FTC as follows:

- Take one tablet each day
- Swallow the tablet whole; do not chew or dissolved it in the mouth
- Try to take it around the same time every day to help keep a constant amount in the blood stream.
- If a dose is forgotten at the usual time, take it as soon as remembered on that day.
- Do not take multiple doses if one or more days are missed.
- Keep medication in the container in which it is supplied (to ensure stability)
- Never give doses of your medication to others
- Inform any clinic or provider about your study participation before accepting prescriptions for other medications.
- Bring their pill bottle to each study visit (even if empty).

Participants will be provided with small cards with contact information for the study physician should they need medical care outside the study.

- - 1. **Product Adherence**

Every reasonable effort will be made to reinforce and monitor adherence to the prescribed study products, TDF/FTC and placebo.

- - 1. **Adherence Counseling**

Adherence counseling will be based on existing interventions used in Botswana to increase adherence to antiretrovirals, and interventions to increase medication adherence among HIV-infected youth in the U.S.[[41]](#endnote-42),[[42]](#endnote-43),[[43]](#endnote-44),[[44]](#endnote-45)  It has been adapted for use by HIV-negative Batswana young adults enrolled in the TDF/FTC trial. The counseling session will be individualized will include the following:

- Basic education about study medications (dose & schedule, how TDF/FTC may work, identifying side effects & how to handle them, refills & storage, do’s and don’ts of taking study medication, etc.)
- Discussion of the participant’s beliefs and attitudes about taking TDF/FTC/Placebo (concerns, expectations, fears)
- Encouragement about talking to study personnel about medications (e.g., what to tell study personnel about your medications; questions you might have and should feel free to ask; the need to be open, even when adherence is poor, so study procedures & adherence counseling can be improved)
- Identifying and solving barriers to adherence (e.g., scheduling and organizational skills, reminder devices, social support, disclosure).
- Discussion about reasons not to engage in pill-sharing and how to handle requests from friends and family to borrow doses.

At each follow-up visit, study counselors will review the study participant’s self-report of adherence and medication diaries (described below), discuss barriers to adhering to the daily dose of study medication, and help identify strategies for overcoming barriers. Rigorous quality assurance procedures will be followed as detailed in the Adherence Counseling SOP.

- - 1. **Adherence Monitoring**

We will best be able to assess safety and efficacy of TDF/FTC in light of knowledge about medication exposure, so monitoring adherence to TDF/FTC or placebo as prescribed is necessary. Since direct observation of pill-taking is not feasible and self-report is subject to several potential biases, four methods will be used to assess this key factor. Assessing medication adherence will be by:

- self-report on interviews

to encourage reporting of nonadherence, participants will be reminded that we understand taking all doses on time is difficult and that we need them to tell us about times when doses were missed or taken late. Interview data on adherence will be collected both face-to-face (monthly) and by ACASI (every six months).

- pill counts at each study visit

participants will be asked to bring all untaken pills back to the study clinic at each visit. They will be counted and redispensed with a new 30-day supply to the participant for the next study visit interval.

- daily diaries

at each study visit, participants will be given diary sheets and instructed to make an entry each day indicating if and when they took study medication; completed diaries will be collected at the next study visit.

- blood levels of TDF/FTC done on unblinded specimens at the conclusion of Phase III (after blinding has been broken), including:
  - from all HIV seroconverters at the end of the Phase III trial

plasma reposited from the visit at which seroconversion was detected. Results will be compared to diaries and pill counts to assess adequacy of self-report to predict therapeutic blood levels around the time of seroconversion.

- - from 20% of all persons who received TDF/FTC and did not seroconvert in Phase III trial

for each person randomly selected, plasma stored from the quarterly visit at which drug level specimens are collected (see section 6.5) be tested for drug levels. Results will be compared to diaries and pill counts to assess correspondence to self-reported adherence.

1. **CONCOMMITANT INTERVENTIONS**

In addition to the intervention under study (TDF/FTC use), we will provide other “standard-of-care” interventions to reduce the risks associated with sexual activity.

- - 1. **HIV Risk Reduction Counseling**

Voluntary counseling and testing procedures in the community or at medical facilities are geared to a single visit. In these trials, we need protocols that make good use of the multiple counseling sessions participants have over time. Therefore, we will structure our counseling sessions based on the Project Respect counseling intervention materials developed and tested by CDC[[45]](#endnote-46) The Rapid Test (one session) protocol will be used for all screening visits and a multisession protocol for the follow-up visits with participants who enroll in the trial. The intent of these protocols is to explore with participants specific behaviors they may be engaging in that increase their risk of HIV infection, discuss with them options for reducing each of those risks, help them develop a concrete plan to take small steps for reducing one or more of the identified risks, using role-plays and other techniques to assist in identifying barriers that might stand in the way and potential ways around them, and evaluate the implementation of their personalized plan and its outcomes at the next visit. This is an iterative process in which participants are encouraged toward continued reductions in risk behaviors. The risk reduction counseling protocol will be tailored to the Botswana context and to the developmental issues of young adults. Risk reduction options which will be promoted include: periodic abstinence (i.e., increasing gaps between sexual partnerships), mutual monogamy (i.e., one partner at a time and avoid partnership with person who have multiple partners), couple HIV testing (e.g. determine serostatus of each new partner), non-penetrative sexual activities, and condom use. In addition to the individualized risk reduction counseling at each quarterly visit for those enrolled in the trials, counseling will be provided at monthly visits when requested by participants. Rigorous quality assurance procedures will be followed as detailed in the Risk Reduction Counseling SOP.

Because of the young age of the study population and the high prevalence of HIV, although they must already be sexually active to be enrolled in the trial, we will counsel them to reduce their number of sexual partners, including the promotion of secondary abstinence (taking a hiatus from sexual activity). For those young adults who insist on continuing their sexual activities, we will counsel them on other methods to reduce their risk of pregnancy and acquiring HIV or other STIs.

- - 1. **STI Treatment**

All participants will be asked about symptoms of STIs and undergo a physical examination at enrollment and every six months in phase III. Based on examination findings and stat labs (see Appendix G), participants will be treated. Treatment will usually be based on laboratory test results. Immediate treatment will be based on syndromic diagnoses (without a confirmed etiology) only for urethral discharge (men), suspected PID (women), genital ulcers, or genital warts. Definitive laboratory test results will be reviewed by the study physicians and any person not adequately treated at the study visit will be recalled for treatment. Whenever possible, single dose, directly observed medications will be given. Partner notification will be done as per MOH guidelines.

- - 1. **Contraception**

In a recent community survey, among young women ages 15-19 yo, 75% were not using any contraception. Of the 25% contracepting, oral contraceptives (6.5%), condoms (9.4%), abstinence (4.8%) and injectables (3.4%) constituted nearly all of the use.[[46]](#endnote-47) Among women attending the family planning clinics in 2002, nearly ¾ opted for condoms (71%), with oral contraceptives (16%) and injectables (11%) the next most common options chosen.[[47]](#endnote-48)

Condom use will be promoted for all participants (male and female) for their protection against HIV and STI infections as well as pregnancy when they choose to be sexually active. Free male and/or female condoms will be provided at each study visit.

However in the TDF trial (and several recent vaginal microbicide trials in the region), the pregnancy rate was unacceptably high among women attempting consistent condom use as their sole means of contraception. Therefore, willingness to use effective contraception (including hormonal contraception or an IUD) will be required for female participants in this trial. Women will be offered oral or injectable contraception methods currently supplied in the MOH family planning clinics, both those already using them, and those wishing to initiate their use. Women who decide not to continue using hormonal contraception in the first 6 months of the study, will be discharged from the trial.

- 1. **Concomitant Medications**

Enrolled study participants may continue use of all concomitant non-prescription medications and any medications prescribed after enrollment into the trial.

All concomitant medications will be reported on applicable study case report forms. In addition to prescribed and over-the-counter medication, vitamins, herbal remedies, and other ingested or inhaled traditional preparations will be recorded. Medications used for the treatment of AEs that occur during study participation also will be recorded on applicable study case report forms.

1. **CLINICAL SAFETY EVENT MANAGEMENT AND REPORTING****[[48]](#endnote-49)**
   1. **Definitions**

For the purposes of documentation and reporting of events related to the safety of participants receiving study medications in these trials, the ICH definitions and terminology will be used.

- - 1. **Adverse Event (AE)**

An AE is any unfavorable and unintended sign (e.g., an abnormal laboratory finding), symptom, or disease temporally associated with the use of an investigational product, whether or not considered related to the product.

An AE **does not** include:

- Diseases or conditions present or detected prior to taking study drug (i.e., pre-existing) which do not worsen during the trial.
- Medical or surgical procedures (e.g., endoscopy, appendectomy); but the condition that leads to the procedure is an AE (bloody diarrhea, appendicitis)
- Situations where an untoward medical event has not occurred (e.g., hospitalization for elective procedure or for social reasons/convenience)
- Overdose of either study drug or concomitant medication without any signs, symptoms, or hospitalization.

.

- - 1. **Adverse Drug Reaction (ADR)**

An ADR is an AE where a causal relationship between the study medication (taken in doses normally used) and the adverse event is at least a reasonable possibility.

- - 1. **Unexpected Adverse Drug Reaction (UADR)**

An unexpected ADR is an UADR, the nature or severity of which is not consistent with the applicable product information (e.g., Investigator’s Brochure for an unapproved investigational medicinal product).

- - 1. **Serious Adverse Event (SAE)**

An SAE is any AE that, if suspected to be related to use of the study medication (ADR), might lead to changes in the way the medication is used (e.g., change in dose, population, required monitoring, consent forms). An SAE is any untoward medical occurrence that at any dose of study medication may have resulted in:

- Death
- An immediate risk of death (i.e., “life threatening”)
- Formal admission to the hospital as an inpatient or prolongation of an existing hospitalization
- A persistent or significant disability or incapacity
- A congenital anomaly or birth defect
- A medical event that, based on clinical judgment, may jeopardize the patient and may require intervention to prevent one of the outcomes listed above

All Grade 4 severity events listed on the DAIDS Table for Grading Severity of Adult Adverse Experiences for Vaccine & Prevention Research Program will be considered SAEs. Grade 3 severity events will be evaluated on a case-by-case basis and may be reported as SAEs if warranted in the clinical judgment of the evaluating physician in consultation with the IOR. However, signs and symptoms will only be reported themselves as SAEs when a diagnosis of the event cannot be established and itself reported as an SAE.

- 1. **Evaluation of Adverse Events**

For every possible AE identified by interview, examination, or laboratory result, the study clinicians will evaluate and document on the appropriate study forms the following information:

- **Nature**

Description of the event or condition

- **Severity**

Using the DAIDS Table for Grading Severity of Adult Adverse Experiences for Vaccine & Prevention Research Programs (see Appendix F)

- **Expectedness**

In light of information in the Investigator’s Brochure (see Appendix E)

- **Relationship to Study Product**

Using good clinical judgment and the following criteria:

- **Unclassifiable**

There is insufficient information about the AE to allow for an assessment of causality

- **Unrelated**

Onset of the AE had no reasonable temporal relationship to administration of the study product

OR a causal relationship is biologically implausible

OR the event is attributed to an alternative etiology

I.e., are considered clearly & incontrovertibly due to causes other than the interaction/intervention

- **Unlikely**

Those AEs that, after careful medical consideration at the time they are evaluated, are considered to be more likely explained by another cause than by administration of the study product*.*

- **Possibly related**

Onset of the AE has a reasonable temporal relationship to study product administration

AND a causal relationship is biologically plausible

I.e., are considered to be unlikely to be related but cannot be ruled out with certainty

- **Probably related**

Onset of the AE has a strong temporal relationship to administration of the study product that cannot be explained by the participant’s clinical state or other factors

AND a causal relationship is biologically plausible

I.e., are believed with a high degree of certainty to be related to the interaction/intervention

- **Definitely related**

Onset of the AE shows a distinct temporal relationship to administration of the study product that cannot be explained by the participant’s clinical state or other factors

OR the AE occurs on re-challenge

OR the AE is a known reaction to the product or chemical group or can be predicted by the product’s pharmacology.

I.e., are believed to be incontrovertibly related to the interaction/intervention

- **Seriousness**

Determination of whether it meets the definition of an SAE (listed above)

- **Requirements for clinical management**

Determine whether to suspend use of study medication, temporarily or

permanently

Conduct any indicated diagnostic or confirmatory testing or examination

Administer or refer for any indicated treatment

Determination of need for unblinding to deliver effective care

- 1. **Reporting of Safety Events**
     1. **Expedited Reporting**

All SAEs whether related or not will be reported expeditiously. Within 72 hours of learning of the SAE investigators will fax (or e-mail) a preliminary report and within 10 days will fax (or e-mail) a complete report (confirming receipt by e-mail or phone) to all of the following:

Human Subjects Contact Ms. Shenaaz El-Halabi

Nat’l Center for HIV, Hepatitis, STD, and TB Prevention Human Subjects Coordinator

U.S. Centers for Disease Control and Prevention Health Research and

Fax: +1.404-639.8606 Development Committee

Phone: +1.404.639.8000 Botswana Ministry of Health

E-mail: [nchstphs@cdc.gov](mailto:nchstphs@cdc.gov) Fax: +267.

Phone: +267.7156.2255

E-mail: sel-halabi@gov.bw

NCHSTP/DHAP Human Subjects

E-mail: [dhsubjects@cdc.gov](mailto:dhsubjects@cdc.gov)

Dr. Chris Elias Gilead Global Drug Safety

Program for Appropriate Technology in Health Gilead Sciences

Chairperson, DSMB Fax: 650-522-5477

Fax: 206-285-6619 Phone: 650-522-5894

Phone: 206-285-3500 E-mail [safety_fc@gilead.com](mailto:safety_fc@gilead.com):

E-mail: celias@path.org

The NCHHSTP human subjects contact will ensure that the CDC IRB is notified promptly for all SAEs which may be related to study drug use.

Expedited reporting of all SAEs will be sent to Gilead, regardless of relationship to drug, with the initial report sent within 24 hours of learning of the SAE. This is at the specific request of Gilead Sciences.

- - 1. **Routine Periodic Reporting**

Only routine reporting will be done for all ADRs, defined as AEs which may be related to study drug use (i.e., possibly, probably, or definitively related by above listed definitions) and that do not meet the criteria for SAE.

Routine reporting will consist of periodically providing a line listing of the events by type (e.g., lab toxicity or clinical event), body site, and study site. These listings will be provided to the Gilead Sciences, CDC Atlanta, and IRBs/ECs for each annual continuation review; for each DSMB review; and on request from any regulatory agency involved in these trials.

- - 1. **Documented on Clinical Forms but Not Otherwise Reported**

All AEs will be documented on the appropriate clinical study forms. Those which are judged “unrelated” to study medication or “unclassifiable” (using the above listed criteria) will not be reported routinely but the data will be available in the database should reporting of specific conditions be requested in future. Examples of such conditions are: accidental injuries (e.g., resulting from motor vehicle accidents or fires), diagnosed infectious diseases (e.g., malaria, urinary tract infection, STIs), progression of pre-existing conditions (e.g., cervical dysplasia).

.

**Yes**

**No**

**Definitely**

**Probably**

**Possibly**

**Unrelated**

**Unlikely**

**Unclassifiable**

**Routine Periodic Reporting**

Complete AE Report

Line listing with annual IRB/EC reports

Line listing for DSMB reviews

**Documented but not Reported**

- Complete AE Report

- Not included in line listings

**Expedited Reporting**

Complete SAE/AE Report

Send initial report within 72 hours

Send complete report within 10 days

**Relationship to TDF?**

temporal

alternate etiology

known reaction

**SAE?**

- 1. **Clinical Management of Adverse Events**

Adverse clinical events will be evaluated and managed at presentation by study physicians by either providing initial treatment or by referral or arranged transport for treatment at the nearest clinic or hospital.

A decision will be made about the continuation of study product based on the following algorithm (See Appendix H)

- - 1. **Laboratory Abnormality or Clinical Event *(except serum creatinine elevation)***

**Grade 1 or 2** Continue study medication at the discretion of the study physician.

**Grade 3 or 4** May continue study medication for up to 3 days while values are confirmed

**Confirmed Grade 3** If unrelated or unlikely related to study drug by above definitions, may

continue dosing

If causal relationship to study drug possible by above definitions

(e.g. possibly, probably, or definitely related),

withhold drug until toxicity returns to < grade 3

then retest to confirm HIV negative

then restart study drug

if toxicity ≥ grade 3 recurs, permanently discontinue dosing

follow as frequently as clinically indicated (up to study closure)

until toxicity resolves

**Confirmed Grade 4** If unrelated or unlikely related to study drug by above definitions, may

continue dosing

If causal relationship to study drug possible by above definitions,

Permanently discontinue study drug

Follow as frequently as clinically indicated (up to study closure) until toxicity resolves

Do not rechallenge

- - 1. **Serum Creatinine Elevation**

The baseline serum creatinine value is defined as the last value obtained from the laboratory prior to the administration of the first dose of study drugs. (See Appendix I)

**All Grades** Continue study medication for up to 3 days while values are confirmed

**Confirmed Grade 1** Continue study medication at the discretion of the study physician

Monitor weekly until return to within 0.3 mg/dL of the baseline

value

**Confirmed Grade 2-4** Permanently discontinue study drug

Monitor as clinically indicated (generally at least monthly up to 3 months) until return to within 0.3 mg/dL of the baseline value

1. **SOCIAL TRIAL RISKS**

Particularly in HIV prevention trials, there are sometimes non-clinical harms that can occur to trial participants. These include unintended breaches in confidentiality, social stigma that may result from a participants disclosure of study participation, and domestic/family/partner disagreements (including violence) resulting from study participation. In these trials, it will be known in the wider community that participants are sexually active and may be taking an antiretroviral medication; there is the possibility of confusion about whether participants are in-fact HIV positive. Since the trials are randomized and blinded, these social risks will not be related to study drug per se but rather to trial participation itself. And since we do not have a comparison standard against which to evaluate the occurrence of some of these possible events, it will be difficult to determine when they are a result of study participation as opposed to ongoing social situations. For those reasons, we will not generally treat reports of these events as adverse events unless they meet clinical AE criteria.

However, any breach in the confidentiality of a study participant by study staff or events (e.g., theft of files) will be reported as an SAE. Staff will be required to sign confidentiality agreements and any intentional breach by a staff member will be grounds for immediate dismissal.

In addition, there may be community harms if misinformation circulates that encourages misuse of antiretroviral medications dispensed to HIV infected person in the community. We will educate all study participants about the dangers in giving doses of their study medication to others (e.g., PrEP may not work, they may have placebo, they have been screened for contraindications, and they are monitored for safety while others would not be). In addition, we will inform HIV care clinicians about the study and provide them with educational materials for counseling HIV infected persons about why they should not give doses of their medications to others, particularly those not known to be HIV-infected. Through community advisory boards and monitoring of reports from the community, we will attend consistently to the understanding of the study and PrEP, identify areas of misunderstanding and take action to clarify confusions and education the community through public meetings, use of public media, and contacts with key community informants.

1. **STATISTICAL SUMMARY**

The primary aim of the study is to provide effective prophylactic levels of TDF/FTC to interrupt sexual HIV transmission. This study is also designed to evaluate its safety in HIV-uninfected young adults in Botswana.

- 1. **Study endpoints and assessment**
     1. **Safety endpoints**

The primary endpoints for safety are:

- Adverse events (AE), including adverse drug reaction (ADR), unexpected adverse drug reaction (UADR), and serious adverse events (SAE).
- Grade 3 or 4 renal or hepatic function laboratory toxicities or clinical toxicities, as defined by Gilead-modified NIAID Adult Common Toxicity Tables, and which cannot be directly attributed to a cause other than study medications.
- Significant changes in bone mineral density

AEs and laboratory and clinical toxicities will be identified by interview, examination, or laboratory results at scheduled visits and will be closely monitored during the trial.

Bone mineral density will be measured using DEXA scanning at baseline and every six months on a subset of study participants.

The secondary safety endpoints include behavioral safety, measured as frequency of the reported risky sexual behavior, adherence to prescribed treatment regimen, measured as proportion of days the study medicine was taken correctly, and development of antiretroviral resistance, measured as incidence of resistance mutations in HIV among the seroconverted subjects.

- - 1. **Efficacy endpoint**

The primary efficacy endpoint is HIV-1 infection status over time. HIV-1 infection status will be identified at monthly visits. The time from randomization to the date of HIV infection will be recorded.

The following procedures will be used to estimate the date of HIV-1 infection:

- If a subject becomes EIA-positive at the *i*-th visit, a PCR test will be performed sequentially on previous plasma samples, starting from the most recent sample, until the earliest PCR-positive plasma sample is identified. The date of the first PCR-positive sample will be considered the date of HIV infection;
- If a subject becomes EIA-positive at the i-th visit, but the (i-1)-th plasma test is PCR-negative, then the date of HIV infection is estimated as the midpoint between the (*i-*1)-th and the *i*-th visits;
- The primary TDF/FTC efficacy parameter, denoted as TEs, is measured as:

TEs=100%*(1-RR),

where RR is the hazard ratio for HIV infection between the TDF/FTC and placebo groups.

- 1. **Study primary efficacy hypothesis**

The primary hypothesis will be evaluated by the standard of a “pivotal test” designed to demonstrate that TDF/FTC efficacy exceeds a predefined threshold level of 10% (lower bound) and with an estimated point efficacy of 65%. A one-sided pivotal test is formulated as follows:

H0: TEs<=10% vs.

HA: TEs>10%.

- 1. **Study Sample Size Justification**
     1. **Effectiveness Outcomes**

Determination of the sample size and power for the phase III study was based on a time-to-event analysis of the primary efficacy endpoint. Calculations for a test of the primary hypothesis were done over a reasonable range of parameters using the Lachin-Foulkes model[[49]](#endnote-50).

The sample size calculations for the phase III study take into account the following:

- The ratio of randomization to TDF/FTC and placebo groups is 1:1;
- Subjects’ enrollment into the study is assumed to be uniformly distributed;
- The length of the accrual period is 15 months (1.25 years);
- The follow-up is extended for 12 months after the last patient is accrued, and the total length of the study is 27 months (2.25 years);
- The loss to follow-up is assumed to be exponential and independent of the study outcome, and is anticipated at a 15% annual rate;
- There will be one interim and a final analysis of the primary endpoint with a group sequential design and application of the O’Brien-Fleming rule.

Sample sizes for a one-sided significance level of alpha=0.025 that yields power of 80% to detect a difference in HIV incidence between trial arms for different levels of TDF/FTC efficacy and HIV incidence are summarized in the table below.

|  | | **Minimum efficacy level** | | |
| --- | --- | --- | --- | --- |
|  |  | **0.1** | **0.15** | **0.2** |
| **Background HIV rate** | **Expected Efficacy** | **1673** | **2025** | **2500** |
| **0.03** | **0.65** |  |  |  |
|  | **0.7** | **1353** | **1611** | **1949** |
|  | **0.75** | **1108** | **1301** | **1548** |
|  | **0.8** | **917** | **1064** | **1249** |
| **0.05** | **0.65** | **1016** | **1229** | **1517** |
|  | **0.7** | **821** | **977** | **1183** |
|  | **0.75** | **673** | **789** | **939** |
|  | **0.8** | **556** | **645** | **757** |
| **0.07** | **0.65** | **734** | **888** | **1096** |
|  | **0.7** | **593** | **706** | **854** |
|  | **0.75** | **486** | **570** | **678** |
|  | **0.8** | **402** | **466** | **547** |

Assumptions: proportional constant hazards.

Adjustments made for group sequential design, staggered entry, differential follow-up, and 15% expected losses to follow-up.

Assuming an HIV incidence rate of 5%, a sample size of 1016 will provide 80% power at one-sided significance level 0.025 for the primary hypothesis test (H0: TE<=10% vs. HA: TE>10%) and detect at least 65% reduction in HIV hazard.

To account for imprecision in estimated HIV incidence rate and losses to follow-up in the study population, enrollment for the phase III study will be targeted at 1200 subjects.

- 1. **Measures to Minimize Bias**

Multiple methods will be used to reduce a number of possible biases:

- Randomization of participants to treatment arms
- Double blinding of treatment arms (participants and study staff)
- Administer interviews in either English or Setswana at participant’s choice
- Using ACASI to ask sensitive sexual behavior questions at biannual visits
- Immediate tracking for missed visits (including home visitation)
- Frequent ascertainment of primary outcomes
  1. **Randomization**

CDC statisticians will generate the allocation sequence using SAS/STAT software[[50]](#endnote-51). To avoid the potential of selection and allocation bias and provide equal allocation of subjects to study arms and genders, the randomization will be stratified by the study site for each gender. Randomization will be performed in a 1:1 ratio using random permuted blocks with fixed block size of 6 for the TDF/FTC and placebo arms.

- 1. **Allocation Concealment**

Participant ID numbers were randomized to placebo or TDF/FTC and the allocation list was given to Gilead; pill bottles will be filled and labeled according to these participant ID numbers and shipped to the study sites. Participant ID numbers were assigned in series to men and women at each of the two sites (4 series of Participant IDs) Site coordinators at each site will receive the male and female IDs for their site. After eligibility is confirmed and an informed consent form is signed, the next Participant ID in sequence for the indicated gender and site will be assigned and recorded on the relevant study documents.

Links between the Participant ID and the random treatment arm assignment will be held only by Dr. Roman Gvetadze (the trial statistician at CDC) and by Dr. Howard Jaffe at Gilead Sciences.

- 1. **Blinding**

Gilead Sciences will supply matching placebo and TDF/FTC tablets labeled with the randomization code (Participant ID) so that study clinic staff can dispense medication corresponding to the assigned treatment group while remaining blinded.

Using this method, the participants, study clinicians and data management staff, monitors, and investigators actively involved in the trial will not know which participants are using TDF/FTC and which are using placebo.

- 1. **Unblinding Procedure**

The randomization code will be held by Dr. Roman Gvetadze (CDC, Atlanta), and Dr. Howard Jaffe (Gilead Sciences, California). Either of them may be requested to provide the code to the Dr. Paxton (CDC) if needed in an emergency to guide treatment decisions for any participant. This is not expected to occur.

At the conclusion of the study, when primary analyses are complete and the study is unblinded to the investigators, participants will be offered the opportunity to learn into which arm they were randomized.

- 1. **Analysis Plan Summary**

All randomized patients will be included in analysis. Continuous data will be summarized with descriptive statistics, including n, mean, standard deviation, minimum, maximum, median and interquartile range. Categorical data will be summarized using counts and percents. A one-sided significance level alpha=0.025 will be used to perform significance testing and construct confidence intervals. Heterogeneously distributed variables will be regarded as potential confounders and will be adjusted for in multivariable modeling.

To assess the success of randomization, a summary of baseline variables by study arm will be tabulated. For descriptive statistics the following methods will be used: categorical variables will be characterized by number and percent in each category, along with exact Poisson confidence limits; for continuous variables, the mean, median, standard deviation, minimum, maximum and interquartile range will be calculated.

Missing data will be summarized by treatment groups, and missing pattern will be assessed using graphical and modeling approaches and appropriate multiple imputation technique will be applied[[51]](#endnote-52). All analyses will be performed using SAS version 9.1 (Cary, NC, U.S.A).

- - 1. **Analysis of Safety Outcomes**

The assessment of TDF/FTC safety will be performed at the same time as the interim and final efficacy analyses, and will be based on generation of data lists, summary tables, and results of statistical comparison of safety events between the study arms.

The safety cohort will include all volunteers who have been randomized. This cohort is used for summaries and analyses of treatment comparability, trial conduct, safety, risk factors, social harms events, and social impact.

TDF/FTC primary safety outcomes will be assessed in terms of rate of severe clinical and laboratory adverse events in each treatment group. Individual participants will contribute only once to the calculation of event rates. If an AE is reported more than once in any single participant during the study period, the greatest severity and the worst-case attribution are to be presented. Analysis of safety data will be stratified by study site and gender.

The date of randomization will be considered the time origin for the safety endpoint analyses. Volunteers who prematurely left the study will be censored at the date of last visit. Factors that may be associated with AEs will be analyzed. Statistical analysis will be performed using the SAS version 9.1 software package (SAS Institute Inc., Cary, NC, USA)[[52]](#endnote-53).

Analysis of secondary safety endpoints, behavioral safety and treatment adherence, will include longitudinal comparison of responses to questions about risky sexual behavior, and adherence proportions over time between the study groups. The development of antiretroviral resistance will be assessed in terms of proportions of detected HIV resistance mutations in seroconverted subjects.

- - - 1. **Data listing**

All adverse events, including those that occurred on several occasions, will be listed by the participant ID and by the blinded treatment group (e.g., Group A and Group B). As ICH guidelines suggest, the following information will be included in the list of adverse events:

- Patient identifier
- Demographic parameters
- Adverse event
- Duration of adverse event
- Severity
- Seriousness
- Actions taken
- Outcome
- Causality assessment
- Date of onset or date of visit at which the event was discovered
- Duration of test drug treatment
- Concomitant treatment during the study.

All SAEs and any AE that led to temporary or permanent suspension of study drug, or significant additional concomitant therapy, will be listed separately.

- - - 1. **Summary tables**

All AEs occurring after the initiation of study treatment will be summarized in tables, which list each AE, the number of patients in each treatment group and the rate of occurrence. Safety events will be grouped by body system, tabulated for each study arm and organized by frequency, severity, relationship to study medication, actions taken to address the event and outcome. In addition to summary tabulation, graphical presentation in the form of scatterplots and flow charts will be used for preliminary examination of AEs between treatment groups.

- 1. **Inferential statistical analysis (Safety)**

Statistical comparison of safety outcomes will use a significance level of alpha-0.05 to perform significance testing and constructed confidence intervals for AEs and SAEs. Analysis of the distribution of AEs and SAEs across groups defined by baseline predictors will help to identify heterogeneously distributed variables. Such variables will be regarded as potential confounders and will be adjucted for in multivariable modeling. Missing data will be summarized by treatment groups, and missing pattersn will be assessed using graphical and modeling approaches and an appropriate multiple imputation technique will be applied.

AEs, SAEs, and laboratory test results will be compared between the blinded study arms (group A and group B; see safety tables. Rates of AEs among group A and B will be compared using chi-square tests. Longitudinal analysis of AEs will be examined using generalized estimating equasion (GEE) logistic regression. The time to adverse event will be examined using Kaplan-Meier plots, log-rank tests, and Cox proportional hazard models to compare the incidence of AEs.

- - 1. **Analysis of Effectiveness Outcomes (Phase III)**
    2. **Analysis Sets**

Two cohorts are used in the analysis of the primary efficacy endpoint: The intent-to-treat (ITT) cohort and the per-protocol or treatment adherent (TA) cohort.

- - - 1. **Intent-to-Treat Cohort**

The ITT (intent-to-treat) cohort includes all volunteers who meet the following criteria:

- Have been randomized and received at least 1 dose of study medication; and
- Do not have HIV infection at Month 0:
- non-reactive by ELISA test at the screening visit, or
- if reactive by ELISA test within first 3 mo, are negative by PCR at enrollment visit.
  - - 1. **Treatment Adherent Cohort**

The treatment adherent cohort is defined as the volunteers who are in the ITT cohort and meet the following criteria:

- Have taken > 65% of doses as assessed by recall interviews at first three monthly visits
  - 1. **Primary Efficacy Analysis**

The analysis for the primary efficacy endpoint will be based on the ITT cohort. The goal of the primary efficacy analysis is to assess the efficacy of TDF/FTC in reducing transmission of HIV-1 in heterosexual young adults in Botswana, and denoted by TES. This parameter represents the percent reduction in HIV-1 infection incidence attributable to TDF/FTC, and is calculated as TES = 100% × (1 – RR), where RR is hazard ratio, estimated using maximum partial likelihood methods and Cox discrete-time proportional hazards modeling. The lower confidence bound on TES is constructed to test the primary null hypothesis H0: TES≤10%, and the alternative hypothesis, Ha: TES>10%, for the specific alternative TES=65%. A statistically significant result with regard to the positive effect of TDF/FTC will be achieved if the 95% lower confidence bound on TES is above 10%. This can equivalently be phrased in terms of a p-value generated from the appropriate log-rank test statistic.

Discrete-time Kaplan-Meier plots will be generated to display the time-to-infection analysis. The log-rank test will be used to compare the two treatment groups at the 0.025 level of one-sided significance.

- - 1. **Secondary Efficacy Analyses**
       1. **Adjusted ITT analysis**

Multivariable analyses of the primary efficacy endpoint using Cox proportional hazards model or other appropriate failure–time analytic methods with heterogeneously distributed covariates will be performed. In addition, univariate analysis entailing each of the covariates will be made, and assessments of possible differential TES by covariate levels will be made via Wald and likelihood ratio tests for interaction in Cox models. The overall HIV-1 infection rate will be estimated using both the ITT and TA cohorts. The HIV infection rate is defined as the number of HIV-1 infections per 100 person-years followed. A confidence interval for the infection rate is estimated based on the Poisson distribution. The estimates of absolute risk complement the analysis of relative risk arising out of the primary efficacy analysis. Likewise, inclusion of the aforementioned covariates will also be made in estimating the HIV-1 infection rate using a Poisson regression model.

- - - 1. **Per-protocol analysis**

The per-protocol analysis will be based on TA cohort and will employ the same methods as used for the ITT analyses.

- - - 1. **Sensitivity Analysis**

Sensitivity analysis will be performed to evaluate the robustness of the estimated TES. The scope of sensitivity analysis will include: ITT versus TA cohorts, ITT versus multiply imputed cohorts, and TA versus multiply-imputed cohorts.

- 1. **Interim Analysis Plan Summary**

The purpose of the interim analysis is two-fold:

- to provide an opportunity to stop the trial in the case of a very high efficacy of TDF/FTC
- to evaluate the need to adjust the sample size

There will be one interim analysis done when either 50% of the expected HIV seroconversions, or 50% of the total expected PY of follow-up, have been observed, whichever comes earlier. The significance level for interim (and final) analyses will be adjusted using the O’Brien-Fleming group sequential design to preserve the overall type I error at the 0.025 level. Specifically, statistical tests at the interim analysis will be performed at a one-sided alpha= 0.001523 (critical Z=2.963).

The following stopping rules will be applied at the time of interim analysis:

- continue trial if Z<2.963, or p>0.001523;
- stop trial if Z>2.963, or p<0.001523
  1. **Final analysis**

The final analysis will be performed at the end of the study. The same statistical methods will be applied as for the interim efficacy analysis. According to the O’Brien-Fleming design, the analysis will be performed using an alpha=0.024477 level of significance (critical Z=1.969).

- 1. **Statistical issues**

Missing or incomplete data caused by dropping out of the study before completion could represent a major problem in the analysis. The expected annual dropout rate of 15% has been taken into account in sample size calculation, so that more patients will be enrolled in order to provide a sufficient number of evaluable patients to achieve the desired power. The causes of dropping out will be carefully evaluated, especially when there are a large number of dropouts. At interim and final analyses, the distribution of subject disposition, duration of follow-up, and reasons for discontinuation will be summarized and compared between treatment groups, which will help to decide the mechanism of non-response is ignorable (missing completely at random, MCAR, or missing at random, MAR) or non-ignorable (missing not at random, MNAR) and to apply an appropriate multiple imputation technique[[53]](#endnote-54),[[54]](#endnote-55).

Estimation of TEs assumes time constant relative risk over the duration of the study. The proportional hazards assumption will be checked by plotting the cumulative hazard functions, and investigating Schoenfeld residuals. Additionally, modeling time dependence will perform formal test of the proportionality hypothesis.

1. **TRAINING PLAN**

Before trial initiation, all trial staff completed Good Clinical Practice training to the FDA/ICH standard. In addition staff underwent protocol training for several weeks on tasks they are assigned. For example, counselors receive in depth training and undergo monitored practice until they have demonstrated proficiency in HIV test counseling, individualized risk reduction counseling, medication adherence counseling, and supportive counseling for HIV positive persons. Similarly, clinicians have training in the exam protocol, specimen collection, AE/SAE identification and reporting, and medication dispensing/accountability. Similar specialized training is offered for each trial position.

1. **MONITORING PLAN**
   1. **Clinical Monitoring Plan**

External study monitors (PPDI) visit the site regularly during the trial to:

- verify compliance with human subjects and other research regulations and guidelines;
- assess adherence to the study protocol, study-specific procedures manual, and local counseling practices; and
- confirm the quality and accuracy of information collected at the study site and entered into the study database.

Site investigators will allow study monitors to inspect study facilities and documentation (e.g., informed consent forms, clinic and laboratory records, other source documents, case report forms), as well as observe the performance of study procedures. Investigators also will allow inspection of all study-related documentation by authorized representatives of the U.S. Centers for Disease Control, Gilead Sciences, Inc., and US and in-country government and regulatory authorities. A site visit log is maintained at the study site to document all visits.

- 1. **Safety Monitoring Plan**

The Principal Investigator is responsible for continuous close monitoring of all adverse events (AEs) that occur among study participants, and for alerting the rest of the study investigators if unexpected concerns arise.

In the Phase III trial, the Data Safety Monitoring Board will be responsible for periodic (or emergency) review of safety data, and decisions about holding accrual or administration of study product for safety concerns.

- 1. **Data Safety and Monitoring Plan**

CDC is also funding a TDF oral prophylaxis trial among injection drug users in Thailand. The Botswana trial uses the DSMB already established and experienced with CDC-funded clinical HIV prevention and vaccine trials in Thailand and Botswana. The DSMB is composed of 3 members from Thailand, 3 members from Botswana, and 2 members from the U.S. The three members from Botswana are: 1) the former Chief Pharmacist of the Drug Regulatory Unit in the Ministry of Health, 2) the Medical Director of the Infection Disease Care Clinic (HIV service) at Princess Marina Hospital (the national referral hospital in Gaborone), and 3) a biostatistician from the University of Botswana.

The DSMB receives blinded safety reports each quarter and meets formally for scheduled reviews and for special meetings when they determine a need for closer examination of specific issues. An interim safety and trial performance review is scheduled midway through the trial (when either 1/2 of expected seroconversions or 1/2 of expected person-years is accrued, whichever comes first) and a final safety and efficacy review at the conclusion of the trial (when the last person enrolled has completed 12 months of follow-up).

A data safety monitoring plan has been written and agreement about the plan reached with the DSMB. It includes the following:

- Specification of the adverse event detection and reporting system
- Content of interim data reports
- Frequency of interim data reviews
- Statistical methods for interim analyses
- Specification of stopping rules
- Distribution of interim review reports

Interim reports will include at least the following information, by treatment arm, clinic, and gender/age strata:

- Number randomized
- Number and timing of missed visits and drop-outs, accumulated follow-up time
- Baseline characteristics of study population
- Medication adherence
- Rates of unprotected vaginal sex
- Protocol violations, including ineligible persons enrolled, number who received an unassigned treatment, instances of unblinding
- Safety and efficacy outcomes

Number, percent, and rate of occurrence of primary outcomes

Number, percent, and rate of occurrence of secondary outcomes

Line listings of adverse events

Interim reports will also include:

- Multivariate analyses of outcomes adjusted for baseline differences between arms/strata, Analyses of efficacy outcomes adjusted for medication adherence
- A summary of GCP monitoring reports by site.

At predetermined review intervals, the board will be convened (in person or by phone) to review the data provided and make a recommendation about whether safety and efficacy interim data indicate the continuation, revision, or discontinuation of the Phase III trial

- 1. **Protocol Compliance Monitoring Plan**

Every attempt will be made to conduct the study in full compliance with the approved protocol. Ongoing monitoring of staff compliance with protocol procedures will be performed on-site by the study coordinators, reviewed by the PI, and corrective actions taken (and documented).

- - 1. **Protocol Amendments**

The protocol will not be amended without prior written approval by the sponsor and the Principal Investigator. All protocol amendments must be submitted to and approved by the CDC IRB and the MOH HRDC prior to implementing the amended procedures

- - 1. **Emergency Protocol Violations**

When a departure from protocol is crucial to protect the safety and well-being of an individual participant, it may be instituted for that patient only and the Principal Investigator will be informed of the event immediately. The PI will promptly notify the CDC IRB and the MOH HRDC in writing including documentation of the reasons for deviation from the protocol, the outcome for the patient, and if any changes in the protocol are indicated based on this event.

- - 1. **Non-Emergency Protocol Violations**

Significant departures from the protocol that result from staff error and are not urgent safety concerns for participants will also be promptly reported to the PI and a memo written to document to occurrence and any corrective actions which have been taken or are recommended to prevent a recurrence. Copies of these memos will also be sent to the CDC IRB and MOH HRDC. Examples of these violations include:

- Omission or improper administration of informed consent
- Enrolling ineligible participants
- Treatment errors, either failure to deliver indicated treatment or administration of incorrect treatment
- Incorrectly timed or omitted study procedures and assessments
- Coercing a participant to remain in the study after they express a desire to withdraw
- Failure to discontinue study medication or discontinue a participant when indicated by the protocol
- Evidence of falsified data by any member of the study staff

1. **LOCAL ADVISORY GROUPS**

We will establish formal mechanisms to solicit and respond to attitudes about study procedures, the reputation of study in the community, and questions arising from participants or the general community. These groups will be consulted throughout the conduct of these trials.

- 1. **Community Advisory Groups**

To facilitate communication with varied sectors of the community, in each of the two trial communities, a Community Advisory Group (CAG) has been established and meets at least quarterly. The CAGs are composed of representatives of local organizations and community sectors whose areas of work overlap significantly with the objectives of, and populations enrolled in, microbicide, PrEP and other HIV prevention studies being conducted through the BOTUSA Project. The two CAGs meet together at least once per year.

- 1. **Participant Advisory Groups**

To provide a venue for consulting with study participants, in each of the two trial communities, a Participant Advisory Group (PAG) has been established and meets quarterly. The PAGs is composed of study participants and a few persons who have been screened but elected not to participate in studies. The two PAGs will meet together at least once per year. Representatives from the PAG will also serve on the CAGs and the Botswana HIV Microbicide Research Reference Group.

- 1. **Botswana HIV Prevention Research Reference Group**

To provide for regular communication with varied local and national government stakeholders and to assure that our procedures for HIV prevention trials (including microbicides) are not in violation of existing policies, an HIV Prevention Research Reference Group has been formed and meets quarterly. Studies are presented to them in the planning stages to obtain their advice, and they are informed about study progress and findings.

1. **DATA MANAGEMENT PLAN**
   1. **Coding of Safety Events and Medications**

Before data entry, all AEs will be coded for diagnosis and site using ICD9-CM. All concomitant medications will be coded by drug class using a modification of American Hospital Formulary Service scheme.

- 1. **Information Management and Analysis Software**

Core data entry and management will be done using Clindex 3.0.4 or later (Fortress Medical, Hopkins, MN), a 21 CFR part 11 compliant combined clinical trials management and clinical trials data management software package. Key data collection forms (CRFs) will be double-entered at each site into this software (e.g., enrollment, HIV lab test results); 10% of all the other forms will be double-entered. Laboratory data and graphic images (e.g., digital photos of rashes with masking of identifying portions if on the face) will also be imported into the database.

Microsoft Access, Visual Basic (VB), and VB Script (Microsoft Corp, Redmond, WA) will be used to develop accessory data entry systems which will interface effectively with the core Clindex database, allowing export and import of other relevant data in a part 11 compliant manner. These accessory data systems will be used, for example, by collaborating local laboratories to enter participant results in real-time, allowing periodic batch importation into the core database. Other accessory data systems anticipated, would include documenting staff training/annual retraining and managing inventory of study product, disposables, and testing kits.

All protocol-related paper forms completed by participants or staff will be scanned into pdf (portable document format) files using Adobe Acrobat, version 5.0 or later (Adobe Systems Inc., Seattle, WA) and PaperPort Pro, version 9 or later (Scansoft Inc., Peabody, MA) will be used to archive scanned forms and other electronic files (e.g., PAP smear and cervical digital image files).

QDS, version 2.0 or later (Nova Research, Bethesda, MD) will be used to program the ACASI screens. An exportable database is produced containing participant responses to questionnaire items which will be imported into analysis software.

NVIVO version 1.3 or later (QSR International Pty Ltd., Melbourne, Australia) will be used for focus group textual data coding and analysis.

All other data analysis will be done using SAS for Windows, version 8 (SAS Institute, NC).

- 1. **Quality Assurance of Form Data Collection**

Procedural, face-to-face interview, clinical examination, and laboratory test result data will be entered onto paper forms (see Appendix L) by the staff member who generated the data. A supervisory staff member will review forms for completeness, date, and sign the form.

Laboratory result forms will be sent to the site physician for review and determination whether any clinical follow-up or treatment that may be indicated. All other forms, and laboratory result forms after they have been physician-reviewed, will be sent to clerks for data entry.

Quality control reports and queries will be routinely generated and distributed to the study clinic staff for verification and resolution.

- 1. **Quality Assurance of Computerized Data Entry**

The protocol rules (eligibility criteria, visit windows, forms expected at each visit, etc) have been programmed into Clindex along with data entry screens for all study data collection forms. Form and field checks have been programmed to minimize the ability to enter data which is out of range or not in protocol compliance. Eligibility, interview, examination, and laboratory result forms will be 100% double-entered by two separate data entry clerks. After verification of low data entry error rates, a random 10% of all other forms will be double-entered. The study data manager will review and reconcile double-entered fields that do not match.

All trial data collected on paper forms will be entered at each study clinic via dial-up connection to a password protected central server at BOTUSA. Data cleaning, query generation, and edits are done on this central master database. Locally and centrally generated queries are sent to relevant study staff for clarification, and final resolution is completed on the master database at the direction of the data manager. Clindex maintains a complete audit trail so that both original data entry and subsequent edited data are in the database with date/time/user stamps and the reason for each data change made.

ACASI data from desktop computers is backed up daily onto servers at the clinics in password-protected folders. These folders are zipped weekly and transferred electronically to the BOTUSA server. The senior data manager then reviews them for completeness. Since this data is entered directly by trial participants into the computer, no queries are generated by this review.

- 1. **Data Storage**

The Investigator will maintain, and store in a secure manner, complete, accurate and current study records throughout the study. In accordance with US federal regulations, for the investigational product tested, the PI will retain all study records for at least five years after the conclusion of the study and two years following the date of marketing approval for the study product for the indication in which it was studied (whichever is longer). Study records include administrative documentation — including protocol registration documents and all reports and correspondence relating to the study — as well as documentation related to each participant screened and/or enrolled in the study — including informed consent forms, locator forms, case report forms, notations of all contacts with the participant, and all other source documents.

1. **ADMINISTRATIVE PROCEDURES**
   1. **Study Initiation**

The following documents were in place at the study site before any potential participants were contacted:

- Signed agreement between Gilead Sciences, Inc. and CDC
- CVs for trial investigators, study coordinators, laboratory coordinators
- Signed protocol and attachments
- Sample CRFs
- Information given to participants (e.g., consents, educational materials, recruitment materials)
- Instructions for handling of investigational product and other trial related medications
- Decoding procedures for blinded trials
- IRB/EC approvals and composition
- Botswana Ministry of Health Drug Regulatory Unit approvals
- Laboratory accreditations
- Normal laboratory test values
- Financial disclosure forms for each trial investigator
- Financial records
- Shipping records
- Site visit log
  1. **Study Conduct**

During the conduct of the study, any revisions to the above listed documents (section 14.3), and additional documents listed here will be in place and available for review by monitors.

- Documentation of investigational product and trial-related materials shipment.
- Investigational products accountability documents
- Relevant communications (other than site visits)
- Signed consent forms
- Source documents
- Completed CRFs with documentation of CRF corrections
- Participant screening log/listing
- Participant enrollment log/listing
- Staff signature list
- SAE notifications
- Interim or annual reports to IRBs, ECs, regulatory authorities, and CDC
- Progress reports to CDC
  1. **Study Coordination**

Gilead Sciences, Inc. will provide TDF/FTC and placebo for the study. Assignment of all sponsor responsibilities for this study is specified in a Clinical Trials Agreement executed by the U.S. Centers for Disease Control and Gilead Sciences, Inc.

The Division of HIV/AIDS Prevention, U.S. Centers for Disease Control will provide statistical and data management support for this study. The HIV Prevention Research Unit at the BOTUSA Project will serve as the coordination and operations center for study development and implementation.

- 1. **Study Completion**

After completion of each trial, all of the documents in sections 14.3 and 14.4 will be in files together with the following:

- Documentation of investigational product destruction (if destroyed at the sites)
- Complete participant ID code list
- Final report by investigator to IRBs, ECs, regulatory authorities, and CDC.
  1. **Site Record Retention and Access to Documents at the Site**

Paper records will be stored on-site until data entry is completed, database queries are resolved, and records have been externally audited by study monitors. They will then be archived according the CDC standards and stored off-site in secure facilities. Permission to destroy the paper records will be sought after closure of the study and completion of all audits and close-out activities.

All paper source documents and some administrative documents will be scanned and stored as electronic files in portable document format (pdf) or as graphics images. These will be archived electronically using CDC standards and at the close-out of the study, will be securely transferred to CDC Atlanta for long-term storage.

1. **LABORATORY SPECIMENS AND BIOHAZARD CONTAINMENT**
   1. **On-site and Local Laboratory Testing**

Clinicians will be trained and their proficiency tested for the performance and interpretation of rapid whole-blood testing for HIV (Determine HIV, UniGold HIV, and Oraquick), syphilis (Determine TP) and urine pregnancy (AimStick®). Upon completion of training, clinicians will perform this testing in the presence of study participants in exam rooms.

There are five types of laboratories involved in these trials. At each study clinic, there is a small on-site laboratory. In each town, there are small HIV prevention trial support laboratories equipped for microbiology, chemistry, haematology and serology. In Gaborone, there is a small HIV prevention and microbicide research laboratory operated by BOTUSA equipped for immunologic and some virologic assays. And there are contract laboratories in South Africa and the United States performing specialized tests not available in Botswana.

On-site laboratories at each site research clinic (Francistown and Gaborone) will be responsible for completing laboratory tests which include: wet prep and KOH slide readings, gram stain, In-Pouch trichomonas culture, and centrifuging of CPT and serum tubes. On-site laboratories will be responsible for the labelling, packaging for shipping, and transport of specimens to local and reference laboratories for additional testing and repository.

The small HIV prevention trial support lab allied with each research clinic will be responsible for completing laboratory tests which include: EIA for HIV, EIA for HSV-2, EIA for hepatitis B profile, chemistry, haematology, gonorrhea and chlamydia (Cobas Amplicor), HIV viral load (Cobas Amplicor), CD4, and ThinPrep slide preparation and initial screening for PAP smears. Cytopathology will be performed in an accredited laboratory in South Africa. MOH pathologists in Francistown and Gaborone will be responsible for final reading of PAP smears (ThinPrep).

Each study-associated laboratory will adhere to standards of good laboratory practice and local standard operating procedures (SOPs) for proper collection, processing, labeling, transport, and storage of specimens. Specimen collection, testing, and /or storage at the on-site and local laboratories will be documented using laboratory data management software as described in the study-specific procedures manual.

The senior laboratory scientist and laboratory coordinator will review proficiency testing results for all labs, designate and monitor the testing of samples selected for quality assurance testing, and perform some of the QA testing (e.g., HIV EIA) at the research lab in Gaborone. The senior laboratory scientist will follow-up directly with on-site and local laboratory staff to resolve any quality assurance problems identified through this process.

- 1. **Central Laboratory Specimens And Shipment Procedures**

Laboratories at Johns Hopkins University and Gilead Sciences with extensive antiretroviral research experience have been contracted to complete the TDF/FTC plasma and intracellular concentration assays.

Assays which BOTUSA labs do not have capacity to perform (e.g., resistance testing, CTL assays) will be performed in other CDC laboratories to be determined.

All specimens will be shipped in accordance with IATA and CDC specimen shipping regulations. All shipments will be documented using the laboratory data management system as described in the study-specific procedures manual

- 1. **HIV Testing Algorithm** (See Appendix J)
     1. **Screening Visits**

Initial HIV testing during screening for eligibility will be done by collection of fingerprick blood samples for dual, parallel, whole-blood rapid testing with Determine and Unigold lateral flow test strips. This procedure has been validated in Francistown in 2000 (100% sensitivity and specificity against dual EIA) and is currently used in the voluntary counseling and testing centers throughout the country. Based on results of these tests, participant counseling and additional testing will be done as follows:

Both tests valid and negative (concordant negative)

- Inform the participant that there is no evidence of HIV infection and tell them about the window period.
- A random 5% of negative tests will have EIA done for QA

Both tests valid and positive (concordant positive)

- Inform the participant that they have HIV infection and that confirmatory tests will be done and the results given them at a follow-up visit in 1-2 weeks
- (EIA) as well as tests to see if they are eligible for antiretroviral treatment (CD4 count).
- All positive tests will have an EIA done for QA
- All persons who test positive will have blood drawn for CD4 testing to prioritize their referral for follow-up care and possible ARV treatment at MOH clinics

Both tests valid, one positive and one negative (discordant)

- Inform the participant that the fingerstick blood test didn’t agree; we can’t tell if they have HIV infection or not; there is a chance that they have been infected very recently but also may not be infected; and that we will test their blood in the lab and give them results at a follow-up visit in 1-2 weeks.
- Treat the participant in all other respects at HIV-uninfected unless and until positive status is confirmed.
- All discordant tests will have an EIA done to determine HIV status
- If positive, blood will be drawn for CD4 count at follow-up visit

Either test invalid/indeterminate (control strip nonreactive)

- Inform the participant that the fingerstick blood test didn’t give a good reading; we can’t tell if they have HIV infection or not; and that we will test their blood in the lab and give them results at a follow-up visit in 1-2 weeks.
- All indeterminate tests will have an EIA done to determine HIV status
- If positive, blood will be drawn for CD4 count to be given to the participant at the follow-up visit
  - 1. **Follow-up Visits**

HIV testing will be done at monthly follow-up visits for two reasons:

1) to reduce the time on TDF/FTC therapy for those who become HIV infected as this may lead to resistance and restrict future ARV treatment choices, and

2) to determine when seroconversion happened as precisely as possible and with behavioral data as closely correlated as possible.

To accomplish this and reduce the number of painful procedures (fingerprick), and realizing that only a very small proportion of follow-up visits will detect HIV seroconversion, we will use a single oral transudate rapid test (Oraquick) to screen for seroconversion at follow-up visits.

Persons who test negative

- will be told that they have no evidence of HIV infection
- 5% will be tested by EIA for QA

Persons who test positive

- Will be told that they may have become HIV infected and dual fingerprick whole blood tests will be done to confirm their status so that they can be counseled appropriately at that visit.
- They will also be told that we will confirm their HIV status in the lab; and schedule a follow-up visit in 1 month
- All positive oral tests will have an EIA done for confirmation and QA (regardless of the rapid test result)
- All persons who test positive will have blood drawn for viral load by PCR (Amplicor) and CD4 testing to prioritize their referral for follow-up care and possible ARV treatment at MOH clinics. They will also have standard and ultrasensitive HIV resistance testing. These results will be provided to the participants and/or their healthcare providers to guide future treatment decisions. Participants will also be provided a letter explaining their participation in this clinical study and encouraged to show this letter to their HIV/AIDS care providers.

Person who have an invalid/indeterminate test

- Will be told that the test didn’t give a good reading, we can’t tell their status, we will test their blood in the lab; and schedule a follow-up visit in 1-2 weeks
- Treat the participant in all other respects at HIV-uninfected unless and until positive status is confirmed.
- All indeterminate tests will have an EIA done to determine HIV status
- If positive, blood will be drawn for viral load and CD4 count results to be given at the next follow-up visit. They will also have standard and ultrasensitive HIV resistance testing. These results will be provided to the participants and/or their healthcare providers to guide future treatment decisions. Participants will also be provided a letter explaining their participation in this clinical study and encouraged to show this letter to their HIV/AIDS care providers.
  - 1. **HIV Seroconvertor Assays**

Following seroconversion at any study visit, the following will be done:

- Test the stored PBMCs from the prior visit (including screening when indicated) by PCR to determine if the participant was in the window period when they previously tested negative.
- Conduct genotypic and phenotypic resistance testing on samples from the seroconversion visit (or the prior visit if virus detected at that visit)
  1. **Specimen Storage and Possible Future Research Testing**

Study site staff will store all specimens collected in this study at least through the end of the study. In addition, study participants will be asked to provide written informed consent for their specimens to be stored for up to 10 years after the end of the study for possible future testing. Testing on these stored specimens not already described in this protocol will only be done after obtaining approval from the HRDC and CDC IRB. The specimens of participants who do not consent to long-term storage and additional testing will be destroyed at the end of the study

- 1. **Biohazard Containment**

As the transmission of HIV and other blood-borne pathogens can occur through contact with contaminated needles, blood, and blood products, appropriate blood and secretion precautions will be employed by all personnel in the drawing of blood, and shipping and handling of all specimens for this study, as currently recommended by the United States Centers for Disease Control and Prevention.

1. **ETHICS AND RESEARCH INTEGRITY**

Individualized HIV risk reduction counseling, HIV testing, condoms, family planning services, and STI diagnosis and treatment are available at no cost, both in the community and within the trial. Therefore, no participant will be deprived of a proven HIV prevention technology to which they would otherwise have access

Clinical care will be provided to participants as follows:

- All participants will receive family planning counseling and women will receive the hormonal contraceptive of their choice from study staff
- STI diagnosis and treatment will be provided at no cost.
- Evaluation of adverse events will be provided with treatment if indicated and available at the study clinic. If not available, referral to MOH facilities will be arranged with the urgency indicated by the condition.

In addition, because of the safety of TDF/FTC during pregnancy is not yet well established, an effective contraceptive method will be required to be used by all female participants enrolling in the trial.

All study participants who require admission to the hospital will receive close monitoring and follow-up.

- 1. **IRB/EC Review and Approval**

The protocol informed consent forms, participant education and recruitment materials, and other requested documents — and any subsequent modifications — will be reviewed and approved by the ethical review bodies responsible for oversight of research conducted at the study site, the CDC IRB and the Health Research Development Committee of the Botswana Ministry of Health.

Subsequent to initial review and approval, the responsible local Institutional Review Boards/Ethical Committees (IRBs/ECs) will review the protocol at least annually. The Principal Investigator will make safety and progress reports to the IRBs/ECs within three months of study termination or completion. These reports will include the total number of participants enrolled in the study, the number of participants who completed the study, and a line listing of all non-serious adverse events (SAEs having already been reported). In addition, all open DSMB reports will be provided to the IRBs/ECs.

- 1. **Risks**

There are few anticipated health risks to participation in the trials other than minor discomfort associated with physical examinations and blood specimen collection. A small number of persons randomized to TDF/FTC may experience side effects (e.g., flatulence) or laboratory toxicities (e.g., elevated creatinine) but most will not and some persons randomized to placebo may also have these findings.

In addition, some sexual behavior questions may be embarrassing.

Although study sites will make every effort to protect participant privacy and confidentiality, it is possible that participants' involvement in the study could become known to others (usually through participant disclosure), and that harm may result (e.g., being misunderstood to be participating in a study for HIV positive persons, or being harassed to share their medication for prevention).

- 1. **Benefits**

Participants in this study will benefit from learning their HIV status, obtaining individualized, ongoing risk reduction counseling, and being monitored and treated for STIs. Participants and others may benefit in the future from any information learned from this study that may lead to the development of a safe and effective intervention that prevents HIV infection.

- 1. **Post-trial Access to Effective Products**

If this study, or other oral antiretroviral prophylaxis studies, determine that TDF/FTC is effective in reducing sexual HIV transmission, all HIV seronegative participants in this trial (both arms) will be provided with TDF/FTC at no cost until the Botswana Drug Regulatory Unit of the MOH makes a determination about approval for use for this indication but no longer than 1 year. At minimum, plans will be made so that participants in the Phase III trial who elect to take TDF/FTC after this trial will be monitored at a level consistent with Phase IV post-marketing studies. CDC will make the necessary provision for TDF/FTC to be provided by Gilead or purchased at cost by CDC.

- 1. **Informed Consent**

Formal assessment will be done as the final eligibility criteria for each potential participant to assess their understanding of the elements of informed consent (purpose of the study, procedures and visit schedule, voluntariness, right to refuse or withdraw, etc). Persons who twice fail the comprehension test will not be offered trial participation.

Oral consent will be obtained to ask eligibility and HIV knowledge questions. Written informed consent will be obtained from each study participant for screening laboratory testing and study enrollment.

The lower age limit of 18 years for permitting autonomous consent and enrollment was selected based on a review of existing regulations and practices in Botswana regarding age of consent for various activities.   There is currently no legal age of consent for participating in medical research.  However, young people of any age are able to obtain a wide range of medical treatment without parental/guardian consent (e.g., antenatal care, family planning, STD diagnosis and treatment).  Recent discussions by the National AIDS Coordinating Committee and the Ministry of Health have led to a recommendation that 16 be the age for autonomous consent to HIV testing and HIV care, including the receipt of antiretrovirals for treatment. Further, the age of 18 years is set as the age at which youth are able to join the military service, vote, drive, gamble, purchase alcohol and be tried for a crime as an adult.

A recent advisory from the Attorney General’s chambers to the Ministry of Health has recommended that, at present, person under the age of 21 not be enrolled in clinical trials without parental/guardian consent. They advised further that when the age for autonomous consent of HIV testing and care is lowered by act of parliament (age 16 is recommended), they would similarly lower the age of participation in HIV clinical trials without parental/guardian consent to the enacted lower age. We therefore will enroll young adults 18-20 when we can obtain the consent of at least one parent for screening and enrollment until the new age limit is established and will then enroll without parental/guardian consent.

To protect the confidentiality of those 18-20 years of age, parental/guardian consent will be sought for them to be screened and, if eligible and consenting, for the young adult child to enroll into the trial without further parental/guardian consultation. In the parental/guardian consent form, it will be clearly stated that if a parent gives consent for screening, they are also consenting to their adult child making an autonomous decision whether to enroll in the trial. It will also be clearly stated that no test results or study information provided by the adult child will be shared with the parent by study staff. Potential participants ages 18-20 whose parent is not willing to provide screening consent with these conditions, will not be screened or enrolled. The assent form for those 18-20 years of age will be the same consent form used for those 21-29 years of age.

Potential participants 18-20 years of age who report that their parents are dead or unavailable because they live a great distance away (> 1 hours travel) will be permitted to identify an adult relative acting as a guardian. In the BAISII Survey, 52.6% of persons ages 15-18 were not living with any biological parent (table 35, page 94)^11^. Identified guardians will sign a document confirming the status of the biological parents and the relationship of the guardian to the participant (See Appendix A). The guardian will provide their Omang and contact information so that the HRDC can, if they wish, contact them to confirm their guardianship role. Where a guardianship is documented, they may sign the parental/guardian consent form permitting the young adult to be screened.

Literate participants will document their provision of informed consent by signing their informed consent forms. Non-literate participants will be asked to document their informed consent by marking their informed consent forms with a thumbprint in the presence of a literate witness.

Consenting participants will be offered a copy of their informed consent forms. In Botswana HIV study participants sometimes decline to receive a copy because of concerns about the confidentiality of their participation if it is found by family members or others.

All consent forms, educational and recruitment materials, and study forms will be translated by fully bilingual staff, each backtranslated by a separate staff member, and then reconciled and reviewed by a third, senior staff member fluent in both languages.

- 1. **Participant Confidentiality**

All study-related information will be stored securely at the study sites. All participant information will be stored in locked file cabinets in areas with access limited to study staff. All laboratory specimens, reports, study data collection, process, and administrative forms will be identified only by a coded number to maintain participant confidentiality. All records that contain names or other personal identifiers, such as locator forms and informed consent forms, will be stored separately from other study records in locked cabinets. All local databases will be secured with password-protected access systems. Forms, lists, logbooks, appointment books, and any other listings that link participant ID numbers to other identifying information will be stored in a separate, locked file in an area with limited access.

A photograph will be taken of each participant at screening and will be used for two purposes. First, to verify the identity of the participant at each visit. This will prevent participants sending others to keep their appointments (and the collection of erroneous data). The photos will also be used during home visits and outreach efforts when participants miss scheduled visits to verify participant identity and prevent inadvertent disclosure of confidential information. People who do not wish to be photographed at the study sites will be offered the option of bringing in a photograph at their second visit (when screening test results are given).

Participant’s study information will not be released without the written permission of the participant, except as necessary for monitoring by PPDI, Gilead Sciences, Inc.; the US Food and Drug Administration, the Botswana Drug Regulatory Unit of the Ministry of Health, and/or other government and regulatory authorities

- 1. **Incentives/Reimbursement**

Pending IRB/EC approval, participants will be compensated for their time and effort (variable amount), and travel (30 Pula each visit, 5.5 USD ) for trial participation. Amounts for time and effort will vary between 5 and 15 Pula (0.90 and 1.80 dollars) for main study visits depending on the duration and invasiveness of procedures of a visit type. (See Appendix C).

- 1. **Management of Pregnancy after Study Enrollment**

Women who express a desire to become pregnant during the study time frame, are unwilling to use effective contraceptive methods, or are found to be pregnant at screening will be ineligible for enrollment in the trial.

Women who become pregnant during the study will be withdrawn from study drug. Drug withdrawal will occur after a single urine pregnancy test; a second pregnancy test for confirmation will be performed 1 month after the first positive test[[55]](#endnote-56).

Antenatal care will not be provided by the study but women will be referred to the antenatal care clinic/office of their choice.

Pregnant participants will be followed through delivery and will be asked to return to the study clinic 4-6 weeks after delivery to assess newborn health. In addition, a medical record release will be obtained to review the delivery medical record. These data will also be anonymously reported to Gilead Sciences.

- 1. **Access to General Medical, Antenatal, and Specialized Care**

There are referral public hospitals and clinics in both cities where the trials will be conducted. Participants who require care for illnesses or conditions identified during screening or during trial follow-up visits, will be referred to these resources.

Similarly, there are community-based organizations that provide services to persons with special problems (e.g., domestic violence, alcohol abuse) to which trial participants will be referred if study counselors identify the need for additional services.

- 1. **HIV-related care**

Botswana provides free highly active antiretroviral therapy and prophylaxis for opportunistic infections to all its citizens with CD4 cell counts <200 cells/µl, AIDS-defining illness, or prior treatment during pregnancy for prevention of perinatal transmission. Specialized ARV clinics are established in both communities where the trials will be done and all seroconverters will be referred there for ongoing medical care.

Trial participants who seroconvert in the study will continue to receive supportive counseling from study counseling staff and will also be referred to local community-based organizations that provide support services to HIV-infected people.

1. **PUBLICATION AND PRESENTATION POLICY**

Publication of the results of this study will be governed by CDC and Government of Botswana publication policies. Any presentation, abstract, or manuscript will be submitted by the Investigator to the Director of BOTUSA, the National Center for HIV/STD, and TB Prevention, and Gilead Sciences for review prior to submission. All manuscripts will be submitted to the HRDC before publication.

1. **APPENDICES**

**A: Consent Forms**

**B: Recruitment and Participant Education Materials**

**C: Visit Reimbursement Table**

**D: Laboratory Testing Table**

**E: Investigator’s Brochure**

**F: DAIDS Table for Grading Severity of Adult Adverse Events**

**G: STI Diagnosis and Treatment Guidelines**

**H: Management of Clinical and Laboratory Adverse Events**

**I: Management of Creatinine Elevation**

**J: HIV Testing Algorithms**

**K: Data Collection Forms**

**L: Qualitative Sub-Study Interview Guide**

**M: Healthcare Provider Letter**

**N: Antiretroviral Resistance Testing Results – Letter for Participants**

**O: Antiretroviral Resistance Testing Results – Letter for Healthcare Providers**

**P: Antiretroviral Resistance Testing Results Template**

**Q: References**

1. **CONSENT FORMS**
   1. **Oral Consent Script for Screening Interview**
   2. **Participant Screening Consent/Assent Form**
   3. **Participant Enrollment Consent/Assent Form**
   4. **Oral Consent Script for DEXA Substudy**
   5. **Parental/Guardian Consent Form**
   6. **Statement of Guardianship**
   7. **Qualitative Substudy Consent Form**
   8. **PK/PD Substudy Consent Form**
   9. **HIV Test Consent Form** (screening visits only)
   10. **Confidential Client Referral Form** (HIV positives only)
   11. **Authorization for Release of Confidential Medical Information** (all non-HIV conditions)

**TDF2 HIV Prevention Study**

**Screening Interview Oral Consent Script**

(FK 7.8)

**Introduction**

The Botswana Ministry of Health and the Centers for Disease Control and Prevention in the United States are doing a research study in Gaborone and Francistown.

- The first goal of this study is to learn if a combination medicine (TDF and FTC) works to prevent HIV infection.
- The second goal is to see if it is safe when used for a year or more by young people in Botswana.

This medicine is usually used along with other medicines to fight HIV in people who are already infected. We think it might also work if taken by itself to prevent HIV infection. But we need to do a study to test if it works this way in young people here.

First we need to ask you a few questions to see if you might be eligible to join the study. If we find that you might be eligible for the study, we will explain more about the study and what the next steps are to see if you can join it.

The questions I want to ask now are simple. I will ask some questions about your education, your health, and your sexual history. The interview will not be embarrassing. We will use a code number, not your name on the interview forms.

There is no cost to you. If you agree to be interviewed today, you will receive 30 Pula (7 USD) for travel costs getting to the clinic and home.

You may refuse to answer questions or. If you have questions or concerns about screening, please call and speak to Dr. Poloko Kebaabetswe (390-1696).

If you have any questions about your rights as someone in a research study, complaints about how you were treated in the study, or feel that you have been harmed during screening, please call and speak to Mrs. Shenaaz El-Halabi, Director of the Health Research Unit at the Botswana Ministry of Health (391-4467).

If you want to go ahead and answer some questions today, please understand that:

- It is your free choice to be interviewed or not
- If you wish to come back at another time, that’s ok.
- If you decide not to be interviewed, you will still receive all regular care you get now at any other clinics in the community

***Ask participant if they wish to be interviewed for screening today***

Did participant agree to the screening interview?

⬜ Yes [Complete Pre-ScreeningForm]

⬜ No [Complete Pre-Screening Form]

**Assigned Screening ID [__] [__] [__] [__] [__]**

⬜⬜⬜________________⬜⬜/⬜⬜/⬜⬜

Staff Administering Consent Date: dd mm yy

**TDF2 HIV Prevention Study**

**Screening Consent/Assent Form**

(FK 7.8)

**Introduction**

The Botswana Ministry of Health and the Centers for Disease Control and Prevention in the United States are doing a research study in Gaborone and Francistown.

- The first goal of this study is to learn if a combination medicine (TDF and FTC) works to prevent HIV infection.
- The second goal is to see if it is safe when used for a year or more by young people in Botswana.

This medicine is usually used along with other medicines to fight HIV in people who are already infected. We think it might also work if taken by itself to prevent HIV infection. But we need to do a study to test if it works this way in young people here.

To see if you are eligible to join the study, we would like to ask you to:

- Learn more about the study using a computer
- Have some counseling about HIV tests
- Allow us to take blood and urine samples for testing in the clinic today
- Have some counseling about how to lower your chances of getting or passing HIV
- Return to the clinic in 1-2 weeks

We call this process screening. To help you decide if you will be screened today, please understand that:

- It is your free choice to be screened or not
- If you wish to be screened, but not today, you can come back at another time
- If you decide not to be screened, you will still receive all regular care you get now at any other clinics in the community
- You will be given information about HIV testing and asked to sign a form giving your permission for an HIV test to be done for you today
- If after counseling, you decide not to have an HIV test, we will tell you how to get a test in the future
- If you give permission for the test, you will usually get the results of your HIV tests today. If the two tests we do disagree with each other, you will get your result when you come back for you next visit after we have tested your blood in the laboratory

**What do I need to do if I agree to be screened?**

We will collect a small amount of urine from women to check if they are pregnant.

Then you will be shown how to use a computer to learn more about the study. You can spend as much time as you need. After that, we will answer any questions you have.

If the study still interests you, we will tell you about HIV tests and answer any questions you have about them. We will ask permission to do an HIV test today from three drops of blood we take by fingerprick. If you give permission, we will do the tests while you watch. We will tell you what the tests show right away. A counselor will talk with you about what the tests mean and how to lower your risk of getting or giving HIV.

If your test shows you have the HIV virus now, we will help you get into care. We will help you get additional counseling to help you deal with knowing that you have the virus. We will take 2 small tubes of blood (about 1 tablespoon) to see how the virus is affecting you so far and ask you to come back in two weeks so we can tell you the test results. But you will not be eligible for the study we will be doing.

We will look at the information we get during your first screening visit to see if you might be eligible for the study. If you might be eligible, we will we take 2 small tubes of blood (about 1 tablespoon) to check your health. We will give you written information about the study to take home and think about. We will tell you more about the study and answer your questions.

If it seems that you might be eligible at the end of the first screening visit, we will give you an appointment to come back in 1-2 weeks when we have results from all the blood tests. We will take your picture so that we can be sure you are the only one who comes to the clinic using your name. This will help protect any information you give the study. We will keep a copy of the photo on a computer to use when you come back for your test results. When you have your last study visit with us, we will delete our copy of your picture from the computer.

If you don’t want us to take your picture, we will ask you to bring a picture from home so we can copy it into the computer to use while you are being screened.

At your second screening visit we will:

- Give you the results of the tests that we did
- Help you to get appointments for any care you might need
- If you tested HIV positive, we will give you some education about HIV infection, how it affects your body, and what treatments are available
- If you tested HIV negative, we will tell you if you are eligible to join the study
- If you tested HIV negative, we will give you a study quiz to see if you understand the study well enough to make a decision about joining it
- If you are eligible and you pass the quiz, we will ask you if you want to join the TDF2 HIV Prevention Study

**Will being screened benefit me?**

If you agree to be screened, you will learn your HIV status and other things about your health. You will get education and counseling about ways to keep from getting or passing HIV. You will be given free condoms (both the kind that men wear and the kind that women wear) and taught how to use them. If you have HIV infection or another health problem, we will help you get care.

**How will my information be protected?**

We will use a code number, not your name, on interview forms, blood and urine samples we take for testing, and test results. The code number we give you will be used to keep track of all your records. Consent forms and information you give us to contact you will be kept locked up away from all the other study forms. Only a few study staff will be able to see the list with both names and code numbers in case we need to contact you.

Before your second screening visit, a study staff member will visit you at home to check that we have enough information to contact you. If you miss an appointment we will need to talk to you to give you another appointment in the clinic so that you can get your test results. The study staff member will come in a car that doesn’t mention the study. If you are not at home, they will not leave a message with other people at your house that would let them know you are being screened here.

**are there any risks to being screened?**

Blood drawing may cause some discomfort or bruising. If your blood test shows that you have the HIV virus now, it may be upsetting to you. We will help you if you get upset by giving counseling when you get your test result and at later visits if you want.

Although we will do everything we know how to do to protect your privacy, there is a very small chance that information about you could become known to others. If we become aware that this may have happened, we will contact you to let you know. We ask that if you become aware that it may have happened, you let us know so we can fix any problem that leads to such a problem.

**Is there any cost for being screened?**

There is no cost to you for screening, blood tests, or counseling. If you agree to be screened today, you will receive the 30 Pula (5.50 USD) for travel costs we discussed before the interview, and 10 Pula (1.90 USD) for the time and effort you will spend being screened.

**What are my rights if I agree to be screened?**

You may refuse to answer questions or give blood or urine at any time during the screening visit. If you have questions or concerns about screening, please call and speak to Dr. Poloko Kebaabetswe (390-1696).

If you have any questions about your rights as someone in a research study, complaints about how you were treated in the study, or feel that you have been harmed during screening, please call and speak to Mrs. Shenaaz El-Halabi, Director of the Health Research Unit at the Botswana Ministry of Health (391-4467).

If you agree to be screened, you will be agreeing to do several things, as best you can including:

- Give permission for blood to be drawn
- Give permission for an HIV test
- Receive the results of your HIV test today
- Return for another screening visit in 1-2 weeks
- Have your photo taken (or give us a photo) for identification
- Have confidential home visits and phone calls made if you miss your next visit
- If you are eligible, consider being in the HIV prevention study

***Ask participant if they wish to be screened for the study***

**Statement of Consent**

I have read this consent form or had it read to me. All my questions have been answered. I have been told the purpose of the screening, what will be done, and the risks and benefits of being screened. I agree to be screened.

__________________ ___________________ ___________________

Name Signature Date

_______

*(thumbprint)

* in case the person is not able to read this form or sign their name, this attests that the consent form has been read and explained accurately by a member of the research staff, and that the person has affixed their thumbprint as consent.

__________________ ___________________ ___________________

Witness Name Signature Date

We are asking your permission for some leftover blood and cells to be stored for future research and testing related to sexually transmitted diseases, including HIV or how your body responds to medicines. They may be stored for up to 10 years. We do not yet know what tests may be done in the future. These tests will only be done after we receive permission from the ethics committees in the Ministry of Health and the Centers for Disease Control and Prevention. If knowing the test results would be helpful to you we will try to tell them to you or your doctor. If you wish to have your specimens removed from storage and destroyed in the future, please contact Dr. Poloko Kebaabetswe (390-1696).

[ ] I agree to have my leftover specimens stored for future testing.

[ ] I do not agree to have my leftover specimens stored for future testing.

__________________ ___________________ ___________________

Name Signature Date

______

*(thumbprint)

* in case the person is not able to read this form or sign their name, this attests that the consent form has been read and explained accurately by a member of the research staff, and that the person has affixed their thumbprint as consent.

__________________ ___________________ ___________________

Witness Name Signature Date

**Assigned Screening ID [__] [__] [__] [__] [__]**

⬜⬜⬜________________⬜⬜/⬜⬜/⬜⬜

Staff Administering Consent Date: dd mm yy

**TDF2 HIV Prevention Study**

**Enrollment Consent/Assent Form**

(FK 8.3)

**Introduction**

The Botswana Ministry of Health and the Centers for Disease Control and Prevention in the United States are doing a research study in Gaborone and Francistown.

- The first goal of this study is to learn if a combination medicine (TDF and FTC) works to prevent HIV infection.
- The second goal is to see if it is safe when used for a year or more by young people in Botswana and if people will take it regularly
- We also want to find out if people change their sexual behavior while they’re in the study

Before deciding whether to be in the study, it is important that you understand that:

- Being in the study is voluntary
- If you join the study but later change your mind, you may stop at any time
- If you decide not to be in the study, or if you stop, you will still receive all regular care provided at other community clinics.

**Why are we doing this study?**

People who are having sex can catch sexually transmitted infections (STIs), including infection with HIV, the virus that causes AIDS. Using condoms is the only way we have to prevent this. But many couples do not use condoms or do not use them every time they have sex. So, we are trying to find new ways to help prevent HIV passing between people when they have sex. One of the new ways is for people who don’t have HIV infection to take medicines that fights the virus. The medicine we will be testing is a combination of tenofovir (also called TDF) and emtricitabine (also called FTC). They come in one pill that is usually used along with other medicines to fight HIV in people who are already infected. In fact the Botswana Ministry of Health is now recommending that TDF and FTC should be used initially to treat people infected with HIV (but always in combination with other medicines). We think it might also work if taken by itself to prevent HIV infection but we haven’t proven that yet.

Because young people are often getting HIV infection in Botswana, we are looking for young people to join the study so we can test TDF/FTC and find out if it works for prevention. There will be 1200 youth in this study, some in Francistown, and some in Gaborone.

We can only test medicines to prevent HIV infection in people who do not have it and are at risk of coming in contact with it by their sexual activities. Since you have tested negative for the virus, may be exposed sexually, and are otherwise very healthy, we would like you to join this study.

**What you need to do if you join the study?**

If you agree to be in the study, we will ask you to come for visits every month for at least 12 months (12 visits). At the end of the first 12 months, if we still don’t have the answer, we may invite you to stay in the study longer.

We would not like to expose unborn children to TDF/FTC so we will ask you to use an effective form of pregnancy prevention while you are in the study.

***First visit (Enrollment)***

At your first visit we will teach you how to answer some questions using a computer. These questions are different than the ones you had for screening. You will be asked more about your sexual history and practices.

We will do a physical examination including an examination of your private parts. We will ask you to give us a urine sample and will take swabs from your private parts to check for sexually transmitted infection (STI) during these exams. If we see any evidence that you have an STI, we will give you treatment for it. We will take a small amount of blood (about 1 tablespoon) for other STI tests and to save for other tests we will do later on.

We will assign you to one of two groups. A computer tells us which group and you have an equal chance to be in either group. One group will get a pill with TDF/FTC, the medicines that may prevent HIV infection and that we want to test for safety. The other group will get a medicine that looks exactly the same but doesn’t have any effect. This is a placebo or “sugar pill”. Until the study is over, we won’t know, and the people in the study won’t know, who is taking which medicine. We do it this way so that we won’t let our hopes about the medicine influence the way we collect the information we need to see if the TDF/FTC is safe. We will talk to you about how to take the medicine.

We will give you counseling about how to lower your chances of ever getting HIV infection. We will give you free condoms. This is important for two reasons. We don’t know who is getting TDF/FTC. Also we don’t know yet if it works to protect people from getting HIV.

If you are one of the first 100 people of your gender enrolled in Gaborone, we will ask you to go to a local radiology clinic for an x-ray of your wrist, lower back, and hip.

We will give you appointments for your next set of study visits. This first visit will last about 2 hours.

***Follow-up Visits – Taking study medicines***

Every month when you come back we will ask you some questions about:

- How well you are doing at taking the pills every day
- How well you are doing at lowering your chances of getting HIV
- How you are feeling physically

We will also

- Collect the bottles of pills we gave you at the last visit and give you a new bottle
- Talk with you about how to handle any problems you are having with taking the pills every day
- Take a swab from your mouth to do an HIV test
- give you more condoms.
- **WOMEN only:** do a urine pregnancy test.

At the first two monthly visits we will

- Take 1 tablespoon of blood.
- After that we will not take blood every month
- The monthly visits will take about 1 hour.

Every three months we will

- Do all the monthly visit things and also
- Counsel you about how to lower your risk of HIV infection
- Take 2 tablespoons of blood
- These visits every three months will take about 2 hours.

At only one of these visits we will

- - Ask you to record what time you took your medicine for the two days before your visit
  - Ask you not to take that day’s medicine until you come to the clinic
  - Take 2 teaspoons of blood before you take your medicine at the clinic
  - Take 3 tablespoons of blood at the end of the visit

The information we get at this special visit will help us understand how the body takes in and stores the study medicine in the cells. This will help us understand how TDF and FTC helps or why it doesn’t help protect cells against getting HIV infection in some people.

Every six months, we will also

- Do a physical exam
- Ask you to give us a urine sample and will take swabs from your private parts to check for STI
- Ask you to use the computer to answer questions
- Give you a quick quiz to see what you remember about the reason for doing the study and your rights in the study
- If you are one of the first 100 people of your gender enrolled in Gaborone, we will do an x-ray of your wrist, lower back, and hip to measure the thickness of your bones.
- These visits every six months will take about 2 ½ hours.

***The last (Exit) Visit***

At the end of the study, we will see you for 1 more visit. This last study visit will take about 1 hour.

***Substudies***

We will have extra studies that some participants will be asked to consider joining. These special smaller studies might involve interviews or extra blood collection. 200 people in Gaborone will be asked to join a study of bone health; 15 people in Gaborone and 15 in Francistown, will be asked to joing a study of how quickly the study medicine is absorbed, and 24 people from both sites will be asked to have a special interview about their experiences in the study. If we ask you to think about joining one of these studies, we will explain what the study is about first and then ask you if you want to join. Whether you decide to join or not, you can stay in the main study.

**Will being in the study benefit me?**

If you join the study, you will learn your HIV status and other things about your health. You will get education and counseling about ways to keep from getting HIV. You will be given free condoms (both the kind that men wear and the kind that women wear) and taught how to use them. If you have a health problem while you are in the study, we will help you get care.

**How will my information be protected?**

We will use a code number, not your name, on interview forms, blood and urine samples, we take for testing, and test results. The code number we give you will be used to keep track of all your records. Consent forms and information you give us to contact you will be kept locked up away from all the other study forms. Only a few study staff will be able to see the list with both names and code numbers in case we need to contact you. It is possible that US or Botswana government agencies that check the quality of studies will review your study file stored with the code number to be sure that we are doing the study correctly.

If we take pictures of conditions we find during a physical exam (like a rash), we will alter the photo so that you can’t be identified and store the photo on a computer so that only our staff can see it.

If you miss any study visits, a staff member will visit you at home to give you another appointment in the clinic. The staff member will come in a car that doesn’t mention the study. If you are not at home, they will not leave a message with other people at you house that would let them know you are in a study here.

**are there any risks to being in the study?**

The interview may make you a little uncomfortable when we ask about your sexual life. Blood drawing may cause some discomfort, bruising, or a small amount of bleeding. A few people get lightheaded when blood is drawn. If any test shows that you have gotten the HIV virus while you are in the study, it may be upsetting to you. We will help you if you get upset by giving counseling when you get your test result, and at later visits if you want.

If you get TDF/FTC, you might have mild symptoms like a mild upset stomach or gassiness. There is a small chance that it will affect your kidneys. We will check for that with your blood tests and will let you know if the tests show any problems. It is also possible that your bones will store less calcium. But in studies with HIV-positive people who took TDF/FTC with other medicines to fight HIV, when this happened it didn’t affect their health. If you are one of the first 100 people of your gender enrolled in Gaborone, we will x-ray your wrist, lower back, and hip every 6 months to check on your bone health.

If you get HIV infection while you are taking TDF/FTC, there is a very small chance that the virus might learn to fight off the medicine and grow even when the medicine is around (also called resistance). This might affect your treatment in the future. We will do an HIV test at every clinic visit so we can stop the study medication quickly if you get infected to try and prevent this. But if you do become infected, we will perform other tests which can tell whether the virus has become resistant to TDF/FTC. These results will be shared with your doctor and can help your doctor identify the best treatment for you.

If you have any bad side effects from the medicine you are taking or if you become infected with HIV, we will help you get the medical care you need at a government hospital or clinic.

Although we will do everything we know how to do to protect your privacy, there is a very small chance that information about you could become known to others. If we become aware that this may have happened, we will contact you to let you know. We ask that if you become aware that it may have happened, you let us know so we can fix any problem that leads to such a problem.

**Can I still be in the study if I get infected with HIV?**

If you get infected with HIV, we will not give you any more study medicine. But you can stay in the study and continue to have visits every month. That way you can see a counselor regularly and talk with the doctor to answer your questions about having HIV. You will get counseling about how to keep from passing HIV infection to others. You will get physical exams and treatment for any sexually transmitted infections we find. You will have the same interviews except those that are about study medicine. And we will do tests every six months to see how your body is coping with the infection.

**Is there any cost for being in the study?**

There is no cost to you for examinations, blood tests, or counseling. If you agree be in the study, we will give you 30 Pula (5.50 USD) for travel costs getting to the clinic and home. For the time and effort you will spend during your study visits, you will also get 15 Pula (2.70 USD) at the long visits every six months. You will get 10 Pula (1.80 USD) for the medium visits at 3 and 9 months. And you will get 5 Pula (0.90 USD) at the short monthly visits.

**What are my rights if i agree to be in the study**

You may refuse to answer questions or give blood or urine at any time during the study. If you have questions or concerns about the study, please call and speak to Dr. Poloko Kebaabetswe (390-1696).

We may need to stop the study medication if you have health problems that mean the medicine is not safe for you or if you are not able to do what is necessary to be a part of the study, for example if you cannot come regularly for study visits.

If you have any questions about your rights as someone in a research study, complaints about how you were treated in the study, or feel that you have been harmed during the study, please call and speak to Mrs. Shenaaz El-Halabi, Director of the Health Research Unit at the Botswana Ministry of Health (391-4467).

If you agree to be in the study, you will be agreeing to do several things, as best you can including:

- Come to the clinic for visits every month for at least 14 months
- Answer the questions asked at each visit
- Use condoms to protect yourself from HIV whenever you have sex
- Take the pills you are given every day
- FEMALES: Take pills or shots to prevent pregnancy
- Have blood be drawn at the first three monthly visits, then every 3 months
- Have oral HIV tests at each visit (unless you get HIV infection while you are in the study)
- Have a physical exam and give a urine sample every six months
- Have confidential home visits and phone calls made if you miss a study visit

***Ask participant if they wish to join the study***

**Statement of Consent**

I have read this consent form or had it read to me. All my questions have been answered. I have been told the purpose of the tenofovir study, what will be done, and the risks and benefits of the study.

[ ] I agree to join.

[ ] I do not agree to join.

__________________ ___________________ ___________________

Name Signature Date

______

*(thumbprint)

* in case the person is not able to read this form or sign their name, this attests that the consent form has been read and explained accurately by a member of the research staff, and that the person has affixed their thumbprint as consent.

__________________ ___________________ ___________________

Witness Name Signature Date

We are asking your permission for some leftover blood, cells, and genital fluids to be stored for future research and testing related to sexually transmitted diseases, including HIV. They may be stored for up to 10 years. Some of these tests may look at parts of your immune system that are inherited. Some tests may look at levels of medication in your blood or how your body responds to the medication. We do not yet know what tests may be done in the future. These tests will only be done after we receive approval from the ethics committees in the Ministry of Health and the Centers for Disease Control and Prevention. If knowing the test results would be helpful to you we will try to tell them to you or your doctor. If you wish to have your specimens removed from storage and destroyed in the future, please contact Dr. Poloko Kebaabetswe (390-1696).

[ ] I agree to have my leftover specimens stored for future testing.

[ ] I do not agree to have my leftover specimens stored for future testing.

__________________ ___________________ ___________________

Name Signature Date

______

*(thumbprint)

* in case the person is not able to read this form or sign their name, this attests that the consent form has been read and explained accurately by a member of the research staff, and that the person has affixed their thumbprint as consent.

__________________ ___________________ ___________________

Witness Name Signature Date

**Assigned Enrollment ID [__] [__] [__] [__] [__]**

⬜⬜⬜________________⬜⬜/⬜⬜/⬜⬜

Staff Administering Consent Date: dd mm yy

**TDF2 HIV Prevention Study**

**DEXA Substudy Oral Consent Script**

(FK 8.1)

**Introduction**

You have decided to join the TDF2 study. You are also eligible to be in the bone health substudy.

The goal of this smaller tudy is to learn if the combination medicine (TDF and FTC) changes the amount of calcium that gets stored in bones

In one study, when newborn monkeys were given extremely high doses of TDF, their bones became weak. In people who have HIV infection, their bones can lose calcium without any medication or when they take TDF. But it doesn’t seem to cause any problems with the overall health of their bones. For example, they don’t have broken bones more often than other people.

We want to check whether TDF and FTC cause any problems for people who don’t have HIV infection. So we are asking 200 people in Gaborone to have bone x-rays every six months.

If you join this bone health study, you will go to Gaborone Private Hospital for x-rays of your wrist, lower back, and hip. Your first x-ray with be in the next week, Then you will have x-rays every six months while you are in the study.

There is no risk to you. The x-rays are very mild and are not uncomfortable. If we find that your bones are not as strong as they should be, we will give you a vitamin to take.

There is no cost to you. If you agree to be join the bone health study, you will receive 30 Pula (7 USD) for travel costs getting to the Gaborone Private Hospital and back.

If you have questions or concerns about the bone health study that we can’t answer for you now, please call and speak to Dr. Poloko Kebaabetswe (390-1696).

If you have any questions about your rights as someone in a research study, complaints about how you were treated in the study, or feel that you have been harmed during screening, please call and speak to Mrs. Shenaaz El-Halabi, Director of the Health Research Unit at the Botswana Ministry of Health (391-4467).

If you want to join the bone health substudy, please understand that:

- It is your free choice to join or not
- If you decide not to join, you can still be in the TDF2 study
- If you decide not to join, you cannot join later

If you decide to join, you can leave the bone health study anytime and still stay in the TDF2 study

***Ask participant if they wish to join the bone health substudy today***

Did participant agree to be in the bone health substudy?

⬜ Yes [Indicate participation on the Clinical Questionnair (IN02), Q9]

⬜ No [Indicate refusal on the Clinical Questionnaire (IN02), Q9]

**Assigned Enrolled ID [__] [__] [__] [__] [__]**

⬜⬜⬜___________ ⬜⬜/⬜⬜/⬜⬜

Staff Administering Consent Date: dd mm

**TDF2 HIV Prevention Study**

**Parental/Guardian Screening Consent Form**

(FK 8.2)

**Introduction**

The Botswana Ministry of Health and the Centers for Disease Control and Prevention in the United States are doing a research study in Gaborone and Francistown.

- The first goal of this study is to learn if a combination medicine (TDF and FTC) works to prevent HIV infection.
- The second goal is to see if it is safe when used for a year or more by young people in Botswana.

This medicine is usually used along with other medicines to fight HIV in people who are already infected. In fact the Botswana Ministry of Health is now recommending that TDF and FTC should be used initially to treat people infected with HIV (but always in combination with other medicines). We think it might also work if taken by itself to prevent HIV infection. But we need to do a study to test if it works this way in young people here.

Because young people are often getting HIV infection in Botswana, we want to include young adults 18-20 years old in the study. Your son or daughter has said they might be interested in being in the study. So we want to describe the study and ask your permission for them to participate if we find they are eligible.

The study has two parts:

First we want to find out if your son or daughter is eligible to join the study. We would like to ask them to:

- Answer a few questions
- Have some counseling about HIV tests
- Decide whether to have a test today
- Allow us to take blood and urine samples for testing in the clinic today
- Have some counseling about how to lower their chances of getting or passing HIV
- Return to the clinic in 1-2 weeks

We call this process screening. We will also ask them if they want to be screened. Screening will only happen if you both agree to it. Please understand that:

- It is your free choice for them to be screened or not
- If you decide not to allow them to be screened, they will still receive all regular care they get now at any other clinics in the community

***Give the parent a copy of the screening consent form, allow them to read it over, and answer any questions they have about screening***

The second part of the study is for those who are found to be eligible during screening. We would ask them to join the study if your son or daughter:

- tests negative for HIV
- may be exposed sexually
- is otherwise very healthy

**Why are we doing this study?**

People who are having sex can catch sexually transmitted infections (STIs), including infection with HIV, the virus that causes AIDS. Using condoms is the only way we have to prevent this. But many couples do not use condoms or do not use them every time they have sex. So, we are trying to find new ways to help prevent HIV passing between people when they have sex. One of the new ways is for people who don’t have HIV infection to take medicines that fights the virus. The medicine we will be testing is a combination of tenofovir (also called TDF) and emtricitabine (also called FTC). They come in one pill that is usually used along with other medicines to fight HIV in people who are already infected. We think it might also work if taken by itself to prevent HIV infection but we haven’t proven that yet.

Because young people are often getting HIV infection in Botswana, we are looking for young people to join the study so we can test TDF/FTC and find out if it works for prevention. There will be 1200 youth in this study, some in Francistown, and some in Gaborone.

**What will they need to do if they join the study?**

We will assign each person to one of two groups. A computer tells us which group and everyone has an equal chance to be in either group. One group will get a pill with TDF/FTC, the medicines that may prevent HIV infection and that we want to test for safety. The other group will get a medicine that looks exactly the same but doesn’t have any effect. This is a placebo or “sugar pill”. Until the study is over, we won’t know, and the people in the study won’t know, who is taking which medicine. We do it this way so that we won’t let our hopes about the medicine influence the way we collect the information we need to see if the TDF/FTC is safe.

If they agree to be in the study, we will ask each participant to come for visits every month for at least 12 months (12 visits). Each month we will ask some questions about taking the pill, how healthy they feel, and their sexual behavior. And we will do an HIV test every month. Monthly for the first three months, and then once every three months after that, we will take a small amount of blood to see how the body is reacting to the pills. At one visit, we will take blood before and after they take their study medicine. Every six months, we will do a physical examination. We will also test for sexually transmitted infections. If we find any infections, we will treat them.

We will give each participant counseling about how to lower their chances of ever getting HIV infection. We will give them free condoms. This is important for two reasons. We don’t know who is getting TDF/FTC. Also we don’t know yet if it works to protect people from getting HIV. In addition to using condoms, we will require all young women in the study to use pills or take shots to prevent pregnancy,

If they are one of the first 100 people of their gender enrolled in Gaborone, we will ask them to go to a local radiology clinic for an x-ray of their wrist, lower back, and hip every six months.

**Will being in the study benefit him or her?**

If they join the study, your son or daughter will learn their HIV status and other things about their health. They will get education and counseling about ways to keep from getting HIV. They will be given free condoms (both the kind that men wear and the kind that women wear). They will be taught how to use them. If they have a health problem while they are in the study, we will help them get care.

**How will their information be protected?**

We will use a code number, not their name, on interview forms, blood and urine samples, we take for testing, and test results. The code number we give them will be used to keep track of all their records. Consent forms and information they give us to contact them will be kept locked up away from all the other study forms. Only a few study staff will be able to see a list with both names and code numbers in case we need to contact them. If we take pictures of conditions we find during a physical exam (like a rash), we will alter the photo so that they can’t be identified. We will store the photo on a computer so that only our staff can see it.

If they miss any study visits, a study staff member will visit them at home to give them another appointment in the clinic. The staff member will come in a car that doesn’t mention the study. If they are not at home, they will not leave a message with other people at their house that would let them know your son or daughter is in a study here.

**are there any risks to being in the study?**

The interview may make them a little uncomfortable when we ask about their sexual life. Blood drawing may cause some discomfort, bruising, or a small amount of bleeding. A few people get lightheaded when blood is drawn. If any test shows that they have gotten the HIV virus while they are in the study, it may be upsetting to them. We will help them if they get upset by giving counseling when they get your test result and at later visits if they want.

If they get TDF/FTC, they might have mild symptoms like a mild upset stomach or gassiness. There is a small chance that it will affect their kidneys. We will check for that with blood tests and will let them know if the tests show any problems. It is also possible that their bones will store less calcium. But in studies with HIV-positive people who took TDF/FTC with other medicines to fight HIV, when this happened it didn’t affect their health.

If they get HIV infection while they are taking TDF/FTC, there is a small chance that the virus might learn to fight off the medicine and grow even when the medicine is around (also called resistance). This might affect their treatment in the future. We will do an HIV test at every clinic visit so we can stop the study medication quickly if they get infected to try and prevent this. But if you do become infected, we will perform other tests which can tell whether the virus has become resistant to TDF/FTC. These results will be shared with your doctor and can help your doctor identify the best treatment for you.

If they have any bad side effects from the medicine they are taking or if they become infected with HIV, we will help them get the medical care they need at a government hospital or clinic.

Although we will do everything we know how to do to protect their privacy, there is a small chance that information about them could become known to others. If we become aware that this may have happened, we will contact them to let them know.

**Can they still be in the study if they get infected with HIV?**

If they get infected with HIV, we will not give them any more study medicine. But they can stay in the study and continue to have visits every month. That way they can see a counselor regularly and talk with the doctor to answer their questions about having HIV. They will get counseling about how to keep from passing HIV infection to others. They will get physical exams and treatment for any sexually transmitted infections we find. They will have the same interviews except those that are about study medicine. And we will do tests every six months to see how their body is coping with the infection.

**Is there any cost for being screened or being in the study?**

There is no cost to them for examinations, blood tests, or counseling. If they agree be in the study, we will give them 30 Pula (5.50 USD) for travel costs getting to the clinic and home. For the time and effort they will spend during study visits, they will also get 15 Pula (2.70 USD) at the long visits every six months. They will get 10 Pula (1.80 USD) for the medium visits at 3 and 9 months. And they will get 5 Pula (0.90) at the short monthly visits.

If you came to the clinic today to learn about the study, we will give you 30 Pula for travel.

**What are their rights if they agree to be screened or be in the study?**

They may refuse to answer questions or give blood or urine at any time during the study. If you or they have questions or concerns about the study, please call and speak to Dr. Poloko Kebaabetswe (390-1696).

We may need to stop the study medication if they have health problems that mean the medicine is not safe for them or if they are not able to do what is necessary to be a part of the study, for example if they cannot come regularly for study visits.

If you or they have any questions about the rights of someone in a research study, complaints about how your son or daughter was treated in the study, or feel that they have been harmed during the study, please call and speak to Mrs. Shenaaz El-Halabi, Director of the Health Research Unit at the Botswana Ministry of Health (391-4467).

**What are we asking you to agree to?**

By giving your consent for your son or daughter to be screened, you will be giving permission for them to:

- Be interviewed
- Make their own decision about having an HIV test today
- Have blood drawn for health tests
- Be counseled about their HIV risks
- Be given condoms
- DAUGHTERS: Be given pills or shots to prevent pregnancy
- Make their own decision about joining the main study and substudies if they are eligible
- Keep information about their health private

If you give your consent for your son or daughter to be screened, we will counsel them to discuss their health and study experiences with you. But we will not tell you anything about:

- what they say during the interview
- what their HIV test results are
- DAUGHTERS: whether your daughter is pregnant
- whether they are eligible for the study
- what decision they make about joining the study.

***Ask parent if they consent for their son or daughter to be screened***

**Statement of Consent**

I have read this consent form or had it read to me. All my questions have been answered. I have been told the purpose of screening and of the TDF/FTC study, what will be done, and the risks and benefits of the study. I agree that my son or daughter may decide for themselves whether to participate in screening, have an HIV test, or join the study. I understand that all information about my son or daughter’s participation in screening or the study will be kept private and not shared with me by study staff.

__________________ ___________________ ___________________

Name Signature Date

______

*(thumbprint)

* in case the person is not able to read this form or sign their name, this attests that the consent form has been read and explained accurately by a member of the research staff, and that the person has affixed their thumbprint as consent.

__________________ ___________________ ___________________

Witness Name Signature Date

**Assigned Screening ID [__] [__] [__] [__] [__]**

⬜⬜⬜________________⬜⬜/⬜⬜/⬜⬜

Staff Administering Consent Date: dd mm yy

**GUARDIANSHIP STATEMENT**

I, __________________________ confirm that I am acting as adult guardian

*(PRINT full name of guardian)*

for _________________________

*(PRINT full name of young adult minor)*

by providing financial assistance, emotional support, and supervision of activities as would be provided by his/her biological parents if they were able and available.

His/her biological parents are:

🞎 deceased

🞎 living > 1 hours travel distance from the study site and unable to travel to the clinic

I also confirm that I am related to him/her as follows *(check only one)*:

🞎 Aunt *(sister of his/her biological parent)* 🞎 Uncle *(brother of his/her biological parent)*

🞎 Grandmother *(mother of his/her biological parent)* 🞎 Grandfather *(father of his/her biological parent)*

🞎 Sister *(daughter of his/her biological parent)* 🞎 Brother  *(son of his/her biological parent)*

🞎 OTHER *(Specify)* ___________________________________________________________________

It has been explained to me that I may be contacted by the Health Research Unit in the Botswana Ministry of Health to confirm my guardianship role as reported on this form. For that reason, the following contact information is provided:

Omang: _____________________

Postal Address: _________________________________________________________

Physical Address: _________________________________________________________

Cell Phone Number: _____________________ *(call and confirm immediately)*

Work Phone Number: _____________________ *(call and confirm immediately)*

Home Phone Number: _____________________ *(call and confirm immediately)*

_________________ ___________________ __ __ __ _______ _______________

*Signature of Guardian Signature of Participant Staff ID and Initials Date(dd/mm/yy)*

**TDF2 HIV Prevention Study**

**Qualitative Substudy Interview Consent Form**

(FK 8.8)

You are in a study to see if taking a drug may help prevent HIV. The study is being done by BOTUSA, a project with the Ministry of Health here in Botswana and the Centers for Disease Control and Prevention in the United States (also called CDC).

We are inviting you to have three extra interviews.The purpose of these interviews is to ask about your opinion of the study and your experience with study procedures. We will use what you tell us to improve the way the study works.

## Your part in the research

If you agree, you will have three interviews, one interview every three months. We will ask about your experience in the study, how easy or difficult it was to take a pill every day and follow other study procedures, how being in the study may or may not have affected your sexual behavior, how you feel about research in general, and what thoughts you have for how to improve the study. You can refuse to answer any questions.

The interviews will be done in private. Each interview will take about one and one-half hours of your time. The interview will be recorded using an audio recorder. If you have any concerns about recording the interview, please let me know. However, recording of interviews is a requirement for participation in the three interviews. We need to record each interview so that we can remember exactly what you say in your own words.

## Possible risks & benefits

There are only mild risks of doing the interview. Some of the interview questions are about personal issues like your sexual behavior. These things might be mildly embarrassing to you. We will make every effort to protect your privacy. We will assign a code number to the interview and your name will not be taken. The interview recordings and notes will be stored in a locked cabinet. Only the study staff will be able to use them. We will destroy the recordings when the research is complete.

There are few benefits to you for taking part in the interviews. What we learn in this research may help people in the study by helping us to improve the way the study is run. And you may find that you enjoy discussing some of the issues we will bring up with you.

## If you decide not to be in the research

You are free to agree to the interviews or not. Your decision will not affect your participation in the TDF2 trial.

## Compensation

These interviews will usually not occur on the same day as a regular TDF2 study visit. So we will provide 30 Pula (5.50 USD) for travel to and from the clinic for each interview. We will also provide you with 10 (1.80 USD) Pula for the time and effort you spend on each interview. So for each interview, you will receive 40 Pula (7.30 USD).

## If you have a problem or have other questions

If you have a problem that you think might be related to taking part in these interviews, please feel free to call Dr. Poloko Kebaabetswe (390-1696). If you have any questions about your rights as someone in a research study, complaints about how you were treated in the study, or feel that you have been harmed during screening, please call Mrs. Shenaaz El-Halabi, Director of the Health Research Unit at the Botswana Ministry of Health (391-4467).

Do you agree to do the three interviews?

**□ Yes** [SIGN STATEMENT OF CONSENT BELOW]

□ **No** [ASK WHY THEY DECLINED TO BE INTERVIEWED AND RECORD IN THE SPACES BELOW (DO NOT INSIST OR PROBE). THANK THEM FOR THEIR TIME.]

NOTES ON REFUSAL TO BE INTERVIEWED:

__________________________________________________________________________________________________________________________________________________________________________________________________________________________________________**________________________________________________________________________________________________________________________________________________________________________________________________________________________**

**Statement of Consent**

I have read this consent form or had it read to me. All my questions have been answered. I have been told the purpose of the interview study, what will be done, and the risks and benefits of the study. I agree to be in the interview study.

__________________ ___________________ ___________________

Name Signature Date

______

*(thumbprint)

* in case the person is not able to read this form or sign their name, this attests that the consent form has been read and explained accurately by a member of the research staff, and that the person has affixed their thumbprint as consent.

__________________ ___________________ ___________________

Witness Name Signature Date

**Assigned Substudy ID [__] [__] [__] [__] [__]**

**TDF2 Participant ID**  **[__] [__] [__] [__] [__]**

⬜⬜⬜________________⬜⬜/⬜⬜/⬜⬜

Staff Administering Consent Date: dd mm yy

**TDF2 HIV Prevention Study**

**PK/PD Consent/Assent Form**

(FK)

**Introduction**

You are participating in the TDF2 study being conducted by BOTUSA, a partnership between the Botswana Ministry of Health and the Centers for Disease Control and Prevention in the United States. This study is being done to see if taking a combination medicine (TDF and FTC) works to prevent HIV infection.

To better understand the results of the TDF2 study, we need to learn more about how quickly TDF/FTC are absorbed into the body and how much stays in the blood and cells over time. We would like you to think about joining a special small study about this. It will last only one day.

**What do I need to do if I agree to be in the small study?**

If you decide to join this small study, we will ask you to:

- Not eat or drink after 7 PM on the day before you come for this study
- On the day of the study:
- Come to the clinic early in the morning
- Not take your study pill until you come to the clinic
- Not eat or drink for 2 hours after you take your study pill
- Stay at the clinic all day or return several times during the day
- Have smallamounts of blood drawn five times during the day

Just before you take your study pill

1 hour after your pill

2 hours after your pill

4 hours after your pill

8 hours after your pill

Altogether we wil take a little less than 6 tablespoons of blood (80 ml). Removing that amount of blood is very safe for a healthy person. It’s about 1/6 of what would be taken during a blood donation drive. Drawing blood several times will let us see how quickly the medicine builds up in your blood and when the amount starts to go down. That information will let predict how much medicine is in your blood and cells during different times of day from one day’s dose until the next day’s dose.

**Will being in the small study benefit me?**

If you agree to be join, there will not be any personal benefit to you. It will help us understand the results of the TDF2 study better.

**How will my information be protected?**

We will use a code number, not your name, on blood samples we take for testing, and test results. This is the same private code number we use for all your TDF2 study records.

**are there any risks to being in this small study?**

Blood drawing may cause some discomfort or bruising. We will use the smallest needles possible to help reduce the discomfort.

**Is there any cost for being in this small study?**

There is no cost to you. If you agree to join, you will receive the 30 Pula (5.50 USD) for travel costs as you do for all TDF2 visits. In addition, at the end of the day, after all five blood draws, you will receive 100 Pula (18.20 USD) for the time and discomfort and to cover your meal costs during the day.

**What are my rights if I agree to be in this small study?**

You may withdraw from this small study at any time. You will still receive your travel reimbursement. If you have questions or concerns about this small study, please call and speak to Dr. Poloko Kebaabetswe (390-1696).

If you have any questions about your rights as someone in a research study, complaints about how you were treated in the study, or feel that you have been harmed, please call and speak to Mrs. Shenaaz El-Halabi, Director of the Health Research Unit at the Botswana Ministry of Health (391-4467).

***Ask participant if they wish to join the PK/PD substudy***

**Statement of Consent**

I have read this consent form or had it read to me. All my questions have been answered. I have been told the purpose of the small study, what will be done, and the risks and benefits of joining. I agree to join.

__________________ ___________________ ___________________

Name Signature Date

_______

*(thumbprint)

* in case the person is not able to read this form or sign their name, this attests that the consent form has been read and explained accurately by a member of the research staff, and that the person has affixed their thumbprint as consent.

__________________ ___________________ ___________________

Witness Name Signature Date

We are asking your permission any leftover blood and cells for future research and testing related to medication levels. They may be stored for up to 10 years. We do not yet know what tests may be done in the future. These tests will only be done after we receive permission from the ethics committees in the Ministry of Health and the Centers for Disease Control and Prevention. You will not know the results of these future research tests. The test results will not affect your health since they will be done a long time after the blood is collected from you. If you want to have your specimens removed from storage and destroyed in the future, please contact Dr. Poloko Kebaabetse at BOTUSA (390-1696).

[ ] I agree to have my leftover specimens stored for future testing.

[ ] I do not agree to have my leftover specimens stored for future testing.

__________________ ___________________ ___________________

Name Signature Date

______

*(thumbprint)

* in case the person is not able to read this form or sign their name, this attests that the consent form has been read and explained accurately by a member of the research staff, and that the person has affixed their thumbprint as consent.

__________________ ___________________ ___________________

Witness Name Signature Date

**Assigned Screening ID [__] [__] [__] [__] [__]**

⬜⬜⬜________________⬜⬜/⬜⬜/⬜⬜

Staff Administering Consent Date: dd mm yy

# INFORMED CONSENT FOR HIV TESTING

# I_______________________________________ (participant name)

Have received counselling with regard to HIV and AIDS. I have considered the implications that taking the HIV antibody test may have for my life and have decided to have the test.

I have been told that that the results of this test will be recorded in my research record without recording my name.

_________________________ ____________________________

Signature Date

__________________________ ____________________________

Counsellor Date

_________

*(thumbprint)

* in case the person is not able to read this form or sign their name, this attests that the consent form has been read and explained accurately by a member of the research staff, and that the person has affixed their thumbprint as consent.

__________________ ___________________ ___________________

Witness Name Signature Date

**Assigned Screening ID [__] [__] [__] [__] [__]**

⬜⬜⬜________________⬜⬜/⬜⬜/⬜⬜

Staff Administering Consent Date: dd mm yy

**CONFIDENTIAL CLIENT REFERRAL FORM**

I _____________________________________ (participant name)

give consent to ___________________________________ (study staff member)

to release my: HIV antibody test results Yes No

HIV viral load test results Yes No

CD4 count Yes No

HIV resistance testing results Yes No

Participation in the TDF2 study Yes No

to _____________________________________________ (care provider name or site)

for purposes of clinical care and follow-up.

___________________________________ ___________________________

Signature Date

This is to verify that ______________________________________ (participant name)

has been counseled and tested positive for HIV infection in our study clinic

on ______________________ (date)

His/her test results were:

HIV viral load _____________ copies per ml Undetectable

CD4 count ______ cells/µl

He/she is being referred to you for ongoing follow-up care.

___________________________________ ___________________________

Signature of Study Site Physician Date

**AUTHORIZATION FOR RELEASE OF CONFIDENTIAL MEDICAL INFORMATION**

I _________________________________________ (participant name)

give permission for _______________________________________ (name of clinic or doctor)

to provide information in my medical records about recent illnesses, diagnoses, and any treatment I may have received during the period

from to __________________________

(date of symptom or illness onset) (today)

I understand that purpose of providing this information is research.

I understand that I can take back my permission in writing at any time. The information recorded from my medical records will not be given to any person other than study staff without first getting my additional consent in writing.

Signature Date signed

Printed name

Signature of study physician Date signed

___________

*(thumbprint)

* in case the person is not able to read this form or sign their name, this attests that the consent form has been read and explained accurately by a member of the research staff, and that the person has affixed their thumbprint as consent.

__________________ ___________________ ___________________

# Witness Name Signature Date

**Assigned Screening or Enrollment ID [__] [__] [__] [__] [__]**

**AUTHORIZATION FOR RELEASE OF CONFIDENTIAL MEDICAL INFORMATION**

I _________________________________________ (participant name)

give permission for _______________________________________ (name of clinic or doctor)

to provide information in my medical records about recent illnesses, diagnoses, and any treatment I may have received during the period

from to __________________________

(date of symptom or illness onset) (today)

I understand that purpose of providing this information is research.

I understand that I can take back my permission in writing at any time. The information recorded from my medical records will not be given to any person other than study staff without first getting my additional consent in writing.

Signature Date signed

Printed name

Signature of study physician Date signed

___________

*(thumbprint)

* in case the person is not able to read this form or sign their name, this attests that the consent form has been read and explained accurately by a member of the research staff, and that the person has affixed their thumbprint as consent.

__________________ ___________________ ___________________

# Witness Name Signature Date

**Assigned Screening or Enrollment ID [__] [__] [__] [__] [__]**

**B. RECRUITMENT AND PARTICIPANT EDUCATION MATERIALS**

1. **Study Information Brochure**
2. **Recruitment Radio and TV Scripts and Print Ads Proposed (PDF file)**
3. **Flyers and Banner**
4. **Screening Multimedia Trial Education Application (PPT file)**
5. **Study Education Booklet**
6. **Study Medication Handout**
7. **PK/PD Visit Instruction Sheet**

**Who is doing this study?**

The study is being done by the BOTUSA Project, a collaboration between the Botswana government and the U.S. Centers for Disease Control and Prevention.

The Health Research Development Committee in the Ministry of Health and an ethics committee at the U.S. Centers for Disease Control and Prevention will review and approve the study procedures before we start and will check during the study to see that participants are safe. An international committee of scientists who are not part of this study will also look at the information we collect to see that participants are safe.

In addition, advice is given to the study team by a national reference group, community advisory groups in Francistown and Gaborone, and participant advisory groups in the two cities.

## For more information about the TDF2 HIV Prevention Study

## please contact:

**HIV Prevention Research Section**

**The BOTUSA Project**

P.O. Box 90 Gaborone

Dr. Poloko Kebaabetswe: 390-1696

Francistown Clinic: 241-0646

Gaborone Clinic: 319-1375


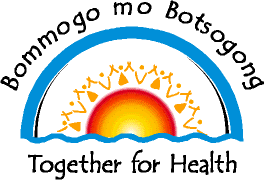


#### The BOTUSA Project

P.O. Box 90

Gaborone, Botswana

Phone (267) 390-1696

Fax (267) 397-3117

Basic Facts About:

The Botswana TDF2 HIV Prevention Study


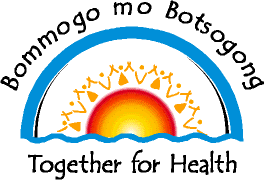


**Why do this study with young adults in Botswana?**

In Botswana, many young women and men are getting infected with HIV, the virus that causes AIDS. There are several ways to protect against getting or giving HIV including: people who have tested negative being faithful to each other, using condoms correctly every time a person has sex, and choosing not to have sex for a while. But for many young adults, it is hard to behave safely all the time. And every time they behave unsafely, they take a chance on getting or giving HIV. In Botswana, sexually-active men and women younger than 30 are getting infected more rapidly than people at other ages so it is especially urgent to find additional ways to prevent HIV infection among them. Having more effective prevention tools will help to achieve the Vision 2016 goal of no new infections in Batswana youth.

**What is Pre-Exposure Prophylaxis?**

Pre-exposure prophylaxis is a medical term for giving antibiotics or vaccines before people come in contact with an infectious agent to try and keep them from getting infected when the contact occurs. We think this might also work for HIV prevention because:

- giving newborns an antiretroviral before they are born seems to help protect them from virus they are exposed to during birth
- giving a single antiretroviral to health care workers hours to days after they are exposed to HIV by a prick from an contaminated needle will protect most against getting infected
- giving antiretrovirals to monkeys before exposing them to SIV (the monkey form of HIV) protects most against getting infected

**What are Tenofovir (TDF) and Emtricitabine (FTC)?**

TDF and FTC are antiretroviral medicines that come together in a single pill. It is used, in combination with other antiretrovirals, to treat people who have HIV infection. It prevents HIV from growing and spreading in the body.

**Why do we think it is safe?**

TDF/FTC is widely used for treatment in the United States, Europe, and in some African countries including Botswana. For treatment of HIV infection, it is taken once a day, doesn’t cause many side effects, and is very slow to develop resistance. It has been tested in a few people without HIV infection for a short period of time and it appeared to be safe for them.

**How will the study be done?**

We will invite 1200 men and women in Francistown and Gaborone to join the study. They must be citizens of Botswana, 18- 29 years old, already sexually active, and healthy. The study will be carefully explained and those people who fully

understand the study will be asked to give their consent to be in the study. We will help everyone in the study to reduce their risk of getting HIV infection by providing frequent counseling, diagnosis and treatment for sexually transmitted infections, and free male and female condoms. In addition, everyone will be given pills to take daily. Half of the people will be randomly assigned to take TDF/FTC and half will be randomly assigned to take a pill that looks and tastes just like TDF/FTC but has no medicine in it (a placebo). Study participants and staff will not know who is taking which pill. That way the hopes and fears we have about TDF/FTC will not affect what we see during the study.

We will see study participants once a month for at least 12 months so that we can see how they feel, how well they are doing in taking a pill every day, and how their sexual behavior may be changing. At each visit, we will do an HIV test and a pregnancy test for women. At some visits we will also do physical examinations and blood tests to check how TDF/FTC and placebo pills may be affecting people in the study. We will stop the pills if a study participant becomes infected or pregnant or there is evidence of developing health problems on the blood tests or exams.

Since often we cannot get people to use condoms every time they have sex, we expect that some people in the study will put themselves at risk from time- to-time and become exposed to HIV. At the end of the study, we will compare how many HIV infections occurred in those who took TDF/FTC and those who took placebo when they didn’t use condoms or didn’t use them correctly.

**Recruitment Radio and TV Scripts and Print Ads Proposed**

**See “PSA” PDF File**

*(protocol pages X-Y if you want to include in a printed copy of the protocol)*

Flyer 1


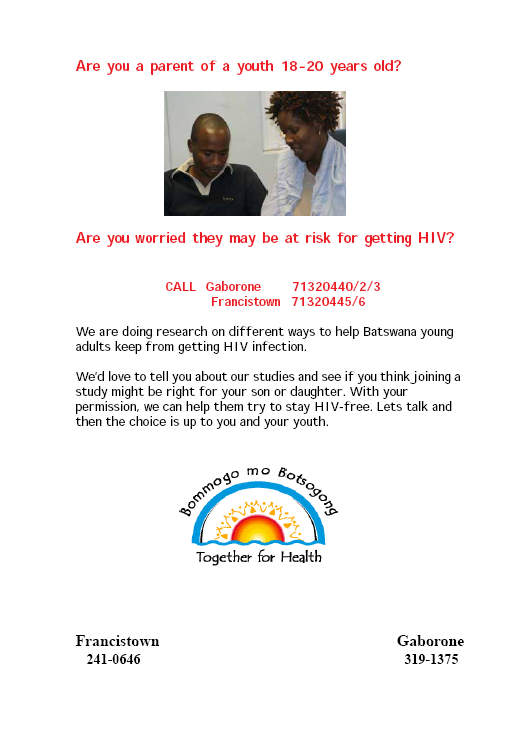


Flyer 2


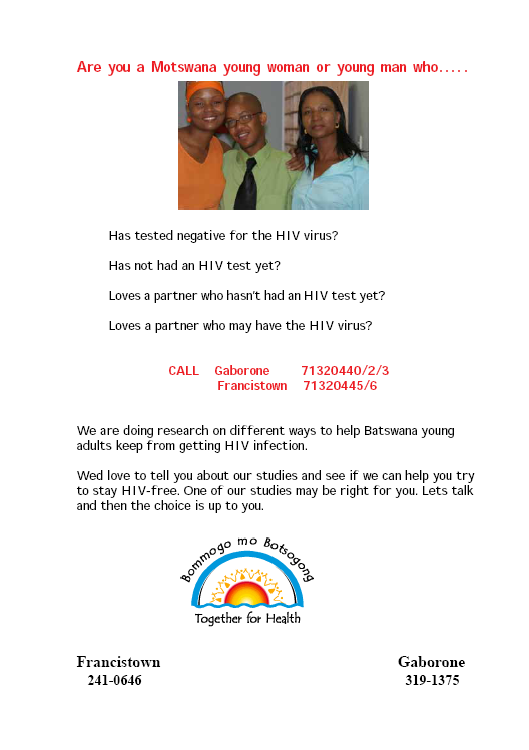


Table/Booth Banner


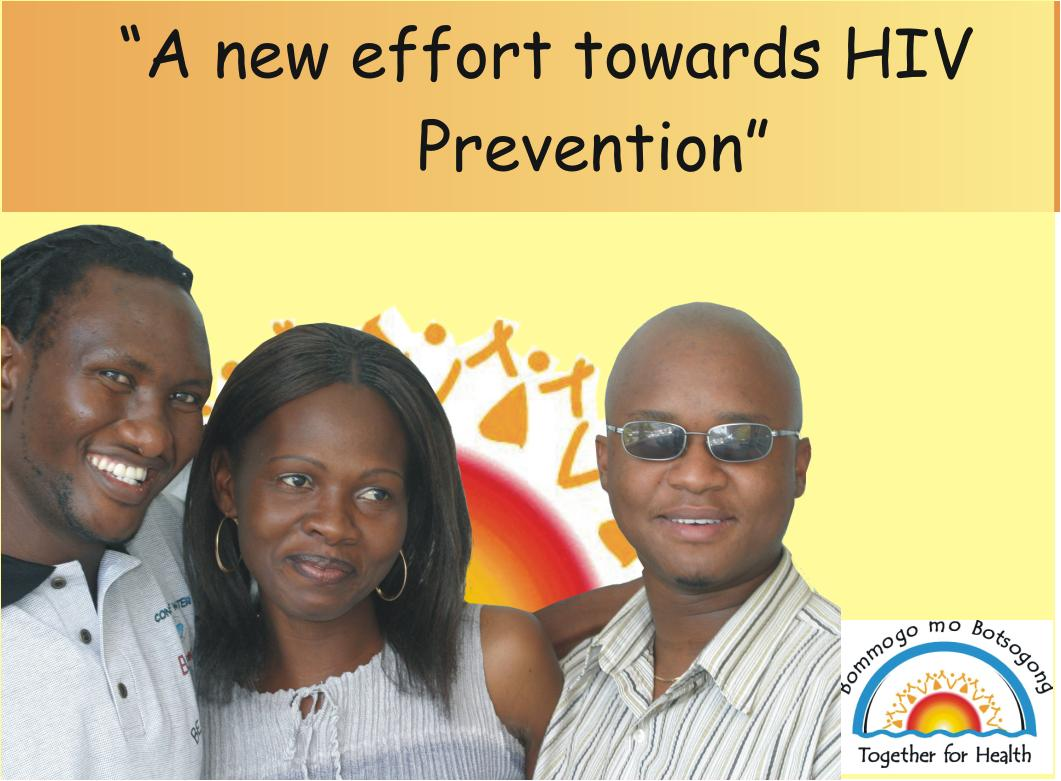


**Screening Multimedia Trial Education Application**

**See “TDF2 Learning Demo” PPT File**

*(not paged electronically because export to Word yields a 200 MB file)*

**Cover Page**

**TDF2 HIV Prevention Study**


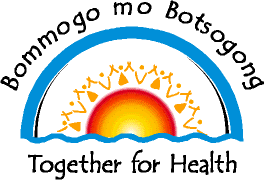


**An activity of the BOTUSA Project**

**A collaboration of the U.S. Centers for Disease Control and Prevention**

**And the Government of Botswana**

Page 2

**Research Clinic Sites and Contact Information**

**Francistown**

Plot 2806

Clinic Phone: 241-0646

**Gaborone**

Plot 725

Clinic Phone: 319-1375

Page 3

**TDF2 HIV Prevention Study**

**Why are we doing this study?**

- **To double-check that TDF and FTC are safe for Batswana women and men to use**
- **To see if taking TDF and FTC (medicines that fights HIV) can help prevent Batswana from getting HIV infection**

**This booklet will give you more information about the study.**

**If you have more questions about the study, please call us or come to visit us.**

Page 4

**Background**

**HIV/AIDS is a serious problem in Botswana,**

**especially for young adults, both women and men.**

- **More than one in every three Batswana women between the ages of 15 and 49 already has HIV infection**
- **Almost one in every four Batswana men between the ages of 15 and 49 already has HIV infection**
- **Most Batswana don’t know if they have the HIV virus because they haven’t had an HIV test yet.**

**HIV is the virus that causes the illness AIDS.**

- **In Botswana, HIV is usually spread between women and men during sex**
- **HIV infection doesn’t make you sick right away but damages your body over several years.**
- **When the immune system of the body is damaged badly, you can’t fight off common sicknesses. That weakness of the immune system is called AIDS.**
- **There is treatment that helps people infected with HIV to stay healthy longer but it doesn’t cure the infection for good.**
- **Preventing people from getting HIV infection is much better than treating people after they have it.**

Page 5

**How to Avoid Getting HIV Infection**

**Right now,**

**the only ways to keep yourself from getting HIV infection are:**

- **Abstain Choose not to have sex yet**

**or**

**Stop having sex for now**

- **Be faithful You and your partner only have sex with each other**

**and**

**Both of you have been tested and are free of HIV**

- **Condomise You and your partner always use condoms**

**Every time you have sex**

**It is very hard for many people to do these things all the time**

- **So many people who can’t follow ABC all the time, get infected with HIV**
- **We need other ways to help prevent HIV infection when people don’t follow ABC**
- **Together with ABC, a backup method would help more people avoid getting HIV infection**

Page 6

**What are TDF and FTC?**

**TDF (Tenofovir Desiproxil Fumarate)**

**FTC (Emtricitabine)**

- **TDF and FTC are medicines that fights the HIV virus very well**
- **A single pill of TDF with FTC is taken once a day**

**Why do we think TDF and FTC will help prevent HIV infection?**

- **In studies with animals and in laboratories, TDF and FTC can prevent infection with HIV and viruses like HIV**
- **But things that work in animals don’t always work in people**

**Why do we think TDF/FTC will be safe for Batswana to use?**

- **TDF and FTC are being used together to fight HIV in people who are already infected but always in combination with other medicines**
- **TDF and FTC are used to treat Africans, Americans, and others around the world**
- **In fact the Botswana Ministry of Health is now recommending that TDF and FTC should be used initially to treat people infected with HIV (but always in combination with other medicines).**
- **TDF and FTC don’t cause many symptoms when people with HIV infection use both medicines together**
- **A few people without HIV infection have taken TDF and FTC and they didn’t have many symptoms either**
- **Since people without HIV infection are healthier than those with HIV**

**we think it will be safe for Batswana without HIV infection to take TDF and FTC**

Page 7

**How TDF and FTC Might Work**

How does HIV infect you?

| - **HIV is in fluids that get shared during sex without a condom** | |
| --- | --- |
| - **HIV is in fluids that get shared during sex without a condom** | |
| - **HIV attaches itself to cells that are part of your body and gets carried into your body.** |  |
| - **In your body, the virus makes new copies of itself** |
| - **Those new copies spread to other nearby cells** |
| - **Eventually you have cells carrying the virus throughout your body** |

How could TDF and FTC prevent HIV infection?

| - **When you take a study pill with TDF and FTC in it, the medicine goes into your blood and is carried to cells throughout your body** | |
| --- | --- |
| - **When HIV enters a cell that has medicine in it, the medicine stops the virus from making new copies of itself** |  |
| - **Because the HIV can’t make new copies, it can’t spread to other cells** |
| - **When the cell carrying HIV dies, the HIV dies too** |
| - **Because the HIV couldn’t spread, you don’t get HIV infection** | |
| - **This is called Pre-Exposure Prophylaxis, or PrEP for short**   - **Pre = before**   - **Exposure = coming in contact with HIV**   - **Prophylaxis = medicine to prevent illness** | |

Page 8

**Why TDF and FTC Might Not Work**

**How could TDF and FTC fail to prevent HIV infection?**

- **If there is not enough medicine in the blood**
- **If some cells don’t have enough medicine in them to block HIV**
- **If HIV has learned how to get around the medicine and grow anyway**

**(this is called resistance)**

Page 9

**How TDF/FTC Will Be Tested**

**TDF/FTC and Placebo**

- **We will ask Batswana women and men who do not have HIV infection but are having sex regularly to join the study**

- **People who join will be divided into two groups**
- **One group will get a pill that has TDF and FTC in it.**

**This pill is called the study medicine.**

- **One group will get a pill that doesn’t have TDF and FTC in it.**

**This pill is called a placebo or sugar pill and it looks and tastes the same as the study medicine**

- **No one at the study clinic will know which people get the study medicine and which people get the placebo pill**
- **At the end of the study, we will compare the two groups to see if there are any differences in their health, the symptoms they report, and the number who get HIV infection while they are in the study**

**Why use a placebo?**

- **We all want the study medicine to work and to be safe**
- **Sometimes if you want something enough, it can effect the way you look at things and you see what you want to see**
- **Since people at the study clinics won’t know who is getting which pill, we can compare results in the study without worrying that we are letting our hopes influence what we see**

Page 10

**How People Will Be Assigned To Groups**

**It will be random**

- **A computer will be used to decide in advance, which study ID number will get which pill**
- **The study ID numbers will be put on the right bottles and shipped to Botswana**
- **Whenever someone joins the study, the next study ID number will be assigned to them and that person will always get the pills with that number on them**
- **At the end of the study, we will learn which numbers were assigned to study medicine and which were assigned to placebo**

Group Z

**1**

**6**

Group A

ID 1

ID 2

ID 3

ID 4

ID 5

ID 6

**Everyone has a equal chance of being in the Study Medicine or Placebo group**

Page 11

**Who Can Be In The TDF2 PrEP Study**

**You can be in the study if…**

- **You are a citizen of Botswana**
- **You are 18-29 years old**
- **You are healthy**
- **You live near Gaborone or Francistown**
- **You are willing to learn about the study**
- **You are willing to have an HIV test**
- **You can show that you understand the study and what we would be asking you to do if you join it.**
- **You agree to join the study**

**You cannot be in the study if…**

- **You are already infected with HIV**
- **You are pregnant or breastfeeding**
- **You are a women who is not willing to prevent pregnancy while you are in the study**

Page 12

**Steps To Joining The Study**

**First Screening Visit**

- **We tell you all about the study**
- **To see if you can be in the study**
  - **We ask you some questions to see if you can be in the study**
  - **We ask your permission for HIV**
  - **We take some blood and urine for laboratory tests**
- **We give you your HIV test results**
  - **And counseling about how to reduce your risks**
  - **And referrals for medical care and support if you test positive**
- **We treat you for any sexually transmitted infections (STIs) we find**
- **We give you an appointment to come back in 1-2 weeks**
- **We will take your photo or ask you to give us a photo to be sure that only you get your test results at other study visits**

**Second Screening Visit**

- **We tell you the results of all your laboratory tests**
- **We ask you some questions to see if you understand the study**
- **We determine if you are eligible for the study**
- **If you are eligible, we will ask you to join the study**

Page 13

**What Happens When In The Study**

**First Study Visit**

- **This visit can be on the same day as your second screening visit**
- **You will be asked to sign an agreement to be in the study**

**This is called an “informed consent”**

- **You will be assigned to a pill**
- **You will be given enough pills to last until your next visit**
- **You will get a diary for you to make notes when you take your pills**
- **You will be counseled about how to take your pills**
- **You will have an interview**
- **If you are in the first 100 people of your gender enrolled in Gaborone, we will ask you to have an x-ray of your wrist, hip, and lower back at the Gaborone Private Hospital so we can check the thickness of your bones.**
- **You will be given appointments to come back to the clinic every month for at least a year**

Page 14

**What Happens When In The Study**

**Every Month at Your Follow-up Visit**

- **We will get back any leftover pills and give you new pills**
- **We will look at your diary and discuss any problems you have taking the pill every day**
- **We will ask you how you are feeling**
- **We will do an HIV test on fluid from your mouth and tell you the results**

**At Some Follow-up Visits**

- **Every three months we will interview you**
  - **Sometimes with a person asking the questions**
  - **Sometimes with a computer asking the questions**

**(we will teach you how to use the computer to answer)**

- **Every three months we will take a blood sample from your arm**
- **Every three months we will talk with you about how to lower your chances of getting HIV infection**
- **Every six months we will do a physical examination and if you join the study in Gaborone, we may ask you to have an x-ray of your wrist, hip, and lower back to check your bone health**

Page 15

**What We Do With You In the Study**

**Interviews**

- **We will interview you and ask about**
  - **What you think about the study and the pills**
  - **How you are feeling physically**
  - **Your sexual behavior during the study**

**Counseling**

- **We will give you private counseling to help you see what you are doing that may cause you to get HIV infection and how to change that behavior**
- **We will talk with you about how to take the pills and help you find ways to take them regularly if you are having problems**
- **If you get HIV infection, we will talk with you privately about how to get care for your infection, what to expect, and how to get support from your family and friends**

**Physical Exams**

- **You will have genital exams to see if you have any sexually transmitted infections. If we find any STIs, we will give you free treatment**

**Specimens**

- **We will take oral fluid swabs and fingerprick blood samples to do HIV tests**
- **We will take urine specimens to test for pregnancy and STIs**
- **Sometimes we will take swabs from your private parts to test for STIs**
- **Sometimes we will take blood specimens from your arm to test for HIV, STIs, and levels of medicine in your blood**

Page 16

**What We Ask From You In the Study**

**If you join the study, it is important that you**

- **Come to your study visits. If you miss a visit, call us and make a new appointment**
- **Take a pill every day**
- **Fill out your pill diary every day**
- **Bring your pill bottle back to the clinic at each visit**
- **Use condoms whenever you have sex**

**It is important that you …**

- **DON”T share your pills with anyone else**
  - **It could ruin the study**
  - **You could run out of pills before your next visit**
  - **It is not safe for HIV positive people to take this pill alone to fight the virus**
  - **Many people don’t know whether they have HIV right now**
- **DON’T trust the pill to protect you from HIV infection**
  - **We don’t know if you are taking the study medicine or placebo**
  - **We aren’t sure yet that the study medicine works to protect against HIV infection**
  - **Even if the study medicine protects sometimes, it probably won’t protect you all the time**

Page 17

**How We Will Know If It Works**

**Will people in study be put at risk for getting HIV?**

- **NO**
- **People who join the study are having sex and so will already have a risk of getting HIV**
  - **If they and their partners have not both tested negative for HIV**
  - **If they or their partner have other partners**
  - **If they or their partners don’t use condoms every time they have sex**
- **In the study, we will help everyone lower their risk**
- **But not everyone will be able to avoid risk completely**
  - **Some partners will refuse to have an HIV test**
  - **Some will forget to use condoms**
  - **Some will have a condom slip off or break**
  - **Some will not know that their partner has other partners**
- **So when they do take risks we can see if the study medicine works as a backup to keep them from getting HIV infection**

Before trial

During trial

RISK

**Condoms + Placebo**

**Condoms +TDF/FTC**

**(if it does NOT work)**

**Condoms +TDF/FTC**

**(IF it works)**

Page 18

**How We Protect you In the Study**

**Information you give us**

- **We keep all the information about you from interviews and lab tests stored under a code number assigned to you. We do not ever use your name on these records.**
- **We lock up the record of your name and information about how to contact you and only let a few people see it when we need to find you because you missed your appointment or we need to tell you about a test result**

**Your health**

- **Everything we do in the study has been reviewed and approved by**
  - **The Health Research Unit of the Ministry of Health**
  - **The research ethics review panel at the U.S. Centers for Disease Control and Prevention**
- **We have arranged for an international group of scientists who are not involved in this study to review the information we get about people in the study every few months. They will stop the study if**
  - **pills are making people sick**
  - **people are taking too many risks because they think the pills will protect them**
  - **we already have enough information to know whether the study medication works**

Page 19

**What To Do If You Have Complaints**

**How you are treated while you are in the study**

- **We have trained our study staff to be as gentle and helpful as possible but if you have complaints about something that happened while you were in the study, you can**
  - **Contact the health research unit in the Ministry of Health**
    - **Name/number**
  - **Contact the main investigator of the study**
    - **Name/number**
  - **Contact the director at the clinic**
    - **Name/number in Gaborone**
    - **Name/number in Francistown**
  - **Contact the community advisory board**
    - **Name/number in Gaborone**
    - **Name/number in Francistown**
  - **Contact the participant advisory board**
    - **Name/number in Gaborone**
    - **Name/number in Francistown**

Page 20

**What Risks Are There If you Join the Study**

- **Some questions we ask may be a little embarrassing for you to answer**
- **Physical examinations may be a little uncomfortable**
- **Taking blood from your finger or arm will be a little painful and might leave a bruise or cause a small amount of bleeding**
- **There may be risks in taking the study medicine that we don’t know about yet**
- **You may find out that you have HIV infection and that will be upsetting**
- **If you are taking the study medicine and you get HIV infection anyway, there is a small chance that your virus may learn how to fight off the medicine so it doesn’t work on the virus any more. This might affect your treatment in the future. But if you do become infected, we will perform other tests which can tell whether the virus has become resistant to TDF and FTC. These results will be shared with your doctor and can help your doctor identify the best treatment for you.**
- **If you are taking the study medicine, there is a small chance it may mildly effect your kidneys or your bones**

Page 21

**What Benefits Are There If You Join the Study**

- **You will know your HIV status while you are in the study**
- **You will get free physical examinations**
- **You will get free treatment for any sexually transmitted infections you have**
- **You will get personal counseling about how to lower your chances of getting HIV**
- **You will get help to get the care and support you need if you find out you have HIV infection**
- **You will get to help fight HIV by being in this research study**

Page 22

**What Compensation Is There In the Study**

**Travel Costs**

- **We don’t want it to cost you anything to come to study visits so we will give you a small payment (30 Pula) at each study visit to cover the cost of your travel**

**Time and Effort**

- **The visits will take up some of your time and we will be asking you to put in some effort to answer questions, give blood, have exams and other things you are only doing because of the study. To show our appreciation for this effort, we will give you a small payment for your time, 5 Pula for short visits, 10 Pula for medium visits, and 15 Pula for the longest visits**

Page 23

**Summing Up**

- **This study will test the safety and effectiveness of a single pill of TDF/FTC daily taken to reduce to risk of becoming infected with HIV**
- **Young Batswana women and men between the ages of 18 and 29 who agree to join the study will be**
  - **assigned to take either a TDF/FTC pill (study medicine) or a placebo pill every day**
  - **asked to come for study visits every month for at least a year**
  - **counseled about how to reduce their chances of getting HIV by changing their behaviors**
  - **given tests and free treatment for sexually transmitted infections**
  - **given an oral HIV test every month**

**It’s Your Choice**

**Ask questions**

**Think about whether joining the study is right for you**

Page 24

**Map to the Francistown Clinic**

**Plot 2806**

**Blue Jacket Street**

**To TatiTown**

**Century**

**Office**

**Supply**

**Woolworths**

**Ntsha**

**House**

**Score FNB**


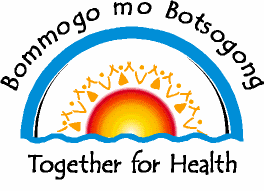


Page 25

**Map to the Gaborone Clinic**

**Plot 725**

**President**

**Hotel**

**Main Mall**

**Ext. 2**

**Clinic**

**Main Police Station**

**Station**


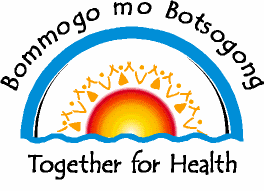


**To Princess**

**Marina Hospital**

**Facts About The Study Medication**

- **The pill you are taking**
  - **may contain medicine**

**or**

- - **may contain no medicine**
- **IF it contains medicine, the medicines are called Tenofovir (or TDF) and Emtricitabine (or FTC). Both medicines are in a single pill.**
- **Medicines used to fight HIV in people who are infected are called antiretrovirals or ARVs. TDF and FTC are antiretrovirals.**
- **We don’t know yet whether TDF/FTC will help to block HIV infection from happening in the first place. That is what this study is trying to find out.**
- **The most common side effects of TDF/FTC in people with HIV infection are: loose stools, upset stomach or passing gas.**
- **These side effects can also occur for other reasons like eating bad food, stomach flu, or reactions to specific foods like milk. So if you have them, it doesn’t mean that you are taking TDF/FTC.**
- **TDF/FTC can also affect some people’s kidneys. You wouldn’t feel anything different so we will check your blood to see if this happens.**
- **Please let us know about any illnesses or health problems you have while you’re in the study. This will help us to find out if TDF/FTC is causing any side effects we don’t expect now.**
- **If you have any serious illnesses while you are in the study, please let your health care worker know that you are in the study so that they can ask us any questions about the study medication that would help them provide you the best treatment for your illness. We will not discuss with them anything about your study visits or the information you have given us here. We will only talk with them about TDF/FTC itself. They can contact:**
  - **Gaborone:**
    - **Dr. Evans Buliva, cell and office phone**
    - **Dr. Rodreck Mutanhaurwa, cell and office phone**
  - **Francistown:**
    - **Dr. Daniel Abebe, cell and office phone**
    - **Dr. Lovemore Chirwa, cell and office phone**

**The Do’s and Don’ts of Taking Study Medication**

**Do’s The Reason**

Take your medicine every day. Will help the medicine work best.

When you’re at home, keep your medicine Will help you locate it when needed.

in the same place.

Keep your medicine in the labeled Will prevent losing or dropping pills

container in which it came.

Close the container after you take out Will prevent spoilage.

your medicine.

Keep your medicine in a dry place, Will prevent spoilage.

out of the sun.

Keep your medicine away from children. Will prevent children from taking it.

When you go on a trip, take enough Will prevent your running out of medicine.

medicine to last while you are away.

Return unused medicine to the study staff. Will prevent taking medicine that may

not be effective.

Talk to the study staff before you stop Will help the study staff work with you to

taking the medicine. solve any problems you are having with the study medicines

**Don’ts The Reason**

Do not share your medicine with anyone else. It probably won’t help them; and it may harm them.

Do not take more than one pill a day, even if It puts too much medicine in your body at one

you forgot to take a dose the day before. time. You need a smaller amount on a regular basis.

**Special Instructions For Your Next Visit**

Before your next visit on ________ __ __ / __ __ we would like you to do two special things: (weekday) dd mm

On your medication diary, ***write down the time you take your*** ***medicine*** on the two days before your visit.

________ __ __ / __ __

(weekday) dd mm

________ __ __ / __ __

(weekday) dd mm

|  | **Tues** | **Wed** |  |
| --- | --- | --- | --- |
|  | 1  **M**  9:15 AM | 2  **A**  2:30 PM |  |
|  |  |  |  |

These dates are highlighted on your medication diary. Please be as exact as possible about the time.

***Don’t take your medicine for that day until you come to the clinic.***

**C. VISIT REIMBURSEMENT TABLE**

Persons attending any study visit (screening, enrollment, follow-up, and exit visits) will be given a small reimbursement at the conclusion of each data collection visit to cover anticipated average travel costs and an amount in recompense for the time, effort, and discomfort of study procedures.

The travel amount (30 Pula/5.50 USD) was calculated as the cost for taking public transportation for a two-way trip of up to 1 hours travel distance from the study clinic.

The time end effort reimbursement was calculated at 5 P per hour (or less) which is the wage for a local domestic worker. Because visit procedures and duration differ throughout the study, reimbursements will vary as follows:

**Table of Time and Effort Reimbursements**

(calculated at 5.5 Pula per U.S. dollar)

| **Visit Type** | **Approx. Duration** | **Procedures** | **Reimbursement**  **Pula (USD)** |
| --- | --- | --- | --- |
| **Screening** | 15 min  10 min  30 min  15 min  5 min  5 min  20 min  15 min  **Total ~ 2.0 hours** | Consent  Screening Interview  RR Counseling  HIV rapid test  Phlebotomy  Urine sample  Trial education  Comprehension test | 10 P ($1.80) |
| **Repeat Test**  (if failed comprehension test) | 45 min  15 min  **Total ~ 1 hour** | Study (re)education  Comprehension Test | 5 P ($0.90) |

| **Visit Type** | **Approx. Duration** | **Procedures** | **Reimbursement**  **Pula (USD)** |
| --- | --- | --- | --- |
| **Enrollment** | 10 min  20 min  30 min  20 min  10 min  15 min  5 min  5 min  **Total ~ 2.5 hours** | Lab eligibility review  Consent  Baseline Interview  Physical Exam/treatment  Drug/Condom Dispensing  Adherence Counseling  Phlebotomy  urine sample | 15 P ($2.70) |
| **Monthly Visit** | 15 min  20 min  10 min  15 min  **Total ~ 1 hour** | Brief Interview  Adherence Counseling  Drug/condom dispensing  HIV rapid test | 5 P ($0.90) |
| **Quarterly Visit** | 30 min  30 min  10 min  5 min  10 min  15 min  **Total ~ 2 hour** | Extended Interview  Adherence and RR Counseling  Phlebotomy  Urine collection  Drug/condom dispensing  HIV rapid test | 10 P ($1.80) |

| **Semi-Annual Visit** | 30 min  30 min  30 min  10 min  5 min  10 min  15 min  **Total ~ 2.5 hours** | Extended Interview  Physical Exam/treatment  Adherence and RR Counseling  Phlebotomy  Urine collection  Drug/Condom dispensing  HIV rapid test | 15 P ($2.70) |
| --- | --- | --- | --- |

| **Exit Visit** | 30 min  10 min  15 min  **Total ~ 1.0 hours** | Brief Interview  Phlebotomy  HIV rapid test | 5 P ($0.90) |
| --- | --- | --- | --- |

| **Visit Type** | **Approx. Duration** | **Procedures** | **Reimbursement**  **Pula (USD)** |
| --- | --- | --- | --- |

| **Semiquantitative**  **Interview** | 90 min  **Total ~ 1.5 hour** | Interview | 10 P ($1.80) |
| --- | --- | --- | --- |
| **Interim Visit** | **Total ~ 0.5 hours** | As indicated | 5 P ($0.90) |
| **DEXA Substudy** | **Total ~ 0.5 hours** | Scanning | 5 P ($0.90) |
| **PK/PD Substudy** | **8 hours** | 3 month visit plus  Additional phlebotomy at 1, 4, 8 hours post dose (total 5 blood draws) | 100 P ($18.20) |

**D. LABORATORY TESTING**

| **Condition/ Agent** | **Specimen** | **Visits** | **Assay** | **Assay site** |
| --- | --- | --- | --- | --- |
| HIV infection  (concurrent rapid test) | fingerstick whole blood | Screening visit | Determine HIV (Abbott)  Oraquick (Orasure) | clinic exam room |
| HIV infection | Oral transudate | All follow-up visits | Oraquick (Orasure) |  |
| Syphilis | fingerstick whole blood | Enrollment visit and  q 12 months | Determine Syphilis (Abbott) |  |
| HIV seroincidence | red-top (8ml) | all positive rapid tests | “detuned” procedure for  Determine HIV (Abbott)  Oraquick (Orasure) | Prevention trial support lab or private labs (chemistry) |
| HIV infection (QA and confirmation) |  | 5% all negative rapid tests  100% discordant and positive rapid tests | EIA: Biorad (1.2.0) |  |
| Chemistries |  | Screening visit and then q 3 months | Cobas Analyzer |  |
| HSV-2 |  | Enrollment visit | HerpesSelect ELISA (Focus) |  |
| syphilis |  | Enrollment and q 12 months | RPR (with titre if +)  Serum TPPA (QA only) |  |
| Hepatitis B |  | Screening visit | EIA |  |
| serum repository | remnant sera | Screening and then quarterly | future testing | stored in on-site clinic lab |
| Gonorrhea | cervical swab (women)  urine (men) | Enrollment and q 6 months | Cobas Amplicor PCR (Roche) | Prevention trial support lab |
| Chlamydia |  |  |  |  |
| Trichomonas | vaginal swab | Enrollment and q 6 months | In-Pouch culture (Biomed) | on-site clinic lab |
| Bacterial vaginosis | vaginal swab | Enrollment and q 6 months | gram stain |  |
| Candida | vaginal or glans swab | only if signs and symptoms | KOH wet prep | on-site clinic lab |
| cervical dysplasia | cytobrush | Enrollment visit and q 12 months | PAP smear (Cytec ThinPrep) | Pathcare cytopathology lab |
| Hgb | purple-top (5 ml) | HIV- only, screening visits | Sysmex (Roche) | Prevention trials support lab or private labs |
| CD4 | purple-top (5 ml) | HIV+ only, at  Screening visit or  Seroconversion visit and  q 6 months thereafter | FacsCount | Prevention trial support lab |
| HIV plasma viral load | CPT tube (8 ml) | HIV+ only, at  Seroconversion visit and q 6 months thereafter | Cobas Amplicor PCR (Roche) | Prevention trial support lab |
| PBMC repository |  | Enrollment, month 1 and 2 and quarterly | future testing | stored in BOTUSA HIV prevention research lab |
| plasma respository |  |  |  |  |
| Pregnancy | Urine (women) | All visits in seronegatives  Seroconversion and exit visit in seroconverters | Rapid β-HCG | clinic exam room |
| Viral resistance | PBMC | All seroconvertors | Visible Genetics/TruGene | CDC-Atlanta laboratory |
| TDF and FTC levels | Pre and post dose  CPT (10 ml)  EDTA (4 ml) | All seroconverters in TDF/FTC arm and random 20% of non-seroconverters | HPLC (TFV) | Johns Hopkins |
|  |  |  | LC/MS/MS (TDP) | Gilead Sciences |
| PBMC activation |  | One quarterly visit | CD38+ flow  (frozen PBMC) | CDC-Atlanta laboratory |

**E. INVESTIGATOR’S BROCHURE**


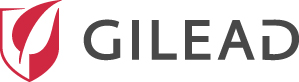


Investigator’s Brochure

Emtricitabine/Tenofovir Disoproxil Fumarate Tablets

Human Immunodeficiency Virus Type 1 Infection

2nd Edition:

17 November 2005

Gilead Sciences, Inc.

333 Lakeside Drive

Foster City, CA 94404

USA

Previous Edition:

12 April 2004

| CONFIDENTIALITY STATEMENT |
| --- |
| The information contained in this document, particularly unpublished data, is the property or under control of Gilead Sciences, Inc., and is provided to you in confidence as an investigator, potential investigator, or consultant, for review by you, your staff, and an applicable Institutional Review Board or Independent Ethics Committee. The information is only to be used by you in connection with authorized clinical studies of the investigational drug described in the protocol. You will not disclose any of the information to others without written authorization from Gilead Sciences, Inc., except to the extent necessary to obtain informed consent from those persons to whom the drug may be administered. |

Table of Contents

Title Page 1

[**1. Document Overview 211**](#__RefHeading___Toc120492192)

[2. Approved United States Package Insert (May 2005) and Patient Information (May 2005) for Truvada (Emtricitabine and Tenofovir Disoproxil Fumarate) Tablets 212](#__RefHeading___Toc120492193)

[3. Nonclinical Studies 240](#__RefHeading___Toc120492194)

[3.1. Toxicology 240](#__RefHeading___Toc120492195)

[3.1.1. Repeated Dose Toxicity 240](#__RefHeading___Toc120492196)

[3.1.2. Mutagenicity and Carcinogenicity 240](#__RefHeading___Toc120492197)

[3.1.3. Mitochondrial Toxicity 241](#__RefHeading___Toc120492198)

[3.1.4. Toxicology Conclusions 241](#__RefHeading___Toc120492199)

[4. Clinical Studies 242](#__RefHeading___Toc120492200)

[4.1. Study GS-01-934 242](#__RefHeading___Toc120492201)

[4.1.1. Demographics and Baseline Characteristics 242](#__RefHeading___Toc120492202)

[4.1.2. Efficacy Results 242](#__RefHeading___Toc120492203)

[4.1.3. Safety Results 247](#__RefHeading___Toc120492204)

[4.2. Study M02-418 249](#__RefHeading___Toc120492205)

[4.2.1. Demographics and Baseline Characteristics 249](#__RefHeading___Toc120492206)

[4.2.2. Efficacy Results 249](#__RefHeading___Toc120492207)

[4.2.3. Safety Results 252](#__RefHeading___Toc120492208)

[5. Marketing Experience 256](#__RefHeading___Toc120492209)

[6. Summary of Data and Guidance for the Investigator 259](#__RefHeading___Toc120492210)

[7. References 260](#__RefHeading___Toc120492211)

[8. Appendices 261](#__RefHeading___Toc120492212)

[Appendix 1. Expected Adverse Events Based on the Truvada Company Core Safety Information (Version 5.1, Effective Date 14 June 2005) 262](#__RefHeading___Toc120492213)

LIST OF TABLES

[Table 1. Treatment Outcomes at Week 48 in Study GS-01-934 (TLOVR Analysis) 243](#__RefHeading___Toc120492215)

[Table 2. Kaplan-Meier Estimates for Loss of Virologic Response and Pure Virologic Failure by Week 48 (Study GS-001-934, ITT) 244](#__RefHeading___Toc120492216)

[Table 3. Distribution of HIV‑1 Subtypes (Study GS-01-934, ITT) 246](#__RefHeading___Toc120492217)

[Table 4. Treatment-Emergent Adverse Events Reported in at Least 10% of Patients in Either Treatment Group in Study GS-01-934 (Week 48 Safety Analysis Set) 247](#__RefHeading___Toc120492218)

[Table 5. Proportion of Patients with Plasma HIV-1 RNA Levels < 50 Copies/mL at Week 48 and Week 96 (Study M02-418) 250](#__RefHeading___Toc120492219)

[Table 6. Comparison of Response Rates in Treatment-Naive Patients Treated With Lopinavir/Ritonavir + 2 NRTI Regimens 250](#__RefHeading___Toc120492220)

[Table 7. Proportion of Patients with Plasma HIV-1 RNA Levels < 50 Copies/mL at Week 48 and Week 96 by Baseline HIV-1 and CD4+ Cell Count (ITT NC = F, Study M02-418) 251](#__RefHeading___Toc120492221)

[Table 8. Treatment-Emergent Adverse Events Reported in at Least 10% of Patients Overall (Week 96, Study M02-418) 253](#__RefHeading___Toc120492222)

[Table 9. Distribution of Maximum Serum Creatinine and Minimum Serum Phosphorus Concentrations Through Week 96 (Studies M02-418 and GS-99-903) 255](#__RefHeading___Toc120492223)

[Table 10. Marketing Authorizations for Emtricitabine/Tenofovir Disoproxil Fumarate Tablets as of 09 November 2005 257](#__RefHeading___Toc120492224)

LIST OF FIGURES

[Figure 1. Mean Change From Baseline (95% CI) in CD4+ Cell Count in Study GS-01-934 (As-treated Analysis Set) 245](#__RefHeading___Toc120492225)

[Figure 2. Mean Change From Baseline in CD4+ Cell Count Over Time After Initiation of LPV/r Once or Twice Daily Plus FTC + TDF Once Daily (Study M02-418) 252](#__RefHeading___Toc120492226)

Glossary of Abbreviations and Definitions of Terms

| 3TC | lamivudine |
| --- | --- |
| ABC | abacavir |
| AE | adverse event |
| ALT | alanine aminotransferase |
| AST | aspartate aminotransferase |
| AUC | area under the concentration versus time curve |
| AZT | zidovudine |
| BID | twice daily |
| BMD | bone mineral density |
| BUN | blood urea nitrogen |
| CI | confidence interval |
| CL_cr_ | creatinine clearance |
| C_max_ | maximum observed concentration of drug in serum, plasma, or peripheral blood mononuclear cells |
| COSTART | Coding Symbols for a Thesaurus of Adverse Reaction Terms |
| COX II | cytochrome c oxidase II |
| d4T | stavudine |
| ddI | didanosine |
| DNA | deoxyribonucleic acid |
| EFV | efavirenz |
| FDA | (United States) Food and Drug Administration |
| FTC | emtricitabine |
| FTCTP | emtricitabine triphosphate |
| GGT | gamma‑glutamyl transferase |
| HBV | hepatitis B virus |
| HIV‑1 | human immunodeficiency virus type 1 |
| HPF | high-power field (microscope) |
| ITT | intent-to-treat |
| LLQ | lower limit of quantitation |
| LPV/r | lopinavir/ritonavir |
| MAC | *Mycobacterium avium* complex |
| MedDRA | Medical Dictionary for Regulatory Activities |
| MITT | modified intent-to-treat |
| mtDNA | mitochondrial DNA |

| Glossary of Abbreviations and Definitions of Terms (Continued) | |
| --- | --- |
| NA | not applicable |
| NC | not calculated |
| NC = F | noncompleter equals failure |
| NNRTI | nonnucleoside reverse transcriptase inhibitor |
| NOAEL | no-observed-adverse-effect level |
| NRTI | nucleoside reverse transcriptase inhibitor |
| NtRTI | nucleotide reverse transcriptase inhibitor |
| PEPFAR | President’s Emergency Plan for AIDS Relief |
| PMPApp | tenofovir diphosphate |
| QD | once daily |
| RBC | red blood cell(s) |
| RNA | ribonucleic acid |
| RT | reverse transcriptase |
| SAE | serious adverse event |
| SD | standard deviation |
| SBT | stable background therapy |
| TDF | tenofovir disoproxil fumarate, tenofovir DF |
| TFV | tenofovir |
| TLOVR | time-to-loss-of-virologic-response algorithm |
| ULN | upper limit of normal range |
| US | United States |

# Document Overview

This investigator’s brochure provides current information about the fixed‑dose combination tablets containing emtricitabine and tenofovir disoproxil fumarate (Truvada®, emtricitabine/tenofovir DF tablets) for use in combination with other antiretroviral agents in the treatment of human immunodeficiency virus type 1 (HIV‑1) in adults. Each fixed‑dose combination tablet contains 200 mg emtricitabine and 300 mg tenofovir disoproxil fumarate (which is equivalent to 245 mg of tenofovir disoproxil) as active ingredients. Emtricitabine/tenofovir DF tablets have been approved for the treatment of HIV‑1 infection in a number of markets worldwide, including the United States and the European Union.

Emtricitabine (FTC) is a nucleoside reverse transcriptase inhibitor (NRTI) approved as a once-a-day capsule (200 mg), in combination with other antiretroviral agents, for the treatment of HIV‑1 infection. Emtricitabine is a synthetic analog of the naturally occurring pyrimidine nucleoside, 2′‑deoxycytidine. Intracellularly, emtricitabine is phosphorylated by cellular enzymes to form emtricitabine triphosphate (FTCTP), the active metabolite. Emtricitabine has been approved for the treatment of HIV‑1 infection in a number of markets worldwide, including the United States and the European Union.

Tenofovir disoproxil fumarate (tenofovir DF, TDF), the oral prodrug of tenofovir, is a nucleotide reverse transcriptase inhibitor (NtRTI) approved as a once-a-day tablet (300 mg), in combination with other antiretroviral agents, for the treatment of HIV‑1 infection. After absorption, tenofovir DF is rapidly converted to tenofovir, which is metabolized intracellularly to the active metabolite, tenofovir diphosphate (PMPApp). Tenofovir DF has been approved for the treatment of HIV‑1 infection in a number of markets worldwide, including the United States and the European Union.

Information in this investigator’s brochure is organized as follows:

- The current approved United States (US) package insert and patient information for Truvada® (emtricitabine and tenofovir disoproxil fumarate) Tablets are provided in Section .
- Results from additional nonclinical studies are presented in Section .
- New information from ongoing and additional clinical studies is presented in Section .
- The marketing history of Truvada is summarized in Section .
- A brief Summary of Data and Guidance for the Investigator is provided in Section .

The Truvada package insert and patient information included in this brochure are applicable for the US only. Sites outside the US should refer to the labeling approved for their respective countries.

# Approved United States Package Insert (May 2005) and Patient Information (May 2005) for Truvada (Emtricitabine and Tenofovir Disoproxil Fumarate) Tablets

**TRUVADA®**

**(emtricitabine and tenofovir disoproxil fumarate)**

**Tablets**

℞ **Only**

**WARNING**

**LACTIC ACIDOSIS AND SEVERE HEPATOMEGALY WITH STEATOSIS,**

**INCLUDING FATAL CASES, HAVE BEEN REPORTED WITH THE USE OF**

**NUCLEOSIDE ANALOGS ALONE OR IN COMBINATION WITH OTHER**

**ANTIRETROVIRALS (SEE WARNINGS).**

**TRUVADA® IS NOT INDICATED FOR THE TREATMENT OF CHRONIC HEPATITIS**

**B VIRUS (HBV) INFECTION AND THE SAFETY AND EFFICACY OF TRUVADA**

**HAVE NOT BEEN ESTABLISHED IN PATIENTS COINFECTED WITH HBV AND**

**HIV. SEVERE ACUTE EXACERBATIONS OF HEPATITIS B HAVE BEEN**

**REPORTED IN PATIENTS WHO HAVE DISCONTINUED EMTRIVA**® **or VIREAD**®**.**

**HEPATIC FUNCTION SHOULD BE MONITORED CLOSELY WITH BOTH CLINICAL**

**AND LABORATORY FOLLOW-UP FOR AT LEAST SEVERAL MONTHS IN**

**PATIENTS WHO DISCONTINUE TRUVADA AND ARE COINFECTED WITH HIV**

**AND HBV. IF APPROPRIATE, INITIATION OF ANTI-HEPATITIS B THERAPY MAY**

**BE WARRANTED (SEE WARNINGS).**

**DESCRIPTION**

TRUVADA Tablets are fixed dose combination tablets containing emtricitabine and

tenofovir disoproxil fumarate. EMTRIVA is the brand name for emtricitabine, a synthetic

nucleoside analog of cytidine. Tenofovir disoproxil fumarate (VIREAD, also known as

tenofovir DF) is converted in vivo to tenofovir, an acyclic nucleoside phosphonate

(nucleotide) analog of adenosine 5′-monophosphate. Both emtricitabine and tenofovir

exhibit inhibitory activity against HIV-1 reverse transcriptase.

TRUVADA Tablets are for oral administration. Each film-coated tablet contains 200 mg

of emtricitabine and 300 mg of tenofovir disoproxil fumarate, (which is equivalent to 245

mg of tenofovir disoproxil), as active ingredients. The tablets also include the following

inactive ingredients: croscarmellose sodium, lactose monohydrate, magnesium

stearate, microcrystalline cellulose, and pregelatinized starch (gluten free). The tablets

are coated with Opadry II Blue Y-30-10701, which contains FD&C Blue #2 aluminum

lake, hypromellose, lactose monohydrate, titanium dioxide, and triacetin.

**Emtricitabine:** The chemical name of emtricitabine is 5-fluoro-1-(2*R*,5*S*)-[2-

(hydroxymethyl)-1,3-oxathiolan-5-yl]cytosine. Emtricitabine is the (-) enantiomer of a

thio analog of cytidine, which differs from other cytidine analogs in that it has a fluorine

in the 5-position.

It has a molecular formula of C8H10FN3O3S and a molecular weight of 247.24. It has the

following structural formula:


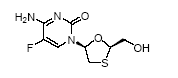


Emtricitabine is a white to off-white crystalline powder with a solubility of approximately

112 mg/mL in water at 25 oC. The partition coefficient (log p) for emtricitabine is -0.43

and the pKa is 2.65.

**Tenofovir disoproxil fumarate:** Tenofovir disoproxil fumarate is a fumaric acid salt of

the bis-isopropoxycarbonyloxymethyl ester derivative of tenofovir. The chemical name

of tenofovir disoproxil fumarate is 9-[(*R*)-2 [[bis[[(isopropoxycarbonyl)oxy]-

methoxy]phosphinyl]methoxy]propyl]adenine fumarate (1:1). It has a molecular formula

of C19H30N5O10P • C4H4O4 and a molecular weight of 635.52. It has the following

structural formula:


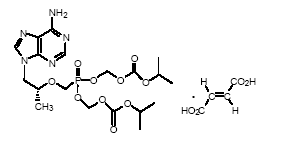


Tenofovir disoproxil fumarate is a white to off-white crystalline powder with a solubility of

13.4 mg/mL in water at 25 °C. The partition coefficient (log p) for tenofovir disoproxil is

1.25 and the pKa is 3.75. All dosages are expressed in terms of tenofovir disoproxil

fumarate except where otherwise noted.

**MICROBIOLOGY**

**Mechanism of Action**

**Emtricitabine:** Emtricitabine, a synthetic nucleoside analog of cytidine, is

phosphorylated by cellular enzymes to form emtricitabine 5'-triphosphate. Emtricitabine

5'-triphosphate inhibits the activity of the HIV-1 reverse transcriptase (RT) by competing

with the natural substrate deoxycytidine 5'-triphosphate and by being incorporated into

nascent viral DNA which results in chain termination. Emtricitabine 5′-triphosphate is a

weak inhibitor of mammalian DNA polymerase α, β, ε and mitochondrial DNA

polymerase γ.

**Tenofovir disoproxil fumarate:** Tenofovir disoproxil fumarate is an acyclic nucleoside

phosphonate diester analog of adenosine monophosphate. Tenofovir disoproxil fumarate

requires initial diester hydrolysis for conversion to tenofovir and subsequent

phosphorylations by cellular enzymes to form tenofovir diphosphate. Tenofovir

diphosphate inhibits the activity of HIV-1 RT by competing with the natural substrate

deoxyadenosine 5′-triphosphate and, after incorporation into DNA, by DNA chain

termination. Tenofovir diphosphate is a weak inhibitor of mammalian DNA polymerases α,

β, and mitochondrial DNA polymerase γ.

**Antiviral Activity**

**Emtricitabine and tenofovir disoproxil fumarate:** In combination studies evaluating

the in vitro antiviral activity of emtricitabine and tenofovir together, synergistic antiviral

effects were observed.

**Emtricitabine:** The in vitro antiviral activity of emtricitabine against laboratory and

clinical isolates of HIV was assessed in lymphoblastoid cell lines, the MAGI-CCR5 cell

line, and peripheral blood mononuclear cells. The IC50 values for emtricitabine were in

the range of 0.0013−0.64 µM (0.0003−0.158 µg/mL). In drug combination studies of

emtricitabine with nucleoside reverse transcriptase inhibitors (abacavir, lamivudine,

stavudine, zalcitabine, zidovudine), non-nucleoside reverse transcriptase inhibitors

(delavirdine, efavirenz, nevirapine), and protease inhibitors (amprenavir, nelfinavir,

ritonavir, saquinavir), additive to synergistic effects were observed. Most of these drug

combinations have not been studied in humans. Emtricitabine displayed antiviral

activity in vitro against HIV-1 clades A, B, C, D, E, F, and G (IC50 values ranged from

0.007−0.075 µM) and showed strain specific activity against HIV-2 (IC50 values ranged

from 0.007−1.5 µM).

**Tenofovir disoproxil fumarate:** The in vitro antiviral activity of tenofovir against

laboratory and clinical isolates of HIV-1 was assessed in lymphoblastoid cell lines,

primary monocyte/macrophage cells and peripheral blood lymphocytes. The IC50 (50%

inhibitory concentration) values for tenofovir were in the range of 0.04−8.5 µM. In drug

combination studies of tenofovir with nucleoside reverse transcriptase inhibitors

(abacavir, didanosine, lamivudine, stavudine, zalcitabine, zidovudine), non-nucleoside

reverse transcriptase inhibitors (delavirdine, efavirenz, nevirapine), and protease

inhibitors (amprenavir, indinavir, nelfinavir, ritonavir, saquinavir), additive to synergistic

effects were observed. Tenofovir displayed antiviral activity in vitro against HIV-1

clades A, B, C, D, E, F, G and O (IC50 values ranged from 0.5−2.2 µM). The IC50 values

of tenofovir against HIV-2 ranged from 1.6 µM to 4.9 µM.

**Resistance**

**Emtricitabine and tenofovir disoproxil fumarate:** HIV-1 isolates with reduced

susceptibility to the combination of emtricitabine and tenofovir have been selected in

vitro. Genotypic analysis of these isolates identified the M184I/V and/or K65R amino

acid substitutions in the viral RT.

**Emtricitabine:** Emtricitabine-resistant isolates of HIV have been selected in vitro.

Genotypic analysis of these isolates showed that the reduced susceptibility to

emtricitabine was associated with a mutation in the HIV RT gene at codon 184 which

resulted in an amino acid substitution of methionine by valine or isoleucine (M184V/I).

Emtricitabine-resistant isolates of HIV have been recovered from some patients treated

with emtricitabine alone or in combination with other antiretroviral agents. In a clinical

study, viral isolates from 6/16 (37.5%) treatment-naïve patients with virologic failure

showed >20-fold reduced susceptibility to emtricitabine. Genotypic analysis of these

isolates showed that the resistance was due to M184V/I mutations in the HIV RT gene.

**Tenofovir disoproxil fumarate:** HIV-1 isolates with reduced susceptibility to tenofovir

have been selected in vitro. These viruses expressed a K65R mutation in RT and

showed a 2–4 fold reduction in susceptibility to tenofovir.

Tenofovir-resistant isolates of HIV-1 have also been recovered from some patients

treated with VIREAD in combination with certain antiretroviral agents. In treatment-naïve

patients, 8/47 (17%) isolates from patients failing VIREAD + lamivudine + efavirenz

through week 144 showed >1.4 fold (median 3.7) reduced susceptibility in vitro to

tenofovir. In treatment-experienced patients, 14/304 (5%, Studies 902 and 907) isolates

from patients failing VIREAD through week 96 showed >1.4 fold (median 2.7) reduced

susceptibility to tenofovir. Genotypic analysis of the resistant isolates showed a mutation

in the HIV-1 RT gene resulting in the K65R amino acid substitution.

**Cross-resistance**

**Emtricitabine and tenofovir disoproxil fumarate:** Cross-resistance among certain

nucleoside reverse transcriptase inhibitors (NRTIs) has been recognized. The M184V/I

and/or K65R substitutions selected in vitro by the combination of emtricitabine and

tenofovir are also observed in some HIV-1 isolates from subjects failing treatment with

tenofovir in combination with either lamivudine or emtricitabine, and either abacavir or

didanosine. Therefore, cross-resistance among these drugs may occur in patients

whose virus harbors either or both of these amino acid substitutions.

**Emtricitabine:** Emtricitabine-resistant isolates (M184V/I) were cross-resistant to

lamivudine and zalcitabine but retained susceptibility in vitro to didanosine, stavudine,

tenofovir, zidovudine, and NNRTIs (delavirdine, efavirenz, and nevirapine). Isolates

from heavily treatment-experienced patients containing the M184V/I amino acid

substitution in the context of other NRTI resistance-associated substitutions may retain

susceptibility to tenofovir. HIV-1 isolates containing the K65R substitution, selected in

vivo by abacavir, didanosine, tenofovir, and zalcitabine, demonstrated reduced

susceptibility to inhibition by emtricitabine. Viruses harboring mutations conferring

reduced susceptibility to stavudine and zidovudine (M41L, D67N, K70R, L210W,

T215Y/F, K219Q/E) or didanosine (L74V) remained sensitive to emtricitabine. HIV-1

containing the K103N substitution associated with resistance to NNRTIs was

susceptible to emtricitabine.

**Tenofovir disoproxil fumarate:** HIV-1 isolates from patients (N=20) whose HIV-1

expressed a mean of 3 zidovudine-associated RT amino acid substitutions (M41L,

D67N, K70R, L210W, T215Y/F or K219Q/E/N) showed a 3.1-fold decrease in the

susceptibility to tenofovir. Multinucleoside resistant HIV-1 with a T69S double insertion

mutation in the RT showed reduced susceptibility to tenofovir.

**CLINICAL PHARMACOLOGY**

**Pharmacokinetics in Adults**

**TRUVADA:** One TRUVADA Tablet was bioequivalent to one EMTRIVA Capsule

(200 mg) plus one VIREAD Tablet (300 mg) following single-dose administration to

fasting healthy subjects (N=39).

**Emtricitabine:** The pharmacokinetic properties of emtricitabine are summarized in

Table 1. Following oral administration of EMTRIVA, emtricitabine is rapidly absorbed

with peak plasma concentrations occurring at 1–2 hours post-dose. In vitro binding of

emtricitabine to human plasma proteins is <4% and is independent of concentration

over the range of 0.02−200 µg/mL. Following administration of radiolabelled

emtricitabine, approximately 86% is recovered in the urine and 13% is recovered as

metabolites. The metabolites of emtricitabine include 3′-sulfoxide diastereomers and

their glucuronic acid conjugate. Emtricitabine is eliminated by a combination of

glomerular filtration and active tubular secretion. Following a single oral dose of

EMTRIVA, the plasma emtricitabine half-life is approximately 10 hours.

**Tenofovir disoproxil fumarate:** The pharmacokinetic properties of tenofovir disoproxil

fumarate are summarized in Table 1. Following oral administration of VIREAD,

maximum tenofovir serum concentrations are achieved in 1.0 ± 0.4 hour. In vitro

binding of tenofovir to human plasma proteins is <0.7% and is independent of

concentration over the range of 0.01–25 µg/mL. Approximately 70−80% of the

intravenous dose of tenofovir is recovered as unchanged drug in the urine. Tenofovir is

eliminated by a combination of glomerular filtration and active tubular secretion.

Following a single oral dose of VIREAD, the terminal elimination half-life of tenofovir is

approximately 17 hours.

**Table 1 Single Dose Pharmacokinetic Parameters for Emtricitabine and**

**Tenofovir in Adults1**

**
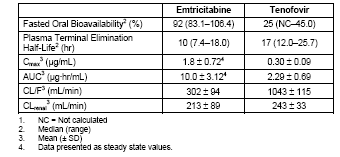
**

**Effects of Food on Oral Absorption**

TRUVADA may be administered with or without food. Administration of TRUVADA

following a high fat meal (784 kcal; 49 grams of fat) or a light meal (373 kcal; 8 grams of

fat) delayed the time of tenofovir Cmax by approximately 0.75 hour. The mean increases

in tenofovir AUC and Cmax were approximately 35% and 15%, respectively, when

administered with a high fat or light meal, compared to administration in the fasted state.

In previous safety and efficacy studies, VIREAD (tenofovir) was taken under fed

conditions. Emtricitabine systemic exposures (AUC and Cmax) were unaffected when

TRUVADA was administered with either a high fat or a light meal.

**Special Populations**

**Race**

**Emtricitabine:** No pharmacokinetic differences due to race have been identified

following the administration of EMTRIVA.

**Tenofovir disoproxil fumarate:** There were insufficient numbers from racial and ethnic

groups other than Caucasian to adequately determine potential pharmacokinetic

differences among these populations following the administration of VIREAD.

**Gender**

**Emtricitabine and tenofovir disoproxil fumarate:** Emtricitabine and tenofovir

pharmacokinetics are similar in male and female patients.

**Pediatric and Geriatric Patients:** Pharmacokinetics of emtricitabine and tenofovir have

not been fully evaluated in children (<18 years) or in the elderly (>65 years) **(see**

**PRECAUTIONS, Pediatric Use, Geriatric Use)**.

**Patients with Impaired Renal Function:** The pharmacokinetics of emtricitabine and

tenofovir are altered in patients with renal impairment **(see WARNINGS, Renal**

**Impairment)**. In patients with creatinine clearance <50 mL/min, Cmax, and AUC0-∞ of

emtricitabine and tenofovir were increased. It is recommended that the dosing interval

for TRUVADA be modified in patients with creatinine clearance 30–49 mL/min.

TRUVADA should not be used in patients with creatinine clearance <30 mL/min and in

patients with end-stage renal disease requiring dialysis **(see WARNINGS, Renal**

**Impairment)**.

**Patients with Hepatic Impairment:** The pharmacokinetics of tenofovir following a 300

mg dose of VIREAD have been studied in non-HIV infected patients with moderate to

severe hepatic impairment. There were no substantial alterations in tenofovir

pharmacokinetics in patients with hepatic impairment compared with unimpaired

patients. The pharmacokinetics of TRUVADA or emtricitabine have not been studied in

patients with hepatic impairment; however, emtricitabine is not significantly metabolized

by liver enzymes, so the impact of liver impairment should be limited.

**Pregnancy: (see PRECAUTIONS, Pregnancy)**

**Nursing Mothers: (see PRECAUTIONS, Nursing Mothers)**

**Drug Interactions: (see PRECAUTIONS, Drug Interactions)**

**TRUVADA:** No drug interaction studies have been conducted using TRUVADA

Tablets.

**Emtricitabine and tenofovir disoproxil fumarate:** The steady state pharmacokinetics

of emtricitabine and tenofovir were unaffected when emtricitabine and tenofovir

disoproxil fumarate were administered together versus each agent dosed alone.

In vitro and clinical pharmacokinetic drug-drug interaction studies have shown the

potential for CYP450 mediated interactions involving emtricitabine and tenofovir with

other medicinal products is low.

Emtricitabine and tenofovir are primarily excreted by the kidneys by a combination of

glomerular filtration and active tubular secretion. No drug-drug interactions due to

competition for renal excretion have been observed; however, coadministration of

TRUVADA with drugs that are eliminated by active tubular secretion may increase

concentrations of emtricitabine, tenofovir, and/or the coadministered drug.

Drugs that decrease renal function may increase concentrations of emtricitabine and/or

tenofovir.

No clinically significant drug interactions have been observed between emtricitabine and

famciclovir, indinavir, stavudine, and tenofovir disoproxil fumarate (see Tables 2 and 3).

Similarly, no clinically significant drug interactions have been observed between

tenofovir disoproxil fumarate and abacavir, adefovir dipivoxil, ribavirin, efavirenz,

emtricitabine, indinavir, lamivudine, lopinavir/ritonavir, methadone and oral

contraceptives in studies conducted in healthy volunteers (see Tables 4 and 5).

**Table 2 Drug Interactions: Changes in Pharmacokinetic Parameters for**

**Emtricitabine in the Presence of the Coadministered Drug1**

**
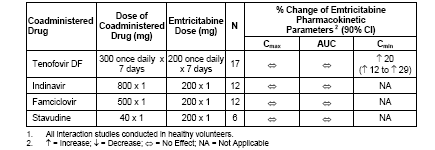
**

**Table 3 Drug Interactions: Changes in Pharmacokinetic Parameters for**

**Coadministered Drug in the Presence of Emtricitabine1**

**
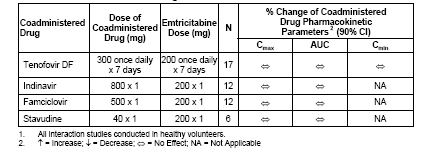
**

**Table 4 Drug Interactions: Changes in Pharmacokinetic Parameters for Tenofovir1**

**in the Presence of the Coadministered Drug**

**
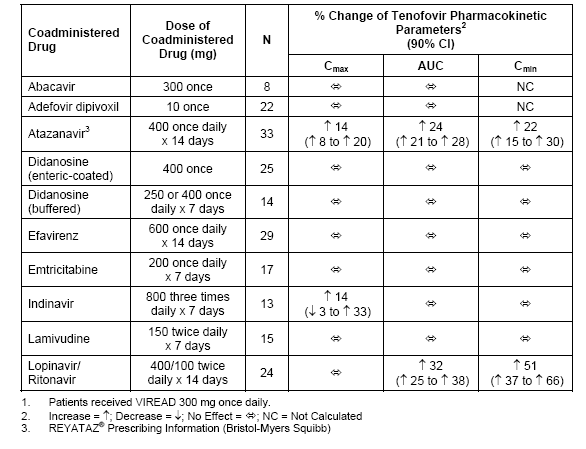
**

**Table 5 Drug Interactions: Changes in Pharmacokinetic Parameters for**

**Coadministered Drug in the Presence of Tenofovir**


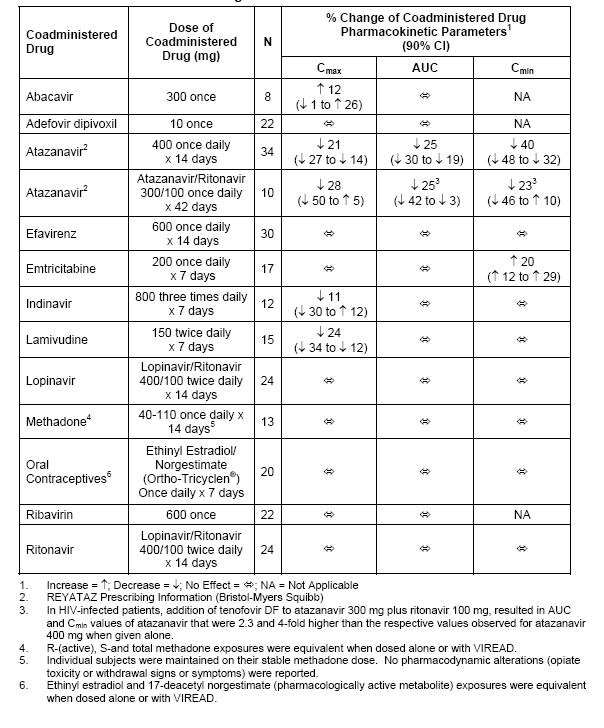


Following multiple dosing to HIV-negative subjects receiving either chronic methadone

maintenance therapy or oral contraceptives, or single doses of ribavirin, steady state

tenofovir pharmacokinetics were similar to those observed in previous studies,

indicating lack of clinically significant drug interactions between these agents and

VIREAD.

Coadministration of tenofovir disoproxil fumarate with didanosine results in changes in

the pharmacokinetics of didanosine that may be of clinical significance. Table 6

summarizes the effects of tenofovir disoproxil fumarate on the pharmacokinetics of

didanosine. Concomitant dosing of tenofovir disoproxil fumarate with didanosine

buffered tablets or enteric-coated capsules significantly increases the Cmax and AUC of

didanosine. When didanosine 250 mg enteric-coated capsules were administered with

tenofovir disoproxil fumarate, systemic exposures of didanosine were similar to those

seen with the 400 mg enteric-coated capsules alone under fasted conditions. The

mechanism of this interaction is unknown.

**Table 6 Drug Interactions: Pharmacokinetic Parameters for Didanosine in the**

**Presence of VIREAD**

**
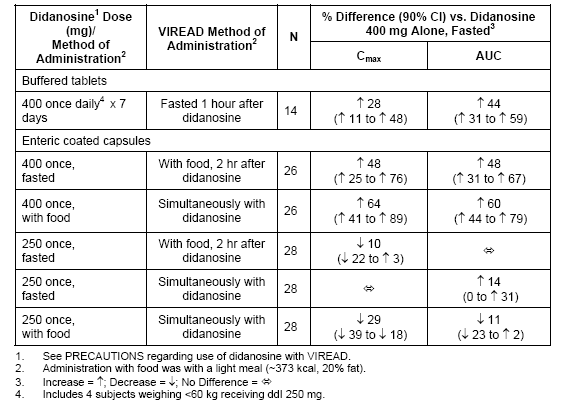
**

**INDICATIONS AND USAGE**

TRUVADA is indicated in combination with other antiretroviral agents (such as nonnucleoside

reverse transcriptase inhibitors or protease inhibitors) for the treatment of

HIV-1 infection in adults. Safety and efficacy studies using TRUVADA Tablets or using

EMTRIVA and VIREAD in combination are ongoing.

EMTRIVA and VIREAD have each been studied as part of multidrug regimens and have

been found to be safe and effective. In clinical study 303 EMTRIVA and lamivudine

(3TC) demonstrated comparable efficacy, safety and resistance patterns as part of

multidrug regimens. These data, and those from study 903, in which lamivudine and

tenofovir were used in combination, support the use of TRUVADA Tablets for the

treatment of HIV-1 infection in treatment-naïve adults **(see Description of Clinical**

**Studies and Adverse Events)**. In treatment experienced patients, the use of

TRUVADA should be guided by laboratory testing and treatment history **(see Microbiology)**.

Additional important information regarding the use of TRUVADA for the treatment of

HIV-1 infection:

- There are no study results demonstrating the effect of TRUVADA on clinical progression of HIV-1.
- It is not recommended that TRUVADA be used as a component of a triple nucleoside regimen.

**Description of Clinical Studies**

For safety and efficacy studies using EMTRIVA or VIREAD in combination with other

antiretroviral agents, also consult the Prescribing Information for these products.

Safety and efficacy studies using TRUVADA Tablets or using EMTRIVA and VIREAD in

combination are ongoing.

**EMTRIVA:**

**Study 303: EMTRIVA QD + Stable Background Therapy (SBT) Compared to**

**Lamivudine BID + SBT**

Study 303 was a 48 week, open-label, active-controlled multicenter study comparing

EMTRIVA (200 mg QD) to lamivudine, in combination with stavudine or zidovudine and

a protease inhibitor or NNRTI in 440 patients who were on a lamivudine-containing

triple-antiretroviral drug regimen for at least 12 weeks prior to study entry and had HIV-1

RNA ≤400 copies/mL.

Patients were randomized 1:2 to continue therapy with lamivudine (150 mg BID) or to

switch to EMTRIVA (200 mg QD). All patients were maintained on their stable

background regimen. Patients had a mean age of 42 years (range 22−80), 86% were

male, 64% Caucasian, 21% African-American and 13% Hispanic. Patients had a mean

baseline CD4 cell count of 527 cells/mm3 (range 37−1909), and a median baseline

plasma HIV RNA of 1.7 log10 copies/mL (range 1.7−4.0). The median duration of prior

antiretroviral therapy was 27.6 months. Treatment outcomes through 48 weeks are

presented in Table 7.

**Table 7 Outcomes of Randomized Treatment at Week 48 (Study 303)**

**
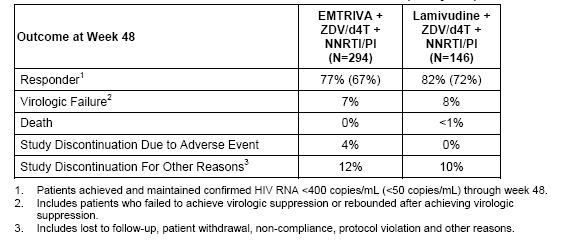
**

The mean increase from baseline in CD4 cell count was 29 cells/mm3 for the EMTRIVA

arm and 61 cells/mm3 for the lamivudine arm. Through 48 weeks, in the EMTRIVA

group 2 patients (0.7%) experienced a new CDC Class C event, compared to 2

patients (1.4%) in the lamivudine group.

**VIREAD:**

**Study 903: VIREAD + Lamivudine + Efavirenz Compared with Stavudine +**

**Lamivudine + Efavirenz**

Data through 144 weeks are reported for Study 903, a double-blind, active-controlled

multicenter study comparing VIREAD (300 mg QD) administered in combination with

lamivudine and efavirenz versus stavudine, lamivudine, and efavirenz in 600

antiretroviral-naïve patients. Patients had a mean age of 36 years (range 18–64), 74%

were male, 64% were Caucasian and 20% were Black. The mean baseline CD4 cell

count was 279 cells/mm3 (range 3–956) and median baseline plasma HIV-1 RNA was

77,600 copies/mL (range 417–5,130,000). Patients were stratified by baseline HIV-1

RNA and CD4 count. Forty-three percent of patients had baseline viral loads >100,000

copies/mL and 39% had CD4 cell counts <200 cells/mm3. Treatment outcomes through

144 weeks are presented in Table 8.

**Table 8 Outcomes of Randomized Treatment (Study 903)**

**At Week 48 At Week 144**


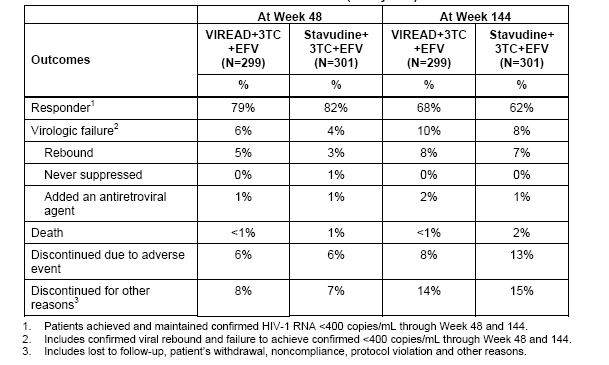


Achievement of plasma HIV-1 RNA concentrations of less than 400 copies/mL at week

144 was similar between the two treatment groups for the population stratified at

baseline on the basis of HIV-1 RNA concentration (> or ≤ 100,000 copies/mL) and CD4

cell count (< or ≥ 200 cells/mm3). Through 144 weeks of therapy, 62% and 58% of

patients in the VIREAD and stavudine arms, respectively achieved and maintained

confirmed HIV-1 RNA <50 copies/mL. The mean increase from baseline in CD4 cell

count was 263 cells/mm3 for the VIREAD arm and 283 cells/mm3 for the stavudine arm.

Through 144 weeks, eleven patients in the VIREAD group and nine patients in the

stavudine group experienced a new CDC Class C event.

Genotypic analyses of patients with virologic failure showed development of efavirenzassociated

and lamivudine-associated mutations to occur most frequently and with no

difference between the treatment arms. The K65R mutation occurred in 8 patients on

the VIREAD arm and in 2 patients on the stavudine arm. Of the 8 patients who

developed K65R in the VIREAD arm through 144 weeks, 7 of these occurred in the first

48 weeks of treatment and one at week 96. Other mutations resulting in resistance to

VIREAD were not identified in this study.

**CONTRAINDICATIONS**

TRUVADA is contraindicated in patients with previously demonstrated hypersensitivity

to any of the components of the product.

**WARNINGS**

**Lactic Acidosis/Severe Hepatomegaly with Steatosis**

Lactic acidosis and severe hepatomegaly with steatosis, including fatal cases, have

been reported with the use of nucleoside analogs alone or in combination with other

antiretrovirals. A majority of these cases have been in women. Obesity and prolonged

nucleoside exposure may be risk factors. Particular caution should be exercised when

administering nucleoside analogs to any patient with known risk factors for liver disease;

however, cases have also been reported in patients with no known risk factors.

Treatment with TRUVADA should be suspended in any patient who develops clinical or

laboratory findings suggestive of lactic acidosis or pronounced hepatotoxicity (which

may include hepatomegaly and steatosis even in the absence of marked transaminase

elevations).

**Patients with HIV and Hepatitis B Virus Coinfection**

It is recommended that all patients with HIV be tested for the presence of hepatitis B

virus (HBV) before initiating antiretroviral therapy. TRUVADA is not indicated for the

treatment of chronic HBV infection and the safety and efficacy of TRUVADA have not

been established in patients coinfected with HBV and HIV. Severe acute exacerbations

of hepatitis B have been reported in patients after the discontinuation of EMTRIVA and

VIREAD. Hepatic function should be closely monitored with both clinical and laboratory

follow up for at least several months in patients who discontinue TRUVADA and are

coinfected with HIV and HBV. If appropriate, initiation of anti-hepatitis B therapy may be

warranted.

**Renal Impairment**

Emtricitabine and tenofovir are principally eliminated by the kidney. Dosing interval

adjustment of TRUVADA is recommended in all patients with creatinine clearance

30–49 mL/min, **(see DOSAGE AND ADMINISTRATION)**. TRUVADA should not be

administered to patients with creatine clearance <30 mL/min or patients requiring

hemodialysis.

Renal impairment, including cases of acute renal failure and Fanconi syndrome (renal

tubular injury with severe hypophosphatemia), has been reported in association with the

use of VIREAD **(see ADVERSE REACTIONS-Post Marketing Experience)**. The

majority of these cases occurred in patients with underlying systemic or renal disease,

or in patients taking nephrotoxic agents, however, some cases occurred in patients

without identified risk factors.

TRUVADA should be avoided with concurrent or recent use of a nephrotoxic agent.

Patients at risk for, or with a history of, renal dysfunction and patients receiving

concomitant nephrotoxic agents should be carefully monitored for changes in serum

creatinine and phosphorus.

**PRECAUTIONS**

**Drug Interactions**

**Tenofovir disoproxil fumarate:** When tenofovir disoproxil fumarate was administered

with didanosine the Cmax and AUC of didanosine administered as either the buffered or

enteric-coated formulation increased significantly (see Table 6). The mechanism of this

interaction is unknown. Higher didanosine concentrations could potentiate didanosineassociated

adverse events, including pancreatitis, and neuropathy. In adults weighing

>60 kg, the didanosine dose should be reduced to 250 mg when it is coadministered

with TRUVADA. Data are not available to recommend a dose adjustment of didanosine

for patients weighing <60 kg. When coadministered, TRUVADA and VIDEX EC® may

be taken under fasted conditions or with a light meal (<400 kcal, 20% fat).

Coadministration of didanosine buffered tablet formulation with TRUVADA should be

under fasted conditions. **Coadministration of TRUVADA and didanosine should be**

**undertaken with caution and patients receiving this combination should be**

**monitored closely for didanosine-associated adverse events. Didanosine should**

**be discontinued in patients who develop didanosine-associated adverse events.**

Atazanavir and lopinavir/ritonavir have been shown to increase tenofovir concentrations.

The mechanism of this interaction is unknown. **Patients receiving atazanavir and**

**lopinavir/ritonavir and TRUVADA should be monitored for TRUVADA-associated**

**adverse events. TRUVADA should be discontinued in patients who develop**

**TRUVADA-associated adverse events.**

Tenofovir decreases the AUC and Cmin of atazanavir. When coadministered with

TRUVADA, it is recommended that atazanavir 300 mg is given with ritonavir 100 mg.

**Atazanavir without ritonavir should not be coadministered with TRUVADA**.

**Emtricitabine and tenofovir disoproxil fumarate:** Since emtricitabine and tenofovir

are primarily eliminated by the kidneys, coadministration of TRUVADA with drugs that

reduce renal function or compete for active tubular secretion may increase serum

concentrations of emtricitabine, tenofovir, and/or other renally eliminated drugs. Some

examples include, but are not limited to adefovir dipivoxil, cidofovir, acyclovir,

valacyclovir, ganciclovir and valganciclovir.

TRUVADA is a fixed-dose combination of emtricitabine and tenofovir disoproxil

fumarate. TRUVADA should not be coadministered with EMTRIVA or VIREAD. Due to

similarities between emtricitabine and lamivudine, TRUVADA should not be

coadministered with other drugs containing lamivudine, including COMBIVIR®, EPIVIR,

EPIVIR-HBV®, EPZICOM™, or TRIZIVIR®.

**Bone Effects**

**Tenofovir disoproxil fumarate:** In study 903 through 144 weeks, decreases from

baseline in bone mineral density (BMD) were seen at the lumbar spine and hip in both

arms of the study. At week 144, there was a significantly greater mean percentage

decrease from baseline in BMD at the lumbar spine in patients receiving VIREAD +

lamivudine + efavirenz (-2.2% ± 3.9) compared with patients receiving stavudine +

lamivudine + efavirenz (-1.0% ± 4.6). Changes in BMD at the hip were similar between

the two treatment groups (-2.8% ± 3.5 in the VIREAD group vs. -2.4% ± 4.5 in the

stavudine group). In both groups, the majority of the reduction in BMD occurred in the

first 24-48 weeks of the study and this reduction was sustained through week 144.

Twenty-eight percent of VIREAD-treated patients vs. 21% of the stavudine-treated

patients lost at least 5% of BMD at the spine or 7% of BMD at the hip. Clinically

relevant fractures (excluding fingers and toes) were reported in 4 patients in the

VIREAD group and 6 patients in the stavudine group. In addition, there were significant

increases in biochemical markers of bone metabolism (serum bone-specific alkaline

phosphatase, serum osteocalcin, serum C-telopeptide and urinary N-telopeptide) in the

VIREAD group relative to the stavudine group, suggesting increased bone turnover.

Serum parathyroid hormone levels and 1,25 Vitamin D levels were also higher in the

VIREAD group relative to the stavudine group. Except for bone specific alkaline

phosphatase, these changes resulted in values that remained within the normal range.

The effects of VIREAD-associated changes in BMD and biochemical markers on longterm

bone health and future fracture risk are unknown.

Bone monitoring should be considered for HIV infected patients who have a history of

pathologic bone fracture or are at risk for osteopenia. Although the effect of

supplementation with calcium and vitamin D was not studied, such supplementation

may be beneficial for all patients. If bone abnormalities are suspected then appropriate

consultation should be obtained.

**Fat Redistribution**

Redistribution/accumulation of body fat including central obesity, dorsocervical fat

enlargement (buffalo hump), peripheral wasting, facial wasting, breast enlargement, and

"cushingoid appearance" have been observed in patients receiving antiretroviral

therapy. The mechanism and long-term consequences of these events are currently

unknown. A causal relationship has not been established.

**Immune Reconstitution Syndrome**

Immune reconstitution syndrome has been reported in patients treated with combination

antiretroviral therapy, including VIREAD. During the initial phase of combination

antiretroviral treatment, patients whose immune system responds may develop an

inflammatory response to indolent or residual opportunistic infections (such as

*Mycobacterium avium* infection, cytomegalovirus, *Pneumocystis jirovecii* pneumonia

(PCP), or tuberculosis), which may necessitate further evaluation and treatment.

**Information for Patients**

TRUVADA is not a cure for HIV infection and patients may continue to experience

illnesses associated with HIV infection, including opportunistic infections. Patients

should remain under the care of a physician when using TRUVADA.

- Patients should be advised that:
- the use of TRUVADA has not been shown to reduce the risk of transmission of HIV to others through sexual contact or blood contamination,
- the long term effects of TRUVADA are unknown,
- TRUVADA Tablets are for oral ingestion only,
- it is important to take TRUVADA with combination therapy on a regular dosing schedule to avoid missing doses,
- redistribution or accumulation of body fat may occur in patients receiving antiretroviral therapy and that the cause and long-term health effects of these conditions are not known.
- TRUVADA should not be coadministered with EMTRIVA or VIREAD, or drugs containing lamivudine, including COMBIVIR, EPIVIR, EPIVIR-HBV, EPZICOM, or TRIZIVIR.

**Animal Toxicology**

Tenofovir and tenofovir disoproxil fumarate administered in toxicology studies to rats,

dogs and monkeys at exposures (based on AUCs) greater than or equal to 6-fold those

observed in humans caused bone toxicity. In monkeys the bone toxicity was diagnosed

as osteomalacia. Osteomalacia observed in monkeys appeared to be reversible upon

dose reduction or discontinuation of tenofovir. In rats and dogs, the bone toxicity

manifested as reduced bone mineral density. The mechanism(s) underlying bone

toxicity is unknown.

Evidence of renal toxicity was noted in 4 animal species. Increases in serum creatinine,

BUN, glycosuria, proteinuria, phosphaturia and/or calciuria and decreases in serum

phosphate were observed to varying degrees in these animals. These toxicities were

noted at exposures (based on AUCs) 2–20 times higher than those observed in

humans. The relationship of the renal abnormalities, particularly the phosphaturia, to

the bone toxicity is not known.

**Carcinogenesis, Mutagenesis, Impairment of Fertility**

**Emtricitabine:** Long-term carcinogenicity studies of emtricitabine in rats and mice are

in progress.

Emtricitabine was not genotoxic in the reverse mutation bacterial test (Ames test),

mouse lymphoma or mouse micronucleus assays.

Emtricitabine did not affect fertility in male rats at approximately 140-fold or in male and

female mice at approximately 60-fold higher exposures (AUC) than in humans given the

recommended 200 mg daily dose. Fertility was normal in the offspring of mice exposed

daily from before birth (in utero) through sexual maturity at daily exposures (AUC) of

approximately 60-fold higher than human exposures at the recommended 200 mg daily

dose.

**Tenofovir disoproxil fumarate:** Long-term oral carcinogenicity studies of tenofovir

disoproxil fumarate in mice and rats were carried out at exposures up to approximately

16 times (mice) and 5 times (rats) those observed in humans at the therapeutic dose for

HIV infection. At the high dose in female mice, liver adenomas were increased at

exposures 16 times that in humans. In rats, the study was negative for carcinogenic

findings at exposures up to 5 times that observed in humans at the therapeutic dose.

Tenofovir disoproxil fumarate was mutagenic in the in vitro mouse lymphoma assay and

negative in an in vitro bacterial mutagenicity test (Ames test). In an in vivo mouse

micronucleus assay, tenofovir disoproxil fumarate was negative when administered to

male mice.

There were no effects on fertility, mating performance or early embryonic development

when tenofovir disoproxil fumarate was administered to male rats at a dose equivalent

to 10 times the human dose based on body surface area comparisons for 28 days prior

to mating and to female rats for 15 days prior to mating through day seven of gestation.

There was, however, an alteration of the estrous cycle in female rats.

**Pregnancy**

Pregnancy Category B:

**Emtricitabine:** The incidence of fetal variations and malformations was not increased in

embryofetal toxicity studies performed with emtricitabine in mice at exposures (AUC)

approximately 60-fold higher and in rabbits at approximately 120-fold higher than

human exposures at the recommended daily dose.

**Tenofovir disoproxil fumarate:** Reproduction studies have been performed in rats and

rabbits at doses up to 14 and 19 times the human dose based on body surface area

comparisons and revealed no evidence of impaired fertility or harm to the fetus due to

tenofovir.

There are, however, no adequate and well-controlled studies in pregnant women.

Because animal reproduction studies are not always predictive of human response,

TRUVADA should be used during pregnancy only if clearly needed.

**Antiretroviral Pregnancy Registry:** To monitor fetal outcomes of pregnant women

exposed to TRUVADA, an Antiretroviral Pregnancy Registry has been established.

Healthcare providers are encouraged to register patients by calling 1-800-258-4263.

**Nursing Mothers: The Centers for Disease Control and Prevention recommend**

**that HIV-infected mothers not breast-feed their infants to avoid risking postnatal**

**transmission of HIV.** Studies in rats have demonstrated that tenofovir is secreted in

milk. It is not known whether tenofovir is excreted in human milk. It is not known

whether emtricitabine is excreted in human milk. Because of both the potential for HIV

transmission and the potential for serious adverse reactions in nursing infants, **mothers**

**should be instructed not to breast-feed if they are receiving TRUVADA.**

**Pediatric Use**

Safety and effectiveness in pediatric patients have not been established.

**Geriatric Use**

Clinical studies of EMTRIVA or VIREAD did not include sufficient numbers of subjects

aged 65 and over to determine whether they respond differently from younger subjects.

In general, dose selection for the elderly patients should be cautious, keeping in mind

the greater frequency of decreased hepatic, renal, or cardiac function, and of

concomitant disease or other drug therapy.

**ADVERSE REACTIONS**

**Clinical Trials**

**TRUVADA:** Safety and efficacy studies using TRUVADA Tablets or using EMTRIVA

and VIREAD in combination are ongoing. Two hundred eighty three HIV-1 infected

patients have received combination therapy with EMTRIVA and VIREAD with either a

non-nucleoside reverse transcriptase inhibitor or protease inhibitor for 24 to 48 weeks in

ongoing clinical studies. Based on these limited data, no new patterns of adverse

events were identified and there was no increased frequency of established toxicities.

For additional safety information about EMTRIVA or VIREAD in combination with other

antiretroviral agents, also consult the Prescribing Information for these products.

**EMTRIVA:** Adverse events that occurred in >5% of patients receiving EMTRIVA with

other antiretroviral agents in clinical trials include abdominal pain, asthenia, headache,

diarrhea, nausea, vomiting, dizziness, and rash event (including rash, pruritus,

maculopapular rash, urticaria, vesiculobullous rash, pustular rash and allergic reaction).

Approximately 1% of patients discontinued participation in the clinical studies because

of these adverse events.

Other adverse events reported include dyspepsia, arthralgia, myalgia, abnormal

dreams, depressive disorder, insomnia, neuropathy, peripheral neuritis, paresthesia,

increased cough, and rhinitis.

All adverse events were reported with similar frequency in EMTRIVA and control

treatment groups with the exception of skin discoloration which was reported with higher

frequency in the EMTRIVA treated group. Skin discoloration, manifested by

hyperpigmentation on the palms and/or soles was generally mild and asymptomatic.

The mechanism and clinical significance are unknown.

Grade 3/4 elevations of ALT and AST (>5 x ULN), bilirubin (>2.5 x ULN), creatine

kinase (>4 x ULN), decreased neutrophils (<750/mm3), pancreatic amylase (>2.0 x

ULN), serum amylase (>2 x ULN), serum glucose (<40 or >250 mg/dL), serum lipase

(>2.0 x ULN) and triglycerides (>750 mg/dL) have been reported to occur in 1–12% of

patients receiving EMTRIVA.

**VIREAD:** Adverse events that occurred in >5% of patients receiving VIREAD with other

antiretroviral agents in clinical trials included: headache, nausea, diarrhea, vomiting,

rash event (including rash, pruritus, maculopapular rash, urticaria, vesiculobullous rash,

and pustular rash), and depression. Less than 1% of patients discontinued participation

in the clinical studies because of gastrointestinal adverse events.

Other adverse events include asthenia, pain, abdominal pain, back pain, chest pain,

fever, flatulence, dizziness, dyspepsia, anorexia, arthralgia, lipodystrophy, insomnia,

peripheral neuropathy (including peripheral neuritis and neuropathy), anxiety,

pneumonia, sweating, myalgia and weight loss.

Grade 3/4 elevations of ALT and AST (>5 x ULN), creatine kinase (>4 x ULN), serum

amylase (>2 x ULN), urine glucose (≥3+), serum glucose (>250 mg/dL) and serum

triglycerides (>750 mg/dL), hematuria (>100 RBC/HPF), total cholesterol (> 300 mg/dL),

and decreased neutrophils (<750/mm3) have been reported to occur in 2–12% of

patients receiving VIREAD.

**Post Marketing Experience**

**EMTRIVA:** No additional events have been identified for inclusion in this section.

**VIREAD:** In addition to adverse events reported from clinical trials, the following events

have been identified during post-approval use of VIREAD. Because they are reported

voluntarily from a population of unknown size, estimates of frequency cannot be made.

These events have been chosen for inclusion because of a combination of their

seriousness, frequency of reporting or potential causal connection to VIREAD.

IMMUNE SYSTEM DISORDERS

Allergic reaction

METABOLISM AND NUTRITION DISORDERS

Hypophosphatemia, Lactic acidosis

RESPIRATORY, THORACIC, AND MEDIASTINAL DISORDERS

Dyspnea

GASTROINTESTINAL DISORDERS

Abdominal pain, Increased amylase, Pancreatitis

HEPATOBILIARY DISORDERS

Increased liver enzymes, Hepatitis

RENAL AND URINARY DISORDERS

Renal insufficiency, Renal failure, Acute renal failure, Fanconi syndrome, Proximal

tubulopathy, Proteinuria, Increased creatinine, Acute tubular necrosis, Nephrogenic

diabetes insipidus

**OVERDOSAGE**

If overdose occurs the patient must be monitored for evidence of toxicity, and standard

supportive treatment applied as necessary.

**Emtricitabine:** Limited clinical experience is available at doses higher than the

therapeutic dose of EMTRIVA. In one clinical pharmacology study single doses of

emtricitabine 1200 mg were administered to 11 patients. No severe adverse reactions

were reported.

Hemodialysis treatment removes approximately 30% of the emtricitabine dose over a 3-

hour dialysis period starting within 1.5 hours of emtricitabine dosing (blood flow rate of

400 mL/min and a dialysate flow rate of 600 mL/min). It is not known whether

emtricitabine can be removed by peritoneal dialysis.

**Tenofovir disoproxil fumarate:** Limited clinical experience at doses higher than the

therapeutic dose of VIREAD 300 mg is available. In one study, 600 mg tenofovir

disoproxil fumarate was administered to 8 patients orally for 28 days, and no severe

adverse reactions were reported. The effects of higher doses are not known.

Tenofovir is efficiently removed by hemodialysis with an extraction coefficient of

approximately 54%. Following a single 300 mg dose of VIREAD, a four-hour

hemodialysis session removed approximately 10% of the administered tenofovir dose.

**DOSAGE AND ADMINISTRATION**

The dose of TRUVADA is one tablet (containing 200 mg of emtricitabine and 300 mg of

tenofovir disoproxil fumarate) once daily taken orally with or without food.

**Dose Adjustment for Renal Impairment:**

Significantly increased drug exposures occurred when EMTRIVA or VIREAD were

administered to patients with moderate to severe renal impairment **(see EMTRIVA or**

**VIREAD Package Insert)**. Therefore, the dosing interval of TRUVADA should be

adjusted in patients with baseline creatinine clearance 30–49 mL/min using the

recommendations in Table 9. The safety and effectiveness of these dosing interval

adjustment recommendations have not been clinically evaluated, therefore, clinical

response to treatment and renal function should be closely monitored in these patients.

**Table 9 Dosage Adjustment for Patients with Altered Creatinine Clearance**

**
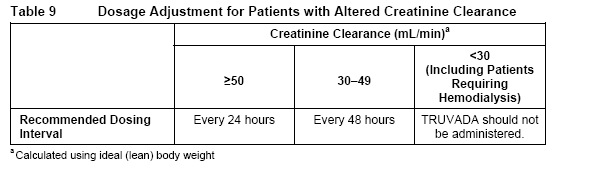
**

**HOW SUPPLIED**

TRUVADA is available as tablets. Each tablet contains 200 mg of emtricitabine and

300 mg of tenofovir disoproxil fumarate (which is equivalent to 245 mg of tenofovir

disoproxil). The tablets are blue, capsule-shaped, film-coated, debossed with

“GILEAD” on one side and with “701” on the other side. Each bottle contains 30 tablets

(NDC 61958-0701-1) and a desiccant (silica gel canister or sachet) and is closed with a

child-resistant closure.

Store at 25 °C (77 °F), excursions permitted to 15–30 °C (59–86 °F).

- Keep container tightly closed
- Dispense only in original container
- Do not use if seal over bottle opening is broken or missing.

Gilead Sciences, Inc.

Foster City, CA 94404

May 2005

EMTRIVA, TRUVADA, and VIREAD are trademarks of Gilead Sciences, Inc. REYATAZ

and VIDEX are trademarks of Bristol-Myers Squibb. COMBIVIR, EPIVIR, EPIVIR-HBV,

EPZICOM, and TRIZIVIR are trademarks of GlaxoSmithKline.

©2004–2005 Gilead Sciences, Inc.

21-752-GS18

**
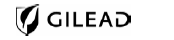
**

**Patient Information**

**TRUVADA® (tru-vah-dah) Tablets**

Generic name: emtricitabine and tenofovir disoproxil fumarate

(em tri SIT uh bean and te NOE’ fo veer dye soe PROX il FYOU-mar-ate)

Read the Patient Information that comes with TRUVADA before you start taking it and

each time you get a refill. There may be new information. This information does not take

the place of talking to your healthcare provider about your medical condition or

treatment. You should stay under a healthcare provider’s care when taking TRUVADA.

**Do not change or stop your medicine without first talking with your healthcare**

**provider**. Talk to your healthcare provider or pharmacist if you have any questions

about TRUVADA.

**What is the most important information I should know about TRUVADA?**

- **Some people who have taken medicine like TRUVADA (nucleoside analogs)**

**have developed a serious condition called lactic acidosis** (build up of an acid in

the blood). Lactic acidosis can be a medical emergency and may need to be treated

in the hospital. **Call your healthcare provider right away if you get the following**

**signs or symptoms of lactic acidosis.**

- You feel very weak or tired.
- You have unusual (not normal) muscle pain.
- You have trouble breathing.
- You have stomach pain with nausea and vomiting.
- You feel cold, especially in your arms and legs.
- You feel dizzy or lightheaded.
- You have a fast or irregular heartbeat.
- **Some people who have taken medicines like TRUVADA have developed**

**serious liver problems called hepatotoxicity**, with liver enlargement

(hepatomegaly) and fat in the liver (steatosis). **Call your healthcare provider right**

**away if you get the following signs or symptoms of liver problems.**

- Your skin or the white part of your eyes turns yellow (jaundice).
- Your urine turns dark.
- Your bowel movements (stools) turn light in color.
- You don’t feel like eating food for several days or longer.
- You feel sick to your stomach (nausea).
- You have lower stomach area (abdominal) pain.
- **You may be more likely to get lactic acidosis or liver problems** if you are

female, very overweight (obese), or have been taking nucleoside analog medicines,

like TRUVADA, for a long time.

- **TRUVADA is not for the treatment of Hepatitis B Virus infection**. Patients

infected with both HBV and human immunodeficiency virus (HIV) who take

TRUVADA need close medical follow-up for several months after stopping treatment

with TRUVADA. Follow-up includes medical exams and blood tests to check for

HBV that could be getting worse. **Patients with Hepatitis B Virus infection, who**

**take TRUVADA and then stop it, may get “flare-ups” of their hepatitis. A**

**“flare-up” is when the disease suddenly returns in a worse way than before.**

**What is TRUVADA?**

TRUVADA is a type of medicine called an HIV (human immunodeficiency virus)

nucleoside analog reverse transcriptase inhibitor (NRTI). TRUVADA contains 2

medicines, EMTRIVA® (emtricitabine) and VIREAD® (tenofovir disoproxil fumarate, or

tenofovir DF) combined in one pill. TRUVADA is always used with other anti-HIV

medicines to treat people with HIV infection. TRUVADA is for adults age 18 and older.

TRUVADA has not been studied in children under age 18 or adults over age 65.

HIV infection destroys CD4 (T) cells, which are important to the immune system. The

immune system helps fight infection. After a large number of T cells are destroyed,

acquired immune deficiency syndrome (AIDS) develops.

TRUVADA helps block HIV reverse transcriptase, a chemical in your body (enzyme)

that is needed for HIV to multiply. TRUVADA lowers the amount of HIV in the blood

(viral load). TRUVADA may also help to increase the number of T cells (CD4 cells).

Lowering the amount of HIV in the blood lowers the chance of death or infections that

happen when your immune system is weak (opportunistic infections).

**TRUVADA does not cure HIV infection or AIDS.** The long-term effects of TRUVADA

are not known at this time. People taking TRUVADA may still get opportunistic

infections or other conditions that happen with HIV infection. Opportunistic infections

are infections that develop because the immune system is weak. Some of these

conditions are pneumonia, herpes virus infections, and *Mycobacterium avium complex*

(MAC) infection. **It is very important that you see your healthcare provider**

**regularly while taking TRUVADA.**

**TRUVADA does not lower your chance of passing HIV to other people through**

**sexual contact, sharing needles, or being exposed to your blood.** For your health

and the health of others, it is important to always practice safer sex by using a latex or

polyurethane condom or other barrier to lower the chance of sexual contact with semen,

vaginal secretions, or blood. Never use or share dirty needles.

**Who should not take TRUVADA?**

Do not take TRUVADA if you are allergic to TRUVADA or any of its ingredients. The

active ingredients of TRUVADA are emtricitabine and tenofovir DF. See the end of this

leaflet for a complete list of ingredients.

**What should I tell my healthcare provider before taking TRUVADA?**

**Tell your healthcare provider if you:**

- **are pregnant or planning to become pregnant.** We do not know if TRUVADA can

harm your unborn child. You and your healthcare provider will need to decide if

TRUVADA is right for you. If you use TRUVADA while you are pregnant, talk to your

healthcare provider about how you can be on the TRUVADA Antiviral Pregnancy

Registry.

- **are breast-feeding.** You should not breast feed if you are HIV-positive

because of the chance of passing the HIV virus to your baby. Also, it is not known if

TRUVADA can pass into your breast milk and if it can harm your baby. If you are a

woman who has or will have a baby, talk with your healthcare provider about the

best way to feed your baby.

- **have kidney problems or are undergoing kidney dialysis treatment.**
- **have bone problems.**
- **have liver problems including Hepatitis B Virus infection.**

**Tell your healthcare provider about all the medicines you take**, including

prescription and non-prescription medicines, vitamins, and herbal supplements.

Especially tell your healthcare provider if you take:

- COMBIVIR®, EMTRIVA, EPIVIR®, EPIVIR-HBV®, EPZICOM™, TRIZIVIR®, or

VIREAD. **TRUVADA should not be used with those medicines.**

- Drugs that contain didanosine (VIDEX®, VIDEX EC®). Tenofovir DF (a

component of TRUVADA) may increase the amount of VIDEX in your blood. **You**

**may need to be followed more carefully if you are taking TRUVADA and**

**VIDEX together**.

- REYATAZTM (atazanavir sulfate) or KALETRA® (lopinavir/ritonavir). These

medicines may increase the amount of tenofovir DF (a component of TRUVADA)

in your blood, which could result in more side effects. You may need to be

followed more carefully if you are taking TRUVADA and REYATAZ or KALETRA

together.

Keep a complete list of all the medicines that you take. Make a new list when medicines

are added or stopped. Give copies of this list to all of your healthcare providers and

pharmacist **every** time you visit your healthcare provider or fill a prescription.

**How should I take TRUVADA?**

- Take TRUVADA exactly as your healthcare provider prescribed it. Follow the

directions from your healthcare provider, exactly as written on the label.

- The usual dose of TRUVADA is 1 tablet once a day. TRUVADA is always used with

other anti-HIV medicines. If you have kidney problems, you may need to take

TRUVADA less often.

- TRUVADA may be taken with or without a meal. Food does not affect how

TRUVADA works. Take TRUVADA at the same time each day.

- If you forget to take TRUVADA, take it as soon as you remember that day. **Do not**

take more than 1 dose of TRUVADA in a day. **Do not** take 2 doses at the same

time. Call your healthcare provider or pharmacist if you are not sure what to do. **It**

**is important that you do not miss any doses of TRUVADA or your anti-HIV**

**medicines.**

- When your TRUVADA supply starts to run low, get more from your healthcare

provider or pharmacy. This is very important because the amount of virus in your

blood may increase if the medicine is stopped for even a short time. The virus may

develop resistance to TRUVADA and become harder to treat.

- Do not change your dose or stop taking TRUVADA without first talking with your

healthcare provider. Stay under a healthcare provider’s care when taking

TRUVADA.

- If you take too much TRUVADA, call your local poison control center or emergency

room right away.

**What should I avoid while taking TRUVADA?**

- **Do not breast-feed.** See “What should I tell my healthcare provider before taking

TRUVADA?”

- **Avoid doing things that can spread HIV infection** since TRUVADA does not stop

you from passing the HIV infection to others.

- **Do not share needles or other injection equipment.**
- **Do not share personal items that can have blood or body fluids on them,**

**like toothbrushes or razor blades.**

- **Do not have any kind of sex without protection.** Always practice safer sex by

using a latex or polyurethane condom or other barrier to reduce the chance of

sexual contact with semen, vaginal secretions, or blood.

- COMBIVIR, EMTRIVA, EPIVIR, EPIVIR-HBV, EPZICOM, TRIZIVIR, or VIREAD.

**TRUVADA should not be used with these medicines.**

**What are the possible side effects of TRUVADA?**

**TRUVADA may cause the following serious side effects** (see “What is the most

important information I should know about TRUVADA?”):

- **Lactic acidosis** (buildup of an acid in the blood). Lactic acidosis can be a medical

emergency and may need to be treated in the hospital. **Call your doctor right**

**away if you get signs of lactic acidosis.** (See “What is the most important

information I should know about TRUVADA?”)

- **Serious liver problems (hepatotoxicity**)**,** with liver enlargement (hepatomegaly)

and fat in the liver (steatosis). Call your healthcare provider right away if you get any

signs of liver problems. (See “What is the most important information I should know

about TRUVADA?”)

- **“Flare-ups” of Hepatitis B Virus infection,** in which the disease suddenly returns

in a worse way than before, can occur if you stop taking TRUVADA. Your

healthcare provider will monitor your condition for several months after stopping

TRUVADA if you have both HIV and HBV infection. TRUVADA is not for the

treatment of Hepatitis B Virus infection.

- **Kidney problems** If you have had kidney problems in the past or take other

medicines that can cause kidney problems, your healthcare provider should do

regular blood tests to check your kidneys.

- **Changes in bone mineral density (thinning bones)** It is not known whether longterm

use of TRUVADA will cause damage to your bones. If you have had bone

problems in the past, your healthcare provider may need to do tests to check your

bone mineral density or may prescribe medicines to help your bone mineral density.

Other side effects with TRUVADA when used with other anti-HIV medicines include:

- Changes in body fat have been seen in some patients taking TRUVADA and other

anti-HIV medicines. These changes may include increased amount of fat in the

upper back and neck (“buffalo hump”), breast, and around the main part of your

body (trunk). Loss of fat from the legs, arms and face may also happen. The cause

and long term health effect of these conditions are not known at this time.

The most common side effects of EMTRIVA or VIREAD when used with other anti-HIV

medicines are: dizziness, diarrhea, nausea, vomiting, headache, rash, and gas. Skin

discoloration (small spots or freckles) may also happen with TRUVADA.

These are not all the side effects of TRUVADA. This list of side effects with TRUVADA

is **not complete** at this time because TRUVADA is still being studied. If you have

questions about side effects, ask your healthcare provider. Report any new or

continuing symptoms to your healthcare provider right away. Your healthcare provider

may be able to help you manage these side effects.

**How do I store TRUVADA?**

- **Keep TRUVADA and all other medicines out of reach of children.**
- Store TRUVADA at room temperature 77 °F (25 °C).
- Keep TRUVADA in its original container and keep the container tightly closed.
- Do not keep medicine that is out of date or that you no longer need. If you throw any

medicines away make sure that children will not find them.

**General information about TRUVADA:**

Medicines are sometimes prescribed for conditions that are not mentioned in patient

information leaflets. Do not use TRUVADA for a condition for which it was not

prescribed. Do not give TRUVADA to other people, even if they have the same

symptoms you have. It may harm them.

This leaflet summarizes the most important information about TRUVADA. If you would

like more information, talk with your healthcare provider. You can ask your healthcare

provider or pharmacist for information about TRUVADA that is written for health

professionals. For more information, you may also call 1-800-GILEAD-5 or access the

TRUVADA website at www.TRUVADA.com.

Do not use TRUVADA if seal over bottle opening is broken or missing.

**What are the ingredients of TRUVADA?**

**Active Ingredients:** emtricitabine and tenofovir DF

**Inactive Ingredients:** Croscarmellose sodium, lactose monohydrate, magnesium

stearate, microcrystalline cellulose, and pregelatinized starch (gluten free). The tablets

are coated with Opadry II Blue Y-30-10701 containing FD&C Blue #2 aluminum lake,

hypromellose, lactose monohydrate, titanium dioxide and triacetin.

℞ **Only**

May 2005

EMTRIVA, TRUVADA, and VIREAD are trademarks of Gilead Sciences, Inc. REYATAZ

and VIDEX are trademarks of Bristol-Myers Squibb. KALETRA is a trademark of Abbott

Laboratories. COMBIVIR, EPIVIR, EPIVIR-HBV, EPZICOM, and TRIZIVIR are

trademarks of GlaxoSmithKline.

©2004-2005 Gilead Sciences, Inc.

21-752-GS18

**
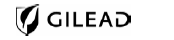
**

**Nonclinical Studies**

Refer to the Truvada package insert (Section ) or the local country labeling (as applicable) for virology and animal toxicology data. This section provides new information that supplements the information in the labeling.

## Toxicology

### Repeated Dose Toxicity

#### Emtricitabine/Tenofovir DF: A 14-Day Oral Gavage Toxicity Study Comparing Non-degraded and Degraded FTC/TDF in Sprague-Dawley Rats

Rats were administered non­degraded or degraded emtricitabine/tenofovir DF in suspension vehicle at doses of 20/30, 67/100 or 200/300 mg/kg/day. There were no deaths related to treatment with emtricitabine/tenofovir DF (degraded or non­degraded). Clinical signs were limited to dose‑related postdosing salivation. There were no treatment-related effects on body weight, food consumption, hematology, biochemistry, or urinalysis parameters. Organ weight evaluation revealed marginal increases in the weights of adrenal glands in most groups; however, no gross or histological changes were identified that might account for this increased adrenal weight. No treatment-related gross changes were observed at necropsy. Microscopic evaluation revealed hyperplasia of the anterior duodenal mucosa overlying Brunner’s glands, which was seen at the high‑dose level in 7 of 10 animals treated with non-degraded emtricitabine/tenofovir DF and in 2 of 10 animals receiving degraded emtricitabine/tenofovir DF and was considered to be treatment-related. Duodenal hyperplasia has been noted in previous studies of tenofovir DF alone.

#### A 4-Week Oral Gavage Toxicity and Toxicokinetic Study With Emtricitabine/Tenofovir Disoproxil Fumarate (FTC/TDF) in Male Dogs With a 4-Week Recovery Period

A 4‑week toxicity study was conducted with emtricitabine and tenofovir DF in dogs to examine the possible exacerbation of renal toxicity with combination treatment and to assess possible effects on the immune system. Male dogs were treated with vehicle, emtricitabine alone (20 mg/kg/day), tenofovir DF alone (30 mg/kg/day), or a low dose (2/3 mg/kg/day) or high dose (20/30 mg/kg/day) of the combination. No adverse effects were observed in the emtricitabine alone group or the low dose combination group. No remarkable changes were observed for immunophenotyping or natural killer cell assay values for any treatment group. Tenofovir DF at 30 mg/kg alone or in combination with 20 mg/kg emtricitabine caused minimally increased activated partial thromboplastin time and creatinine. Minimal tubular epithelial necrosis and slight to moderate tubular epithelial regeneration were seen in animals given tenofovir DF at 30 mg/kg alone or in combination with 20 mg/kg emtricitabine. There were no overall differences in the incidences and mean severities of the renal findings between the two groups. Renal findings were reversible after a 4‑week recovery period (examined for combination only). Systemic exposure (AUC) was not altered with combination dosing when compared with the agents dosed individually. The no-observed-adverse-effect level (NOAEL) for the combination of emtricitabine/tenofovir DF is 2/3 mg/kg/day in dogs.

### Mutagenicity and Carcinogenicity

A fixed ratio of 2:3 (emtricitabine:tenofovir DF) was evaluated in two in vitro genotoxicity studies to reflect the ratio of the active substances in the clinical product (Truvada).

The combination of emtricitabine and tenofovir DF was negative in the in vitro bacterial assay (Ames Assay). The combination was positive for inducing forward mutations in the in vitro mouse lymphoma cell assay in the presence or absence of S9 metabolic activation. The increases in mutant frequency occurred at concentrations similar to that observed with tenofovir DF alone.

### Mitochondrial Toxicity

In vitro studies were conducted to assess the potential for mitochondrial toxicity of individual nucleoside or nucleotide analog reverse transcriptase inhibitors (NRTIs) and specified dual and triple combinations of NRTIs in HepG2 cells. Cells were treated for up to 25 days with concentrations of NRTIs equal to 1 time and 10 times the maximal therapeutic plasma levels. Assay endpoints included cell growth, extracellular production of lactic acid, relative cellular content of mitochondrial DNA (mtDNA) and mtDNA-encoded cytochrome c oxidase II (COX II), and intracellular lipid accumulation.

Tenofovir (TFV), lamivudine (3TC), or emtricitabine (FTC) alone had no time-dependent or concentration-dependent effects on cytotoxicity (cell counts) or mitochondrial parameters in HepG2 liver cells. In contrast, didanosine (ddI) and stavudine (d4T) had consistent effects on all measured parameters indicative of mitochondrial toxicity, with marked decreases in mtDNA and COX II levels in particular. Abacavir (ABC) and zidovudine (AZT) caused increases in lactate production and lipid content, but had no apparent effect on mtDNA or COX II levels. The dual combinations of high‑dose 3TC + AZT and FTC + AZT with or without TFV appeared to have greater cytotoxicity than the agents alone. Combination treatment had little or no additive effect on lactate or on mtDNA and COX II. Effects on mitochondrial parameters observed with the various combinations were generally attributed to AZT, ddI, or d4T, which caused alterations when administered alone. An amelioration of effects on mtDNA or COX II levels was observed when high‑dose TFV and 3TC was combined with high‑dose ddI. Combinations of NRTIs did not appear to increase lipid levels above those caused by individual agents.

These studies demonstrate that the potential of FTC and TFV to interfere with mitochondrial functions is low, whether administered alone or in combination with other licensed NRTIs. While some modest increases in cytotoxicity were seen in high‑dose combinations with FTC or TFV and other NRTIs, ddI and d4T (alone or in combination) had substantial dose-related and time-related effects on mtDNA levels and, consequently, on the cellular content of the mtDNA-encoded protein COX II and lactate production. There was no evidence that the specific combination of TFV and ddI resulted in greater cytotoxicity or mitochondrial changes relative to the effects of ddI alone. AZT effects were manifested as an increase in extracellular lactate without significant effects on mtDNA. Dual and triple combinations of NRTIs did not generally cause additive or synergistic effects on the measured mitochondrial endpoints.

### Toxicology Conclusions

The NOAEL for the emtricitabine/tenofovir DF combination was 67/100 mg/kg/day in rats and 2/3 mg/kg/day in dogs. These NOAELs are similar to those reported previously for tenofovir DF alone.

No exacerbation of target organ toxicity or mutagenicity was apparent when emtricitabine and tenofovir DF were administered together compared with each agent alone.

On the basis of in vitro assays, emtricitabine and tenofovir, whether administered alone or in combination, demonstrate a low potential for interfering with mitochondrial function.

# Clinical Studies

Refer to the Truvada package insert (Section ) or the local country labeling (as applicable) for clinical pharmacology, safety, and efficacy data. This section provides new information that supplements the information in the labeling.

Two clinical studies (Studies GS‑01‑934 and M02‑418) not described in the Truvada package insert have been conducted in which emtricitabine and tenofovir DF were administered concurrently. Results of both studies are summarized in this section.

## Study GS-01-934

Study GS‑01‑934 is an ongoing, 144‑week, randomized, open‑label, parallel, multicenter, active‑controlled study to assess the noninferiority of the regimen of efavirenz + emtricitabine + tenofovir DF relative to the regimen of efavirenz + Combivir (lamivudine/zidovudine) in antiretroviral-naive, HIV‑1 infected patients. The regimen of efavirenz once daily and Combivir (lamivudine/zidovudine) twice daily serves as the active control treatment and is compared with a once‑daily regimen of efavirenz, emtricitabine, and tenofovir DF. Randomization was performed in a ratio of 1:1 to the two treatment groups, and patients were stratified on the basis of baseline CD4+ cell count (≥ 200 or < 200 cells/mm^3^). If efavirenz-associated central nervous system toxicities occurred, nevirapine could be substituted for efavirenz.

The primary efficacy endpoint for this study was the achievement and maintenance of confirmed HIV‑1 RNA < 400 copies/mL through Week 48, as defined by the US Food and Drug Administration (FDA) time-to-loss-of-virologic-response (TLOVR) algorithm {5059}. The primary efficacy analysis (modified intent-to-treat [MITT] analysis set) excluded patients who were antiretroviral treatment-experienced or had primary nonnucleoside reverse transcriptase inhibitor (NNRTI) resistance mutations at baseline.

A two‑sided 95% confidence interval (CI) for the difference in the primary endpoint between the two treatment groups (emtricitabine + tenofovir DF minus Combivir), stratified by baseline CD4+ cell count, was constructed. The regimen containing emtricitabine + tenofovir DF was declared noninferior to the regimen containing Combivir if the lower confidence bound was greater than −0.13.

### Demographics and Baseline Characteristics

Of the 509 patients in the intent-to-treat (ITT) analysis set, 14% were female, 59% were white, 23% were black, and 16% were Hispanic. The median age was 37 years (range, 18 to 80 years). Mean baseline plasma HIV‑1 RNA concentrations were approximately 5 log_10_ copies/mL for both the emtricitabine + tenofovir DF and Combivir groups, and mean baseline CD4+ cell counts were 246 and 245 cells/mm^3^, respectively.

### Efficacy Results

The proportion of responders was significantly higher in the emtricitabine + tenofovir DF group compared with the Combivir group for the primary efficacy analysis, as well as for the ITT analysis set and achievement and maintenance of confirmed HIV‑1 RNA < 50 copies/mL through Week 48 for both the MITT and ITT analysis sets (Table 1).

Table 1. Treatment Outcomes at Week 48 in Study GS-01-934 (TLOVR Analysis)

|  | EFV + FTC + TDF | | EFV + Combivir | |
| --- | --- | --- | --- | --- |
| Treatment Outcome at Week 48 | n/N | % | n/N | % |
| Responder (HIV‑1 RNA < 400 copies/mL), MITT | 206/244 | 84% | 177/243 | 73% |
| p‑value^a^ | 0.002 | | | |
| Difference (95% CI)^b^ | 11% (4% to 19%) | | | |
| Responder (HIV‑1 RNA < 400 copies/mL), ITT | 207/255 | 81% | 179/254 | 71% |
| p‑value^a^ | 0.005 | | | |
| Difference (95% CI)^b^ | 11% (3% to 18%) | | | |
| Responder (HIV‑1 RNA < 50 copies/mL), MITT | 194/244 | 80% | 171/243 | 70% |
| p‑value^a^ | 0.021 | | | |
| Difference (95% CI)^b^ | 9% (2% to 17%) | | | |
| Responder (HIV‑1 RNA < 50 copies/mL), ITT | 195/255 | 77% | 173/254 | 68% |
| p‑value^a^ | 0.034 | | | |
| Difference (95% CI)^b^ | 9% (1% to 16%) | | | |

EFV, efavirenz; FTC, emtricitabine; TDF, tenofovir disoproxil fumarate; MITT, modified intent-to-treat; CI, confidence interval; ITT, intent-to-treat

a The p‑value was based on the Cochran-Mantel-Haenszel test stratified on baseline CD4+ cell count.

b The difference and 95% confidence interval were stratum weighted on baseline CD4+ cell count using normal approximation.

Source: GS‑01‑934 Clinical Study Report

Because the 95% confidence intervals (MITT and ITT) for the treatment effect lie entirely above the limit of ≥ −0.13 required for demonstration of noninferiority and, furthermore, lie entirely above zero, and because the p‑values support rejection of the null hypothesis of no difference between the treatment groups, the efavirenz + emtricitabine + tenofovir DF regimen was concluded to be superior to the efavirenz + Combivir regimen.

Similar results were observed in the ITT analysis for which the last plasma HIV‑1 RNA concentration before a change in antiretroviral therapy (ART switch) was carried forward for patients with primary NNRTI resistance mutations at baseline.

The proportions of patients with loss of virologic response and proportions of patients with pure virologic failure (plasma HIV‑1 RNA lower limit of quantitation [LLQ], 400 copies/mL) by Week 48 were significantly lower for the emtricitabine + tenofovir DF group compared with the Combivir group (Kaplan-Meier estimates, Table 2). Time to loss-of-virologic response and time to pure virologic failure were defined as follows:

- Time to loss-of-virologic response (HIV‑1 RNA cutoff at LLQ) for patients who achieved a confirmed virologic response (two consecutive HIV‑1 RNA below LLQ) before study drug discontinuation was the time to the earliest premature study regimen discontinuation, or confirmed HIV‑1 RNA above LLQ (two consecutive HIV‑1 RNA ≥ LLQ, or the last HIV‑1 RNA ≥ LLQ followed by loss to follow‑up). Patients who did not achieve a confirmed virologic response before study drug discontinuation were assumed to have lost virologic response on study Day 1.
- Time to pure virologic failure (HIV‑1 RNA cutoff at LLQ) for patients who achieved a confirmed virologic response (two consecutive HIV‑1 RNA below LLQ) was the time to the earliest date of confirmed HIV‑1 RNA above LLQ (two consecutive HIV‑1 RNA ≥ LLQ or the last HIV‑1 RNA ≥ LLQ followed by premature study discontinuation). Patients who did not achieve a confirmed HIV‑1 RNA below LLQ were assumed to have failed on study Day 1.

Table 2. Kaplan-Meier Estimates for Loss of Virologic Response and Pure Virologic Failure by Week 48 (Study GS-001-934, ITT)

| Kaplan-Meier Estimate | EFV + FTC + TDF (N = 255) | EFV + Combivir (N = 254) | p‑value^a^ |
| --- | --- | --- | --- |
| Loss of Virologic Response |  |  |  |
| HIV‑1 RNA, LLQ = 400 copies/mL | 19% | 30% | 0.003 |
| HIV‑1 RNA, LLQ = 50 copies/mL | 23% | 32% | 0.046 |
| Pure Virologic Failure |  |  |  |
| HIV‑1 RNA, LLQ = 400 copies/mL | 9% | 16% | 0.026 |
| HIV‑1 RNA, LLQ = 50 copies/mL | 16% | 24% | 0.063 |

EFV, efavirenz; FTC, emtricitabine; TDF, tenofovir disoproxil fumarate; LLQ, lower limit of quantitation

a The p‑value is based on the log‑rank test stratified on baseline CD4+ cell count.

Source: GS‑01‑934 Clinical Study Report

Mean decreases from baseline to Week 48 in plasma HIV‑1 RNA were similar between the emtricitabine + tenofovir DF group (3.31 log_10_ copies/mL) and the Combivir group (3.26 log_10_ copies/mL).

CD4+ cell count increased over time for both treatment groups (). Mean increases from baseline to Week 48 were significantly greater for the emtricitabine + tenofovir DF group (190 cells/mm^3^) compared with the Combivir group (158 cells/mm^3^, p = 0.002).

| Figure 1. Mean Change From Baseline (95% CI) in CD4+ Cell Count in Study GS-01-934 (As-treated Analysis Set) |
| --- |

Source: GS‑01‑934 Clinical Study Report

Efficacy in Subgroups

Baseline Plasma HIV‑1 RNA and CD4+ Cell Count

In Study GS‑01‑934, virologic response (achievement and maintenance of plasma HIV‑1 RNA < 400 copies/mL, MITT analysis set) was significantly greater for the efavirenz + emtricitabine + tenofovir DF regimen compared with the efavirenz + Combivir regimen in patients with high baseline CD4+ cell counts (≥ 200 cells/mm^3^, p = 0.015) and patients with either low or high baseline viral loads (plasma HIV‑1 RNA ≤ 100,000 or > 100,000 copies/mL; p = 0.039 or p = 0.017, respectively). For patients with baseline CD4+ cell counts ≥ 200 cells/mm^3^, a significantly higher proportion in the emtricitabine + tenofovir DF group compared with the Combivir group also achieved and maintained plasma HIV‑1 RNA < 50 copies/mL (p = 0.041) through Week 48.

HIV‑1 Subtype

An additional analysis by HIV‑1 subtypes was conducted {7922}. The distribution of HIV‑1 subtypes in study patients is summarized in Table 3. All non‑B HIV‑1 isolates in both treatment groups were considered wild-type at baseline.

Table 3. Distribution of HIV‑1 Subtypes (Study GS-01-934, ITT)

| HIV‑1 Subtype | EFV + FTC + TDF (N = 255) n (%) | EFV + Combivir (N = 254) n (%) |
| --- | --- | --- |
| B | 236 (92.5%) | 236 (93%) |
| Non‑B^a^ | 15 (6%) | 13 (5%) |
| AG | 4 (1.5%) | 4 (1.6%) |
| C | 5 (2%) | 0 |
| AE | 1 (< 1%) | 3 (1.2%) |
| A | 2 (< 1%) | 2 (< 1%) |
| ND^b^ | 4 (1.5%) | 5 (2%) |

a All others detected (BF, D, G, and complex) were represented by ≤ 2 patients (i.e., < 1% of ITT subset).

b Subtype could not be determined because of technical failure of baseline genotyping.

Source: {7922}

Treatment response rates were similar for patients with HIV‑1 subtype B and those with non‑B HIV‑1 subtypes. Of the patients with non‑B HIV‑1, 87% (13/15) in the emtricitabine + tenofovir DF group (p = 0.74 versus HIV‑1 B subtypes [81%, 191/236]) and 69% (9/13) in the Combivir group (p = 1.00 versus HIV‑1 B subtypes [70%, 166/236]) achieved and maintained confirmed HIV‑1 RNA < 400 copies/mL through Week 48, as defined by the FDA TLOVR algorithm.

Virology Results

At Week 48, genotypic and phenotypic resistance analyses were performed for all patients who had confirmed plasma HIV‑1 RNA > 400 copies/mL by Week 48 or early study drug discontinuation, corresponding to 5% of patients in the emtricitabine + tenofovir DF group and 10% of patients in the Combivir group. Genotypic resistance to efavirenz, predominantly the K103N mutation, was the most common form of resistance that developed; it occurred as a single mutation or in combination with the M184V/I mutation, which causes reduced susceptibility to both emtricitabine and lamivudine. The K103N mutation was observed in 9 of 12 patients in the emtricitabine + tenofovir DF group and 16 of 22 patients in the Combivir group. The M184V/I mutation was the most common NRTI‑associated mutation; it developed in 2 of 12 patients in the emtricitabine + tenofovir DF group and in 7 of 22 patients in the Combivir group. The M184V/I mutation occurred predominantly in combination with efavirenz resistance. One patient in the Combivir group developed a thymidine analog-associated mutation, and no patient in either treatment group developed the K65R mutation, which is associated with reduced susceptibility to tenofovir DF. No novel patterns of mutations associated with reduced phenotypic susceptibility to either emtricitabine or tenofovir DF were detected in any patient.

No patients in the emtricitabine + tenofovir DF group with non‑B subtype HIV‑1 isolates met resistance analysis criteria by Week 48 (i.e., had confirmed plasma HIV‑1 RNA > 400 copies/mL by Week 48 or early study drug discontinuation). One patient in the Combivir group (subtype AG isolate) met resistance analysis criteria and developed efavirenz resistance and the M184V mutation.

In the small population of patients coinfected with HIV and hepatitis B virus (HBV), no emergence of HBV mutations that could be associated with emtricitabine + tenofovir DF therapy or Combivir therapy was observed through Week 48.

In summary, in antiretroviral treatment-naive HIV‑1 infected patients treated for 48 weeks, the once‑daily regimen of emtricitabine, tenofovir DF, and efavirenz administered without regard to food demonstrated superior antiviral efficacy relative to the regimen of Combivir and efavirenz. Through 48 weeks, development of resistance to efavirenz was the most common form of resistance that developed in both treatment groups of the study. Resistance to emtricitabine (M184V/I mutation) developed infrequently, and no resistance to tenofovir DF (K65R mutation) developed. No novel mutations resulting in phenotypic resistance to either tenofovir DF or emtricitabine developed by Week 48.

### Safety Results

Adverse Events

Up to Week 48, treatment-emergent adverse events (AEs) were reported in 484 patients: 94% of patients (242/257) in the emtricitabine + tenofovir DF group and 95% of patients (242/254) in the Combivir group. Treatment-emergent AEs reported up to Week 48 in at least 10% of patients in either treatment group are summarized by preferred term using the Medical Dictionary for Regulatory Activities (MedDRA, Version 7.1) in decreasing order of frequency for the emtricitabine + tenofovir DF group in Table 4.

Table 4. Treatment-Emergent Adverse Events Reported in at Least 10% of Patients in Either Treatment Group in Study GS-01-934 (Week 48 Safety Analysis Set)

| Adverse Event (Preferred Term)^a^ | EFV + FTC + TDF (N = 257) | | EFV + Combivir (N = 254) | |
| --- | --- | --- | --- | --- |
|  | n | % | n | % |
| Any Event | 242 | 94% | 242 | 95% |
| Dizziness | 71 | 28% | 70 | 28% |
| Nausea | 61 | 24% | 80 | 31% |
| Diarrhea | 52 | 20% | 34 | 13% |
| Abnormal Dreams | 43 | 17% | 34 | 13% |
| Fatigue | 35 | 14% | 36 | 14% |
| Headache | 37 | 14% | 37 | 15% |
| Insomnia | 35 | 14% | 43 | 17% |
| Nasopharyngitis | 35 | 14% | 19 | 7% |
| Rash | 35 | 14% | 27 | 11% |
| Upper Respiratory Tract Infection | 26 | 10% | 20 | 8% |
| Depression | 21 | 8% | 29 | 11% |
| Vomiting | 14 | 5% | 25 | 10% |

a All adverse events reported in ≥ 10% of patients in either treatment group are included, regardless of severity grade or relationship to treatment. Each patient is counted only once per treatment and preferred term.

Source: GS‑01‑934 Clinical Study Report

The most frequently reported AEs in the emtricitabine + tenofovir DF group were dizziness (28%), nausea (24%), and diarrhea (20%). In the Combivir group, the most frequently reported AEs were nausea (31%), dizziness (28%), and insomnia (17%). In both treatment groups, the most frequently reported treatment-emergent AEs assessed as related to study drugs were nausea (11% in the emtricitabine + tenofovir DF group and 26% in the Combivir group) and diarrhea (6% in both groups).

Anemia (including decreased hemoglobin) was reported as a study drug‑related AE for 6% of patients in the Combivir group and was not reported in any patients in the emtricitabine + tenofovir DF group. Grade 3 or 4 anemia, the only treatment-emergent Grade 3 or 4 AE reported in more than 1% of patients in either treatment group, was reported in 4% of patients in the Combivir group and no patients in the emtricitabine + tenofovir DF group. Of the ten Grade 3 or 4 anemia events, 7 were reported as serious adverse events (SAEs).

The overall incidence of treatment-emergent SAEs was similar between the two treatment groups (8% in the emtricitabine + tenofovir DF group and 9% in the Combivir group). Anemia was the only SAE considered by the investigator to be possibly or probably related to study drugs (3% of patients in the Combivir group and no patients in the emtricitabine + tenofovir DF group).

Up to Week 48, 3 deaths have occurred in this study; one death (emtricitabine + tenofovir DF group) was due to Kaposi sarcoma, one death (Combivir group) was due to progressive multifocal leukoencephalopathy, and one death (Combivir group) was due to a suspected drug overdose. All deaths were assessed by the investigator as not related to study drugs.

AEs leading to study drug discontinuation occurred in a significantly smaller percentage of patients in the emtricitabine + tenofovir DF group compared with the Combivir group (4% vs. 9%, p = 0.019). The most frequently occurring AE leading to study drug discontinuation was anemia, including decreased hemoglobin (6% of patients in the Combivir group and no patients in the emtricitabine + tenofovir DF group).

Adverse Events of Special Interest

Renal Function

There was no evidence of a tenofovir DF effect on renal function, as measured by changes from baseline or maximum graded toxicity of serum creatinine or serum phosphorus concentrations. No confirmed (2 consecutive values at least 1 day apart) graded postbaseline laboratory toxicities of serum creatinine elevation or serum phosphorus decrease were reported among patients receiving tenofovir DF. No patient discontinued study drugs because of a renal AE.

Bone Fractures

The incidence of bone fractures at Week 48 was similar between the two treatment groups (3 in the emtricitabine + tenofovir DF group and 2 in the Combivir group). All fractures were considered by the investigator to be unrelated to any of the study drugs, and no change in study regimen dosing was made as a result of any fracture.

Skin Discoloration

AEs of skin hyperpigmentation were reported for 16 patients (11 in the emtricitabine + tenofovir DF group and 5 in the Combivir group). Of the 16 cases of hyperpigmentation, 5 were assessed by a dermatologist as related or probably related to study drugs (4 in the emtricitabine + tenofovir DF group and 1 in the Combivir group); the remainder were assessed as not related to study drugs, were pending dermatologist assessment, or were not assessable (because the patient refused dermatologist evaluation). All hyperpigmentation events (except one Grade 2 event in the Combivir group, pending assessment) were of Grade 1 severity.

Laboratory Abnormalities

The mean increases in fasting total serum cholesterol and fasting serum low‑density lipoprotein concentrations from baseline to Week 48 were significantly smaller for the emtricitabine + tenofovir DF group relative to the Combivir group (p < 0.001 and p = 0.013, respectively).

## Study M02-418

Study M02‑418 was a 96‑week, randomized, open‑label, multicenter study to evaluate the safety, tolerability, pharmacokinetics, and antiviral activity of once‑daily compared with twice‑daily dosing of lopinavir/ritonavir (800 mg/200 mg once daily vs. 400 mg/100 mg twice daily) in combination with emtricitabine (200 mg) and tenofovir DF (300 mg) in the treatment of antiretroviral-naive, HIV‑infected patients. Patients were randomized in a 3:2 ratio to the once‑daily and twice‑daily lopinavir/ritonavir groups, respectively. All patients also received tenofovir DF 300 mg and emtricitabine 200 mg once daily.

The primary efficacy variable was the proportion of patients with a plasma HIV‑1 RNA concentration < 50 copies/mL at Week 48 on the basis of an intent-to-treat (ITT) analysis, in which noncompleters were considered nonresponders (NC = F). This estimate was provided for each treatment group, in addition to the corresponding 95% CI for the difference in proportions (once‑daily group minus twice‑daily group), based on the normal approximation to the binomial distribution. If the lower limit of the CI was above −20%, the once‑daily group was to be considered noninferior to the twice‑daily group.

### Demographics and Baseline Characteristics

Of the 190 patients enrolled and treated, 22% were female and 46% were nonwhite. Their mean age at the beginning of the study was 38.6 years (range, 19 to 75 years). Approximately 45% of patients had baseline CD4+ cell count < 200 cells/mm^3^ and 38% had baseline plasma HIV‑1 RNA > 100,000 copies/mL. The mean time since diagnosis of HIV‑1 infection was 2.3 years (range, 0.1 to 18.5 years).

### Efficacy Results

At Week 48, in the ITT NC = F analysis, 70% of the patients in the once‑daily group and 64% of patients in the twice‑daily group achieved plasma HIV‑1 RNA concentrations < 50 copies/mL. Through Week 96, the virologic response remained similar; 57% of patients in the once‑daily group and 53% of patients in the twice‑daily group achieved plasma HIV‑1 RNA concentrations < 50 copies/mL (ITT NC = F). The proportion of patients with plasma HIV‑1 RNA concentrations < 50 copies/mL at Week 48 and at Week 96 is presented by analysis type in Table 5.

Table 5. Proportion of Patients with Plasma HIV-1 RNA Levels < 50 Copies/mL at Week 48 and Week 96 (Study M02-418)

|  | LPV/r Once Daily + FTC + TDF | | LPV/r Twice Daily + FTC + TDF | | Difference Once Daily − Twice Daily (95% CI) |
| --- | --- | --- | --- | --- | --- |
| **Week 48** |  |  |  |  |  |
| ITT (NC = F)^a^ | 81/115 | 70% | 48/75 | 64% | 6% (−7%, 20%) |
| ITT (M = F)^b^ | 80/115 | 70% | 47/75 | 63% | 7% (−7%, 21%) |
| Observed data^c^ | 80/89 | 90% | 47/54 | 87% | 3% (−8%, 14%) |
| **Week 96** |  |  |  |  |  |
| ITT (NC = F)^a^ | 66/115 | 57% | 40/75 | 53% | 4% (−10%, 19%) |
| ITT (M = F)^b^ | 66/115 | 57% | 40/75 | 53% | 4% (−10%, 19%) |
| Observed data^c^ | 66/74 | 89% | 40/44 | 91% | 2% (−13%, 9%) |

LPV/r, lopinavir/ritonavir; FTC, emtricitabine; TDF, tenofovir disoproxil fumarate; CI, confidence interval

a Intent-to-treat (ITT) noncompleter equals failure (NC = F) analysis: Any patient with a missing value at a visit was considered to have plasma HIV‑1 RNA > 50 copies/mL unless the values immediately preceding and immediately following were < 50 copies/mL.

b ITT missing equals failure (M = F) analysis: Any patient with a missing value for any reason at a given visit was considered > 50 copies/mL.

c Observed data: Missing values were excluded from the analysis.

Source: 96‑Week M02‑418 Clinical Study Report

The 48‑week response rates demonstrated for the regimen of lopinavir/ritonavir plus emtricitabine and tenofovir DF are consistent with those previously reported in Abbott Laboratories Study M98‑863 for a regimen of lopinavir/ritonavir and two NRTIs (lamivudine and stavudine) {3504}. A comparison of virologic response rates for these regimens is shown in Table 6.

Table 6. Comparison of Response Rates in Treatment-Naive Patients Treated With Lopinavir/Ritonavir + 2 NRTI Regimens

| Study | Treatment Group | HIV-1 RNA < 50 copies/mL at Week 48 (ITT NC = F) |
| --- | --- | --- |
| Study M02-418 | LPV/r Once Daily + FTC + TDF (N = 115) | 70% |
|  | LPV/r Twice Daily + FTC + TDF (N = 75) | 64% |
| Abbott Study M98-863 | LPV/r Twice Daily + 3TC + d4T (N = 326) | 67% |

ITT, intent-to-treat; NC = F, noncompleter equals failure; LPV/r, lopinavir/ritonavir; FTC, emtricitabine; TDF, tenofovir disoproxil fumarate; 3TC, lamivudine; d4T, stavudine

Source: 48‑Week M02‑418 Clinical Study Report, {3504}

In the analysis of Study M02‑418 based on the United States (US) Food and Drug Administration (FDA) time-to-loss-of-virologic-response (TLOVR) algorithm, 71% of patients in the once‑daily group and 65% of patients in the twice‑daily group achieved and maintained confirmed plasma HIV‑1 RNA levels < 50 copies/mL through 48 weeks (treatment difference once‑daily minus twice‑daily, 6%; 95% confidence interval [CI], −8%, 20%). Similarly, through 96 weeks, the response rate was 57% for the once‑daily group and 55% for the twice‑daily group (treatment difference, 2%; 95% CI, −11.7%, 17.2%).

Proportions of patients in the once‑daily group with plasma HIV‑1 RNA < 50 copies/mL were numerically higher in the subgroup of patients with low baseline CD4+ cell count (< 200 cells/mm^3^) at Week 48 and Week 96 (Table 7).

Table 7. Proportion of Patients with Plasma HIV-1 RNA Levels < 50 Copies/mL at Week 48 and Week 96 by Baseline HIV-1 and CD4+ Cell Count (ITT NC = F, Study M02-418)

| Response Rate | LPV/r Once Daily + FTC + TDF | | LPV/r Twice Daily + FTC + TDF | |
| --- | --- | --- | --- | --- |
| **Week 48** |  |  |  |  |
| Plasma HIV RNA < 100,000 copies/mL | 43/64 | 67% | 35/53 | 66% |
| Plasma HIV RNA > 100,000 copies/mL | 38/51 | 75% | 13/22 | 59% |
| CD4+ cell count < 200 cells/mm^3^ | 40/50 | 80% | 22/36 | 61% |
| CD4+ cell count ≥ 200 cells/mm^3^ | 41/65 | 63% | 26/39 | 67% |
| **Week 96** |  |  |  |  |
| Plasma HIV RNA < 100,000 copies/mL | 38/64 | 59% | 28/53 | 53% |
| Plasma HIV RNA > 100,000 copies/mL | 28/51 | 55% | 12/22 | 55% |
| CD4+ cell count < 200 cells/mm^3^ | 30/50 | 60% | 20/36 | 56% |
| CD4+ cell count ≥ 200 cells/mm^3^ | 36/65 | 55% | 20/39 | 51% |

LPV/r, lopinavir/ritonavir; FTC, emtricitabine; TDF, tenofovir disoproxil fumarate

Source: 96‑Week M02‑418 Clinical Study Report

Statistically significant decreases in mean plasma HIV‑1 RNA levels were seen in both treatment groups as early as the first visit after the baseline evaluation (Week 4) and were maintained at all subsequent visits through Week 48. The mean decrease from baseline to Week 48 was 3.14 log_10_ copies/mL in the once-daily group and 3.00 log_10_ copies/mL in the twice‑daily group. At Week 96, the mean decrease from baseline was 3.09 log_10_ copies/mL in both dosing groups.

At Week 48, the mean change from baseline in CD4+ cell count was 185 cells/mm^3^ in the once‑daily group and 196 cells/mm^3^ in the twice‑daily group. This immunologic benefit is very similar to the mean increase from baseline of 207 cells/mm^3^ reported for the lopinavir/ritonavir group in Study M98‑863 through 48 weeks of therapy {3504}. Statistically significant increases in mean CD4+ cell count were observed in both groups at all visits Figure 2. Results of the 96‑week analysis were consistent with changes observed at 48 weeks; mean increases from baseline to Week 96 in CD4+ cell count were 244 cells/mm^3^ in the once‑daily group and 264 cells/mm^3^ in the twice‑daily group.

Figure 2. Mean Change From Baseline in CD4+ Cell Count Over Time After Initiation of LPV/r Once or Twice Daily Plus FTC + TDF Once Daily (Study M02-418)

| 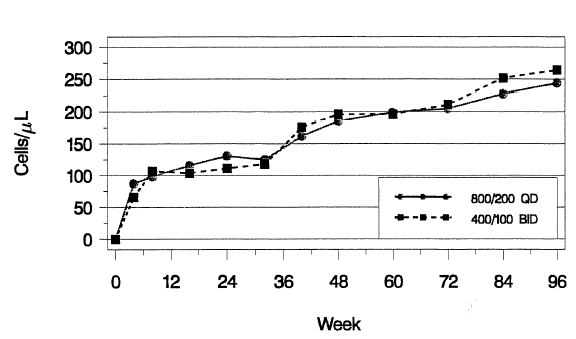 |
| --- |

QD, once daily; BID, twice daily

Source: 96‑Week M02‑418 Clinical Study Report

Virology Results

Genotypic resistance testing was conducted for all available samples in which HIV‑1 RNA was > 500 copies/mL from Weeks 12 to 96. Of the 23 patients with available genotypic resistance data, 4 patients demonstrated an M184V/I mutation in reverse transcriptase, indicating the development of emtricitabine resistance. No development of tenofovir resistance (K65R mutation in reverse transcriptase) was observed, and no other reverse transcriptase mutations suggestive of selection of nucleoside reverse transcriptase inhibitor resistance (positions 41, 44, 62, 65, 67, 69, 70, 74, 115, 118, 210, 215, or 219) emerged in any patient.

In summary, efficacy results for Study M02‑418 demonstrate that a regimen of emtricitabine and tenofovir DF administered with lopinavir/ritonavir (once daily and twice daily) results in significant virological and immunological benefit in antiretroviral therapy–naive patients. Efficacy was consistent with that previously reported in Abbott Study M98‑863 for a regimen of lopinavir/ritonavir and two NRTIs {3504}. Emtricitabine and tenofovir DF administered with lopinavir/ritonavir was effective regardless of baseline antiviral load and CD4+ cell count. The data also indicate a very low HIV‑1 mutation rate in patients experiencing virologic failure, indicating that emtricitabine and tenofovir DF, when used in combination as part of a potent antiretroviral regimen, do not rapidly select for the K65R or the M184V/I mutation.

### Safety Results

Adverse Events

Overall, the AE profile during 96 weeks of treatment in this study was similar in terms of the nature, frequency, and severity of events with that reported in previous trials of lopinavir/ritonavir in combination with NRTIs {7719}.

The most commonly reported possibly or probably related treatment-emergent AEs overall were diarrhea and nausea. Treatment-emergent AEs reported in ≥ 10% of patients overall through Week 96 are summarized by preferred term using the Coding Symbols for Thesaurus of Adverse Reaction Terms (COSTART, 5th Edition) in Table 8. The majority of treatment-emergent AEs were reported by investigators as mild or moderate in severity.

Treatment-emergent AEs of at least moderate severity and probable, possible, or unknown relationship to lopinavir/ritonavir were reported in 53 patients (28%) overall; those reported in > 2% of patients in either group through Week 96 included diarrhea, nausea, vomiting, hyperlipemia, abdominal pain, headache, hypercholesteremia, and amenorrhea, in decreasing order of frequency.

Scrutiny of the AE profile did not identify any new adverse drug reactions potentially associated with the combined use of tenofovir DF and emtricitabine. Frequent AEs (diarrhea, nausea) reported in this study are currently expected in the US package insert for the emtricitabine/tenofovir DF combination tablet. Other frequent unexpected events (e.g., pharyngitis, infection, and fungal dermatitis) are considered typical of the HIV‑1 infected population undergoing antiretroviral therapy, and none were considered by the investigator to be related to study treatment.

Table 8. Treatment-Emergent Adverse Events Reported in at Least 10% of Patients Overall (Week 96, Study M02-418)

|  | Number (%) of Patients | | | | | |
| --- | --- | --- | --- | --- | --- | --- |
| Adverse Event (Preferred Term) | LPV/r Once Daily + FTC + TDF (N = 115) | | LPV/r Twice Daily + FTC + TDF (N = 75) | | Overall (N = 190) | |
| Any Event | 109 | 95% | 69 | 92% | 178 | 94% |
| Diarrhea | 74 | 64% | 29 | 39% | 103 | 54% |
| Nausea | 36 | 31% | 22 | 29% | 58 | 31% |
| Pharyngitis | 31 | 27% | 16 | 21% | 47 | 25% |
| Infection | 19 | 17% | 10 | 13% | 29 | 15% |
| Pain | 19 | 17% | 10 | 13% | 29 | 15% |
| Asthenia | 15 | 13% | 10 | 13% | 25 | 13% |
| Headache | 14 | 12% | 11 | 15% | 25 | 13% |
| Vomiting | 16 | 14% | 8 | 11% | 24 | 13% |
| Rash | 17 | 15% | 6 | 8% | 23 | 12% |
| Depression | 12 | 10% | 7 | 9% | 19 | 10% |
| Fungal Dermatitis | 15 | 13% | 4 | 5% | 19 | 10% |
| Insomnia | 10 | 9% | 8 | 11% | 18 | 9% |
| Anorexia | 5 | 4% | 8 | 11% | 13 | 7% |

LPV/r, lopinavir/ritonavir; FTC, emtricitabine; TDF, tenofovir disoproxil fumarate

Source: 96‑Week M02‑418 Clinical Study Report

Two deaths occurred during the 96‑week treatment period of the study. Each of the events leading to death (lymphoma-like syndrome and AIDS) was considered by the investigator to be not related to study drug.

Treatment-emergent SAEs were reported in a total of 25 patients (12 in the once‑daily group and 13 in the twice‑daily group). SAEs for five patients (liver damage, diarrhea, nephritis, bronchitis and pharyngitis, and hepatitis and immune system disorder) were assessed by the investigator as possibly or probably related to lopinavir/ritonavir.

Clinical AEs that led to premature study drug discontinuation during the 96‑week treatment period of the study occurred in 27 patients. AEs leading to discontinuation were considered possibly or probably related to lopinavir/ritonavir in 22 patients and were, in most cases, gastrointestinal in nature, with diarrhea, nausea, or both being the most common (12 patients). Discontinuations due to these events tended to occur early in the study.

Adverse Events of Special Interest

Renal Function
[truncated: 93,230 more chars]
